# Silylium-Ion-Promoted (3 + 2) Annulation of Allenylsilanes with Internal Alkynes Involving a Pentadienyl-to-Allyl Cation Electrocyclization

**DOI:** 10.1021/jacs.4c09885

**Published:** 2024-11-06

**Authors:** Honghua Zuo, Zheng-Wang Qu, Sebastian Kemper, Hendrik F. T. Klare, Stefan Grimme, Martin Oestreich

**Affiliations:** † Institut für Chemie, Technische Universität Berlin, Straße des 17. Juni 115, 10623 Berlin, Germany; ‡ Mulliken Center for Theoretical Chemistry, Clausius-Institut für Physikalische und Theoretische Chemie, Rheinische Friedrich-Wilhelms-Universität Bonn, Beringstraße 4, 53115 Bonn, Germany

## Abstract

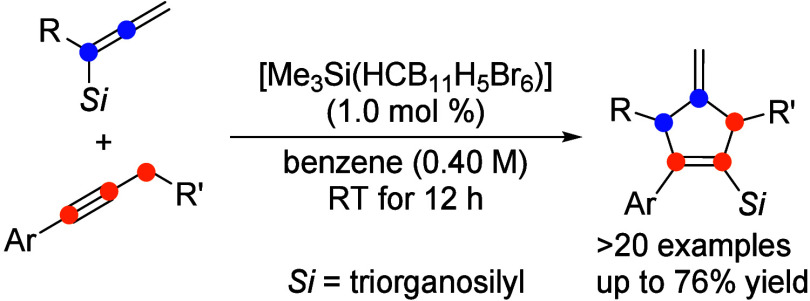

A (3 + 2) annulation of allenyl- and, after rapid isomerization, propargylsilanes with internal 1-aryl-1-alkynes to form 4-methylenecyclopentenes is reported. The reaction is initiated by a silylium ion, and the catalytic cycle is subsequently maintained by the self-regeneration of the silylium-ion promoter. Unlike the well-established Danheiser annulation, where the allenylsilane serves as a *three-carbon synthon*, the present transformation engages the allenylsilane as a *two-carbon synthon*. Experimental observations and DFT calculations unveil a reaction cascade involving various β-silicon-stabilized carbocations, where a pentadienyl-to-allyl cation electrocyclization is the key step.

Allenylsilanes are versatile synthetic reagents^1^ that have been widely employed as propargylic anion equivalents in both Lewis acid- and base-mediated nucleophilic addition to carbonyl electrophiles with notable regiocontrol.^2^ In these reactions, the allenylsilane preferentially reacts with the electrophile at its terminus to formally yield β-silicon-stabilized vinyl cation **I**. This intermediate can also undergo a 1,2-silyl shift to give the regioisomeric vinyl cation **II**, again stabilized by the β-silicon atom (Scheme 1A).^3^ By this, Danheiser as well as Panek and co-workers achieved a variety of (3 + 2) annulations to construct five-membered heterocycles with allenylsilanes participating as *three-carbon synthons*.^4^ Importantly, silylated cyclopentenes emerge from the regiocontrolled (3 + 2) annulation of α,β-unsaturated ketones with allenylsilanes in the presence of a Lewis acid via cationic intermediate **III**, a reaction now known as the Danheiser annulation (Scheme 1B).^5^

**Scheme 1:**
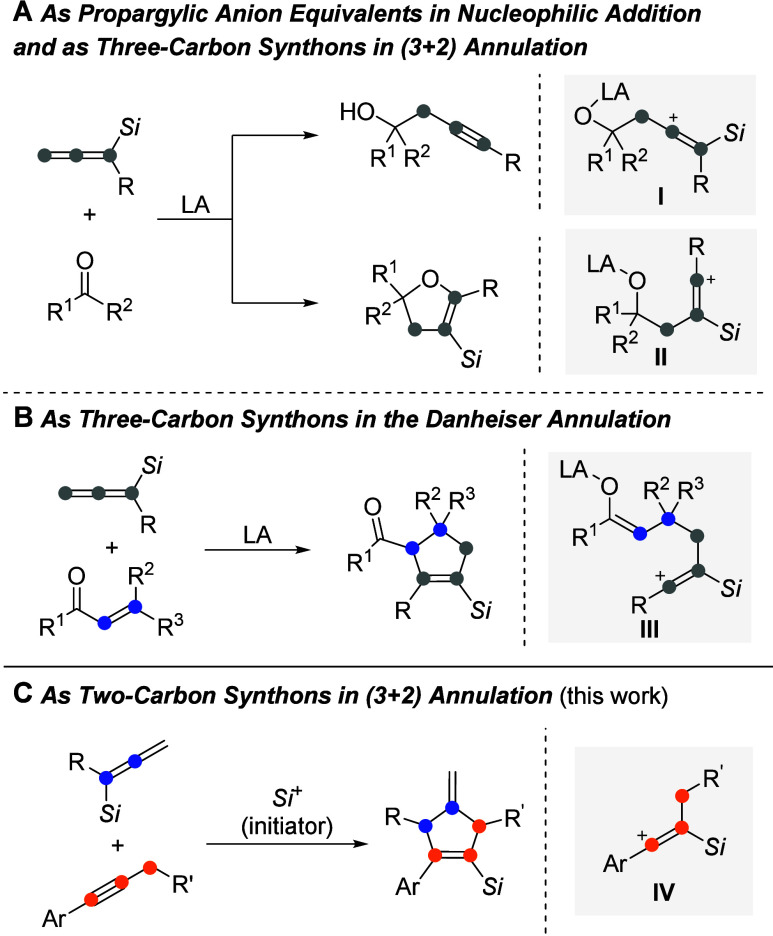
Known and New Chemistry of Allenylsilanes Ar = aryl; any of the R groups = aryl and/or alkyl; *Si* = triorganosilyl; LA = Lewis acid.

Both of the above reaction patterns commence with the nucleophilic addition of allenylsilane to a Lewis acid-activated acceptor. Except for an isolated report,^6^ we are not aware of any further examples that involve the direct nucleophilic addition of an allenylsilane to other carbocation-like intermediates. One such carbon electrophile could be a β-silicon-stabilized vinyl cation **IV** derived from the addition of a silylium ion across a C–C triple bond (Scheme 1C).^7^ Electrophilic alkyne activation by silylium ions is known,^8^ and our laboratory recently demonstrated two cases.^9^ To further exploit cationic adduct **IV** in synthetic methodology with our expertise in silylium ion chemistry,^10^ we decided to probe its reactivity toward allenylsilanes where the silicon moiety could be subsequently released in the form of a silylium ion to eventually close a catalytic cycle.^11^ The net result is a (3 + 2) annulation of allenylsilanes with alkynes with the allenylsilane now serving as a *two-carbon synthon*.

We began our investigation with α-methyl-substituted allenylsilane **1a** and internal alkyne **2a** as model substrates (Table 1). An unexpected reaction outcome was obtained with 1.0 mol % [Me_3_Si(HCB_11_H_5_Br_6_)] as the initiator in benzene as the solvent, affording the annulation product **3aa** in 72% yield (entry 1). A screening of arene solvents showed that the reaction is chiefly dependent on their polarity (entries 2–5). Not surprisingly, other initiators such as Reed’s benzenium ion^12^ (entry 6) and the trityl salt [Ph_3_C][HCB_11_H_5_Br_6_] (entry 7) did initiate the reaction, but both furnished lower yields. To further improve the reaction efficiency, the amount of the alkyne reactant was gradually increased from 1.2 to 2.0 equiv (entries 8–10). At 1.25 equiv of alkyne **2a**, the yield of **3aa** reached a maximum, producing **3aa** in 68% isolated yield (entry 10).

**Table 1:**
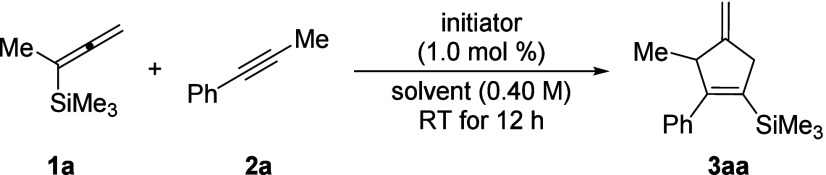
Optimization of Reaction Conditions^a^

entry	initiator	solvent	equiv of **2a**	yield (%)^b^
1	[Me_3_Si(HCB_11_H_5_Br_6_)]	PhH	1.2	72
2	[Me_3_Si(HCB_11_H_5_Br_6_)]	PhF	1.2	37
3	[Me_3_Si(HCB_11_H_5_Br_6_)]	PhCl	1.2	43
4	[Me_3_Si(HCB_11_H_5_Br_6_)]	*o*-C_6_H_4_Cl_2_	1.2	<10
5	[Me_3_Si(HCB_11_H_5_Br_6_)]	PhMe	1.2	41
6	[H(C_6_H_6_)][HCB_11_H_5_Br_6_]	PhH	1.2	51
7	[Ph_3_C][HCB_11_H_5_Br_6_]	PhH	1.2	42
8	[Me_3_Si(HCB_11_H_5_Br_6_)]	PhH	1.25	74^c^^,^^d^
9	[Me_3_Si(HCB_11_H_5_Br_6_)]	PhH	1.5	62
10	[Me_3_Si(HCB_11_H_5_Br_6_)]	PhH	2.0	56

a All reactions were performed on a 0.20 mmol scale under an argon atmosphere in 0.5 mL of the indicated solvent.

b Yields were determined by ^1^H NMR spectroscopy using CH_2_Br_2_ as an internal standard.

c 85% conversion corresponding to 87% yield.

d 68% isolated yield after flash chromatography on silica gel.

With the optimized reaction conditions in hand, we sought to evaluate the generality of this (3 + 2) annulation reaction (Scheme 2, top). The model reaction was run on a 1.0 mmol scale in a slightly diminished yield of 62%. A range of methyl- and halogen-containing substrates **2b**–**j** was tolerated. Yields of the corresponding products **3ab**–**aj** were moderate to good, except for **3af** bearing an *o*-tolyl group. This is likely due to the increased steric hindrance. The molecular structure of **3ae** was determined by X-ray diffraction analysis. Apart from the monosubstituted cases, a decent yield was also recorded for 3,5-dimethyl-substituted product **3ak**. In addition, analogous β-naphthyl-substituted alkyne **2l** was also compatible, furnishing product **3al** in 56% yield. It is noteworthy that applying a thien-2-yl-substituted alkyne was also feasible, although the yield of product **3am** was relatively lower. We attributed this to the competing Lewis pair formation between the sulfur donor and the silylium ion.^9b^ Substituent variation at the alkyne terminus was also possible. Benzyl- and *n*-propyl-substituted alkynes worked equally well, giving rise to products **3an** and **3ao** in 60% and 65% yield, respectively. Unfortunately, non-2-yne as an example of an aliphatic alkyne did not show any reactivity toward this annulation reaction (not shown), presumably because the corresponding alkyl-substituted vinyl cation is energetically not accessible. In turn, the neighboring group effect of the aryl group facilitates vinyl cation formation.^13^ We then varied the substitution pattern at allenylsilane **1** to arrive at methylenecyclopentenes **3** with different side-chain R and silyl groups, respectively. Replacing the methyl group attached to the allenyl moiety by other alkyl groups while keeping the SiMe_3_ part led to the formation of products **3ba** and **3ca** in decent yields. Particularly, an aryl substituent as in **3da** was compatible while internal allenylsilane **1e** was not. Next, we changed the substituents at the silicon atom. Replacing one of the methyl groups in Me_3_Si by an ethyl (**1f**), *n*-butyl (**1g**), or isopropyl (**1h**) group afforded the corresponding products **3fa**–**ha** in moderate to good yields. The trend of decreasing yields is likely due to increasing steric hindrance, and the *t*BuMe_2_Si group (**1j**) was too bulky for the annulation to occur. In addition, product **3ia** bearing a Et_3_Si group brought about a similar result. The messy reaction outcome with a Me_2_PhSi group (**1k**) could originate from known substituent redistribution processes.^14^

**Scheme 2:**
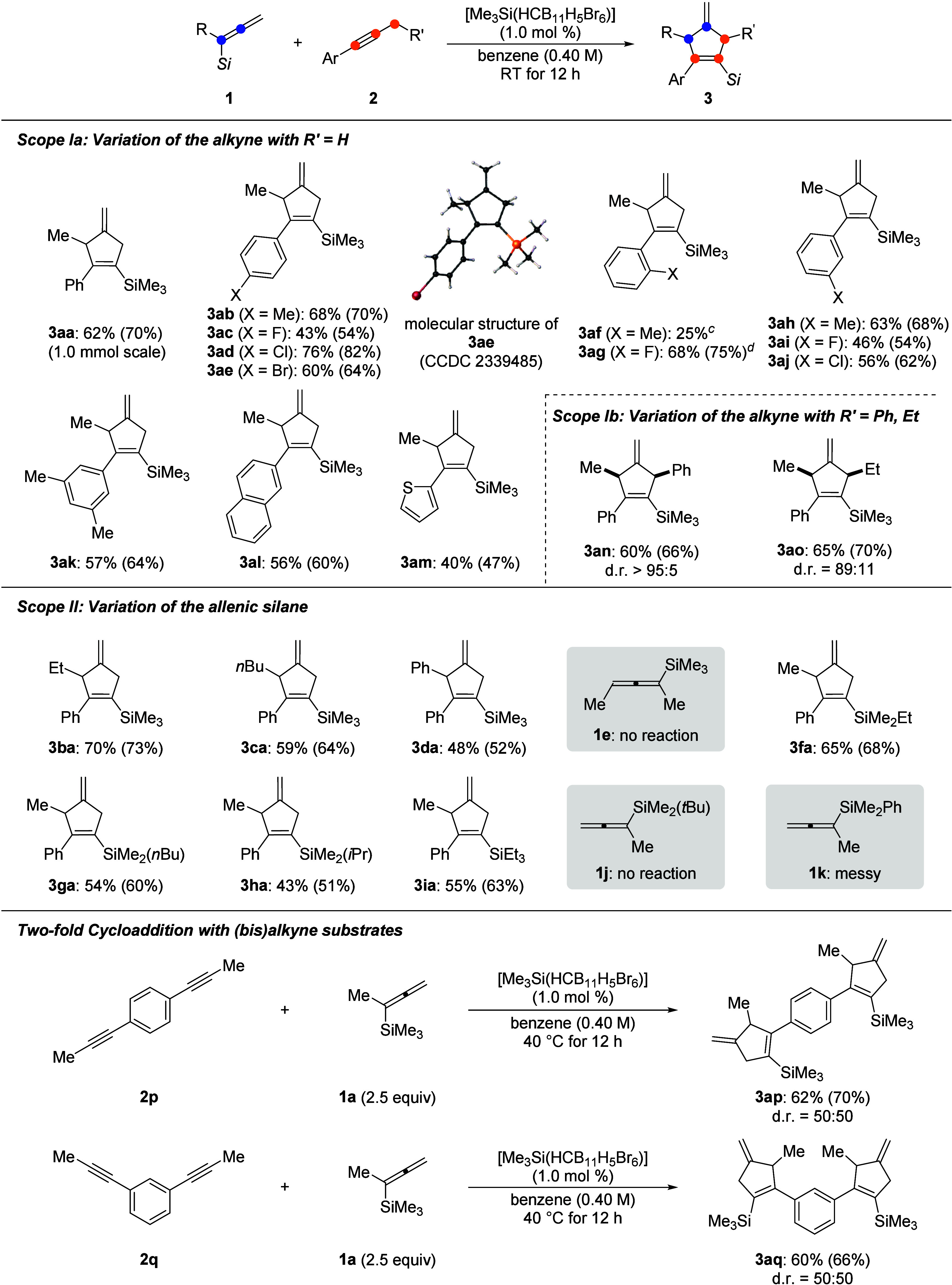
Substrate Scope of the (3 + 2) Annulation^,^ All reactions were performed with allenylsilane **1** (0.20 mmol), alkyne **2** (0.25 mmol, 1.25 equiv) or (bis)alkyne **2p** or **2q** (0.20 mmol), and initiator [Me_3_Si(HCB_11_H_5_Br_6_)] (2.0 μmol, 1.0 mol %) under an argon atmosphere in benzene (0.5 mL) at room temperature for 12 h. Isolated yields refer to analytically pure material after flash chromatography on silica gel; yields in parentheses were determined by ^1^H NMR spectroscopy using CH_2_Br_2_ as an internal standard. The NMR yield is given. The reaction was performed at 40 °C.

This annulation reaction was also applicable to bis(alkyne) substrates (Scheme 2, bottom). A twofold cyclization proceeded smoothly at both sites in the presence of a slight excess of allenylsilane **1a**, affording products **3ap** and **3aq** in synthetically useful yields. However, a 1,3,5-trisalkynylated platform was reluctant to react under these reaction conditions (not shown).

To gain insight into the reaction mechanism, several control experiments were performed (Scheme 3). A deuterium-labeling experiment showed that it is the methyl group in **2a**-*d*_3_ that participates in the annulation reaction, turning the alkyne into a *three-carbon synthon* (eq 1). The incorporation of deuterium in product **3aa**-*d*_3_ suggests that this reaction passes through less obvious rearrangement or migration processes. We then treated allenylsilane **1a** with 1.0 mol % [Me_3_Si(HCB_11_H_5_Br_6_)] in the absence of an alkyne reactant, and propargylsilane **4a** did form in almost quantitative yield within minutes (eq 2). Employing **4a** as the substrate brought about product formation in 60% yield (eq 3). This observation is strong evidence of a mechanism where allenylsilane **1** is isomerized into the corresponding propargylsilane **4**.

**Scheme 3:**
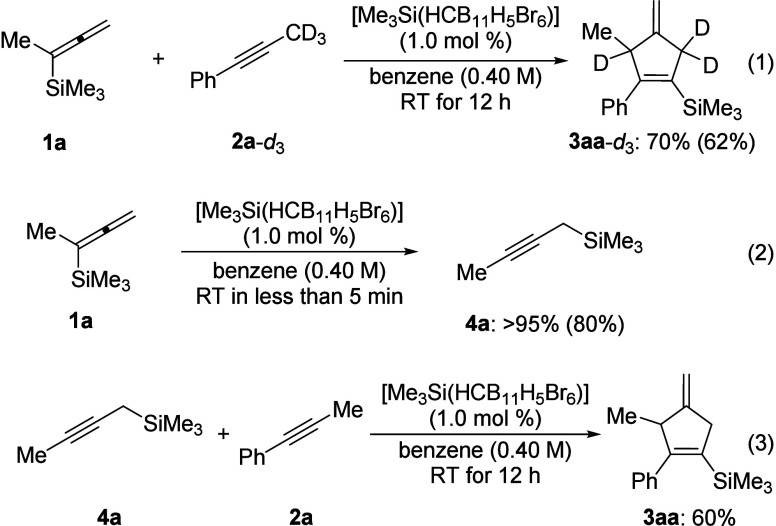
Mechanistic Control Experiments^,^ Yields refer to NMR yields using CH_2_Br_2_ as an internal standard. Isolated yields of **3aa**-*d*_3_ and **4a** after flash chromatography on silica gel are given in parentheses.

We therefore conducted DFT calculations at the PW6B95-D3/def2-QZVP+COSMO-RS//TPSS-D3/def2-TZVP+COSMO level of theory^15^ in benzene solution to elucidate the mechanism of this catalysis. According to the facile isomerization of allenylsilane **1a** to propargylsilane **4a** in the control experiment (Scheme 3, eq 2) and the DFT-computed Me_3_Si^+^ binding free energies (in kcal/mol) in the order **1a** (20.7) ∼ **4a** > **3aa** (15.6) ∼ **2a** (15.4) > [HCB_11_H_5_Br_6_]^−^ (12.4), facile Me_3_Si^+^ transfer from [Me_3_Si(HCB_11_H_5_Br_6_)] to allenylsilane **1a** (or propargylsilane **4a**) leads to **A**^+^ as the actual promoter. The initial fast **A**^+^-mediated isomerization of **1a** to **4a** is slightly exergonic over a low barrier of 18.3 kcal/mol (see Table S2 for details). The final Gibbs free energy profile for the slower **A**^+^-promoted annulation of propargylsilane **4a** with alkyne **2a** is shown in Figure 1. The in situ-formed carbocation **A**^+^ transfers Me_3_Si^+^ selectively to the methyl-substituted alkyne carbon atom of **2a** over a moderate barrier of 20.1 kcal/mol (via **TS1**^+^) to form the carbocation **B**^+^, which is 5.3 kcal/mol endergonic, along with released propargylsilane **4a**. The subsequent addition of **4a** to vinyl cation **B**^+^ is kinetically 1.7 kcal/mol more facile (via **TS2**^+^),^16^ leading to the carbocation **C**^+^ that is slightly exergonic by −0.5 kcal/mol with respect to the initial reactants. Starting from **C**^+^, the [1,5]-hydride shift encounters a barrier of only 8.5 kcal/mol (via **TS3**^+^) to form the low-lying pentadienyl cation **D**^+^. The following electrocyclization of **D**^+^ is again very facile, which is −10.9 kcal/mol exergonic over a low barrier of 10.9 kcal/mol (via **TS4**^+^) to form the 2-cyclopenten-1-yl cation **E**^+^.^17^ As an alternative mechanism, the direct conversion of intermediate **C**^+^ into **E**^+^ by vinyl cation C(sp^3^)–H insertion could be considered.^18^ However, no concerted transition state was found in our DFT calculations, likely due to the conjugate system of **C**^+^, which favors a facile 1,5-H shift and subsequent electrocyclization. A further [1,2]-hydride shift of the cyclic allyl cation **E**^+^ is 4.2 kcal/mol endergonic over a low barrier of 12.4 kcal/mol (via **TS5**^+^) to form the β-silicon-stabilized carbocation **F**^+^. Finally, Me_3_Si^+^ transfer from **F**^+^ to propargylsilane **4a** is −1.8 kcal/mol exergonic over a low barrier of 17.3 kcal/mol (via **TS6**^+^) with respect to low-lying allyl cation **E**^+^, leading to the desired cyclic product **3aa** along with regenerated carbocation **A**^+^ as the propagating silylium-ion species. The (3 + 2) annulation reaction of propargylsilane **4a** with alkyne **2a** promoted by **A**^+^ is −37.6 kcal/mol exergonic to form methylenecyclopentene **3aa** with the initial Me_3_Si^+^ transfer from **A**^+^ to alkyne **2a** (via **TS1**^+^) as the rate-limiting step with an overall barrier of 20.1 kcal/mol.

**Figure 1:**
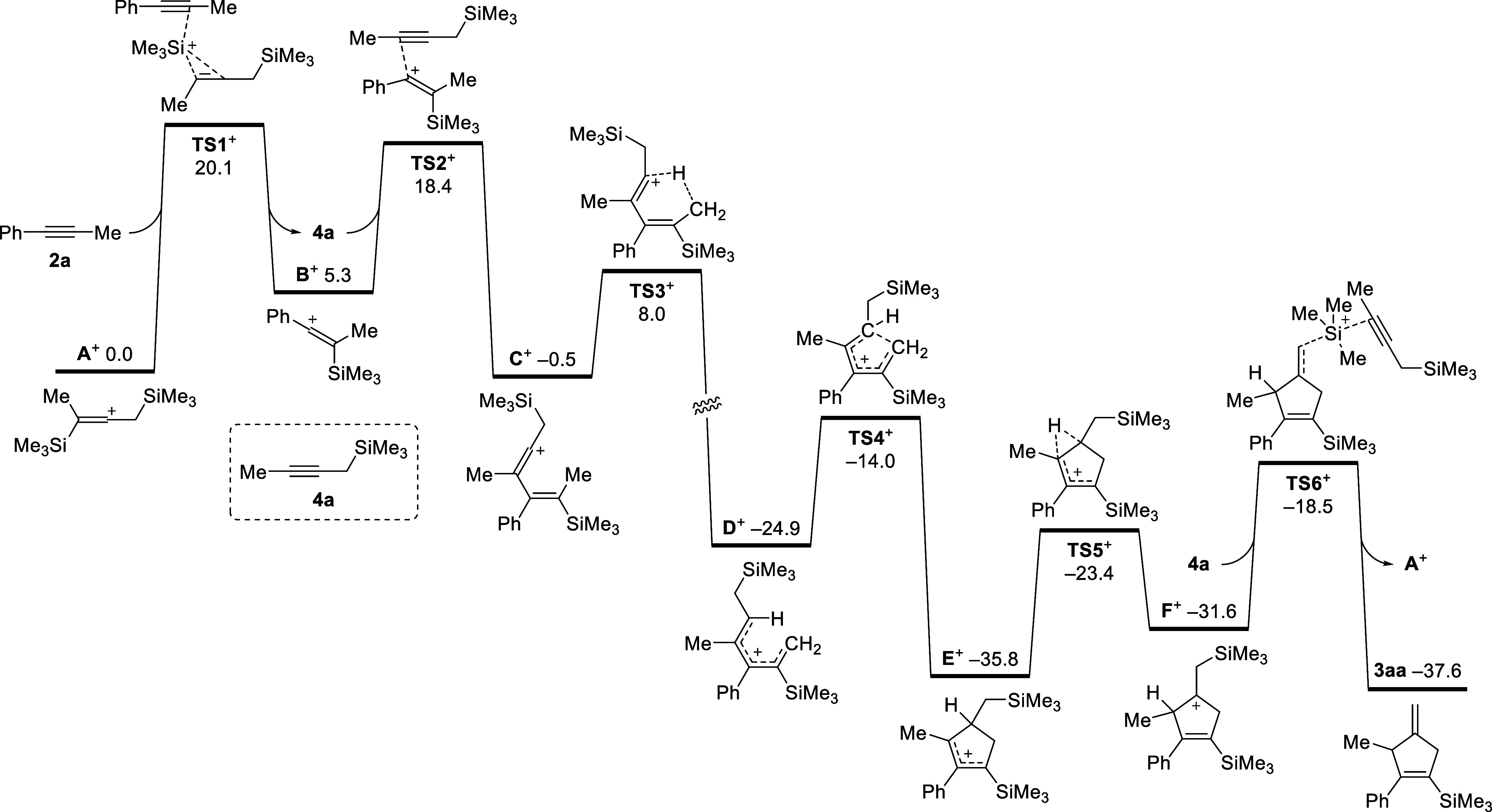
DFT-computed Gibbs free energy profile (in kcal/mol, at 298 K and 1 M concentration) for the (3 + 2) annulation of propargylsilane **4a** with alkyne **2a** catalyzed by in situ-formed carbocation **A**^+^.

Based on these computations and the above control experiments, we propose the catalytic cycle outlined in Scheme 4. It starts with the formation of the β,β′-bis(silicon)-stabilized vinyl cation intermediate **5** from allenylsilane **1**. Site-selective silylium-ion transfer from **5** to alkyne **2** generates propargylsilane **4** along with another β-silicon-stabilized vinyl cation **6**. Nucleophilic attack of the C–C triple bond in **4** results in the formation of vinyl cation **7**, which subsequently undergoes an intramolecular [1,5]-hydride shift to form the pentadienyl cation intermediate **8**. Its conrotatory 4π-electrocyclization^17^ leads to allyl cation **9**. Another intramolecular hydride shift yields yet another β-silicon-stabilized carbocation, namely, tertiary carbenium ion **10**. From this, the annulation product **3** is released by β-elimination of the silylium ion, which in turn is captured by propargylsilane **4**, thereby closing the cycle.

**Scheme 4:**
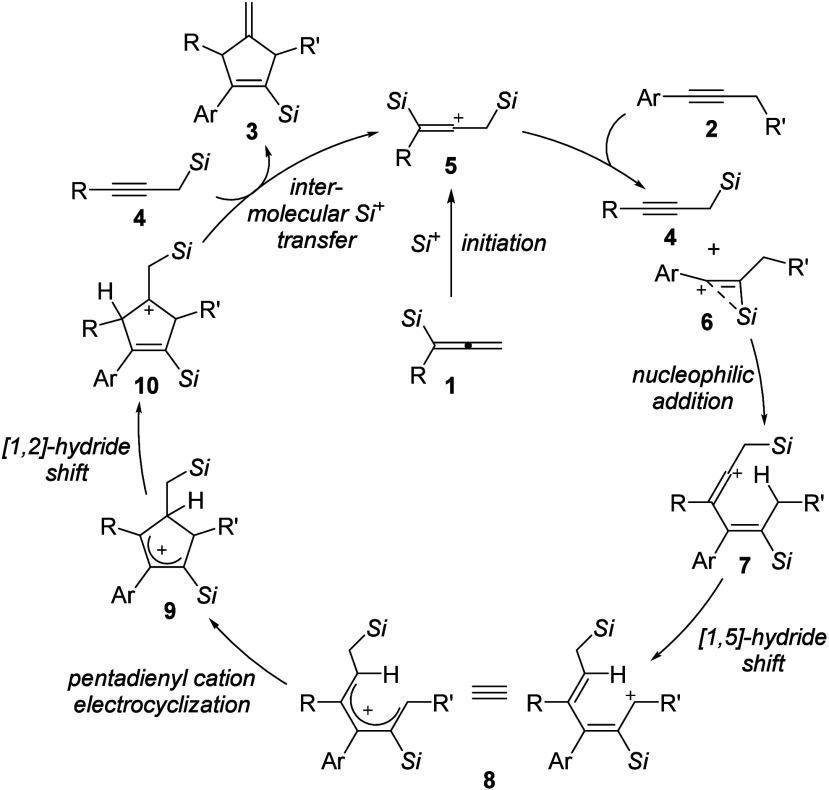
Catalytic Cycle The counteranion [HCB_11_H_5_Br_6_]^−^ has been omitted for clarity.

To illustrate the synthetic value of the annulation products,^19^ we investigated a series of synthetic transformations of **3aa** (Scheme 5). Protodesilylation of **3aa** was readily achieved by the addition of acetic acid to afford the skipped diene **11** in excellent yield.^20^ The exocyclic double bond can be chemoselectively hydrogenated with Wilkinson’s catalyst,^21^ giving product **12** in 88% yield with a diastereomeric ratio of 86:14. We then conducted other addition reactions across the methylene group. A highly diastereoselective hydroboration of **3aa** was accomplished by applying the method developed by Liu and co-workers,^22^ and oxidative degradation of the resulting boronic ester **13** gave primary alcohol **14** in good yield with anti-Markovnikov selectivity. Hydrosilylation of **3aa** with Et_3_SiH in the presence of B(C_6_F_5_)_3_ as the Lewis acid yielded **15** in equally high yield with superb diastereoselectivity.^23^ Moreover, the vinylic silyl group in **15** can be chemoselectively replaced in the presence of the inert C(sp^3^)–Si bond by iododesilylation with *N*-iodosuccinimide (NIS) to give **16**.^24^

**Scheme 5:**
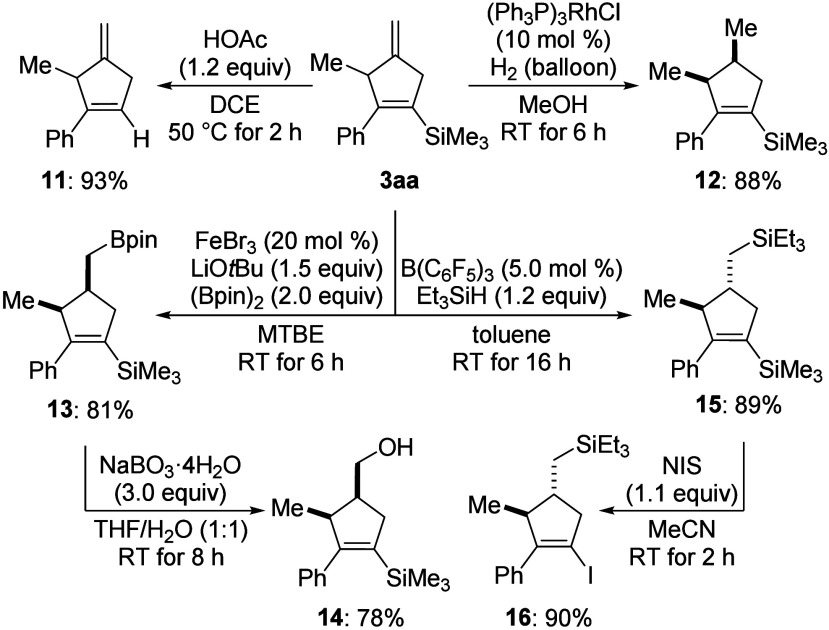
Synthetic Transformations of **3aa** Isolated yields refer to analytically pure material after flash chromatography on silica gel; the diastereomeric ratio (d.r.) was greater than 95:5 throughout, except for **12** with d.r. = 86:14, as verified by ^1^H NMR spectroscopy of the crude reaction mixtures.

In summary, we disclosed here a (3 + 2) annulation of allenyl- and, after rapid isomerization, propargylsilanes with internal 1-aryl-1-alkynes, where the former contributes two and the latter three carbon atoms to the newly formed five-membered carbocycle. This distinguishes the present ring-forming reaction from the well-established Danheiser annulation, where the allenylsilane serves as a *three-carbon synthon* rather than a *two-carbon synthon*. After initiation by a silylium ion, the catalytic cycle is subsequently maintained by self-regeneration of the silylium-ion promoter. The key step is a pentadienyl-to-allyl cation electrocyclization as supported by DFT calculations. The reaction scope is reasonably broad, and the annulation products can be converted into other cyclopentene building blocks.

## Data Availability

The data underlying this study are available in the published article and its Supporting Information.

## Supporting Information Available

Experimental and computational details, characterization data including NMR spectra, and crystallographic data (PDF)
