# Supplementary material for: Silylium-Ion-Promoted (3 + 2) Annulation of Allenylsilanes with Internal Alkynes Involving a Pentadienyl-to-Allyl Cation Electrocyclization
Source: J Am Chem Soc. 2024 Nov 6;146(46):31377–83. doi: 10.1021/jacs.4c09885 (PMC11583337; doi:10.1021/jacs.4c09885)
Supplement: Supplementary file 1 — ja4c09885_si_001.pdf [file ja4c09885_si_001.pdf]

Silylium-Ion-Promoted (3+2) Annulation of Allenylsilanes with Internal Alkynes Involving a Pentadienyl-to-Allyl Cation Electrocyclization

Honghua Zuo,† Zheng-Wang Qu,*,‡ Sebastian Kemper,† Hendrik F. T. Klare,† Stefan Grimme,‡ and Martin Oestreich*,†

†Institut für Chemie, Technische Universität Berlin, Straße des 17. Juni 115, 10623 Berlin, Germany

‡Mulliken Center for Theoretical Chemistry, Institut für Physikalische und Theoretische Chemie, Rheinische Friedrich-Wilhelms-Universität Bonn, Beringstraße 4, 53115 Bonn, Germany

qu@thch.uni-bonn.de

martin.oestreich@tu-berlin.de

Supporting Information

Table of Contents

1 General Information S3

2 Preparation of Reed's Ion-Like Carborate Salts S4

3 Optimization of the Reaction Conditions S5

4 Experimental Details for the Substrate Synthesis S6

4.1 Synthesis of Allenylsilanes (GP 1) S6

4.2 Synthesis of Allenylsilanes (GP 2) S6

4.3 Characterization Data for Allenylsilanes **1** S7

4.4 Synthesis of Internal Alkynes S11

4.5 Characterization Data for Internal Alkynes **2** S13

5 Experimental Details for the Silylium-Ion-Promoted (3+2) Annulation of Allenylsilanes with Internal Alkynes S15

5.1 General Procedure for the (3+2) Annulation (GP 3) S15

5.2 Characterization Data for Annulation Products **3** S16

6 Mechanistic Control Experiments S28

7 Experimental Details for the Synthetic Transformations of 3aa S32

8 Determination of the Relative Configuration S38

9 Crystallographic Data S42

10 NMR Spectra S44

11 Computational Data S156

12 References S172

1 General Information

All reactions were performed in flame-dried glassware using an MBraun glovebox (O_2 < 0.5 ppm, H_2O < 1.0 ppm) or conventional Schlenk techniques under a static pressure of argon (glovebox) or nitrogen (fume hood) unless otherwise stated. All given elevated temperatures refer to external oil bath temperatures. Standard solvents and reagents were obtained from commercial suppliers and used as received unless otherwise stated. Glassware was dried overnight at 150 °C or flame dried using a heat gun. All plastic syringes and needles used in the glovebox were dried overnight at 60 °C. Liquids and solutions were transferred with syringes. Technical grade solvents for extraction and chromatography were distilled prior to use. Tetrahydrofuran (THF) was dried over sodium and freshly distilled prior to use. Dry benzene (C_6H_6) and *n*-pentane were obtained from an MBraun solvent purification system (SPS-800), degassed by three freeze-pump-thaw cycles, and stored in a glovebox over thermally activated 4 Å molecular sieves. Dichloromethane (CH_2Cl_2), toluene (C_7H_8), fluorobenzene (C_6H_5F), chlorobenzene (C_6H_5Cl), bromobenzene (C_6H_5Br), and 1,2-dichlorobenzene (1,2-$C_6H_4Cl_2$) were dried over CaH_2, distilled, degassed by three freeze-pump-thaw cycles, and stored in a glovebox over thermally activated 4 Å molecular sieves. Thin-layer chromatography (TLC) was performed on Macherey-Nagel Alugram® Xtra SIL G/UV254 silica gel 60 pre-coated aluminum-backed plates (200 µm layer thickness). Product spots were visualized under UV light (λ_{max} = 254 nm) and with a ceric ammonium molybdate stain. Column chromatography was performed on Grace 60 (40–63 µm, 230–400 mesh, ASTM) silica gel. ^{1}H, ^{13}C, ^{19}F, and ^{29}Si NMR spectra were recorded in $CDCl_3$ on a Bruker AV400, AV500 and AV700 instrument, respectively. Chemical shifts are reported in parts per million (ppm) and are referenced to the residual solvent resonance as the internal standard ($CHCl_3$: δ = 7.26 ppm for ^{1}H NMR and $CDCl_3$: δ = 77.16 ppm for ^{13}C NMR). ^{19}F and ^{29}Si NMR spectra are referenced in compliance with the unified scale for NMR chemical shifts as recommended by the IUPAC stating the chemical shift relative to CCl_3F and TMS, respectively.[S1] Data are reported as follows: chemical shift, multiplicity (s = singlet, d = doublet, t = triplet, q = quartet, sept = septet, m = multiplet, br = broad), coupling constants (Hz), and integration. Infrared (IR) spectra were recorded on an Agilent Technologies Cary 630, and the signals are reported in wavenumbers (cm^{-1}). Data for the single crystal structure determination were collected with an Agilent SuperNova diffractometer equipped with a CCD area Atlas detector and a mirror monochromator by utilizing Cu-K_α radiation (λ = 1.5418 Å). Melting points (m.p.) were determined with a Stuart Scientific SMP20 melting point apparatus and were not corrected. High resolution mass spectra (HRMS) were obtained from the Center for Mass Spectrometry at the Institut für Chemie, Technische Universität Berlin on a Thermo Fisher Scientific LTQ Orbitrap XL apparatus using APCI, ESI or LIFDI techniques with a linear ion trap analyzer.

2 Preparation of Reed's Ion-Like Carborate Salts

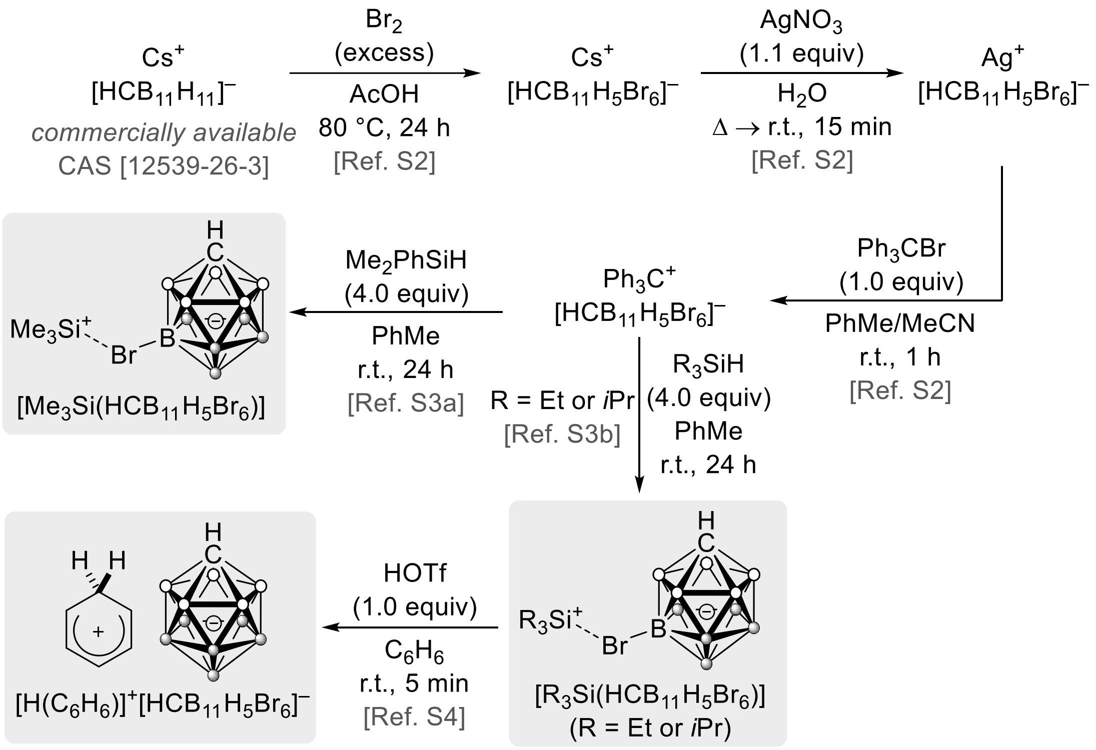

Scheme S1. Preparation of Reed's ion-like carborate salts

3 Optimization of the Reaction Conditions[a]

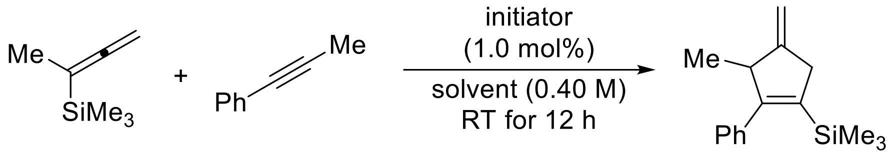

1a **2a** **3aa**

entry	initiator	solvent	**2a** (equiv)	yield (%)[b]
1	[$Me_3Si(HCB_{11}H_5Br_6)$]	PhH	1.2	72
2	[$Me_3Si(HCB_{11}H_5Br_6)$]	PhF	1.2	37
3	[$Me_3Si(HCB_{11}H_5Br_6)$]	PhCl	1.2	43
4	[$Me_3Si(HCB_{11}H_5Br_6)$]	PhBr	1.2	40
5	[$Me_3Si(HCB_{11}H_5Br_6)$]	1,2-$C_6H_4Cl_2$	1.2	< 10
6	[$Me_3Si(HCB_{11}H_5Br_6)$]	PhMe	1.2	41
7	[$Me_3Si(HCB_{11}H_5Br_6)$]	*p*-xylene	1.2	46
8	[$Me_3Si(HCB_{11}H_5Br_6)$]	$PhCF_3$	1.2	< 10
9	[$Me_3Si(HCB_{11}H_5Br_6)$]	*n*-hexane	1.2	NR
10	[$Me_3Si(HCB_{11}H_5Br_6)$]	CH_2Cl_2	1.2	trace
11	[$Et_3Si(HCB_{11}H_5Br_6)$]	PhH	1.2	68
12	[*i*$Pr_3Si(HCB_{11}H_5Br_6)$]	PhH	1.2	16
13	[Et_3Si(toluene)][$B(C_6F_5)_4$]	PhH	1.2	54
14	[$H(C_6H_6)$][$HCB_{11}H_5Br_6$]	PhH	1.2	51
15	[Ph_3C][$HCB_{11}H_5Br_6$]	PhH	1.2	42
16	$AlCl_3$	PhH	1.2	trace
17	TfOH	PhH	1.2	NR
18	[$Me_3Si(HCB_{11}H_5Br_6)$]	PhH	1.25	74 (68)[c]
19	[$Me_3Si(HCB_{11}H_5Br_6)$]	PhH	1.5	62
20	[$Me_3Si(HCB_{11}H_5Br_6)$]	PhH	2.0	56

[a]All reactions were performed on a 0.20 mmol scale under argon atmosphere in 0.5 mL of indicated solvent. [b]Yields were determined by ^1H NMR spectroscopy using CH_2Br_2 as an internal standard. [c]Isolated yield after flash chromatography on silica gel is given in parentheses.

4 Experimental Details for the Substrate Synthesis

4.1 Synthesis of Allenylsilanes (GP 1)

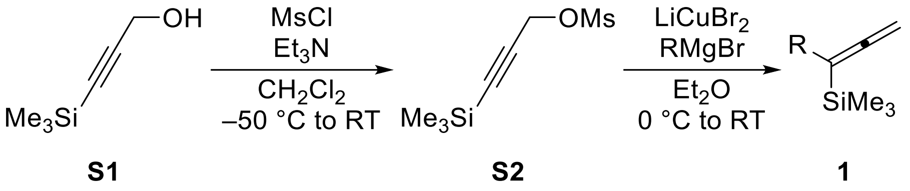

Step 1: According to a reported procedure,[S5] propargyl alcohol **S1** (10.0 mmol, 1.0 equiv) and Et_3N (15.0 mmol, 1.52 g, 1.5 equiv) are dissolved in CH_2Cl_2 (60 mL) and cooled to −50 °C. Then, MsCl (12.0 mmol, 1.37 g, 1.2 equiv) is added dropwise to this solution. After stirring for 30 min, the resulting mixture is gradually warmed to room temperature and stirred for additional 2 h. Upon completion, the reaction mixture is poured into water (50 mL), and the aqueous phase is extracted with CH_2Cl_2 (3 × 20 mL). The combined organic phases are dried over Na_2SO_4 and concentrated under reduced pressure to afford pure mesylate **S2** as a light yellow oil, which is directly used in the next step without further purification.

Step 2: A solution of CuBr (12.0 mmol, 1.72 g, 1.2 equiv) and LiBr (12.0 mmol, 1.04 g, 1.2 equiv) in Et_2O (20 mL) is cooled to 0 °C. Freshly prepared RMgBr (12.0 mmol, 1.2 equiv) is then slowly added to this mixture. After stirring at 0 °C for 30 min, mesylate **S2** (dissolved in 5.0 mL of Et_2O) is added, and the resulting mixture is gradually warmed to room temperature and stirred for additional 12 h. Upon completion, the reaction mixture is quenched by the addition of saturated NH_4Cl solution (20 mL), and the aqueous phase is extracted with Et_2O (3 × 15 mL). The combined organic phases are dried over Na_2SO_4 and carefully concentrated under reduced pressure (40 °C, 750 mbar). Purification of the residue by flash column chromatography on silica gel using *n*-pentane as the eluent affords the corresponding allenylsilane **1** as a colorless oil.

4.2 Synthesis of Allenylsilanes (GP 2)

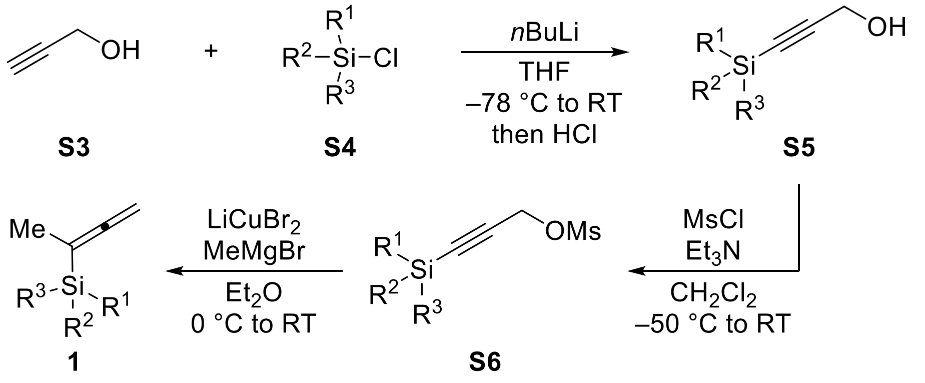

Step 1: According to a reported procedure,[S6] propargyl alcohol (**S3**, 10.0 mmol, 561 mg,

1.0 equiv) is dissolved in THF (30 mL) and cooled to −78 °C, followed by the dropwise addition of *n*BuLi (8.8 mL, 2.5 M solution in *n*-hexane, 22.0 mmol, 2.2 equiv). After stirring for 30 min, chlorosilane **S4** (24.0 mmol, 2.4 equiv) is slowly added, and the resulting mixture is gradually warmed to room temperature and stirred for additional 6 h. Upon completion, the reaction mixture is carefully quenched with 2 N HCl solution (20 mL). After stirring for 30 min, the aqueous phase is extracted with CH_2Cl_2 (3 × 20 mL). The combined organic phases are dried over Na_2SO_4 and concentrated under reduced pressure. Purification of the residue by flash column chromatography on silica gel using *n*-pentane/EtOAc (50:1 to 20:1) as the eluent affords the corresponding propargyl alcohol **S5** as a light yellow oil.

Step 2: Propargyl alcohol **S5** (10.0 mmol, 1.0 equiv) and Et_3N (15.0 mmol, 1.52 g, 1.5 equiv) are dissolved in CH_2Cl_2 (60 mL) and cooled to −50 °C. Then, MsCl (12.0 mmol, 1.37 g, 1.2 equiv) is added dropwise to this solution. After stirring for 30 min, the resulting mixture is gradually warmed to room temperature and stirred for additional 2 h. Upon completion, the reaction mixture is poured into water (50 mL), and the aqueous phase is extracted with CH_2Cl_2 (3 × 20 mL). The combined organic phases are dried over Na_2SO_4 and concentrated under reduced pressure to afford pure mesylate **S6** as a light yellow oil, which is directly used in the next step without further purification.

Step 3: A solution of CuBr (12.0 mmol, 1.72 g, 1.2 equiv) and LiBr (12.0 mmol, 1.04 g, 1.2 equiv) in Et_2O (20 mL) is cooled to 0 °C. MeMgBr (12.0 mmol, 1.2 equiv) is then slowly added to this mixture. After stirring at 0 °C for 30 min, mesylate **S6** (dissolved in 5.0 mL of Et_2O) is added, and the resulting mixture is gradually warmed to room temperature and stirred for additional 12 h. Upon completion, the reaction mixture is quenched by the addition of saturated NH_4Cl solution (20 mL). The aqueous phase is extracted with Et_2O (3 × 15 mL), and the combined organic phases are dried over Na_2SO_4 and carefully concentrated under reduced pressure (40 °C, 750 mbar). Purification of the residue by flash column chromatography on silica gel using *n*-pentane as the eluent affords the corresponding allenylsilane **1** as a colorless oil.

4.3 Characterization Data for Allenylsilanes 1

Buta-2,3-dien-2-yltrimethylsilane (1a)

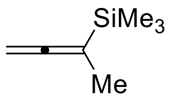

1a
$C_7H_{14}Si$
M = 126.27 g/mol

Prepared from MeMgBr according to **GP 1**. Flash column chromatography on silica gel using *n*-pentane as the eluent afforded substrate **1a** as a colorless oil (1.07 g, 85% yield).

R*f* = 0.80 (cyclohexane, stained with $KMnO_4$). **^{1}H NMR** (500 MHz, $CDCl_3$, 298 K): δ = 4.26 (q, *J* = 3.1 Hz, 2H), 1.69 (t, *J* = 3.1 Hz, 3H), 0.09 (s, 9H) ppm. **^{13}C{^{1}H} NMR** (126 MHz, $CDCl_3$, 298 K): δ = 208.8, 89.4, 67.4, 15.3, −1.9 ppm. **^{29}Si DEPT NMR** (99 MHz, $CDCl_3$, 298 K, optimized for *J* = 7.0 Hz): δ = −4.1 ppm. The NMR spectroscopic data are in accordance with those reported.[S5]

Trimethyl(penta-1,2-dien-3-yl)silane (1b)

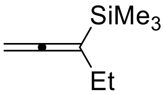

1b
$C_8H_{16}Si$
M = 140.30 g/mol

Prepared from EtMgBr according to **GP 1**. Flash column chromatography on silica gel using *n*-pentane as the eluent afforded substrate **1b** as a colorless oil (1.10 g, 79% yield). **R*f*** = 0.75 (cyclohexane, stained with $KMnO_4$). **IR** (ATR): ṽ = 2960, 2898, 1925, 1630, 1457, 1247, 1083, 1004, 931, 833, 809, 751, 691 cm^{-1}. **^{1}H NMR** (500 MHz, $CDCl_3$, 298 K): δ = 4.37–4.34 (m, 2H), 2.03–1.90 (m, 2H), 1.06 (t, *J* = 7.3 Hz, 3H), 0.10 (s, 9H) ppm. **^{13}C{^{1}H} NMR** (101 MHz, $CDCl_3$, 298 K): δ = 208.2, 96.4, 69.4, 21.9, 13.7, −1.6 ppm. **^{29}Si DEPT NMR** (79 MHz, $CDCl_3$, 298 K, optimized for *J* = 7.0 Hz): δ = −4.8 ppm.

Hepta-1,2-dien-3-yltrimethylsilane (1c)

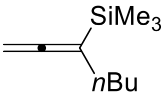

1c
$C_{10}H_{20}Si$
M = 168.36 g/mol

Prepared from *n*BuMgBr according to **GP 1**. Flash column chromatography on silica gel using *n*-pentane as the eluent afforded substrate **1c** as a colorless oil (1.22 g, 73% yield). **R*f*** = 0.70 (cyclohexane, stained with $KMnO_4$). **^{1}H NMR** (500 MHz, $CDCl_3$, 298 K): δ = 4.32 (t, *J* = 3.3 Hz, 2H), 2.00–1.89 (m, 2H), 1.49–1.40 (m, 2H), 1.39–1.30 (m, 2H), 0.91 (t, *J* = 7.3 Hz, 3H), 0.10 (s, 9H) ppm. **^{13}C{^{1}H} NMR** (101 MHz, $CDCl_3$, 298 K): δ = 208.4, 94.7, 68.8, 31.4, 28.6, 22.6, 14.1, −1.5 ppm. **^{29}Si DEPT NMR** (79 MHz, $CDCl_3$, 298 K, optimized for *J* = 7.0 Hz): δ = −4.7 ppm. The NMR spectroscopic data are in accordance with those reported.[S7]

Trimethyl(1-phenylpropa-1,2-dien-1-yl)silane (1d)

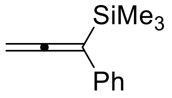

1d
$C_{12}H_{16}Si$
M = 188.35 g/mol

Prepared from PhMgBr according to **GP 1**. Flash column chromatography on silica gel using *n*-pentane as the eluent afforded substrate **1d** as a colorless oil (1.45 g, 77% yield). **R*f*** = 0.73 (cyclohexane). **^{1}H NMR** (500 MHz, $CDCl_3$, 298 K): *δ* = 7.34–7.28 (m, 4H), 7.22–7.16 (m, 1H), 4.68 (s, 2H), 0.26 (s, 9H) ppm. **^{13}C{^{1}H} NMR** (126 MHz, $CDCl_3$, 298 K): *δ* = 211.3, 137.2, 128.6, 127.8, 126.3, 98.8, 70.6, −0.4 ppm. **^{29}Si DEPT NMR** (99 MHz, $CDCl_3$, 298 K, optimized for *J* = 7.0 Hz): *δ* = −5.0 ppm. The NMR spectroscopic data are in accordance with those reported.[S8]

Buta-2,3-dien-2-yl(ethyl)dimethylsilane (1f)

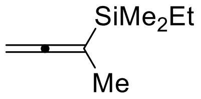

1f
$C_8H_{16}Si$
M = 140.30 g/mol

Prepared from chlorodimethylethylsilane and MeMgBr according to **GP 2**. Flash column chromatography on silica gel using *n*-pentane as the eluent afforded substrate **1f** as a colorless oil (954 mg, 68% yield).
R*f* = 0.75 (cyclohexane, stained with $KMnO_4$). **IR** (ATR): ṽ = 2955, 2913, 2876, 2128, 1931, 1631, 1249, 1055, 957, 809, 776, 695 cm^{-1}. **^{1}H NMR** (500 MHz, $CDCl_3$, 298 K): *δ* = 4.26 (d, *J* = 3.1 Hz, 2H), 1.68 (t, *J* = 3.1 Hz, 3H), 0.94 (t, *J* = 7.9 Hz, 3H), 0.59 (q, *J* = 7.9 Hz, 2H), 0.07 (s, 6H) ppm. **^{13}C{^{1}H} NMR** (126 MHz, $CDCl_3$, 298 K): *δ* = 209.1, 88.4, 67.3, 15.5, 7.4, 6.7, −4.2 ppm. **^{29}Si DEPT NMR** (79 MHz, $CDCl_3$, 298 K, optimized for *J* = 17.0 Hz): *δ* = −1.7 ppm.

Buta-2,3-dien-2-yl(butyl)dimethylsilane (1g)

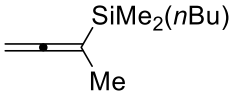

1g
$C_{10}H_{20}Si$
M = 168.36 g/mol

Prepared from chlorodimethylbutylsilane and MeMgBr according to **GP 2**. Flash column chromatography on silica gel using *n*-pentane as the eluent afforded substrate **1g** as a

colorless oil (943 mg, 56% yield).

R*f* = 0.73 (cyclohexane, stained with $KMnO_4$). **IR** (ATR): ṽ = 2956, 2919, 2857, 2190, 1931, 1630, 1376, 1248, 929, 837, 808, 779, 701 cm^{-1}. **^{1}H NMR** (500 MHz, $CDCl_3$, 298 K): *δ* = 4.26 (q, *J* = 3.1 Hz, 2H), 1.68 (t, *J* = 3.1 Hz, 3H), 1.40–1.21 (m, 4H), 0.88 (t, *J* = 6.9 Hz, 3H), 0.67–0.54 (m, 2H), 0.06 (s, 6H) ppm. **^{13}C{^{1}H} NMR** (101 MHz, $CDCl_3$, 298 K): *δ* = 209.0, 88.6, 67.3, 26.6, 26.2, 15.5, 14.6, 14.0, −3.6 ppm. **^{29}Si DEPT NMR** (79 MHz, $CDCl_3$, 298 K, optimized for *J* = 27.0 Hz): *δ* = −3.1 ppm.

Buta-2,3-dien-2-yl(isopropyl)dimethylsilane (1h)

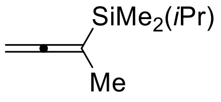

1h
$C_9H_{18}Si$
M = 154.33 g/mol

Prepared from chloro(*iso*propyl)dimethylsilane and MeMgBr according to **GP 2**. Flash column chromatography on silica gel using *n*-pentane as the eluent afforded substrate **1h** as a colorless oil (941 mg, 61% yield).

R*f* = 0.78 (cyclohexane, stained with $KMnO_4$). **IR** (ATR): ṽ = 2953, 2862, 2567, 2025, 1930, 1631, 1461, 1249, 998, 881, 806, 767, 683 cm^{-1}. **^{1}H NMR** (500 MHz, $CDCl_3$, 298 K): *δ* = 4.26 (q, *J* = 3.1 Hz, 2H), 1.69 (t, *J* = 3.1 Hz, 3H), 0.96 (d, *J* = 6.4 Hz, 6H), 0.93–0.81 (m, 1H), 0.04 (s, 6H) ppm. **^{13}C{^{1}H} NMR** (126 MHz, $CDCl_3$, 298 K): *δ* = 209.4, 87.7, 67.3, 17.7, 16.0, 13.4, −5.8 ppm. **^{29}Si DEPT NMR** (99 MHz, $CDCl_3$, 298 K, optimized for *J* = 15.0 Hz): *δ* = 0.2 ppm.

Buta-2,3-dien-2-yltriethylsilane (1i)

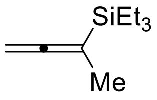

1i
$C_{10}H_{20}Si$
M = 168.36 g/mol

Prepared from chlorotriethylsilane and MeMgBr according to **GP 2**. Flash column chromatography on silica gel using *n*-pentane as the eluent afforded substrate **1i** as a colorless oil (1.29 g, 77% yield).

R*f* = 0.71 (cyclohexane, stained with $KMnO_4$). **IR** (ATR): ṽ = 2953, 2909, 2875, 2411, 2057, 1930, 1629, 1457, 1237, 1006, 928, 809, 717 cm^{-1}. **^{1}H NMR** (500 MHz, $CDCl_3$, 298 K): *δ* = 4.25 (q, *J* = 3.1 Hz, 2H), 1.68 (t, *J* = 3.1 Hz, 3H), 0.95 (t, *J* = 7.9 Hz, 9H), 0.61 (q, *J* = 7.9 Hz, 6H) ppm. **^{13}C{^{1}H} NMR** (126 MHz, $CDCl_3$, 298 K): *δ* = 209.6, 86.4, 67.1, 16.0,

7.5, 3.1 ppm. **^{29}Si DEPT NMR** (99 MHz, $CDCl_3$, 298 K, optimized for J = 31.0 Hz): δ = 1.8 ppm.

4.4 Synthesis of Internal Alkynes

Internal alkynes **2a**–**2j**, **2l**, and **2n** were already prepared and characterized in our previous work on alkyne difunctionalization.[S9] The other alkynes were synthesized according to the following procedures.

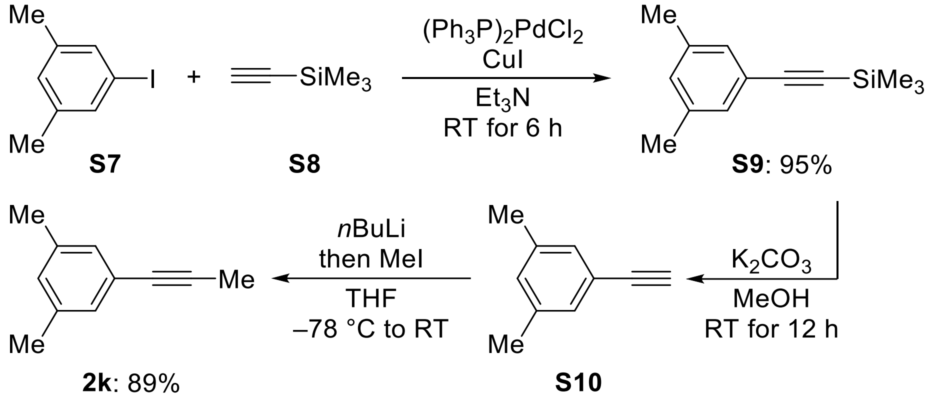

Step 1: According to a reported procedure,[S10] a solution of 3,5-dimethyliodobenzene (**S7**, 5.0 mmol, 1.16 g, 1.0 equiv) and trimethylsilylacetylene (**S8**, 6.0 mmol, 589.3 mg 1.2 equiv) in Et_3N (10 mL) was added dropwise to a suspension of $(Ph_3P)_2PdCl_2$ (42.1 mg, 0.06 mmol, 2.0 mol %) and CuI (17.1 mg, 0.09 mmol, 3.0 mol %) in Et_3N (15 mL). The reaction mixture was maintained for 6 h at ambient temperature. Upon completion of the reaction (monitored by TLC), the reaction was quenched by the addition of saturated aqueous NH_4Cl solution (15 mL). The resulting mixture was extracted with EtOAc (3 × 20 mL), and the combined organic phases were dried over Na_2SO_4 and concentrated under reduced pressure. Purification of the residue by flash column chromatography on silica gel using *n*-pentane as the eluent afforded silylated arylalkyne **S9** as a colorless oil (961.3 mg, 95% yield).

Step 2: K_2CO_3 (9.5 mmol, 1.31 g, 2.0 equiv) was added to a solution of alkyne **S9** (4.75 mmol, 961.2 mg, 1.0 equiv) in MeOH (20 mL). The resulting suspension was stirred at room temperature for 12 h. Upon completion of the reaction (monitored by TLC), the resulting mixture was filtered through a short pad of celite to remove all precipitates. The filtrate was concentrated under reduced pressure to afford 1-ethynyl-3,5-dimethylbenzene (**S10**) as a light yellow oil, which was directly used in the next step without further purification.

Step 3: Alkyne **S10** (4.75 mmol, 618.4 mg, 1.0 equiv) was dissolved in THF (25 mL), and the solution was cooled to –78 °C. Then, *n*BuLi (2.3 mL, 2.5 M solution in *n*-hexane, 5.7 mmol, 1.2 equiv) was added dropwise. After stirring at –78 °C for 30 min, iodomethane (842.8 mg, 5.94 mmol, 1.25 equiv) was slowly added, and the resulting

mixture was allowed to gradually warm to ambient temperature and stirred for additional 6 h. Upon completion (monitored by TLC), the reaction was quenched by the addition of saturated aqueous NH_4Cl solution (5.0 mL). The resulting mixture was extracted with CH_2Cl_2 (3 × 20 mL), and the combined organic phases were dried over Na_2SO_4 and concentrated under reduced pressure. Purification of the residue by flash column chromatography on silica gel using *n*-pentane as the eluent afforded internal alkyne **2k** as a colorless oil (609.7 mg, 89% yield).

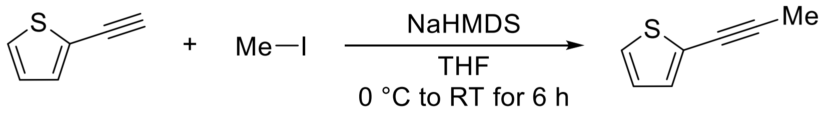

S11 **2m**: 85%

According to a reported procedure,[S11] 2-ethynylthiophene (**S11**, 5.0 mmol, 540.8 mg, 1.0 equiv) was dissolved in THF (25 mL), and the solution was cooled to 0 °C. Then, NaHMDS (3.2 mL, 1.9 M solution in THF, 6.0 mmol, 1.2 equiv) was added dropwise to this solution. After stirring at 0 °C for 30 min, iodomethane (887.1 mg, 6.25 mmol, 1.25 equiv) was added slowly, and the resulting mixture was allowed to gradually warm to ambient temperature and stirred for additional 6 h. Upon completion (monitored by TLC), the reaction was quenched by the addition of saturated aqueous NH_4Cl solution (5.0 mL), and the resulting mixture was extracted with CH_2Cl_2 (3 × 20 mL). The combined organic phases were dried over Na_2SO_4 and concentrated under reduced pressure. Purification of the residue by flash column chromatography on silica gel using *n*-pentane as the eluent afforded internal alkyne **2m** as a pale yellow oil (519.3 mg, 85% yield).

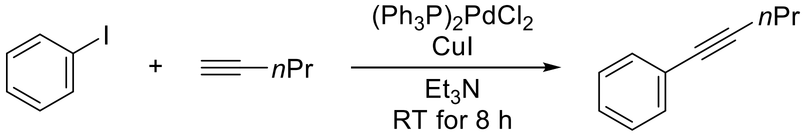

S12 **S13** **2o**: 93%

According to a reported procedure,[S10] a solution of iodobenzene (**S12**, 5.0 mmol, 1.02 g, 1.0 equiv) and pent-1-yne (**S13**, 6.0 mmol, 408.7 mg 1.2 equiv) in Et_3N (5.0 mL) was added dropwise to a suspension of $(Ph_3P)_2PdCl_2$ (42.1 mg, 0.06 mmol, 2.0 mol %) and CuI (17.1 mg, 0.09 mmol, 3.0 mol %) in Et_3N (15 mL). The reaction mixture was stirred at room temperature for additional 8 h. Upon completion (monitored by TLC), the reaction was quenched by the addition of saturated aqueous NH_4Cl solution (15 mL), and the resulting mixture was extracted with EtOAc (3 × 20 mL). The combined organic phases were dried over Na_2SO_4 and concentrated under reduced pressure. Purification of the residue by flash column chromatography on silica gel using *n*-pentane as the eluent afforded internal alkyne **2o** as a colorless oil (670.6 mg, 93% yield).

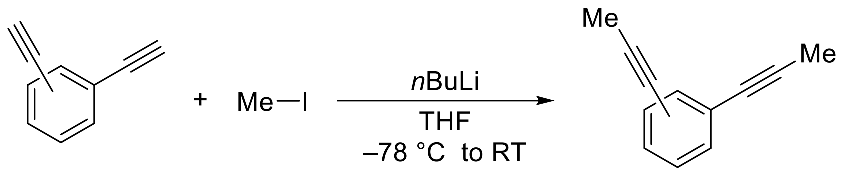

S14 **2p** or **2q**

According to a reported procedure,[S12] the *para*- or *meta*-substituted bis(alkyne) **S14** (5.0 mmol, 1.0 equiv) was dissolved in THF (25 mL), and the solution cooled to −78 °C. Then, *n*BuLi (4.8 mL, 2.5 M solution in *n*-hexane, 12.0 mmol, 2.4 equiv) was added dropwise to this solution. After stirring at −78 °C for 30 min, iodomethane (1.78 g, 12.5 mmol, 2.5 equiv) was added slowly, and the resulting mixture was allowed to gradually warm to ambient temperature and stirred for additional 8 h. Upon completion (monitored by TLC), the reaction was quenched by the addition of saturated aqueous NH_4Cl solution (5.0 mL), and the resulting mixture was extracted with CH_2Cl_2 (3 × 20 mL). The combined organic phases were dried over Na_2SO_4 and concentrated under reduced pressure. Purification of the residue by flash column chromatography on silica gel using *n*-pentane as the eluent afforded bis(alkyne) **2p** (663.1 mg, 86% yield) or **2q** (632.3 mg, 82% yield) as a white solid.

4.5 Characterization Data for Internal Alkynes 2

1,3-Dimethyl-5-(prop-1-yn-1-yl)benzene (2k)

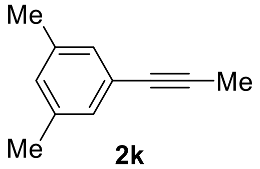

2k
$C_{11}H_{12}$
M = 144.22 g/mol

^{1}H NMR (500 MHz, $CDCl_3$, 298 K): *δ* = 7.04–7.02 (m, 2H), 6.93–6.90 (m, 1H), 2.28 (s, 6H), 2.04 (s, 3H) ppm. **^{13}C{^{1}H} NMR** (101 MHz, $CDCl_3$, 298 K): *δ* = 137.8, 129.6, 129.3, 123.7, 85.1, 80.1, 21.2, 4.4 ppm. The NMR spectroscopic data are in accordance with those reported.[S13]

2-(Prop-1-yn-1-yl)thiophene (2m)

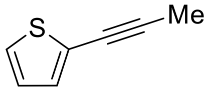

2m
C_7H_6S
M = 122.19 g/mol

^{1}H NMR (500 MHz, $CDCl_3$, 298 K): *δ* = 7.06 (dd, *J* = 5.2, 1.2 Hz, 1H), 7.01 (d, *J* = 3.6 Hz, 1H), 6.83 (dd, *J* = 5.1, 3.6 Hz, 1H), 1.97 (s, 3H) ppm. **^{13}C{^{1}H} NMR** (101 MHz, $CDCl_3$,

298 K): δ = 131.0, 126.9, 126.0, 90.1, 73.0, 4.8 ppm. The NMR spectroscopic data are in accordance with those reported.[S11]

Pent-1-yn-1-ylbenzene (2o)

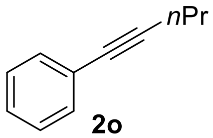

2o
$C_{11}H_{12}$
M = 144.22 g/mol

^{1}H NMR (500 MHz, $CDCl_3$, 298 K): δ = 7.44–7.37 (m, 2H), 7.32–7.25 (m, 3H), 2.40 (t, J = 7.1 Hz, 2H), 1.64 (h, J = 7.2 Hz, 2H), 1.06 (t, J = 7.2 Hz, 3H) ppm. **^{13}C{^{1}H} NMR** (126 MHz, $CDCl_3$, 298 K): δ = 131.7, 128.3, 127.6, 124.3, 90.4, 80.9, 22.4, 21.5, 13.67 ppm. The NMR spectroscopic data are in accordance with those reported.[S14]

1,4-Di(prop-1-yn-1-yl)benzene (2p)

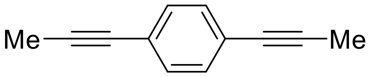

2p
$C_{12}H_{10}$
M = 154.21 g/mol

^{1}H NMR (500 MHz, $CDCl_3$, 298 K): δ = 7.29 (s, 4H), 2.05 (s, 6H) ppm. **^{13}C{^{1}H}** NMR (126 MHz, $CDCl_3$, 298 K): δ = 131.5, 123.3, 87.4, 79.7, 4.5 ppm. The NMR spectroscopic data are in accordance with those reported.[S15]

1,3-Di(prop-1-yn-1-yl)benzene (2q)

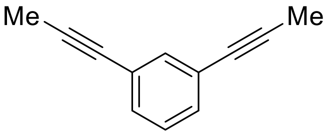

2q
$C_{12}H_{10}$
M = 154.21 g/mol

^{1}H NMR (400 MHz, $CDCl_3$, 298 K): δ = 7.43–7.39 (m, 1H), 7.31–7.25 (m, 2H), 7.23–7.15 (m, 1H), 2.04 (s, 6H) ppm. **^{13}C{^{1}H}** NMR (101 MHz, $CDCl_3$, 298 K): δ = 134.6, 130.7, 128.3, 124.3, 86.4, 79.2, 4.4 ppm. The NMR spectroscopic data are in accordance with those reported.[S16]

5 Experimental Details for the Silylium-Ion-Promoted (3+2) Annulation of Allenylsilanes with Internal Alkynes

5.1 General Procedure for the (3+2) Annulation (GP 3)

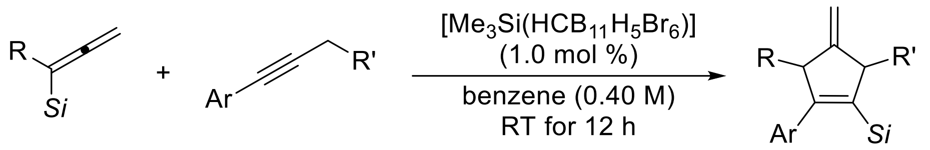

1 **2** **3**

In an argon-filled glovebox, allenylsilane **1** (0.20 mmol, 1.0 equiv) and alkyne **2** (0.25 mmol, 1.25 equiv) are dissolved in benzene (0.5 mL). After stirring for 1 min, silylium carborate [$Me_3Si(HCB_{11}H_5Br_6)$] (1.4 mg, 2.0 *μ*mmol, 1.0 mol %) is added, and the resulting reaction mixture is stirred for additional 12 h at room temperature. Upon completion of the reaction, the reaction mixture is removed from the glovebox, and all volatiles are evaporated under reduced pressure. CH_2Br_2 (34.8 mg, 0.20 mmol, 1.0 equiv) is subsequently added as an internal standard, and C_6D_6 (0.5 mL) is used as the NMR solvent to determine the yield by ^1H NMR spectroscopy. Purification by flash column chromatography on silica gel using *n*-pentane as the eluent affords the (3+2) annulation product **3**.

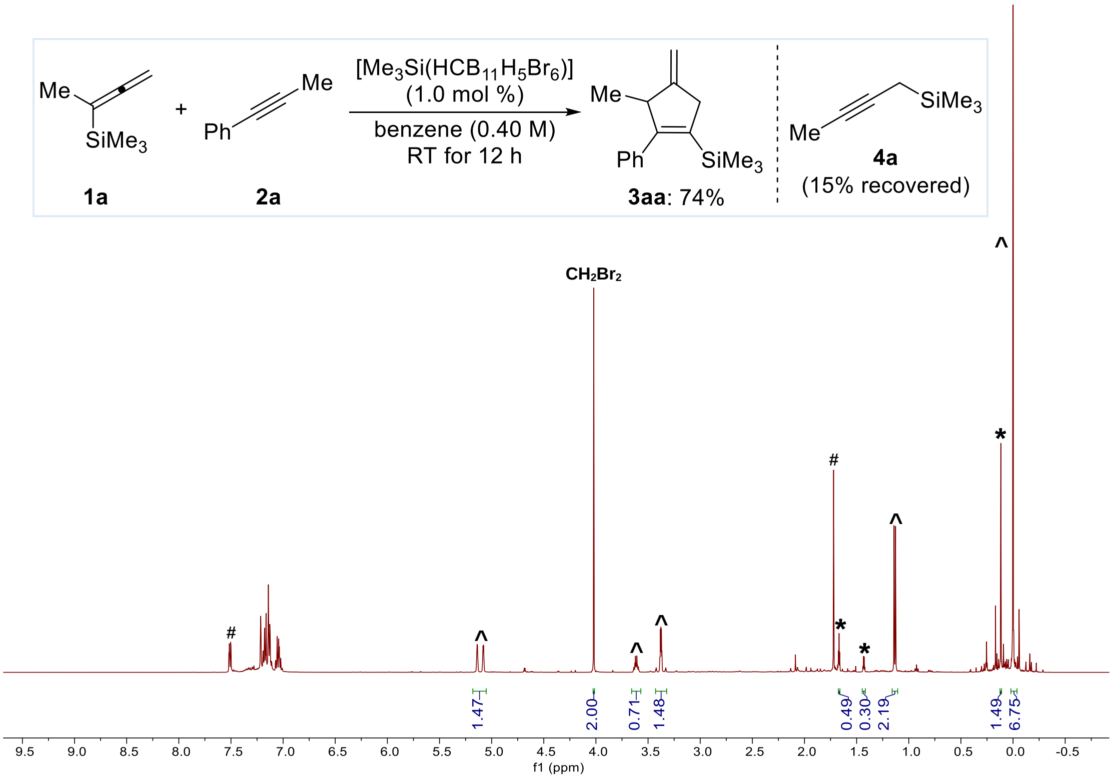

Figure S1. Crude ^1H NMR spectrum of the model reaction ($^{\#}$ for **2a**, ^ for **3aa**, * for **4a**)

5.2 Characterization Data for Annulation Products 3

Trimethyl(3-methyl-4-methylene-2-phenylcyclopent-1-en-1-yl)silane (3aa)

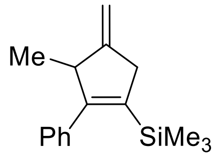

3aa
$C_{16}H_{22}Si$
M = 242.44 g/mol

Prepared from **1a** (25.3 mg, 0.20 mmol) and **2a** (29.0 mg, 0.25 mmol) according to **GP 3**. Flash column chromatography on silica gel using *n*-pentane afforded product **3aa** as a colorless oil (32.9 mg, 68% yield).

R*f* = 0.71 (cyclohexane). **IR** (ATR): ṽ = 3057, 2954, 2663, 2333, 2108, 1878, 1700, 1592, 1442, 1247, 1058, 833, 754, 697 cm^{-1}. **^{1}H NMR** (500 MHz, $CDCl_3$, 298 K): *δ* = 7.46–7.37 (m, 3H), 7.28–7.23 (m, 2H), 5.20–5.03 (m, 2H), 3.73–3.63 (m, 1H), 3.49–3.39 (m, 2H), 1.16 (d, *J* = 7.1 Hz, 3H), 0.02 (s, 9H) ppm. **^{13}C{^{1}H} NMR** (126 MHz, $CDCl_3$, 298 K): *δ* = 157.9, 156.1, 140.2, 136.6, 128.6, 127.9, 127.0, 105.4, 50.7, 44.1, 19.1, −0.5 ppm. **^{29}Si DEPT NMR** (99 MHz, $CDCl_3$, 298 K, optimized for *J* = 7.0 Hz): *δ* = −9.9 ppm. **HRMS** (APCI): calculated for $C_{16}H_{21}Si^+$ $[M-H]^+$: 241.1407; Found 241.1407.

Trimethyl(3-methyl-4-methylene-2-(p-tolyl)cyclopent-1-en-1-yl)silane (3ab)

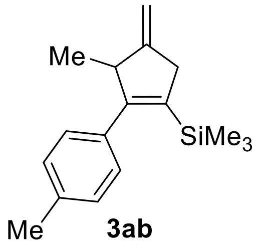

3ab
$C_{17}H_{24}Si$
M = 256.46 g/mol

Prepared from **1a** (25.3 mg, 0.20 mmol) and **2b** (32.5 mg, 0.25 mmol) according to **GP 3**. Flash column chromatography on silica gel using *n*-pentane afforded product **3ab** as a colorless oil (34.9 mg, 68% yield).

R*f* = 0.69 (cyclohexane). **IR** (ATR): ṽ = 3022, 2953, 2476, 2094, 1997, 1905, 1700, 1509, 1246, 1058, 833, 753, 689 cm^{-1}. **^{1}H NMR** (400 MHz, $CDCl_3$, 298 K): *δ* = 7.14–7.08 (m, 2H), 7.05–6.99 (m, 2H), 5.06–4.85 (m, 2H), 3.59–3.48 (m, 1H), 3.36–3.22 (m, 2H), 2.36 (s, 3H), 1.03 (d, *J* = 7.2 Hz, 3H), −0.1 (s, 9H) ppm. **^{13}C{^{1}H} NMR** (101 MHz, $CDCl_3$, 298 K): *δ* = 157.9, 156.3, 137.1, 136.5, 136.1, 128.5, 128.4, 105.3, 50.6, 44.0, 21.4, 19.2, −0.4 ppm. **^{29}Si DEPT NMR** (79 MHz, $CDCl_3$, 298 K, optimized for *J* = 7.0 Hz): *δ* = −9.9 ppm. **HRMS** (APCI): calculated for $C_{17}H_{23}Si^+$ $[M-H]^+$: 255.1564; Found 255.1563.

(2-(4-Fluorophenyl)-3-methyl-4-methylenecyclopent-1-en-1-yl)trimethylsilane (3ac)

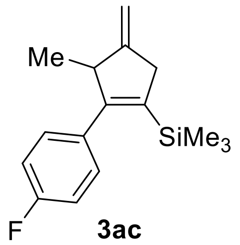

3ac
$C_{16}H_{21}FSi$
M = 260.43 g/mol

Prepared from **1a** (25.3 mg, 0.20 mmol) and **2c** (33.5 mg, 0.25 mmol) according to **GP 3**. Flash column chromatography on silica gel using *n*-pentane afforded product **3ac** as a colorless oil (22.5 mg, 43% yield).

R*f* = 0.75 (cyclohexane). **IR** (ATR): ṽ = 3069, 2955, 2895, 2305, 2094, 1998, 1890, 1701, 1598, 1504, 1221, 1156, 1059, 886, 829, 753, 690 cm^{-1}. **^{1}H NMR** (500 MHz, $CDCl_3$, 298 K): δ = 7.12–7.04 (m, 2H), 7.03–6.96 (m, 2H), 5.03–4.90 (m, 2H), 3.54–3.46 (m, 1H), 3.34–3.24 (m, 2H), 1.01 (d, *J* = 7.1 Hz, 3H), −0.11 (s, 9H) ppm. **^{13}C{^{1}H} NMR** (101 MHz, $CDCl_3$, 298 K): δ = 162.1 (d, $J_{C,F}$ = 245.3 Hz), 156.7, 155.8, 137.3, 136.1 (d, $J_{C,F}$ = 3.2 Hz), 130.1 (d, $J_{C,F}$ = 8.0 Hz), 114.8 (d, $J_{C,F}$ = 21.2 Hz), 105.5, 50.7, 44.0, 19.1, −0.5 ppm. **^{19}F NMR** (471 MHz, $CDCl_3$, 298 K): δ = −115.8 ppm. **^{29}Si DEPT NMR** (79 MHz, $CDCl_3$, 298 K, optimized for *J* = 7.0 Hz): δ = −9.8 ppm. **HRMS** (APCI): calculated for $C_{16}H_{20}FSi^+$ $[M-H]^+$: 259.1313; Found 259.1311.

(2-(4-Chlorophenyl)-3-methyl-4-methylenecyclopent-1-en-1-yl)trimethylsilane (3ad)

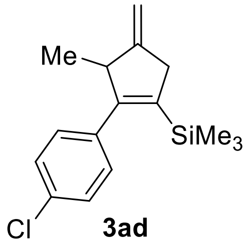

3ad
$C_{16}H_{21}ClSi$
M = 276.88 g/mol

Prepared from **1a** (25.3 mg, 0.20 mmol) and **2d** (37.7 mg, 0.25 mmol) according to **GP 3**. Flash column chromatography on silica gel using *n*-pentane afforded product **3ad** as a colorless oil (42.3 mg, 76% yield).

R*f* = 0.65 (cyclohexane). **IR** (ATR): ṽ = 3068, 2955, 2896, 2568, 2061, 1952, 1897, 1701, 1658, 1485, 1247, 1090, 1014, 886, 828, 753, 690 cm^{-1}. **^{1}H NMR** (400 MHz, $CDCl_3$, 298 K): δ = 7.32–7.23 (m, 2H), 7.10–7.02 (m, 2H), 5.03–4.92 (m, 2H), 3.56–3.47 (m, 1H), 3.36–3.23 (m, 2H), 1.01 (d, *J* = 7.2 Hz, 3H), −0.10 (s, 9H) ppm. **^{13}C{^{1}H} NMR** (101 MHz, $CDCl_3$, 298 K): δ = 156.5, 155.6, 138.6, 137.7, 132.8, 129.9, 128.1, 105.6, 50.6, 44.1, 19.1, −0.5 ppm. **^{29}Si DEPT NMR** (79 MHz, $CDCl_3$, 298 K, optimized for *J* = 7.0 Hz): δ = −9.8 ppm. **HRMS** (APCI): calculated for $C_{16}H_{20}ClSi^+$ $[M-H]^+$: 275.1017; Found 275.1017.

(2-(4-Bromophenyl)-3-methyl-4-methylenecyclopent-1-en-1-yl)trimethylsilane (3ae)

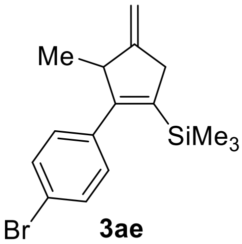

3ae
$C_{16}H_{21}BrSi$
M = 321.33 g/mol

Prepared from **1a** (25.3mg, 0.20 mmol) and **2e** (48.8 mg, 0.25 mmol) according to **GP 3**. Flash column chromatography on silica gel using *n*-pentane afforded product **3ae** as a white solid (38.8 mg, 60% yield). For the molecular structure of **3ae**, see section 9.

R*f* = 0.68 (cyclohexane). **M.P.** = 40–42 °C. **IR** (ATR): ṽ = 3068, 2954, 2895, 2482, 2087, 1933, 1897, 1702, 1657, 1480, 1246, 1069, 1010, 883, 823, 753, 690 cm^{-1}. **^{1}H NMR** (500 MHz, $CDCl_3$, 298 K): δ = 7.45–7.40 (m, 2H), 7.02–6.98 (m, 2H), 5.02–4.92 (m, 2H), 3.55–3.48 (m, 1H), 3.34–3.24 (m, 2H), 1.01 (d, *J* = 7.1 Hz, 3H), −0.1 (s, 9H) ppm. **^{13}C{^{1}H} NMR** (126 MHz, $CDCl_3$, 298 K): δ = 156.5, 155.6, 139.1, 137.7, 131.1, 130.3, 120.9, 105.6, 50.6, 44.2, 19.1, −0.5 ppm. **^{29}Si DEPT NMR** (99 MHz, $CDCl_3$, 298 K, optimized for *J* = 7.0 Hz): δ = −9.8 ppm. **HRMS** (APCI): calculated for $C_{16}H_{20}BrSi^+$ [M−H]$^+$: 319.0512; Found 319.0510.

(2-(2-Fluorophenyl)-3-methyl-4-methylenecyclopent-1-en-1-yl)trimethylsilane (3ag)

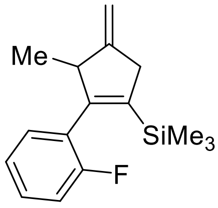

3ag
$C_{16}H_{21}FSi$
M = 260.43 g/mol

Prepared from **1a** (25.3 mg, 0.20 mmol) and **2g** (33.5 mg, 0.25 mmol) according to **GP 3**. Flash column chromatography on silica gel using *n*-pentane afforded product **3ag** as a colorless oil (35.6 mg, 68% yield).

R*f* = 0.71 (cyclohexane). **IR** (ATR): ṽ = 3068, 2956, 2895, 2258, 1704, 1659, 1486, 1447, 1247, 1060, 887, 832, 752, 690 cm^{-1}. **^{1}H NMR** (500 MHz, $CDCl_3$, 298 K): δ = 7.32–7.25 (m, 1H), 7.14–7.10 (m, 2H), 7.08–7.03 (m, 1H), 5.06–4.96 (m, 2H), 3.65–3.58 (m, 1H), 3.41–3.30 (m, 2H), 1.06 (dd, *J* = 7.2, 0.8 Hz, 3H), −0.08 (s, 9H) ppm. **^{13}C{^{1}H} NMR** (126 MHz, $CDCl_3$, 298 K): δ = 160.1 (d, $J_{C,F}$ = 244.8 Hz), 155.7, 151.5, 139.5, 131.5 (d, $J_{C,F}$ = 4.2 Hz), 128.9 (d, $J_{C,F}$ = 8.0 Hz), 127.6 (d, $J_{C,F}$ = 17.5 Hz), 123.5 (d, $J_{C,F}$ = 3.6 Hz), 115.3 (d, $J_{C,F}$ = 22.3 Hz), 105.4, 50.3, 43.9, 18.7, −1.1 ppm. **^{19}F NMR** (471 MHz, $CDCl_3$, 298 K): δ = −114.3 ppm. **^{29}Si DEPT NMR** (99 MHz, $CDCl_3$, 298 K, optimized for *J* =

7.0 Hz): δ = −9.6 ppm. **HRMS** (APCI): calculated for $C_{16}H_{20}FSi^+$ $[M-H]^+$: 259.1313; Found 259.1313.

Trimethyl(3-methyl-4-methylene-2-(m-tolyl)cyclopent-1-en-1-yl)silane (3ah)

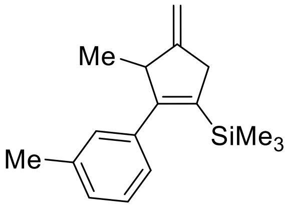

3ah
$C_{17}H_{24}Si$
M = 256.46 g/mol

Prepared from **1a** (25.3 mg, 0.20 mmol) and **2h** (32.5 mg, 0.25 mmol) according to **GP 3**. Flash column chromatography on silica gel using *n*-pentane afforded product **3ah** as a colorless oil (32.4 mg, 63% yield).

R*f* = 0.69 (cyclohexane). **IR** (ATR): ṽ = 3032, 2954, 2895, 2481, 2086, 1868, 1701, 1657, 1246, 1059, 833, 782, 753, 701 cm^{-1}. **^{1}H NMR** (400 MHz, $CDCl_3$, 298 K): δ = 7.22–7.15 (m, 1H), 7.10–7.04 (m, 1H), 6.97–6.90 (m, 2H), 5.03–4.92 (m, 2H), 3.60–3.51 (m, 1H), 3.37–3.24 (m, 2H), 2.35 (s, 3H), 1.04 (d, *J* = 7.1 Hz, 3H), −0.10 (s, 9H) ppm. **^{13}C{^{1}H} NMR** (101 MHz, $CDCl_3$, 298 K): δ = 158.1, 156.2, 140.0, 137.2, 136.2, 129.3, 127.7, 127.7, 125.6, 105.3, 50.6, 44.0, 21.6, 19.2, −0.5 ppm. **^{29}Si DEPT NMR** (79 MHz, $CDCl_3$, 298 K, optimized for *J* = 7.0 Hz): δ = −9.9 ppm. **HRMS** (APCI): calculated for $C_{17}H_{23}Si^+$ $[M-H]^+$: 255.1564; Found 255.1563.

(2-(3-Fluorophenyl)-3-methyl-4-methylenecyclopent-1-en-1-yl)trimethylsilane (3ai)

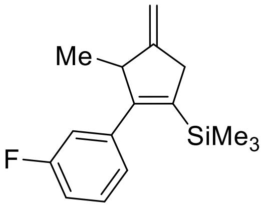

3ai
$C_{16}H_{21}FSi$
M = 260.43 g/mol

Prepared from **1a** (25.3 mg, 0.20 mmol) and **2i** (33.5 mg, 0.25 mmol) according to **GP 3**. Flash column chromatography on silica gel using *n*-pentane afforded product **3ai** as a colorless oil (24.2 mg, 46% yield).

R*f* = 0.77 (cyclohexane). **IR** (ATR): ṽ = 3070, 2955, 2896, 2332, 2092, 1933, 1705, 1578, 1481, 1246, 1152, 1059, 833, 783, 753, 692 cm^{-1}. **^{1}H NMR** (500 MHz, $CDCl_3$, 298 K): δ = 7.31–7.25 (m, 1H), 7.01–6.95 (m, 1H), 6.95–6.90 (m, 1H), 6.89–6.83 (m, 1H), 5.05–4.95 (m, 2H), 3.59–3.52 (m, 1H), 3.38–3.28 (m, 2H), 1.05 (d, *J* = 7.1 Hz, 3H), −0.07 (s, 9H) ppm. **^{13}C{^{1}H} NMR** (126 MHz, $CDCl_3$, 298 K): δ = 162.6 (d, $J_{C,F}$ = 245.9 Hz), 156.5, 155.6,

142.5 (d, $J_{C,F}$ = 7.2 Hz), 137.7, 129.4 (d, $J_{C,F}$ = 8.5 Hz), 124.4 (d, $J_{C,F}$ = 2.9 Hz), 115.5 (d, $J_{C,F}$ = 20.9 Hz), 113.9 (d, $J_{C,F}$ = 21.0 Hz), 105.6, 50.6, 44.1, 19.1, −0.6 ppm. **^{19}F NMR** (471 MHz, $CDCl_3$, 298 K): δ = −114.0 ppm. **^{29}Si DEPT NMR** (79 MHz, $CDCl_3$, 298 K, optimized for J = 7.0 Hz): δ = −9.7 ppm. **HRMS** (APCI): calculated for $C_{16}H_{20}FSi^+$ $[M{-}H]^+$: 259.1313; Found 259.1310.

(2-(3-Chlorophenyl)-3-methyl-4-methylenecyclopent-1-en-1-yl)trimethylsilane (3aj)

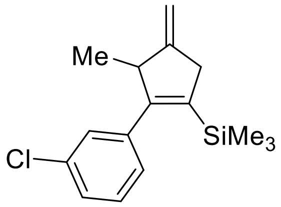

3aj
$C_{16}H_{21}ClSi$
M = 276.88 g/mol

Prepared from **1a** (25.3 mg, 0.20 mmol) and **2j** (37.7 mg, 0.25 mmol) according to **GP 3**. Flash column chromatography on silica gel using *n*-pentane afforded product **3aj** as a colorless oil (31.3 mg, 56% yield).

R*f* = 0.67 (cyclohexane). **IR** (ATR): ṽ = 3065, 2955, 2894, 2480, 2287, 2091, 1936, 1869, 1703, 1585, 1471, 1247, 1076, 880, 831, 782, 752, 691 cm^{-1}. **^{1}H NMR** (500 MHz, $CDCl_3$, 298 K): δ = 7.26–7.21 (m, 2H), 7.15–7.12 (m, 1H), 7.03–6.99 (m, 1H), 5.02–4.94 (m, 2H), 3.57–3.50 (m, 1H), 3.36–3.24 (m, 2H), 1.03 (d, J = 7.1 Hz, 3H), −0.09 (s, 9H) ppm. **^{13}C{^{1}H} NMR** (126 MHz, $CDCl_3$, 298 K): δ = 156.3, 155.5, 142.1, 138.1, 133.8, 129.2, 128.7, 127.1, 126.8, 105.7, 50.6, 44.1, 19.1, −0.5 ppm. **^{29}Si DEPT NMR** (99 MHz, $CDCl_3$, 298 K, optimized for J = 7.0 Hz): δ = −9.7 ppm. **HRMS** (APCI): calculated for $C_{16}H_{20}ClSi^+$ $[M{-}H]^+$: 275.1017; Found 275.1018.

(2-(3,5-Dimethylphenyl)-3-methyl-4-methylenecyclopent-1-en-1-yl)trimethylsilane (3ak)

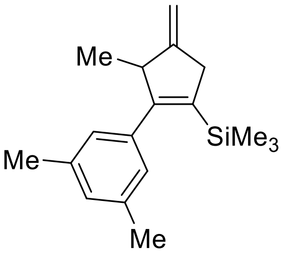

3ak
$C_{18}H_{26}Si$
M = 270.49 g/mol

Prepared from **1a** (25.3 mg, 0.20 mmol) and **2k** (36.1 mg, 0.25 mmol) according to **GP 3**. Flash column chromatography on silica gel using *n*-pentane afforded product **3ak** as a colorless oil (31.1 mg, 57% yield).

R$_f$ = 0.62 (cyclohexane). **IR** (ATR): ṽ = 3383, 2952, 2917, 2330, 2091, 1933, 1700, 1599, 1445, 1376, 1246, 1038, 833, 754, 691 cm^{-1}. **^{1}H NMR** (500 MHz, $CDCl_3$, 298 K): δ = 6.92–6.87 (m, 1H), 6.77–6.73 (m, 2H), 5.04–4.90 (m, 2H), 3.59–3.51 (m, 1H), 3.34–3.24 (m, 2H), 2.31 (s, 6H), 1.04 (d, J = 7.2 Hz, 3H), −0.10 (s, 9H) ppm. **^{13}C{^{1}H} NMR** (101 MHz, $CDCl_3$, 298 K): δ = 158.2, 156.3, 139.9, 137.1, 135.9, 128.5, 126.4, 105.2, 50.4, 44.0, 21.4, 19.3, −0.4 ppm. **^{29}Si DEPT NMR** (79 MHz, $CDCl_3$, 298 K, optimized for J = 7.0 Hz): δ = −10.0 ppm. **HRMS** (APCI): calculated for $C_{18}H_{25}Si^+$ [M−H]$^+$: 269.1720; Found 269.1719.

Trimethyl(3-methyl-4-methylene-2-(naphthalen-2-yl)cyclopent-1-en-1-yl)silane (3al)

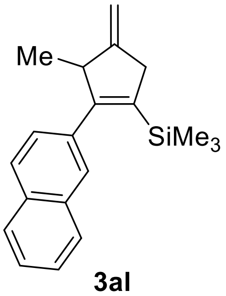

3al
$C_{20}H_{24}Si$
M = 292.50 g/mol

Prepared from **1a** (25.3 mg, 0.20 mmol) and **2l** (41.6 mg, 0.25 mmol) according to **GP 3**. Flash column chromatography on silica gel using *n*-pentane afforded product **3al** as a colorless oil (32.8 mg, 56% yield).

R$_f$ = 0.60 (cyclohexane). **IR** (ATR): ṽ = 3052, 2954, 2893, 2291, 2085, 1941, 1701, 1589, 1245, 1058, 881, 833, 746, 689 cm^{-1}. **^{1}H NMR** (400 MHz, $CDCl_3$, 298 K): δ = 7.84–7.71 (m, 3H), 7.59–7.53 (m, 1H), 7.47–7.38 (m, 2H), 7.27–7.21 (m, 1H), 5.04–4.90 (m, 2H), 3.70–3.59 (m, 1H), 3.39–3.27 (m, 2H), 1.02 (d, J = 7.1 Hz, 3H), −0.14 (s, 9H) ppm. **^{13}C{^{1}H} NMR** (101 MHz, $CDCl_3$, 298 K): δ = 157.8, 156.1, 137.7, 137.2, 133.2, 132.6, 128.0, 127.8, 127.4, 127.3, 126.9, 126.1, 125.7, 105.5, 50.6, 44.2, 19.2, −0.4 ppm. **^{29}Si DEPT NMR** (79 MHz, $CDCl_3$, 298 K, optimized for J = 7.0 Hz): δ = −9.8 ppm. **HRMS** (APCI): calculated for $C_{20}H_{23}Si^+$ [M−H]$^+$: 291.1564; Found 291.1561.

Trimethyl(3-methyl-4-methylene-2-(thiophen-2-yl)cyclopent-1-en-1-yl)silane (3am)

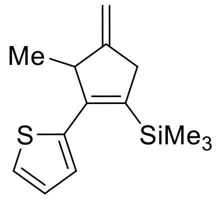

3am
$C_{14}H_{20}SSi$
M = 248.46 g/mol

Prepared from **1a** (25.3 mg, 0.20 mmol) and **2m** (30.5 mg, 0.25 mmol) according to **GP 3**.

Flash column chromatography on silica gel using *n*-pentane afforded product **3am** as a colorless oil (21.5 mg, 40% yield).

R*f* = 0.58 (cyclohexane). **IR** (ATR): ṽ = 3071, 2954, 2895, 2650, 2289, 2093, 1942, 1700, 1596, 1246, 1054, 876, 834, 755, 691 cm^{-1}. **^{1}H NMR** (500 MHz, $CDCl_3$, 298 K): *δ* = 7.26–7.21 (m, 1H), 7.00–6.95 (m, 1H), 6.86–6.81 (m, 1H), 5.01–4.92 (m, 2H), 3.52–3.44 (m, 1H), 3.36–3.25 (m, 2H), 1.13 (d, *J* = 7.1 Hz, 3H), −0.01 (s, 9H) ppm. **^{13}C{^{1}H} NMR** (101 MHz, $CDCl_3$, 298 K): *δ* = 155.4, 150.0, 141.2, 140.6, 126.6, 126.2, 124.7, 105.7, 51.4, 44.1, 19.7, −0.6 ppm. **^{29}Si DEPT NMR** (79 MHz, $CDCl_3$, 298 K, optimized for *J* = 7.0 Hz): *δ* = −9.7 ppm. **HRMS** (APCI): calculated for $C_{14}H_{19}SSi^+$ $[M-H]^+$: 247.0971; Found 247.0971.

Trimethyl(3-methyl-4-methylene-2,5-diphenylcyclopent-1-en-1-yl)silane (3an)

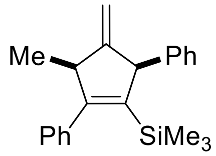

3an
$C_{22}H_{26}Si$
M = 318.54 g/mol

Prepared from **1a** (25.3 mg, 0.20 mmol) and **2n** (48.1 mg, 0.25 mmol) according to **GP 3**. Flash column chromatography on silica gel using *n*-pentane afforded product **3an** as a colorless oil (38.3 mg, 60% yield).

R*f* = 0.52 (cyclohexane). **IR** (ATR): ṽ = 3024, 2958, 2894, 2292, 2100, 1942, 1801, 1652, 1587, 1245, 1066, 890, 833, 751, 697 cm^{-1}. **^{1}H NMR** (500 MHz, $CDCl_3$, 298 K): *δ* = 7.39–7.26 (m, 7H), 7.25–7.19 (m, 3H), 5.03 (t, *J* = 2.3 Hz, 1H), 4.77 (t, *J* = 2.4 Hz, 1H), 4.60 (t, *J* = 2.4 Hz, 1H), 3.79–3.71 (m, 1H), 1.07 (d, *J* = 7.2 Hz, 3H), −0.31 (s, 9H) ppm. **^{13}C{^{1}H} NMR** (101 MHz, $CDCl_3$, 298 K): *δ* = 160.8, 159.7, 146.0, 139.9, 139.7, 128.8, 128.6, 128.4, 128.1, 127.4, 126.4, 108.1, 61.8, 50.2, 20.3, 0.1 ppm. **^{29}Si DEPT NMR** (79 MHz, $CDCl_3$, 298 K, optimized for *J* = 7.0 Hz): *δ* = −9.0 ppm. **HRMS** (APCI): calculated for $C_{22}H_{25}Si^+$ $[M-H]^+$: 317.1720; Found 317.1720.

(5-Ethyl-3-methyl-4-methylene-2-phenylcyclopent-1-en-1-yl)trimethylsilane (3ao)

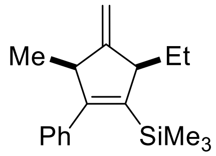

3ao
$C_{18}H_{26}Si$
M = 270.49 g/mol

Prepared from **1a** (25.3 mg, 0.20 mmol) and **2o** (36.1 mg, 0.25 mmol) according to **GP 3**.

Flash column chromatography on silica gel using *n*-pentane afforded product **3ao** as a colorless oil (35.3 mg, 65% yield).

R*f* = 0.69 (cyclohexane). **IR** (ATR): ṽ = 3059, 2960, 2870, 2250, 2090, 1939, 1873, 1653, 1587, 1246, 1025, 831, 754, 698 cm^{-1}. **^{1}H NMR** (500 MHz, $CDCl_3$, 298 K): *δ* = 7.33–7.26 (m, 3H), 7.20–7.14 (m, 2H), 5.06–4.97 (m, 2H), 3.63–3.55 (m, 1H), 3.54–3.48 (m, 1H), 1.78–1.68 (m, 1H), 1.65–1.55 (m, 1H), 1.00 (d, *J* = 7.2 Hz, 3H), 0.88 (t, *J* = 7.4 Hz, 3H), −0.09 (s, 9H) ppm. **^{13}C{^{1}H} NMR** (126 MHz, $CDCl_3$, 298 K): *δ* = 159.0, 158.6, 140.2, 140.1, 128.5, 127.9, 127.2, 105.9, 56.2, 51.1, 28.3, 20.5, 10.0, 0.2 ppm. **^{29}Si DEPT NMR** (99 MHz, $CDCl_3$, 298 K, optimized for *J* = 7.0 Hz): *δ* = −10.0 ppm. **HRMS** (APCI): calculated for $C_{18}H_{25}Si^+$ [M−H]$^+$: 269.1720; Found 269.1721.

(3-Ethyl-4-methylene-2-phenylcyclopent-1-en-1-yl)trimethylsilane (3ba)

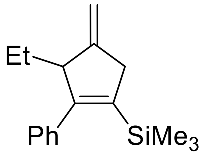

3ba
$C_{17}H_{24}Si$
M = 256.46 g/mol

Prepared from **1b** (28.1 mg, 0.20 mmol) and **2a** (29.0 mg, 0.25 mmol) according to **GP 3**. Flash column chromatography on silica gel using *n*-pentane afforded product **3ba** as a colorless oil (36.0 mg, 70% yield).

R*f* = 0.71 (cyclohexane). **IR** (ATR): ṽ = 3057, 2957, 2233, 2094, 1875, 1701, 1588, 1442, 1246, 1069, 881, 832, 754, 698 cm^{-1}. **^{1}H NMR** (400 MHz, $CDCl_3$, 298 K): *δ* = 7.41–7.32 (m, 3H), 7.29–7.21 (m, 2H), 5.20–4.98 (m, 2H), 3.69–3.59 (m, 1H), 3.40–3.31 (m, 2H), 1.69–1.57 (m, 1H), 1.58–1.43 (m, 1H), 0.84 (t, *J* = 7.2 Hz, 3H), −0.01 (s, 9H) ppm. **^{13}C{^{1}H} NMR** (101 MHz, $CDCl_3$, 298 K): *δ* = 156.0, 153.6, 140.2, 138.1, 128.5, 127.9, 127.0, 106.1, 57.3, 44.8, 25.3, 9.2, −0.4 ppm. **^{29}Si DEPT NMR** (79 MHz, $CDCl_3$, 298 K, optimized for *J* = 7.0 Hz): *δ* = −10.0 ppm. **HRMS** (APCI): calculated for $C_{17}H_{23}Si^+$ [M−H]$^+$: 255.1564; Found 255.1564.

(3-Butyl-4-methylene-2-phenylcyclopent-1-en-1-yl)trimethylsilane (3ca)

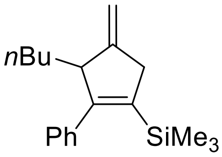

3ca
$C_{19}H_{28}Si$
M = 284.52 g/mol

Prepared from **1c** (33.7 mg, 0.20 mmol) and **2a** (29.0 mg, 0.25 mmol) according to **GP 3**.

Flash column chromatography on silica gel using *n*-pentane afforded product **3ca** as a colorless oil (33.6 mg, 59% yield).

R*f* = 0.66 (cyclohexane). **IR** (ATR): ṽ = 3056, 2955, 2868, 2201, 1942, 1701, 1594, 1443, 1248, 1049, 836, 755, 698 cm^{-1}. **^{1}H NMR** (500 MHz, $CDCl_3$, 298 K): *δ* = 7.41–7.34 (m, 3H), 7.27–7.23 (m, 2H), 5.16–5.01 (m, 2H), 3.66–3.60 (m, 1H), 3.41–3.32 (m, 2H), 1.61–1.54 (m, 1H), 1.49–1.40 (m, 1H), 1.34–1.23 (m, 4H), 0.90 (t, *J* = 6.6 Hz, 3H), 0.00 (s, 9H) ppm. **^{13}C{^{1}H} NMR** (101 MHz, $CDCl_3$, 298 K): *δ* = 156.6, 154.1, 140.2, 137.6, 128.6, 127.8, 127.0, 106.0, 56.4, 44.6, 32.4, 27.3, 23.0, 14.2, −0.4 ppm. **^{29}Si DEPT NMR** (79 MHz, $CDCl_3$, 298 K, optimized for *J* = 7.0 Hz): *δ* = −10.0 ppm. **HRMS** (APCI): calculated for $C_{19}H_{27}Si^+$ [M−H]$^+$: 283.1877; Found 283.1876.

Trimethyl(4-methylene-2,3-diphenylcyclopent-1-en-1-yl)silane (3da)

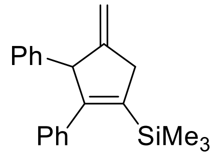

3da
$C_{21}H_{24}Si$
M = 304.51 g/mol

Prepared from **1d** (37.7 mg, 0.20 mmol) and **2a** (29.0 mg, 0.25 mmol) according to **GP 3**. Flash column chromatography on silica gel using *n*-pentane afforded product **3da** as a colorless oil (29.3 mg, 48% yield).

R*f* = 0.48 (cyclohexane). **IR** (ATR): ṽ = 3058, 2951, 2893, 2264, 1943, 1703, 1588, 1442, 1246, 1069, 833, 751, 695 cm^{-1}. **^{1}H NMR** (500 MHz, $CDCl_3$, 298 K): *δ* = 7.23–7.18 (m, 2H), 7.15–7.10 (m, 4H), 7.09–7.05 (m, 2H), 6.98–6.93 (m, 2H), 5.05 (qd, *J* = 2.2, 0.8 Hz, 1H), 4.79 (qd, *J* = 2.3, 0.8 Hz, 1H), 4.65 (t, *J* = 2.3 Hz, 1H), 3.53–3.36 (m, 2H), −0.04 (s, 9H) ppm. **^{13}C{^{1}H} NMR** (126 MHz, $CDCl_3$, 298 K): *δ* = 155.6, 155.1, 143.7, 139.7, 139.4, 128.6, 128.4, 128.3, 127.6, 127.0, 126.2, 108.3, 63.8, 44.6, −0.4 ppm. **^{29}Si DEPT NMR** (79 MHz, $CDCl_3$, 298 K, optimized for *J* = 7.0 Hz): *δ* = −9.5 ppm. **HRMS** (APCI): calculated for $C_{21}H_{23}Si^+$ [M−H]$^+$: 303.1564; Found 303.1557.

Ethyldimethyl(3-methyl-4-methylene-2-phenylcyclopent-1-en-1-yl)silane (3fa)

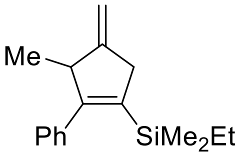

3fa
$C_{17}H_{24}Si$
M = 256.46 g/mol

Prepared from **1f** (28.1 mg, 0.20 mmol) and **2a** (29.0 mg, 0.25 mmol) according to **GP 3**.

Flash column chromatography on silica gel using *n*-pentane afforded product **3fa** as a colorless oil (33.3 mg, 65% yield).

R*f* = 0.75 (cyclohexane). **IR** (ATR): ṽ = 3057, 2952, 2873, 2231, 1942, 1701, 1589, 1490, 1247, 1064, 809, 768, 697 cm^{-1}. **^{1}H NMR** (500 MHz, $CDCl_3$, 298 K): *δ* = 7.32–7.23 (m, 3H), 7.14–7.10 (m, 2H), 5.02–4.92 (m, 2H), 3.58–3.51 (m, 1H), 3.35–3.24 (m, 2H), 1.03 (d, *J* = 7.1 Hz, 3H), 0.83 (t, *J* = 7.9 Hz, 3H), 0.45–0.33 (m, 2H), −0.15 (s, 3H), −0.18 (s, 3H) ppm. **^{13}C{^{1}H} NMR** (126 MHz, $CDCl_3$, 298 K): *δ* = 158.3, 156.2, 140.2, 135.8, 128.6, 127.8, 127.0, 105.3, 50.8, 44.3, 19.2, 7.8, 7.6, −2.8, −3.0 ppm. **^{29}Si DEPT NMR** (99 MHz, $CDCl_3$, 298 K, optimized for *J* = 7.0 Hz): *δ* = −7.0 ppm. **HRMS** (APCI): calculated for $C_{17}H_{23}Si^+$ $[M-H]^+$: 255.1564; Found 255.1562.

Butyldimethyl(3-methyl-4-methylene-2-phenylcyclopent-1-en-1-yl)silane (3ga)

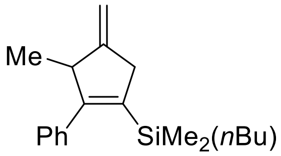

3ga
$C_{19}H_{28}Si$
M = 284.52 g/mol

Prepared from **1g** (33.7 mg, 0.20 mmol) and **2a** (29.0 mg, 0.25 mmol) according to **GP 3**. Flash column chromatography on silica gel using *n*-pentane afforded product **3ga** as a colorless oil (30.7 mg, 54% yield).

R*f* = 0.70 (cyclohexane). **IR** (ATR): ṽ = 3058, 2954, 2920, 2214, 1948, 1701, 1597, 1444, 1248, 1075, 881, 835, 761, 698 cm^{-1}. **^{1}H NMR** (500 MHz, $CDCl_3$, 298 K): *δ* = 7.34–7.23 (m, 3H), 7.15–7.10 (m, 2H), 5.04–4.92 (m, 2H), 3.59–3.51 (m, 1H), 3.35–3.26 (m, 2H), 1.26–1.11 (m, 4H), 1.04 (d, *J* = 7.2 Hz, 3H), 0.83 (t, *J* = 7.1 Hz, 3H), 0.45–0.34 (m, 2H), −0.13 (s, 3H), −0.17 (s, 3H) ppm. **^{13}C{^{1}H} NMR** (101 MHz, $CDCl_3$, 298 K): *δ* = 158.1, 156.2, 140.2, 136.0, 128.6, 127.8, 127.0, 105.3, 50.8, 44.3, 26.7, 26.2, 19.2, 15.8, 13.9, −2.2, −2.4 ppm. **^{29}Si DEPT NMR** (79 MHz, $CDCl_3$, 298 K, optimized for *J* = 21.0 Hz): *δ* = −8.4 ppm. **HRMS** (APCI): calculated for $C_{19}H_{27}Si^+$ $[M-H]^+$: 283.1877; Found 283.1882.

***Iso*-propyldimethyl(3-methyl-4-methylene-2-phenylcyclopent-1-en-1-yl)silane (3ha)**

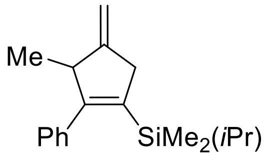

3ha
$C_{18}H_{26}Si$
M = 270.49 g/mol

Prepared from **1h** (30.9 mg, 0.20 mmol) and **2a** (29.0 mg, 0.25 mmol) according to **GP 3**.

Flash column chromatography on silica gel using *n*-pentane afforded product **3ha** as a colorless oil (23.3 mg, 43% yield).

R*f* = 0.64 (cyclohexane). **IR** (ATR): ṽ = 3057, 2949, 2862, 2313, 1946, 1701, 1597, 1491, 1378, 1248, 1067, 999, 880, 762, 698 cm^{-1}. **^{1}H NMR** (500 MHz, $CDCl_3$, 298 K): δ = 7.32–7.24 (m, 3H), 7.14–7.09 (m, 2H), 5.03–4.92 (m, 2H), 3.57–3.49 (m, 1H), 3.35–3.27 (m, 2H), 1.04 (d, *J* = 7.1 Hz, 3H), 0.90 (d, *J* = 7.4 Hz, 3H), 0.86 (d, *J* = 7.4 Hz, 3H), 0.74–0.68 (m, 1H), −0.18 (s, 3H), −0.26 (s, 3H) ppm. **^{13}C{^{1}H} NMR** (101 MHz, $CDCl_3$, 298 K): δ = 158.5, 156.2, 140.3, 135.3, 128.6, 127.8, 126.9, 105.2, 51.0, 44.6, 19.3, 17.8, 13.9, −4.6, −4.8 ppm. **^{29}Si DEPT NMR** (79 MHz, $CDCl_3$, 298 K, optimized for *J* = 27.0 Hz): δ = −4.4 ppm. **HRMS** (APCI): calculated for $C_{18}H_{25}Si^+$ [M−H]$^+$: 269.1720; Found 269.1725.

Triethyl(3-methyl-4-methylene-2-phenylcyclopent-1-en-1-yl)silane (3ia)

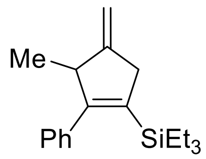

3ia
$C_{19}H_{28}Si$
M = 284.52 g/mol

Prepared from **1i** (33.7 mg, 0.20 mmol) and **2a** (29.0 mg, 0.25 mmol) according to **GP 3**. Flash column chromatography on silica gel using *n*-pentane afforded product **3ia** as a colorless oil (31.3 mg, 55% yield).

R*f* = 0.77 (cyclohexane). **IR** (ATR): ṽ = 3060, 2951, 2908, 2873, 2195, 1944, 1701, 1587, 1488, 1236, 1060, 1002, 878, 718, 698 cm^{-1}. **^{1}H NMR** (500 MHz, $CDCl_3$, 298 K): δ = 7.32–7.24 (m, 3H), 7.15–7.11 (m, 2H), 5.04–4.91 (m, 2H), 3.57–3.49 (m, 1H), 3.35–3.26 (m, 2H), 1.04 (d, *J* = 7.2 Hz, 3H), 0.82 (t, *J* = 7.9 Hz, 9H), 0.44–0.33 (m, 6H) ppm. **^{13}C{^{1}H} NMR** (126 MHz, $CDCl_3$, 298 K): δ = 159.0, 156.3, 140.3, 134.1, 128.5, 127.8, 127.0, 105.1, 51.0, 44.8, 19.4, 7.6, 3.7 ppm. **^{29}Si DEPT NMR** (99 MHz, $CDCl_3$, 298 K, optimized for *J* = 31.0 Hz): δ = −2.1 ppm. **HRMS** (APCI): calculated for $C_{19}H_{27}Si^+$ [M−H]$^+$: 283.1877; Found 283.1880.

1,4-Bis(5-methyl-4-methylene-2-(trimethylsilyl)cyclopent-1-en-1-yl)benzene (3ap)

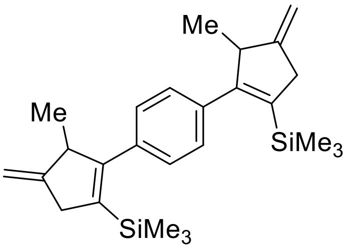

3ap
$C_{26}H_{38}Si_2$
M = 406.76 g/mol

Prepared from **1a** (63.1 mg, 0.50 mmol) and **2p** (30.8 mg, 0.20 mmol) according to **GP 3**. Flash column chromatography on silica gel using *n*-pentane afforded product **3ap** as a pale yellow oil (50.4 mg, 62% yield).

R*f* = 0.72 (cyclohexane). **IR** (ATR): ṽ = 3069, 2955, 2894, 2247, 1934, 1701, 1658, 1592, 1246, 1059, 880, 827, 732, 698 cm^{-1}. **^{1}H NMR** (400 MHz, $CDCl_3$, 298 K): δ = 7.06 (s, 4H), 5.04–4.90 (m, 4H), 3.61–3.51 (m, 2H), 3.38–3.23 (m, 4H), 1.01 (d, *J* = 7.2, 6H), −0.08 (s, 18H) ppm. **^{13}C{^{1}H} NMR** (101 MHz, $CDCl_3$, 298 K): δ = 157.81/157.77, 156.2, 138.74/138.70, 136.4, 128.00/127.97, 105.3, 50.7/50.6, 44.2/44.1, 19.1, −0.36/−0.39 ppm. **^{29}Si DEPT NMR** (79 MHz, $CDCl_3$, 298 K, optimized for *J* = 7.0 Hz): δ = −9.9 ppm. **HRMS** (APCI): calculated for $C_{26}H_{37}Si_2^+$ $[M-H]^+$: 405.2428; Found 405.2428.

1,3-Bis(5-methyl-4-methylene-2-(trimethylsilyl)cyclopent-1-en-1-yl)benzene (3aq)

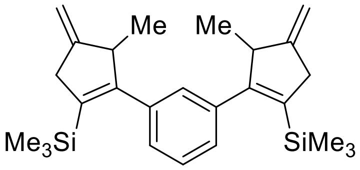

3aq
$C_{26}H_{38}Si_2$
M = 406.76 g/mol

Prepared from **1a** (63.1 mg, 0.50 mmol) and **2q** (30.8 mg, 0.20 mmol) according to **GP 3**. Flash column chromatography on silica gel using *n*-pentane afforded product **3aq** as a pale yellow oil (48.8 mg, 60% yield).

R*f* = 0.67 (cyclohexane). **IR** (ATR): ṽ = 3068, 2954, 2895, 2474, 2251, 2090, 1938, 1874, 1657, 1582, 1246, 1059, 879, 830, 753, 689 cm^{-1}. **^{1}H NMR** (400 MHz, $CDCl_3$, 298 K): δ = 7.26–7.19 (m, 1H), 7.05–6.98 (m, 2H), 6.94–6.86 (m, 1H), 5.03–4.88 (m, 4H), 3.63–3.49 (m, 2H), 3.38–3.25 (m, 4H), 1.01 (d, *J* = 7.1 Hz, 6H), −0.10 (s, 9H), −0.11 (s, 9H) ppm. **^{13}C{^{1}H} NMR** (101 MHz, $CDCl_3$, 298 K): δ = 157.9/157.8, 156.11/156.08, 139.83/139.78, 136.45/136.41, 128.4, 128.1, 127.45, 127.41/127.38, 127.2, 105.4/105.3, 50.8/50.7, 44.3/44.1, 19.2/19.1, −0.3/−0.4 ppm. **^{29}Si DEPT NMR** (79 MHz, $CDCl_3$, 298 K, optimized for *J* = 7.0 Hz): δ = −9.9 ppm. **HRMS** (APCI): calculated for $C_{26}H_{37}Si_2^+$ $[M-H]^+$: 405.2428; Found 405.2423.

6 Mechanistic Control Experiments

Synthesis of Deuterated Alkyne 2a-*d*3

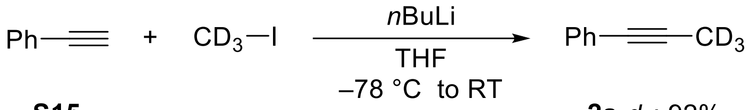

S15 **2a**-*d*3: 92%

According to a reported procedure,[S17] phenylacetylene (**S15**, 5.0 mmol, 1.0 equiv) was dissolved in THF (25 mL), and the solution was cooled to −78 °C. Then, *n*BuLi (2.4 mL, 2.5 M solution in *n*-hexane, 6.0 mmol, 1.2 equiv) was added dropwise to this solution. After stirring for 30 min, iodomethane-*d*3 (906 mg, 6.25 mmol, 1.25 equiv) was added slowly. The resulting mixture was allowed to gradually warm to ambient temperature and stirred for additional 8 h. Upon completion (monitored by TLC), the reaction was quenched by the addition of saturated aqueous NH_4Cl solution (5.0 mL), and the resulting mixture was extracted with CH_2Cl_2 (3 × 20 mL). The combined organic phases were dried over Na_2SO_4 and concentrated under reduced pressure. Purification of the residue by flash column chromatography on silica gel using *n*-pentane as the eluent afforded deuterated alkyne **2a**-*d*3 as a colorless oil (548.2 mg, 92% yield).

(Prop-1-yn-1-yl-d3)benzene (2a-*d*3)

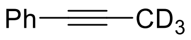

2a-*d*3
$C_9H_5D_3$
M = 119.18 g/mol

R*f* = 0.65 (cyclohexane). **¹H NMR** (500 MHz, $CDCl_3$, 298 K): δ = 7.44–7.37 (m, 2H), 7.33–7.26 (m, 3H) ppm. **¹³C{¹H} NMR** (126 MHz, $CDCl_3$, 298 K): δ = 131.6, 128.3, 127.6, 124.2, 85.9, 79.9, 3.8 (hept, $J_{C,D}$ = 40.1, 20.0 Hz) ppm. **²H NMR** (77 MHz, $CDCl_3$, 298 K): δ = 2.01 (s, 3 × ²H) ppm. The NMR spectroscopic data are in accordance with those reported.[S17]

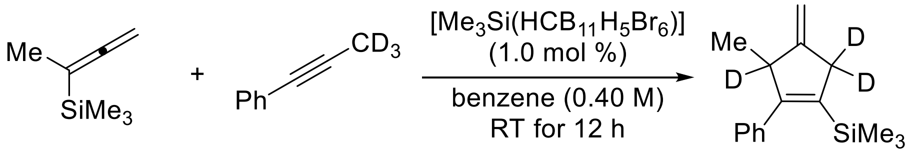

(1)

1a **2a**-*d*3 **3aa**-*d*3: 70% (62%)

In an argon-filled glovebox, allenylsilane **1a** (25.3 mg, 0.20 mmol, 1.0 equiv) and deuterated alkyne **2a**-*d*3 (29.8 mg, 0.25 mmol, 1.25 equiv) were dissolved in benzene (0.5 mL), and the solution was stirred for 1 min. Then, $[Me_3Si(HCB_{11}H_5Br_6)]$ (1.4 mg, 2.0 *μ*mmol, 1.0 mol %) was added, and the resulting mixture was stirred at room temperature for 12 h. Upon completion, the reaction mixture was removed from the

glovebox, and all volatiles were evaporated under reduced pressure. CH_2Br_2 (34.8 mg, 0.20 mmol, 1.0 equiv) was subsequently added as an internal standard, and C_6D_6 (0.5 mL) was used as the NMR solvent for NMR spectroscopic analysis, revealing the formation of **3aa**-*d*3 in 70% NMR yield. Purification by flash column chromatography on silica gel using *n*-pentane as the eluent afforded cycloaddition product **3aa**-*d*3 as a colorless oil (30.4 mg, 62% yield).

Trimethyl(3-methyl-4-methylene-2-phenylcyclopent-1-en-1-yl-3,5,5-d3)silane (3aa-*d*3)

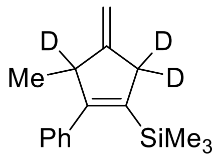

3aa-*d*3
$C_{16}H_{19}D_3Si$
M = 245.46 g/mol

R*f* = 0.73 (cyclohexane). **IR** (ATR): ṽ = 3056, 2954, 2896, 2479, 2091, 1881, 1699, 1492, 1378, 1248, 1049, 834, 751, 697 cm^{-1}. **^{1}H NMR** (500 MHz, $CDCl_3$, 298 K): δ = 7.33–7.25 (m, 3H), 7.15–7.11 (m, 2H), 5.01 (s, 1H), 4.96 (s, 1H) 1.03 (s, 3H), −0.11 (s, 9H) ppm. **^{13}C{^{1}H} NMR** (126 MHz, $CDCl_3$, 298 K): δ = 158.0, 156.0, 140.2, 136.5, 128.6, 127.9, 127.0, 105.4, 50.3 (t, $J_{C,D}$ = 20.1 Hz), 43.4 (m), 19.1, −0.5 ppm. **^{2}H NMR** (77 MHz, $CDCl_3$, 298 K): δ = 3.56 (br, 1 × ^{2}H), 3.30 (br, 2 × ^{2}H) ppm. **^{29}Si DEPT NMR** (99 MHz, $CDCl_3$, 298 K, optimized for J = 7.0 Hz): δ = −9.9 ppm. **HRMS** (APCI): calculated for $C_{16}H_{19}D_2Si^+$ $[M-D]^+$: 243.1533; Found 243.1535.

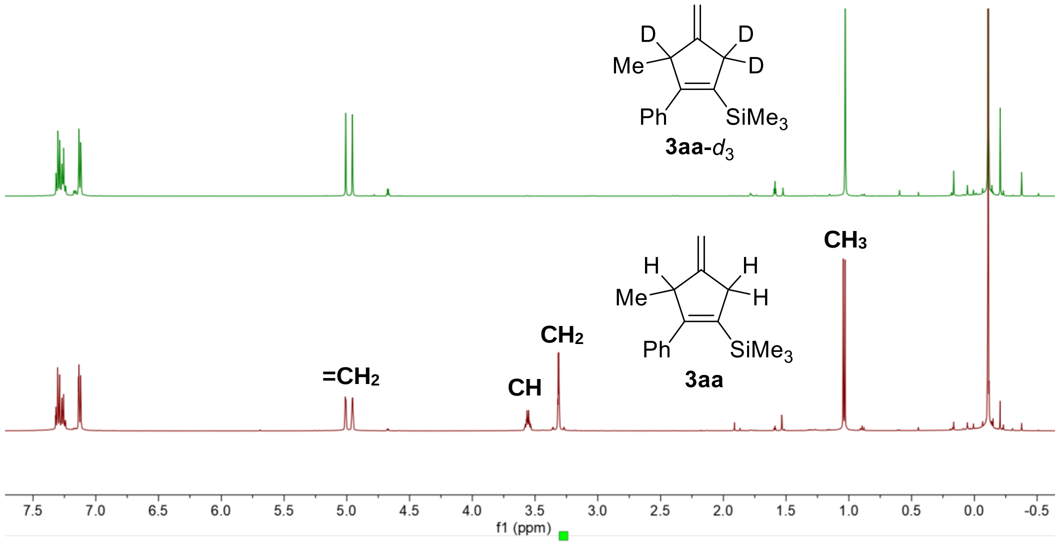

Figure S2. ^{1}H NMR spectra of deuterated product **3aa-*d*3** (top) and non-deuterated product **3aa** (bottom)

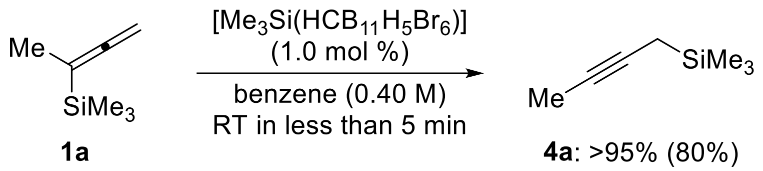

(2)

1a **4a**: >95% (80%)

In an argon-filled glovebox, allenylsilane **1a** (25.3 mg, 0.20 mmol, 1.0 equiv) was dissolved in benzene (0.5 mL), and the solution was stirred for 1 min. Then, $[Me_3Si(HCB_{11}H_5Br_6)]$ (1.4 mg, 2.0 *µ*mmol, 1.0 mol %) was added, and the resulting mixture was stirred at room temperature for 1 min. Next, the reaction mixture was immediately removed from the glovebox and quenched with one drop of Et_3N, and all volatiles were evaporated under reduced pressure (760 mbar, 40 °C). CH_2Br_2 (34.8 mg, 0.20 mmol, 1.0 equiv) was subsequently added as an internal standard, and C_6D_6 (0.5 mL) was used as the NMR solvent for NMR spectroscopic analysis, revealing the formation of **3aa**-*d*$_3$ in >95% NMR yield. Purification by flash column chromatography on silica gel using *n*-pentane as the eluent afforded propargylsilane **4a** as a colorless oil (20.2 mg, 80% yield).

But-2-yn-1-yltrimethylsilane (4a)

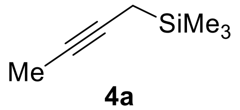

4a
$C_7H_{14}Si$
M = 126.27 g/mol

R*f* = 0.78 (cyclohexane). **^{1}H NMR** (400 MHz, $CDCl_3$, 298 K): δ = 1.77 (t, J = 2.8 Hz, 3H), 1.39 (q, J = 2.8 Hz, 2H), 0.08 (s, 9H) ppm. **^{13}C{^{1}H} NMR** (101 MHz, $CDCl_3$, 298 K): δ = 76.5, 74.0, 7.0, 3.7, −1.9 ppm. **^{29}Si DEPT NMR** (79 MHz, $CDCl_3$, 298 K, optimized for J = 7.0 Hz): δ = 2.6 ppm. The NMR spectroscopic data are in accordance with those reported.[S18]

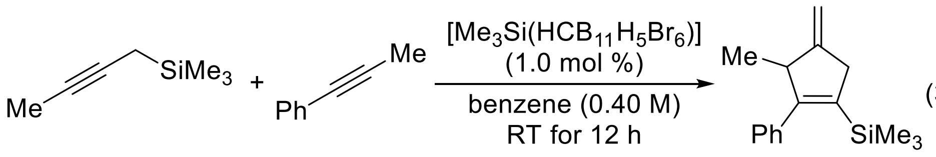

(3)

4a **2a** **3aa**: 60%

In an argon-filled glovebox, propargylsilane **4a** (25.3 mg, 0.20 mmol, 1.0 equiv) and alkyne **2a** (29 mg, 0.25 mmol, 1.25 equiv) were dissolved in benzene (0.5 mL), and the solution was stirred for 1 min. Then, $[Me_3Si(HCB_{11}H_5Br_6)]$ (1.4 mg, 2.0 *µ*mmol, 1.0 mol %) was added, and the resulting mixture was stirred at room temperature for 12 h. Upon completion, the reaction mixture was removed from the glovebox, and all volatiles were evaporated under reduced pressure. CH_2Br_2 (34.8 mg, 0.20 mmol, 1.0 equiv) was

subsequently added as an internal standard, and C_6D_6 (0.5 mL) was used as the NMR solvent for NMR spectroscopic analysis, revealing the formation of **3aa** in 60% NMR yield.

7 Experimental Details for the Synthetic Transformations of 3aa

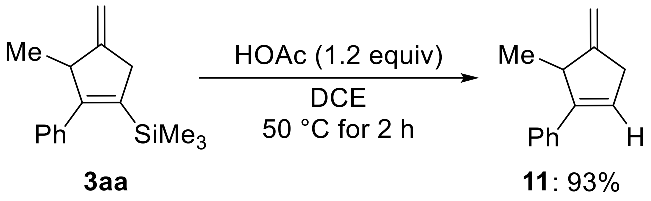

Compound **3aa** (48.5 mg, 0.20 mmol) was weighed into an oven-dried Schlenk tube. The tube was sealed, evacuated and backfilled with N_2 for three times before adding DCE (1.0 mL). Then, HOAc (14.4 mg, 13.7 *µ*L, 1.2 equiv) was added via a syringe in one portion, and the resulting mixture was stirred at 50 °C for 2 h. Upon completion of the reaction (monitored by TLC), all volatiles were evaporated under reduced pressure. Purification of the residue by flash column chromatography on silica gel using *n*-pentane as the eluent afforded product **11** as a colorless oil (31.7 mg, 92% yield).

(5-Methyl-4-methylenecyclopent-1-en-1-yl)benzene (11)

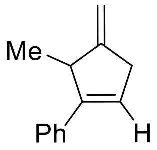

11
$C_{13}H_{14}$
M = 170.26 g/mol

R*f* = 0.73 (cyclohexane). **IR** (ATR): ṽ = 3055, 2963, 2281, 2084, 1944, 1658, 1492, 1446, 1246, 989, 880, 754, 693 cm^{-1}. **^{1}H NMR** (400 MHz, $CDCl_3$, 298 K): *δ* = 7.43–7.38 (m, 2H), 7.37–7.30 (m, 2H), 7.28–7.21 (m, 1H), 6.08–6.00 (m, 1H), 5.09–4.93 (m, 2H), 3.79–3.69 (m, 1H), 3.34–3.13 (m, 2H), 1.21 (d, *J* = 7.1 Hz, 3H) ppm. **^{13}C{^{1}H} NMR** (101 MHz, $CDCl_3$, 298 K): *δ* = 155.3, 147.1, 136.3, 128.5, 127.2, 126.3, 124.2, 106.7, 44.5, 38.7, 20.5 ppm. **HRMS** (APCI): calculated for $C_{13}H_{15}^{+}$ $[M+H]^{+}$: 171.1168; Found 171.1170.

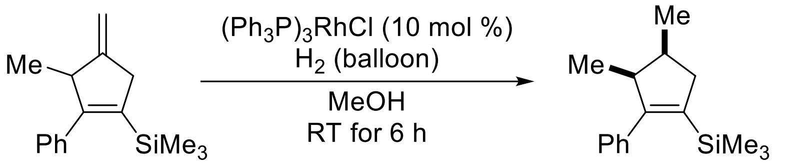

Compound **3aa** (48.5 mg, 0.20 mmol) and $(Ph_3P)_3RhCl$ (18.5 mg, 0.020 mmol, 10 mol %) were weighed into an oven-dried Schlenk tube. The tube was sealed, evacuated and backfilled with H_2 for three times before adding MeOH (1.0 mL). The resulting mixture was stirred at room temperature for 6 h. Upon completion of the reaction (monitored by TLC), all volatiles were evaporated under reduced pressure. Purification of the residue by flash column chromatography on silica gel using *n*-pentane as the eluent afforded product **12** as a colorless oil (43.0 mg, 88% yield, d.r. = 86:14).

(3,4-Dimethyl-2-phenylcyclopent-1-en-1-yl)trimethylsilane (12)

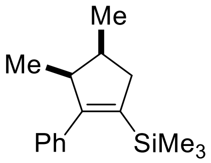

12
$C_{16}H_{24}Si$
M = 244.45 g/mol

R*f* = 0.74 (cyclohexane). **IR** (ATR): ṽ = 3055, 2954, 2830, 2334, 2092, 1803, 1705, 1589, 1442, 1246, 879, 830, 752, 697 cm^{-1}. **^{1}H NMR** (500 MHz, $CDCl_3$, 298 K): δ = 7.32–7.26 (m, 3H), 7.17–7.13 (m, 2H), 2.99–2.86 (m, 1H), 2.69–2.58 (m, 1H), 2.54–2.42 (m, 1H), 2.25–2.08 (m, 1H), 1.02 (d, *J* = 7.0 Hz, 3H), 0.84 (d, *J* = 7.3 Hz, 3H), −0.11 (s, 9H) ppm. **^{13}C{^{1}H} NMR** (101 MHz, $CDCl_3$, 298 K): δ = 159.3, 141.0, 137.2, 128.7, 127.7, 126.6, 49.7, 44.7, 37.1, 15.6, 12.9, −0.5 ppm. **^{29}Si DEPT NMR** (79 MHz, $CDCl_3$, 298 K, optimized for *J* = 7.0 Hz): δ = −10.2 ppm. **HRMS** (APCI): calculated for $C_{16}H_{23}Si^+$ $[M-H]^+$: 243.1564; Found 243.1569.

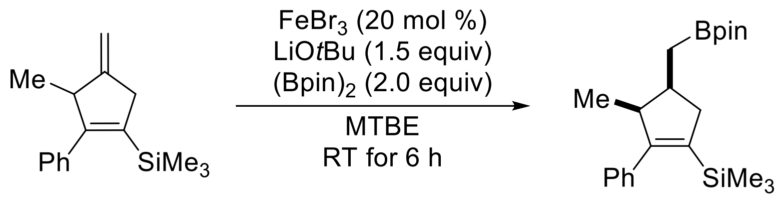

3aa **13**: 81% (d.r. > 95:5)

$FeBr_3$ (11.8 mg, 0.04 mmol, 20 mol %), LiO*t*Bu (24.0 mg, 0.30 mmol, 1.5 equiv) and $(Bpin)_2$ (101.6 mg, 0.40 mmol, 2.0 equiv) were weighed into an oven-dried Schlenk tube. The tube was sealed, evacuated and backfilled with N_2 for three times before adding compound **3aa** (48.5 mg, 0.20 mmol) and MTBE (2.0 mL). The resulting mixture was stirred at room temperature for 6 h. Upon completion (monitored by TLC), the reaction mixture was filtered through a short pad of celite, and the filtrate was evaporated under reduced pressure. Purification of the residue by flash column chromatography on silica gel using *n*-pentane/EtOAc (50/1 to 25/1) as the eluent afforded product **13** as a colorless oil (60.0 mg, 81% yield, d.r. > 95:5).

Trimethyl(3-methyl-2-phenyl-4-((4,4,5,5-tetramethyl-1,3,2-dioxaborolan-2-yl)methyl)cyclopent-1-en-1-yl)silane (13)

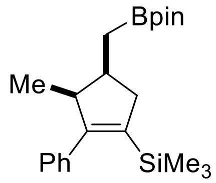

13
$C_{22}H_{35}BO_2Si$
M = 370.42 g/mol

R*f* = 0.52 (cyclohexane/EtOAc = 5/1). **IR** (ATR): ṽ = 3054, 2955, 2829, 2088, 1874, 1589, 1441, 1368, 1313, 1246, 1143, 884, 831, 753, 699 cm^{-1}. **^{1}H NMR** (400 MHz, $CDCl_3$, 298 K): δ = 7.44–7.35 (m, 3H), 7.30–7.24 (m, 2H), 3.06–2.95 (m, 1H), 2.81–2.67 (m, 2H), 2.37–2.24 (m, 1H), 1.41 (s, 12H), 1.09–1.03 (m, 2H), 0.96 (d, *J* = 7.1 Hz, 3H), 0.00 (s, 9H) ppm. **^{13}C{^{1}H} NMR** (101 MHz, $CDCl_3$, 298 K): δ = 159.9, 141.1, 137.2, 128.7, 127.6, 126.6, 83.0, 50.0, 44.6, 39.3, 24.94, 12.9, −0.5 ppm. **^{11}B NMR** (128 MHz, $CDCl_3$, 298 K): δ = 34.2 ppm. **^{29}Si DEPT NMR** (79 MHz, $CDCl_3$, 298 K, optimized for *J* = 7.0 Hz): δ = −10.2 ppm. **HRMS** (ESI): calculated for $C_{22}H_{34}BO_2Si^+$ $[M-H]^+$: 369.2416; Found 369.2423.

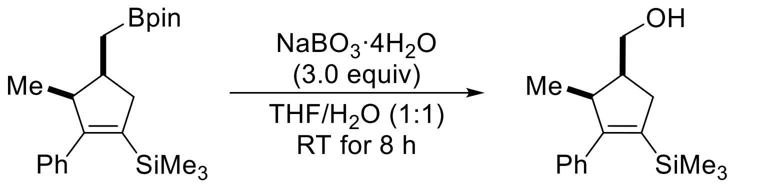

13 **14**: 78% (d.r. > 95:5)

Compound **13** (74.1 mg, 0.20 mmol) was weighed into an Schlenk tube and dissolved in THF (0.5 mL) and H_2O (0.5 mL). Then, $NaBO_3$•$4H_2O$ (92.3 mg, 0.60 mmol, 3.0 equiv) was added in one portion, and the resulting mixture was stirred at room temperature for 8 h. Upon completion of the reaction (monitored by TLC), CH_2Cl_2 (1.0 mL) was added and the organic layer was separated. The aqueous phase was extracted with CH_2Cl_2 (3 × 5 mL). The combined organic phases were dried over Na_2SO_4, and all volatiles were evaporated under reduced pressure. Purification of the residue by flash column chromatography on silica gel using *n*-pentane/EtOAc (30/1 to 15/1) as eluent afforded product **14** as a light yellow oil (40.6 mg, 78% yield, d.r. > 95:5).

(2-Methyl-3-phenyl-4-(trimethylsilyl)cyclopent-3-en-1-yl)methanol (14)

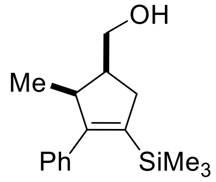

14
$C_{16}H_{24}OSi$
M = 260.45 g/mol

R*f* = 0.42 (cyclohexane/EtOAc = 3/1). **IR** (ATR): ṽ = 3349, 3052, 2957, 2924, 2293, 2092, 1872, 1492, 1246, 1065, 999, 833, 752, 691 cm^{-1}. **^{1}H NMR** (500 MHz, $CDCl_3$, 298 K): δ = 7.46–7.36 (m, 3H), 7.29–7.24 (m, 2H), 3.99–3.92 (m, 1H), 3.87–3.80 (m, 1H), 3.25–3.16 (m, 1H), 2.81–2.70 (m, 2H), 2.49–2.40 (m, 1H), 1.62 (br, 1H), 1.03 (d, *J* = 7.1 Hz, 3H), 0.02 (s, 9H) ppm. **^{13}C{^{1}H} NMR** (101 MHz, $CDCl_3$, 298 K): δ = 159.4, 140.2, 136.5, 128.7, 127.8, 126.9, 64.0, 47.9, 44.9, 39.5, 12.9, −0.5 ppm. **^{29}Si DEPT NMR** (79 MHz, $CDCl_3$, 298 K, optimized for *J* = 7.0 Hz): δ = −9.9 ppm. **HRMS** (APCI): calculated for $C_{16}H_{23}OSi^+$ $[M-H]^+$: 259.1513; Found 259.1517.

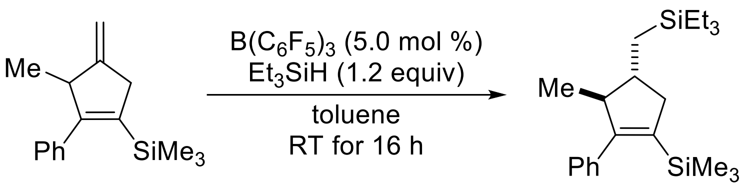

3aa **15**: 89% (d.r. > 95:5)

In an argon-filled glovebox, compound **3aa** (48.5 mg, 0.20 mmol) was weighed into a GC vial and dissolved in toluene (0.5 mL). Then, Et_3SiH (27.9 mg, 0.24 mmol, 1.2 equiv) was added, and the resulting mixture was stirred at room temperature for 1 min. $B(C_6F_5)_3$ (5.1 mg, 0.010 mmol, 5.0 mol %) was added, and the reaction mixture was stirred at room temperature for additional 16 h. After quenching the reaction by the addition of Et_2O (one drop), all volatiles were evaporated under vacuum. Purification of the residue by flash column chromatography on silica gel using *n*-pentane as the eluent afforded product **15** as a colorless oil (63.9 mg, 89% yield, d.r. > 95:5).

Triethyl((2-methyl-3-phenyl-4-(trimethylsilyl)cyclopent-3-en-1-yl)methyl)silane (15)

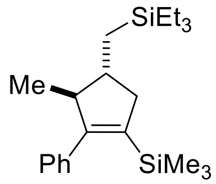

15
$C_{22}H_{38}Si_2$
M = 358.72 g/mol

R*f* = 0.76 (cyclohexane). **IR** (ATR): ṽ = 3023, 2951, 2874, 2089, 1872, 1489, 1372, 1245, 1012, 881, 831, 753, 697 cm^{-1}. **^{1}H NMR** (400 MHz, $CDCl_3$, 298 K): δ = 7.34–7.23 (m, 3H), 7.20–7.07 (m, 2H), 2.79–2.70 (m, 1H), 2.68–2.57 (m, 1H), 2.18–2.06 (m, 1H), 1.92–1.80 (m, 1H), 1.01 (*approx.* t, *J* = 7.9 Hz, 10H), 0.93 (d, *J* = 6.9 Hz, 3H), 0.76–0.66 (m, 1H), 0.61 (q, *J* = 7.9 Hz, 6H), −0.10 (s, 9H) ppm. **^{13}C{^{1}H} NMR** (101 MHz, $CDCl_3$, 298 K): δ = 158.5, 140.9, 137.4, 128.4, 127.7, 126.6, 56.3, 45.8, 44.0, 18.1, 17.1, 7.7, 4.1, −0.5 ppm. **^{29}Si DEPT NMR** (79 MHz, $CDCl_3$, 298 K, optimized for *J* = 35.0 Hz): δ = 6.4, −10.4 ppm. **HRMS** (APCI): calculated for $C_{22}H_{37}Si_2^+$ [M−H]$^+$: 357.2428; Found 357.2435.

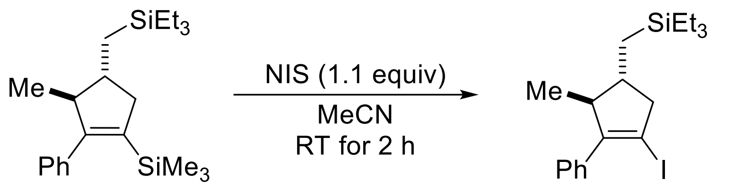

15 **16**: 90% (d.r. > 95:5)

NIS (49.5 mg, 0.22 mmol, 1.1 equiv) was weighed into a Schlenk tube. The tube was sealed, evacuated and backfilled with N_2 for three times before adding compound **15** (71.7 mg, 0.20 mmol) and MeCN (1.0 mL). The reaction mixture was stirred at room temperature for 2 h, and then quenched by the addition of saturated $Na_2S_2O_3$ solution (0.5 mL). The organic layer was separated, and the aqueous phase was extracted with CH_2Cl_2 (3 × 5 mL). The combined organic phases were dried over Na_2SO_4, and all volatiles were evaporated under reduced pressure. Purification of the residue by flash column chromatography on silica gel using *n*-pentane as the eluent afforded product **16** as a pale yellow oil (74.3 mg, 90% yield, d.r. > 95:5).

Triethyl((4-iodo-2-methyl-3-phenylcyclopent-3-en-1-yl)methyl)silane (16)

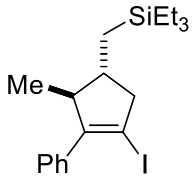

16
$C_{19}H_{29}ISi$
M = 412.43 g/mol

R*f* = 0.68 (cyclohexane). **IR** (ATR): ṽ = 3024, 2950, 2871, 2283, 1942, 1799, 1596, 1490, 1237, 1010, 754, 722, 694 cm^{-1}. **^{1}H NMR** (500 MHz, $CDCl_3$, 298 K): δ = 7.39–7.34 (m, 2H), 7.31–7.25 (m, 3H), 3.03–2.94 (m, 1H), 2.68–2.61 (m, 1H), 2.52–2.42 (m, 1H), 2.05–1.96 (m, 1H), 1.00–0.92 (m, 13H), 0.77–0.68 (m, 1H), 0.57 (q, J = 7.9 Hz, 6H). **^{13}C{^{1}H} NMR** (101 MHz, $CDCl_3$, 298 K): δ = 151.9, 138.0, 128.2, 128.1, 127.5, 91.6, 53.1, 52.7, 44.0, 18.6, 17.4, 7.6, 4.0. **^{29}Si DEPT NMR** (79 MHz, $CDCl_3$, 298 K, optimized for J = 35.0 Hz): δ = 6.2 ppm. **HRMS** (APCI): calculated for $C_{19}H_{28}ISi^+$ $[M{-}H]^+$: 411.0999; Found 411.1001.

8 Determination of the Relative Configuration

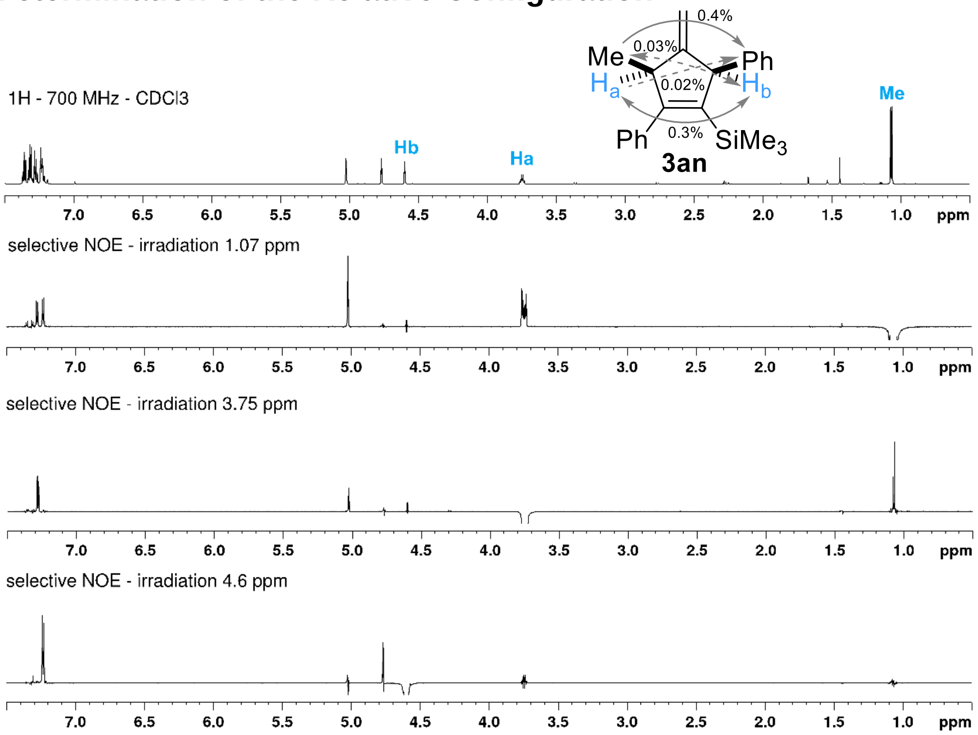

Figure S3. Enhancement factors of selected NOEs taken from selective NOE measurements

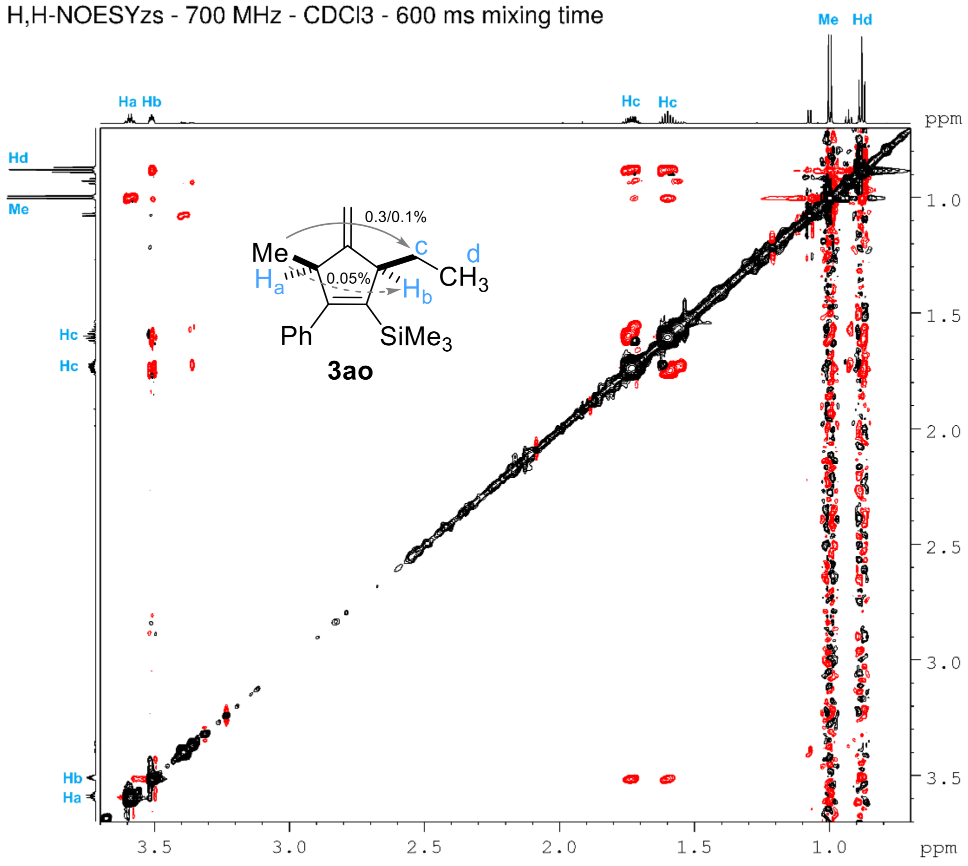

Figure S4. Enhancement factors of selected NOEs taken from a NOESY measurement

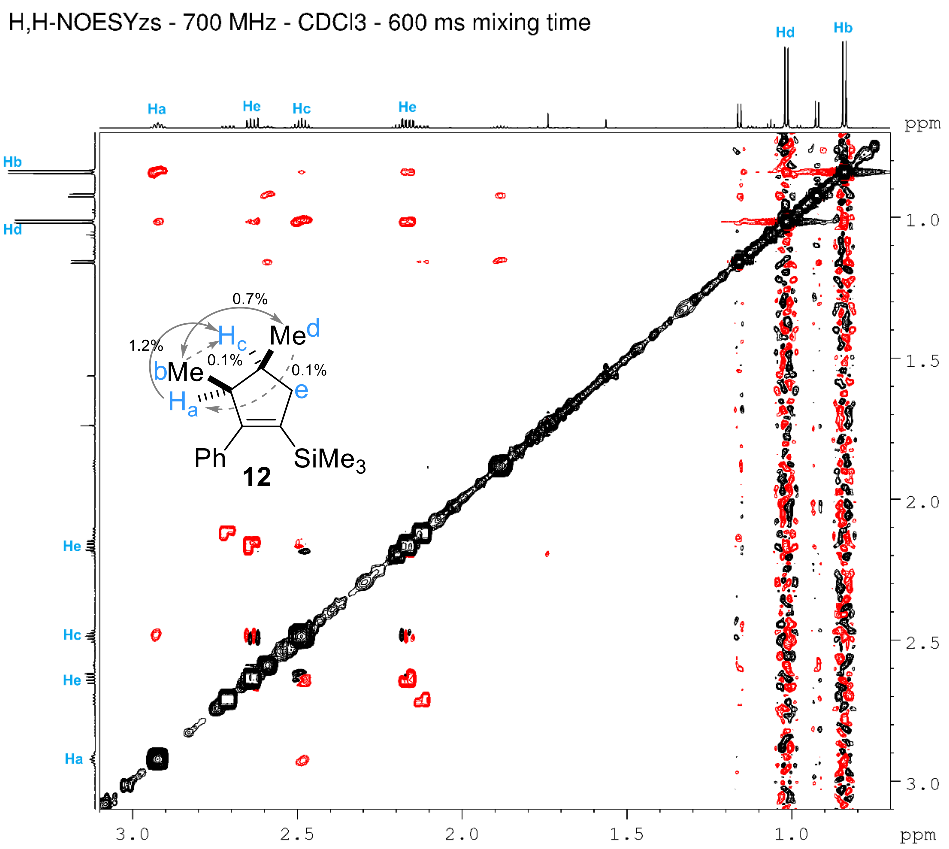

Figure S5. Enhancement factors of selected NOEs taken from a NOESY measurement

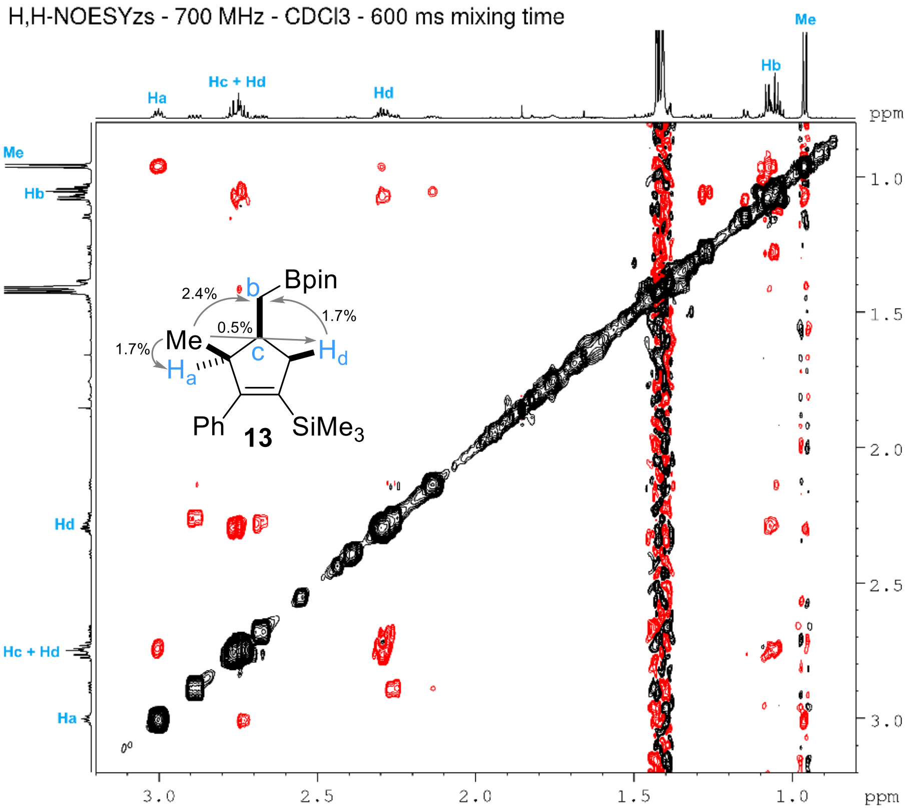

Figure S6. Enhancement factors of selected NOEs taken from a NOESY measurement

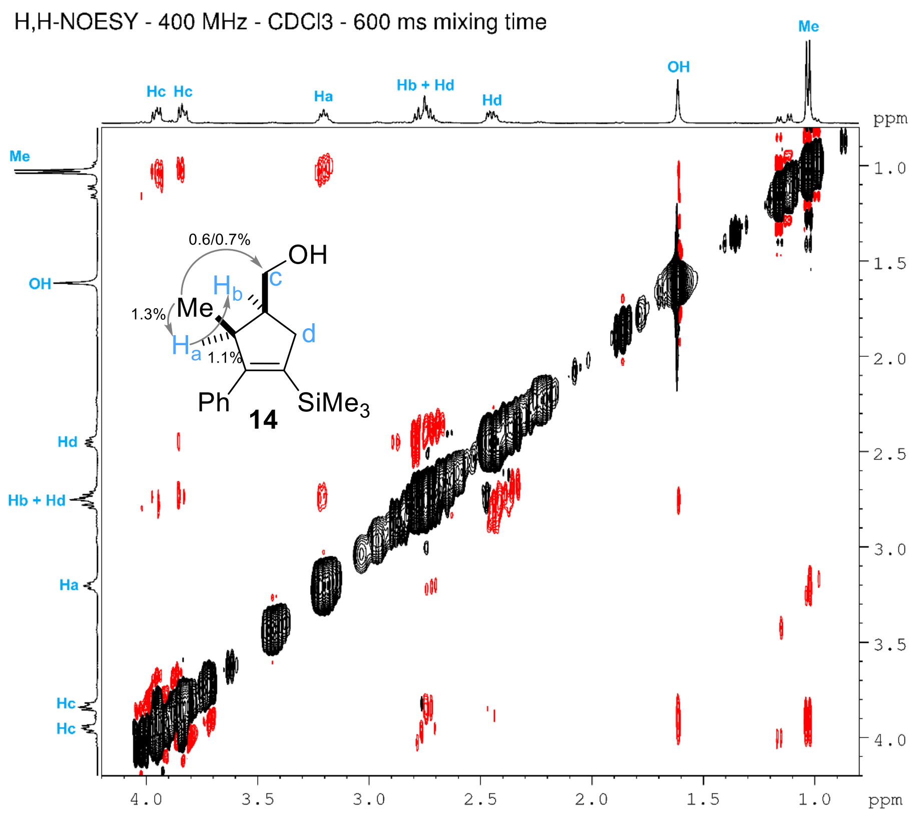

Figure S7. Enhancement factors of selected NOEs taken from a NOESY measurement

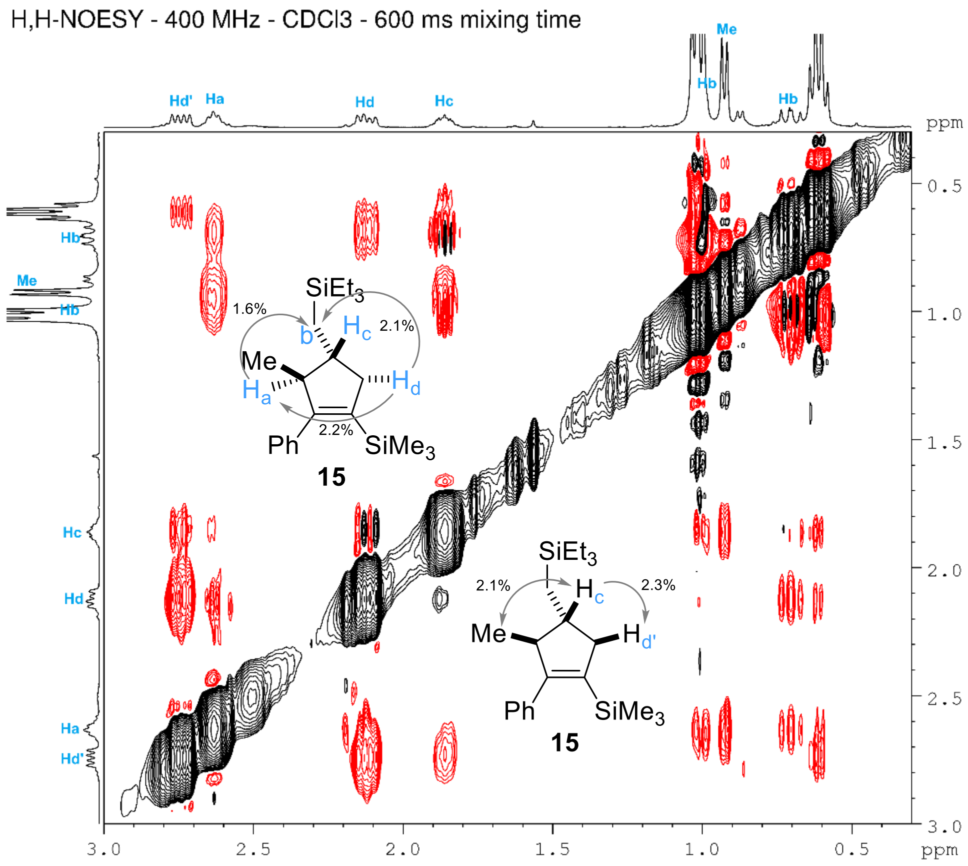

Figure S8. Enhancement factors of selected NOEs taken from a NOESY measurement

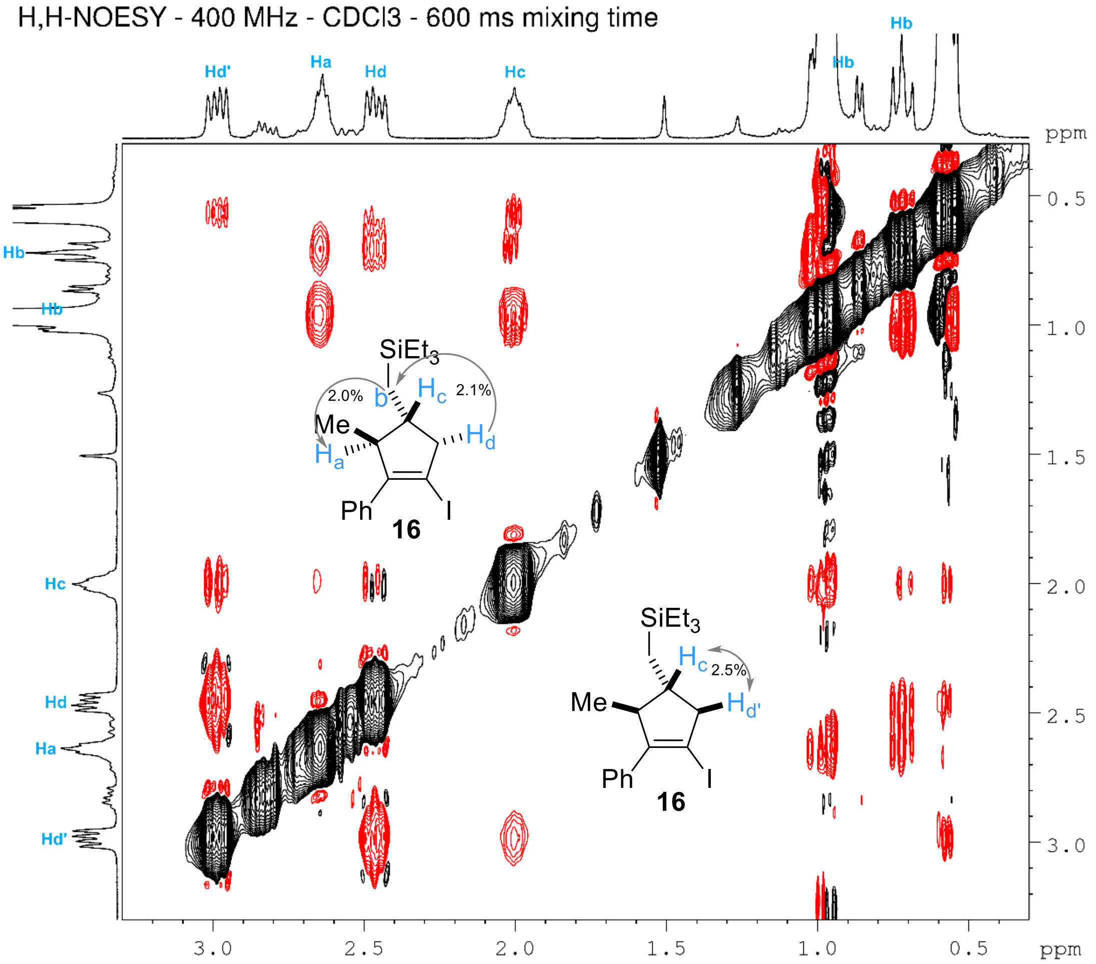

Figure S9. Enhancement factors of selected NOEs taken from a NOESY measurement

9 Crystallographic Data

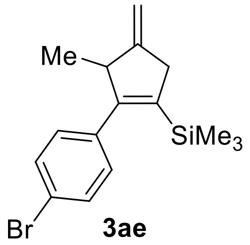

3ae
$C_{16}H_{21}BrSi$
M = 321.33 g/mol

Single crystals of **3ae** suitable for X-ray diffraction analysis were obtained by slow evaporation of a solution of **3ae** in $CHCl_3$ and *n*-hexane at room temperature under argon atmosphere (*Note: slow oxidation of* ***3ae*** *under air was observed*).
CCDC 2339485 contains the supplementary crystallographic data for this compound.

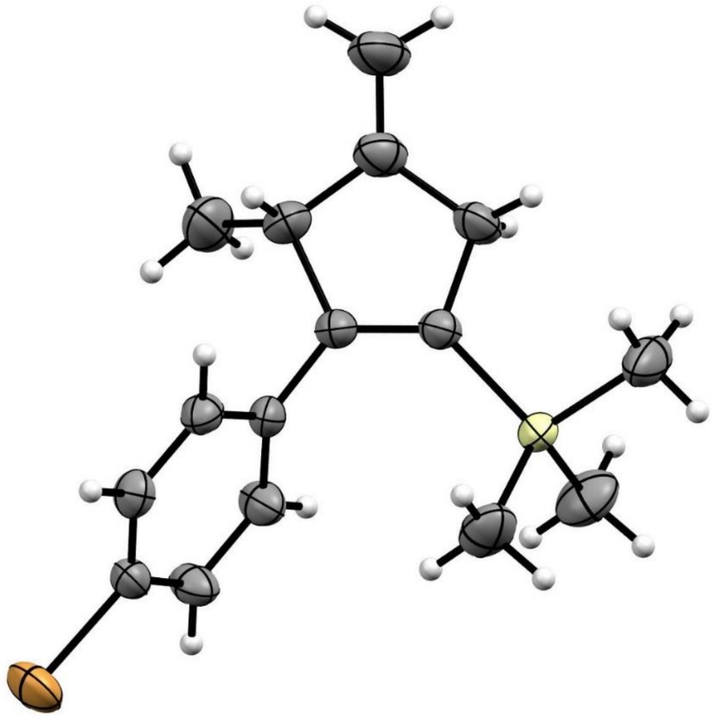

Figure S10. Molecular structure of compound **3ae**
(thermal ellipsoids are shown at the 50% probability level)

Table S1. Crystal data and structure refinement for compound **3ae**

Empirical formula	$C_{16}H_{21}BrSi$	
Formula weight	321.33	
Temperature	150.01(10) K	
Wavelength	1.54184 Å	
Crystal system	Monoclinic	
Space group	$P2_1/n$ (no. 14)	
Unit cell dimensions	a = 8.8849(3) Å	α = 90°
	b = 18.7550(7) Å	β = 100.731(3)°
	c = 9.9671(4) Å	γ = 90°
Volume	1631.84(11) Å^3	
Z	4	
Density (calculated)	1.308 Mg/m^3	
Absorption coefficient	3.976 mm^{-1}	
F(000)	664	
Crystal size	0.268 x 0.218 x 0.064 mm^3	
Theta range for data collection	4.716 to 72.454°	
Index ranges	–10<=h<=10, –22<=k<=22, –8<=l<=12	
Reflections collected	6456	
Independent reflections	3129 [R(int) = 0.0253]	
Completeness to theta = 67.684°	99.9 %	
Absorption correction	Semi-empirical from equivalents	
Max. and min. transmission	1.00000 and 0.01515	
Refinement method	Full-matrix least-squares on F^2	
Data / restraints / parameters	3129 / 0 / 167	
Goodness-of-fit on F^2	1.060	
Final R indices [I>2sigma(I)]	R1 = 0.0407, wR2 = 0.1114	
R indices (all data)	R1 = 0.0466, wR2 = 0.1167	
Extinction coefficient	n/a	
Largest diff. peak and hole	0.580 and –0.660 e.Å^{-3}	

10 NMR Spectra

Figure S11. ^{1}H NMR spectrum (500 MHz, $CDCl_3$, 298 K) of trimethyl(penta-1,2-dien-3-yl)silane (**1b**)

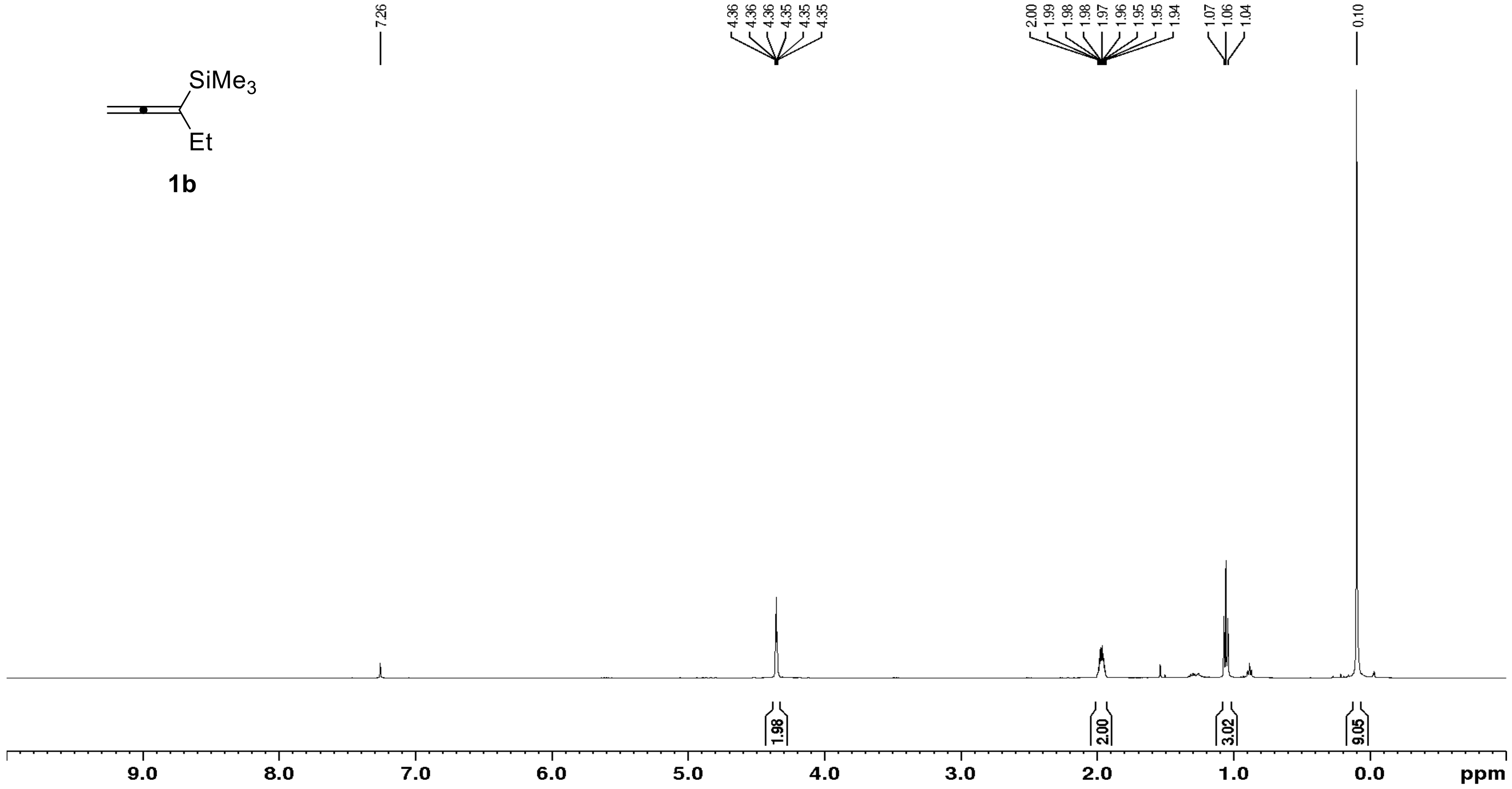

Figure S12. $^{13}C\{^{1}H\}$ NMR spectrum (101 MHz, $CDCl_3$, 298 K) of trimethyl(penta-1,2-dien-3-yl)silane (**1b**)

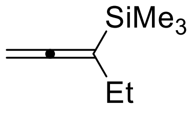

1b

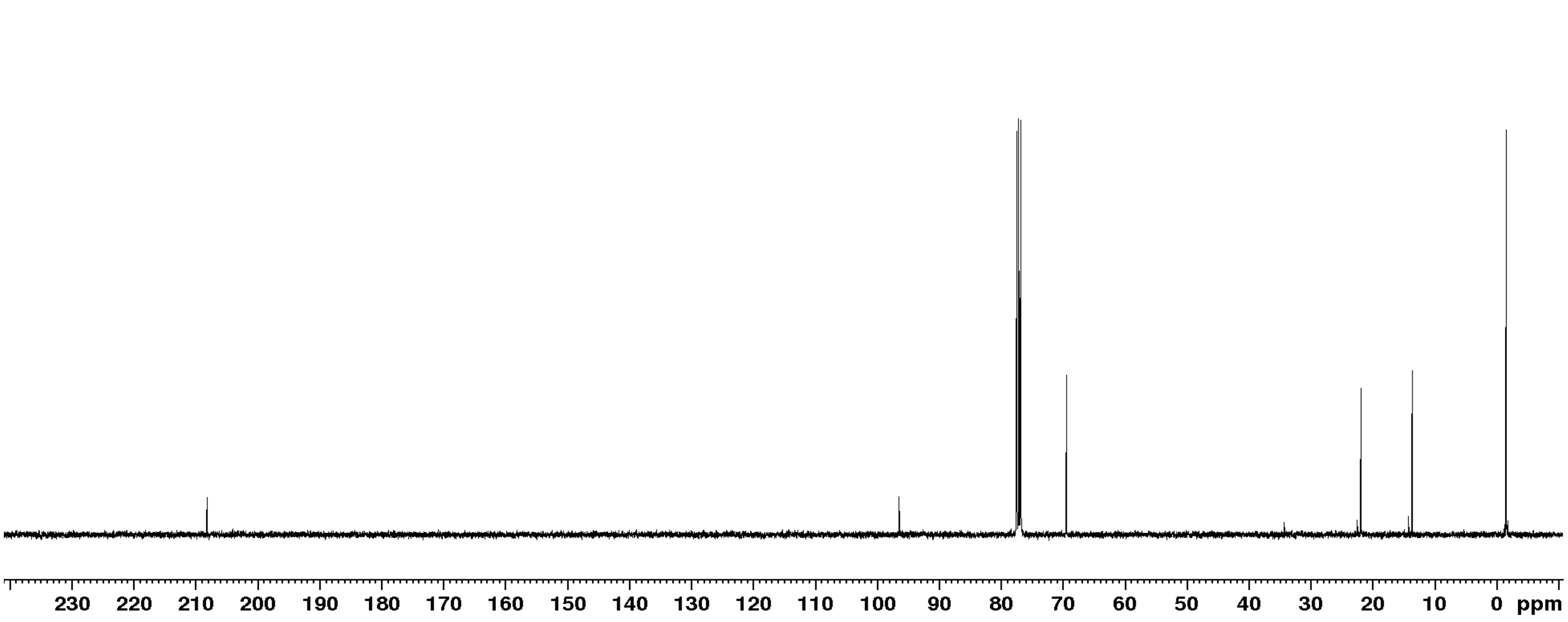

Figure S13. ^{29}Si DEPT NMR spectrum (79 MHz, $CDCl_3$, 298 K, optimized for J = 7.0 Hz) of trimethyl(penta-1,2-dien-3-yl)silane (**1b**)

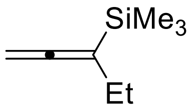

1b

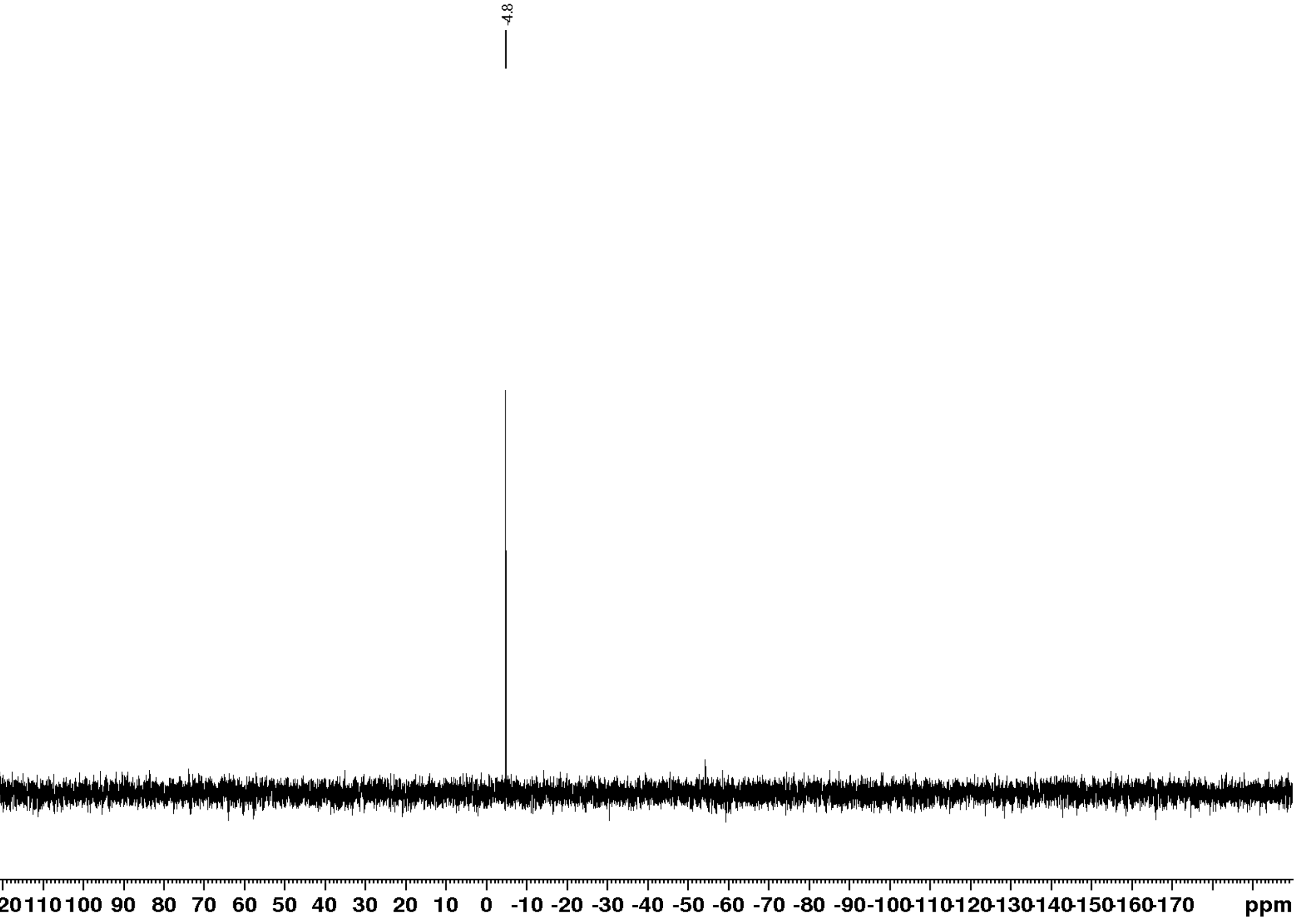

Figure S14. ^{1}H NMR spectrum (500 MHz, $CDCl_3$, 298 K) of buta-2,3-dien-2-yl(ethyl)dimethylsilane (**1f**)

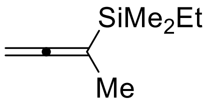

1f

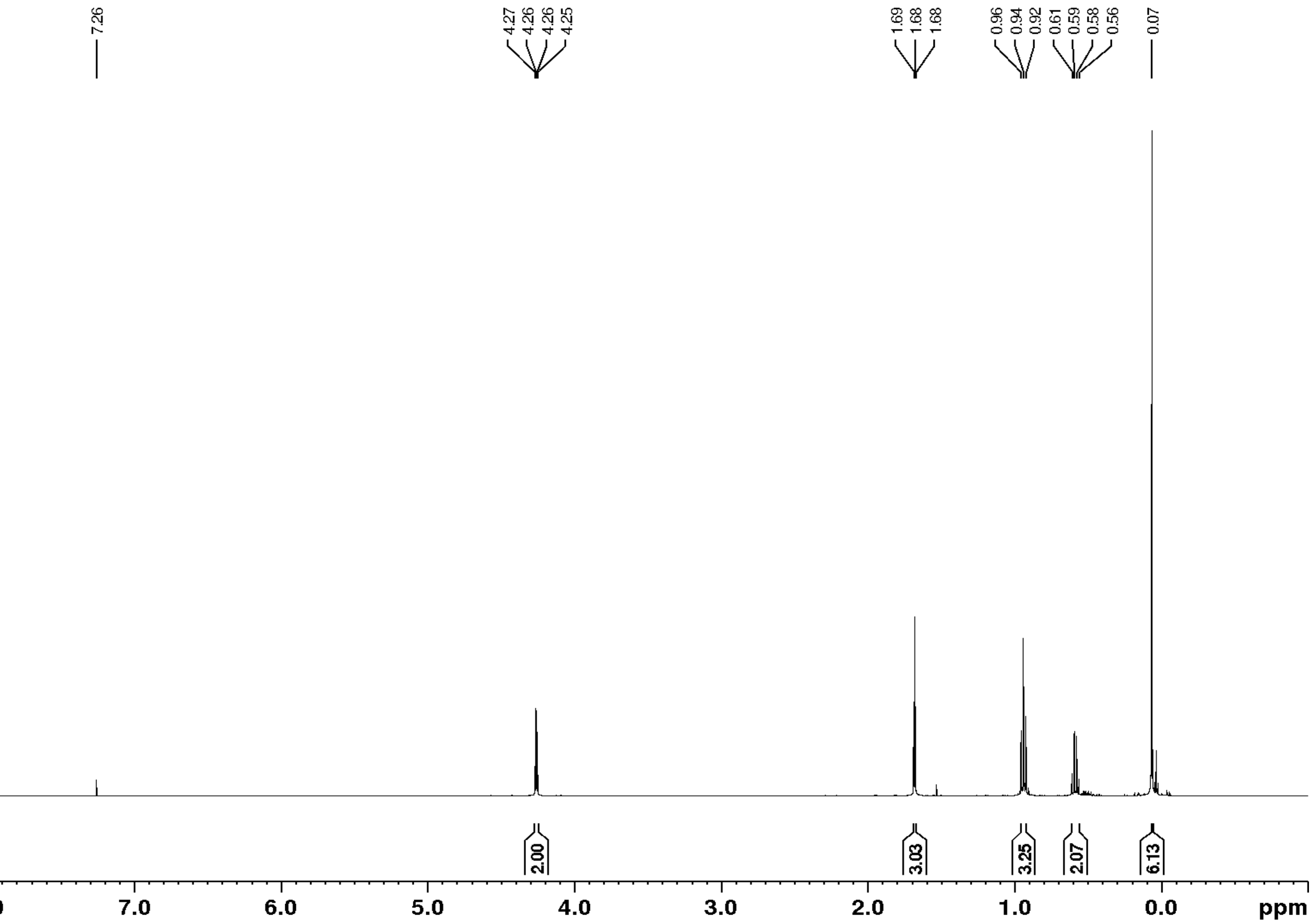

Figure S15. $^{13}C\{^{1}H\}$ NMR spectrum (126 MHz, $CDCl_3$, 298 K) of buta-2,3-dien-2-yl(ethyl)dimethylsilane (**1f**)

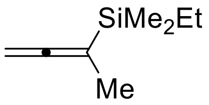

1f

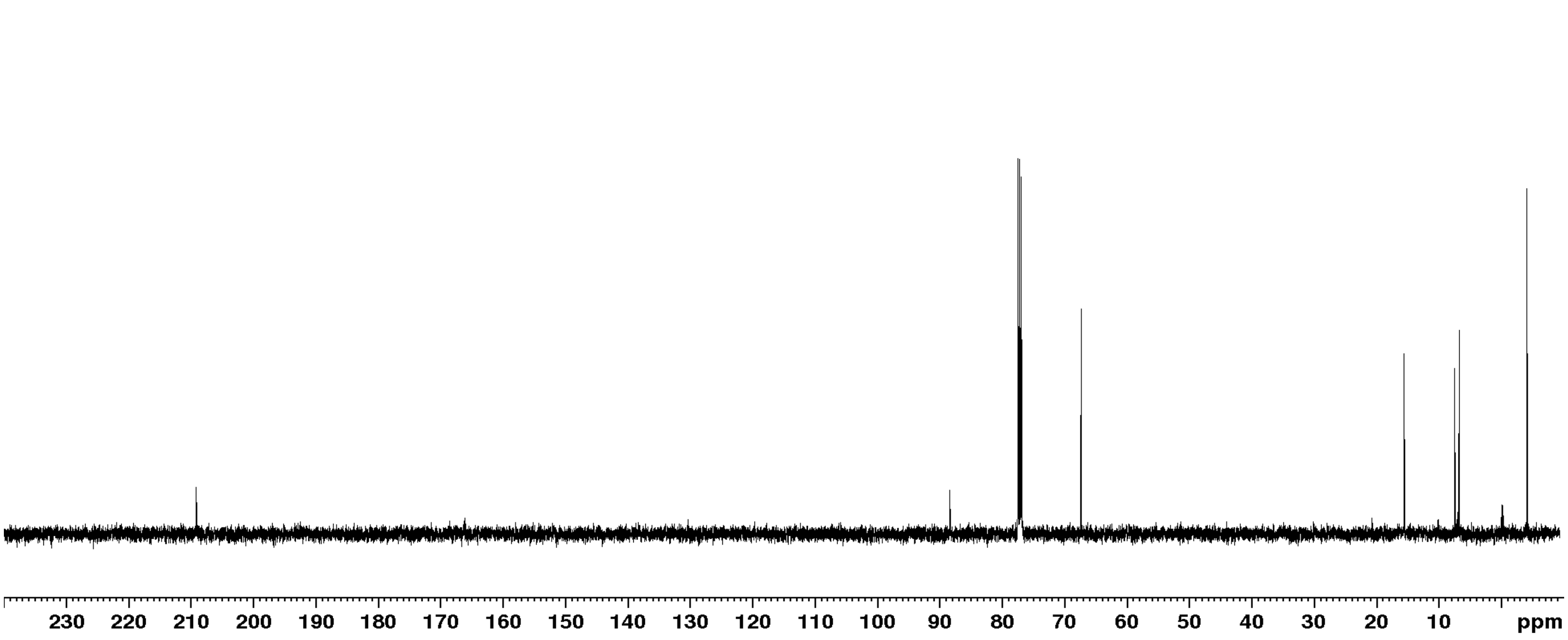

Figure S16. ^{29}Si DEPT NMR spectrum (79 MHz, $CDCl_3$, 298 K, optimized for *J* = 17.0 Hz) of buta-2,3-dien-2-yl(ethyl)dimethylsilane (**1f**)

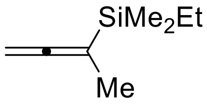

1f

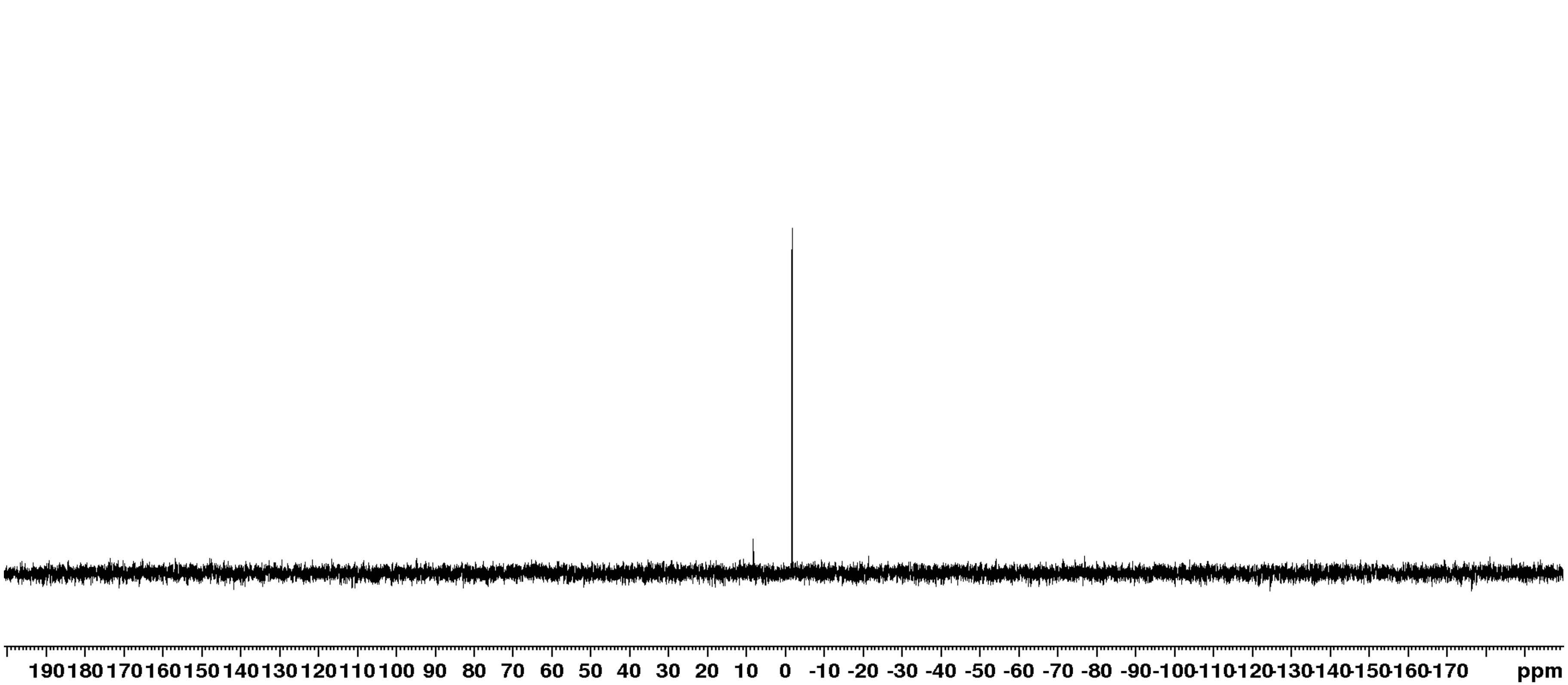

Figure S17. ^{1}H NMR spectrum (500 MHz, $CDCl_3$, 298 K) of buta-2,3-dien-2-yl(butyl)dimethylsilane (**1g**)

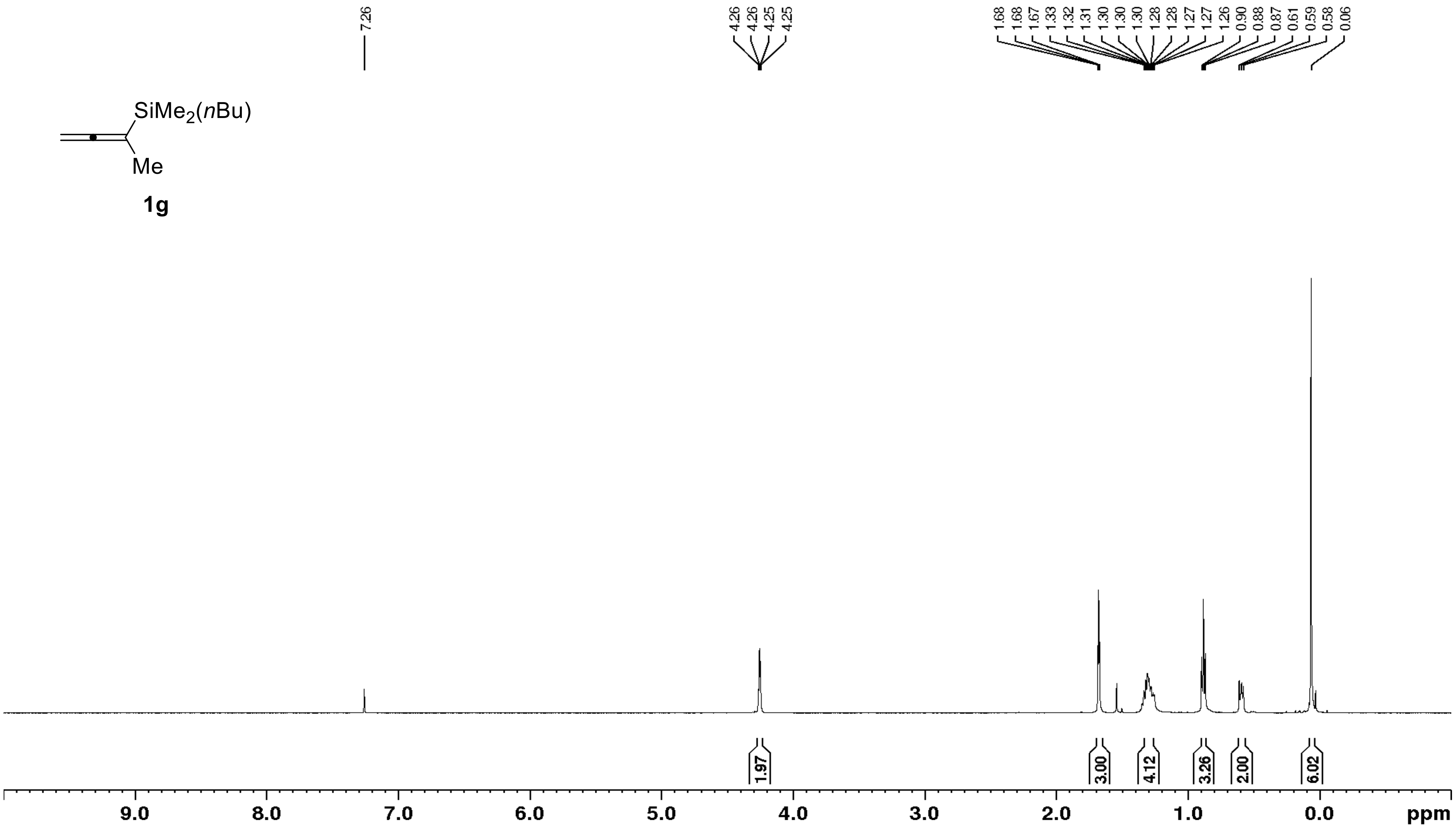

Figure S18. $^{13}C\{^{1}H\}$ NMR spectrum (101 MHz, $CDCl_3$, 298 K) of buta-2,3-dien-2-yl(butyl)dimethylsilane (**1g**)

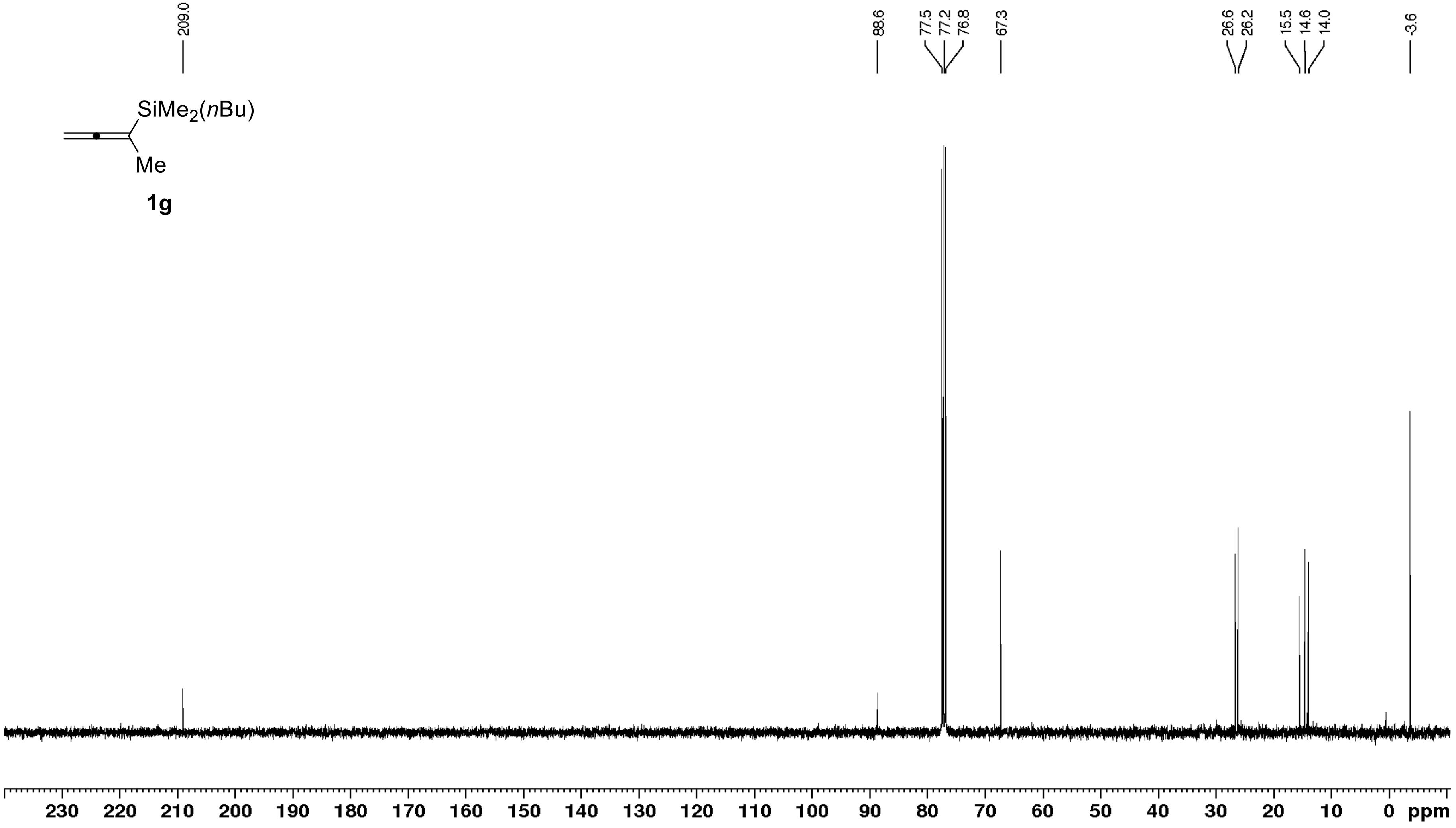

Figure S19. ^{29}Si DEPT NMR spectrum (79 MHz, $CDCl_3$, 298 K, optimized for *J* = 27.0 Hz) of buta-2,3-dien-2-yl(butyl)dimethylsilane (**1g**)

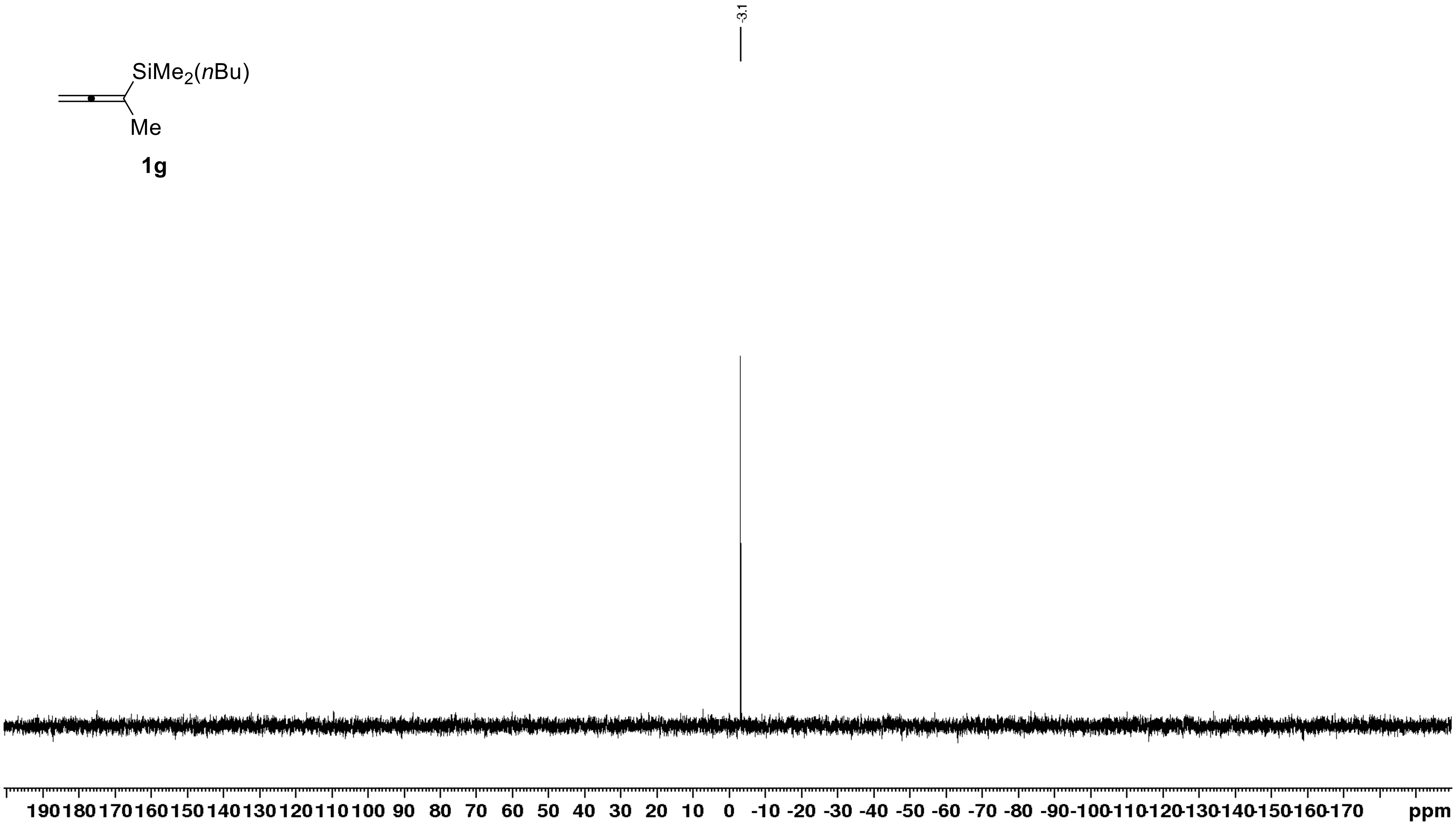

1g

Figure S20. ^{1}H NMR spectrum (500 MHz, $CDCl_3$, 298 K) of buta-2,3-dien-2-yl(*iso*propyl)dimethylsilane (**1h**)

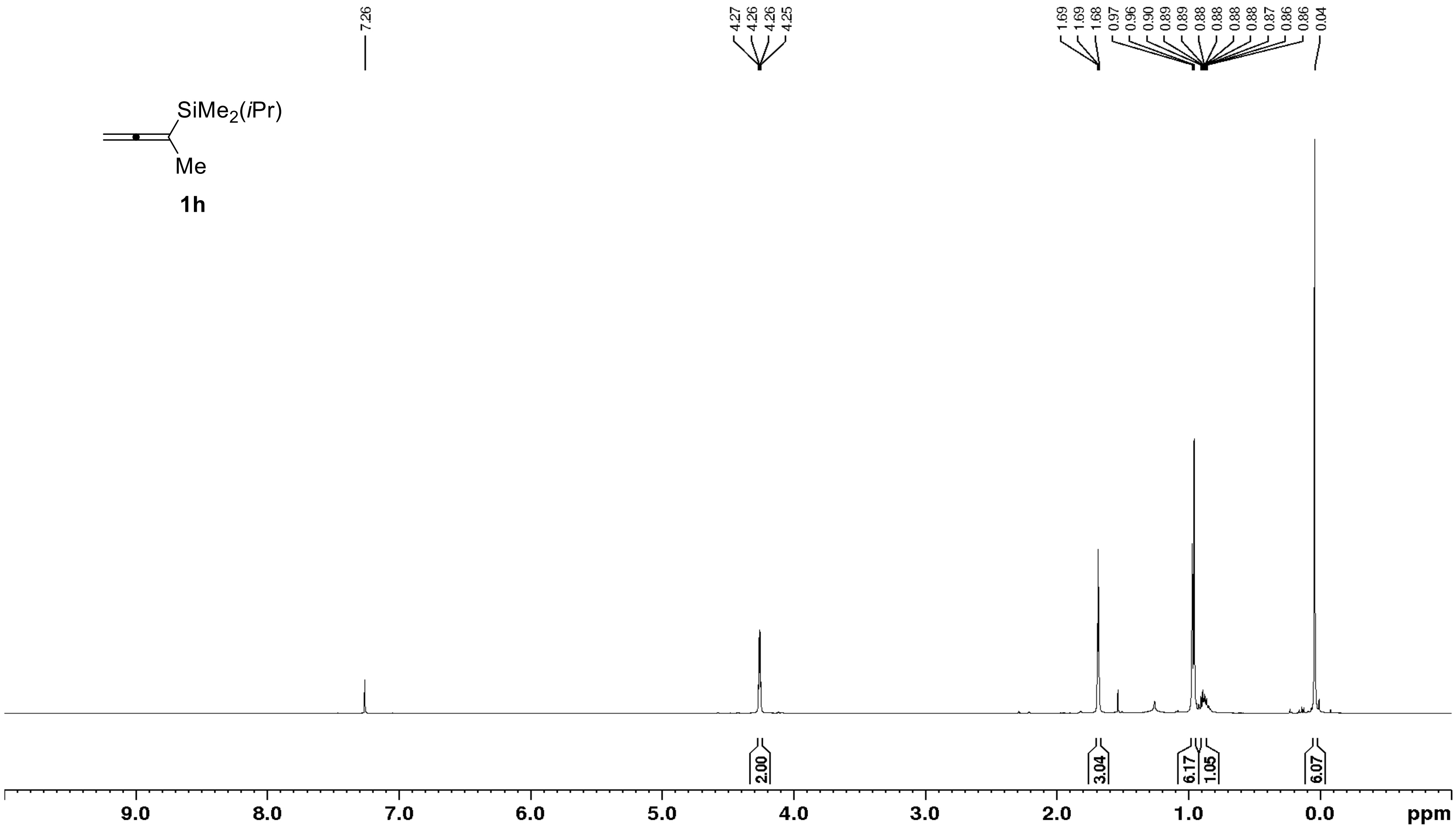

Figure S21. $^{13}C\{^{1}H\}$ NMR spectrum (126 MHz, $CDCl_3$, 298 K) of buta-2,3-dien-2-yl(*iso*propyl)dimethylsilane (**1h**)

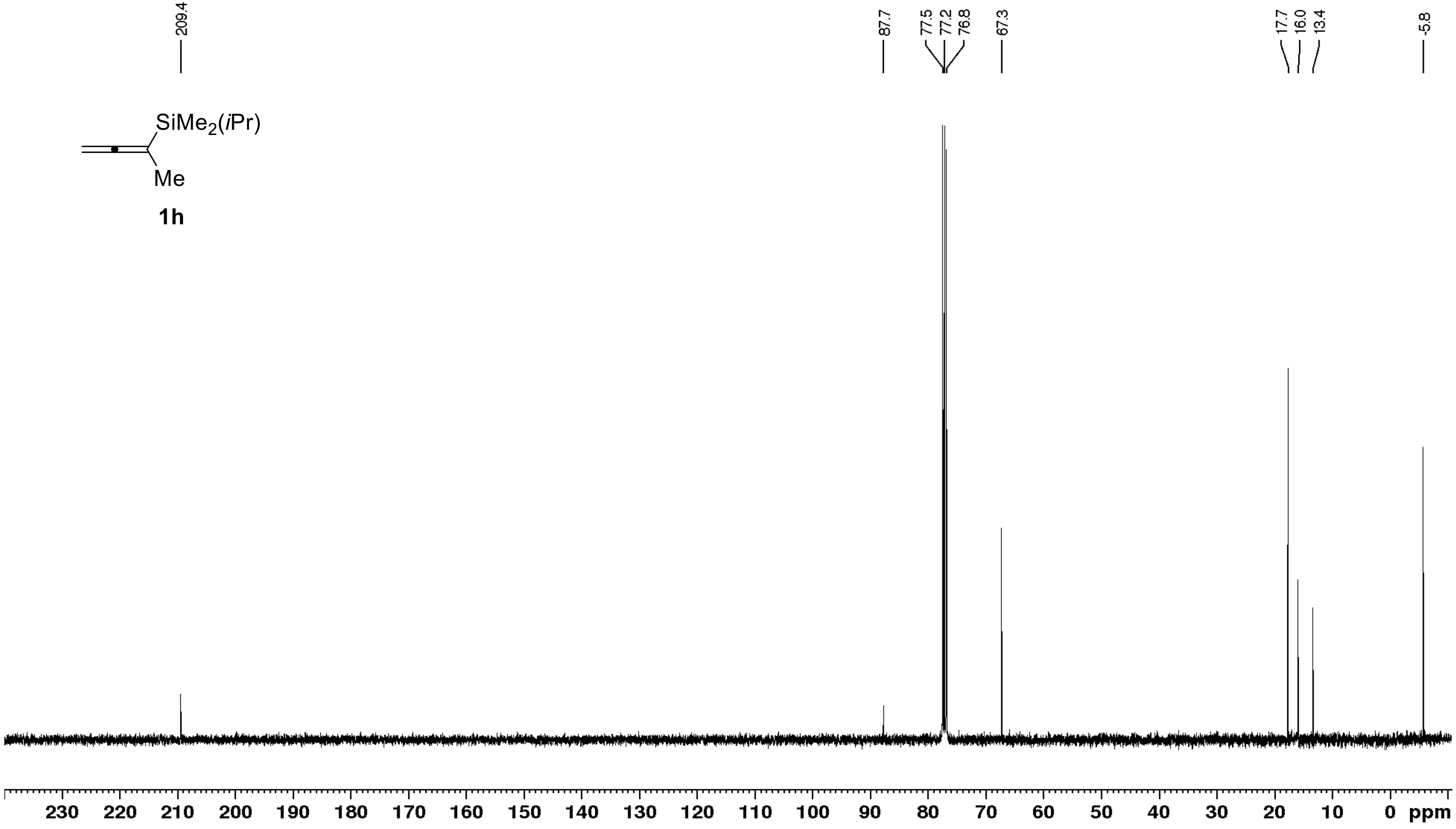

Figure S22. ^{29}Si DEPT NMR spectrum (99 MHz, $CDCl_3$, 298 K, optimized for J = 15.0 Hz) of buta-2,3-dien-2-yl(*iso*propyl)dimethylsilane (**1h**)

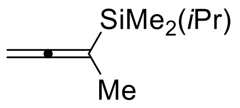

1h

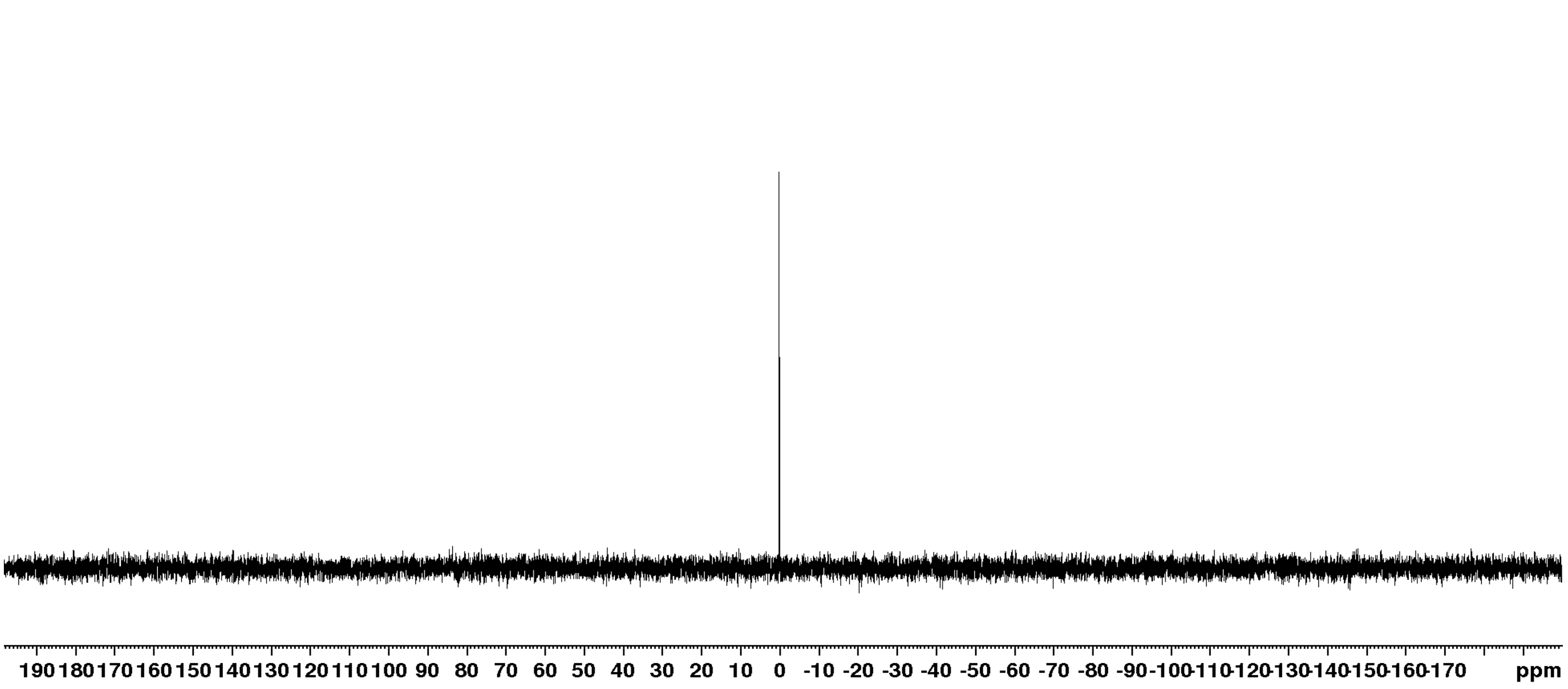

Figure S23. ^{1}H NMR spectrum (500 MHz, $CDCl_3$, 298 K) of buta-2,3-dien-2-yltriethylsilane (**1i**)

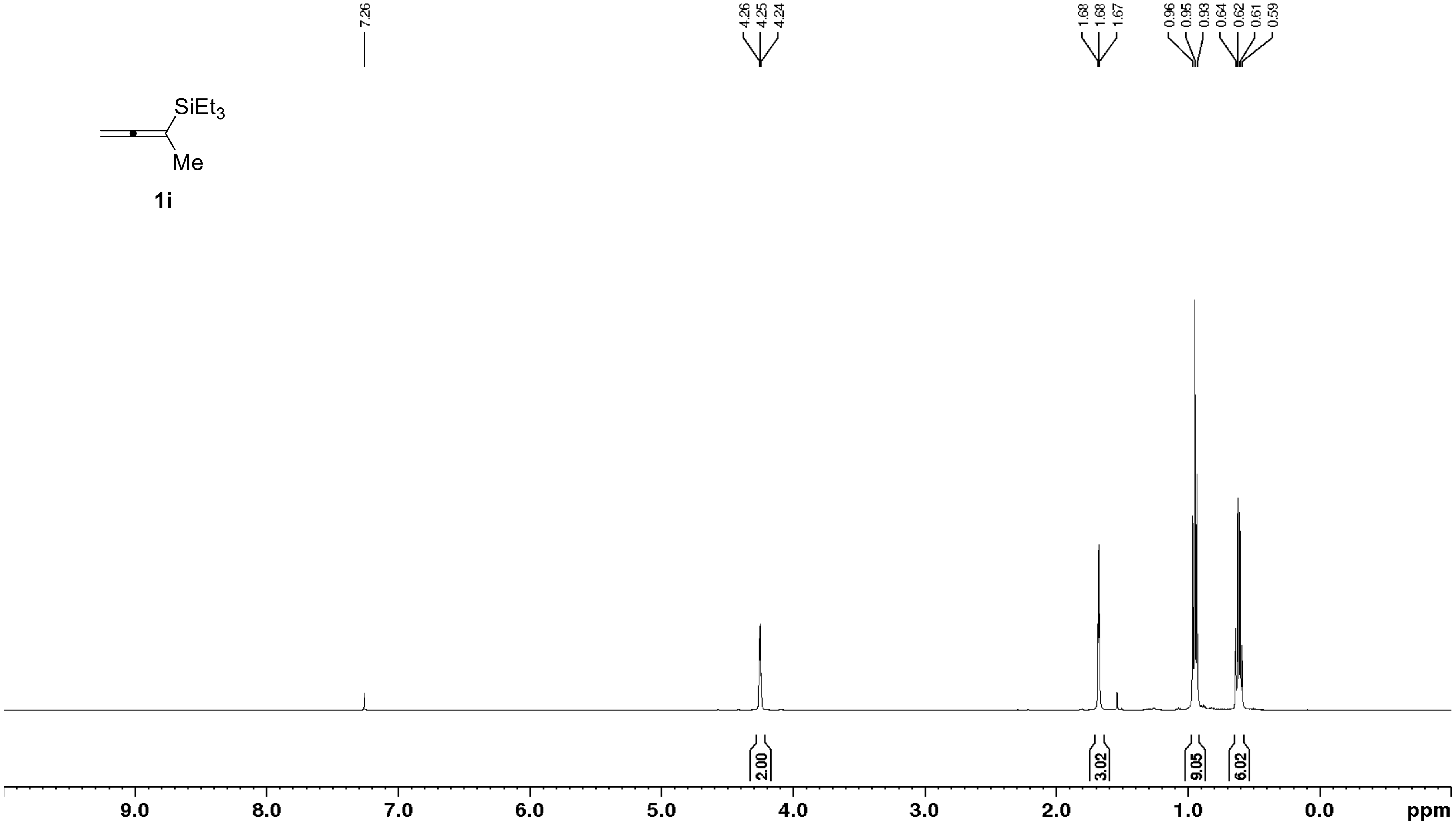

Figure S24. $^{13}C\{^1H\}$ NMR spectrum (126 MHz, $CDCl_3$, 298 K) of buta-2,3-dien-2-yltriethylsilane (**1i**)

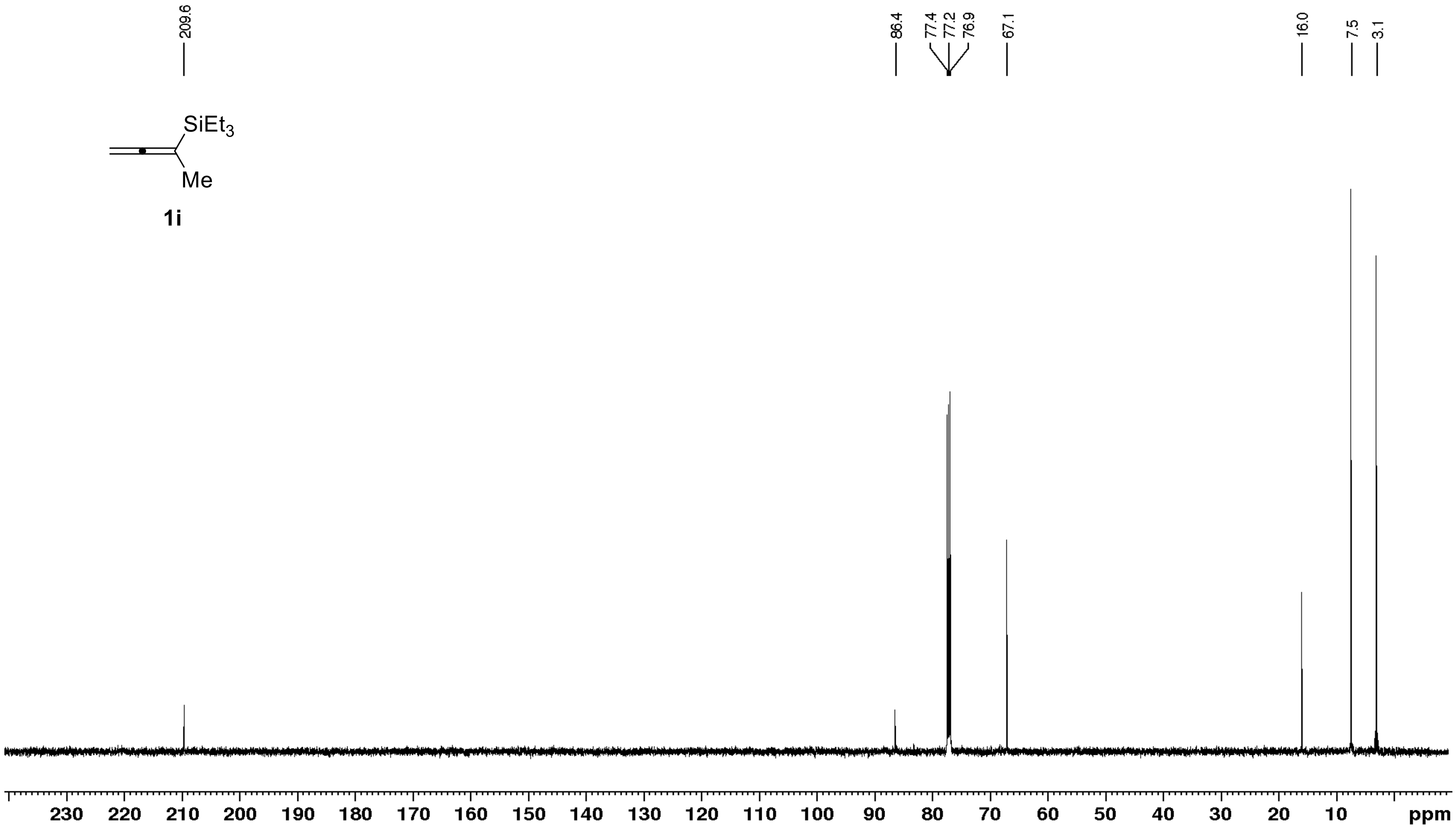

Figure S25. ^{29}Si DEPT NMR spectrum (99 MHz, $CDCl_3$, 298 K, optimized for J = 31.0 Hz) of buta-2,3-dien-2-yltriethylsilane (**1i**)

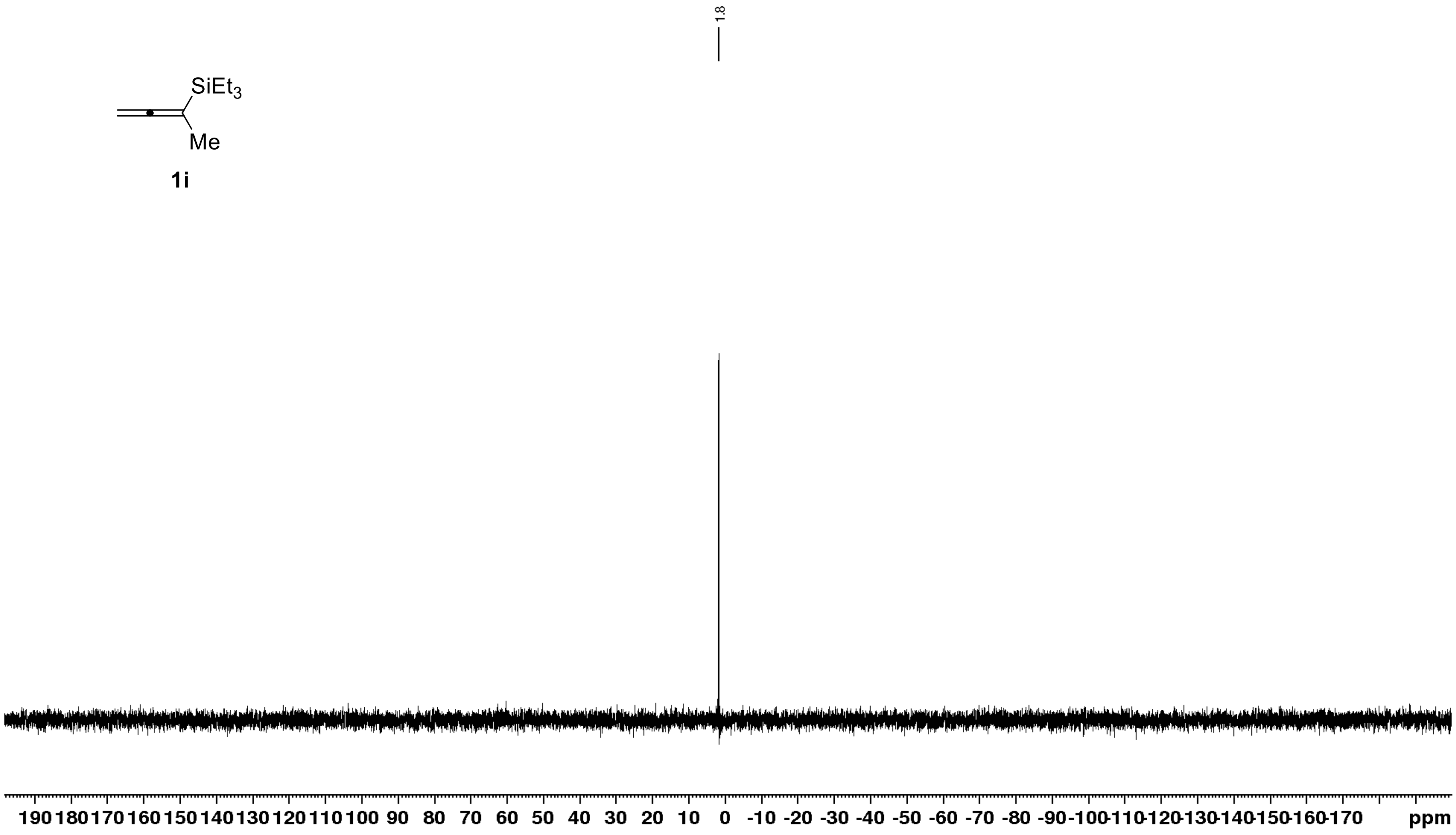

Figure S26. ^{1}H NMR spectrum (500 MHz, $CDCl_3$, 298 K) of **3aa**

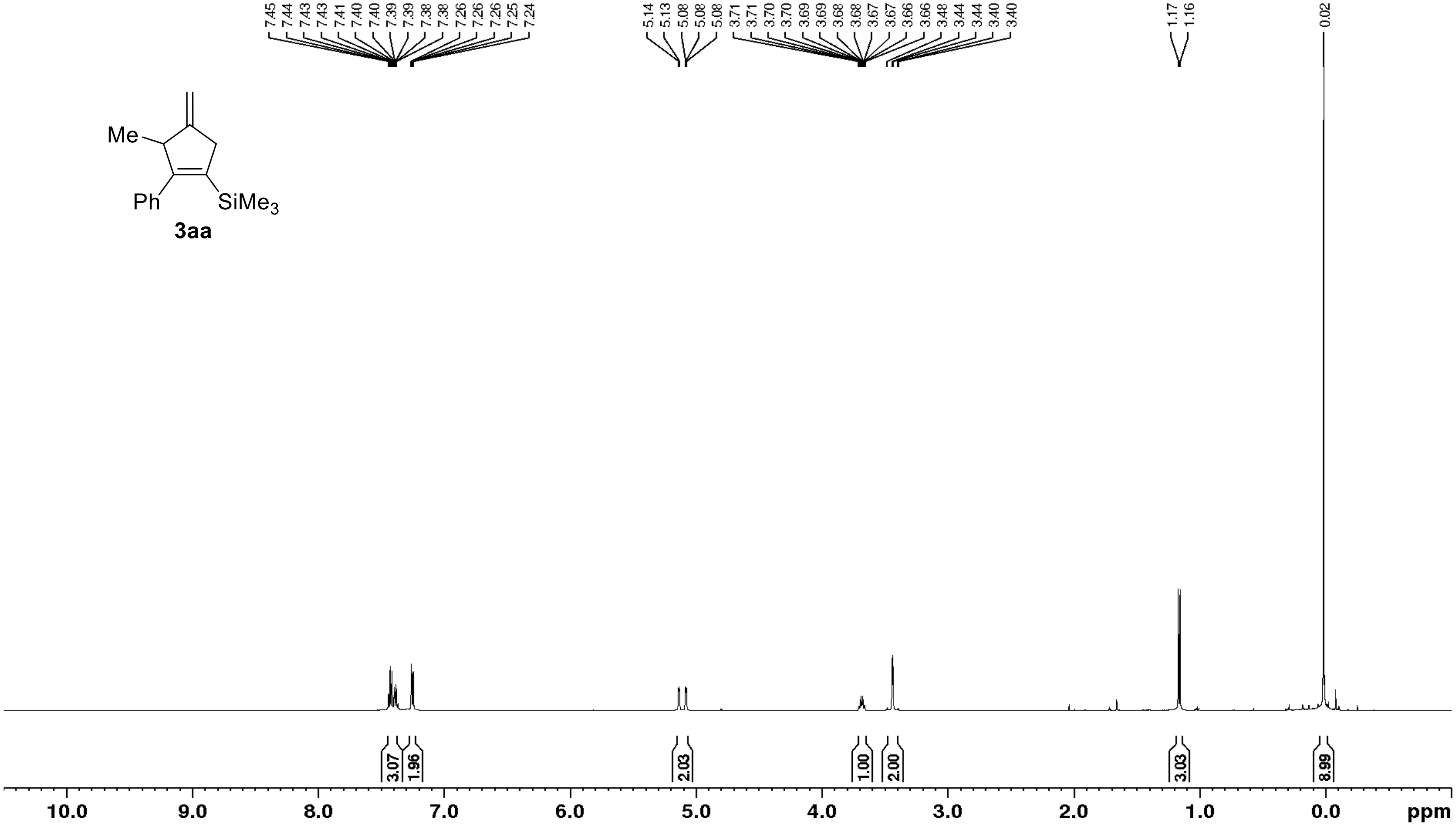

Figure S27. $^{13}C\{^1H\}$ NMR spectrum (126 MHz, $CDCl_3$, 298 K) of **3aa**

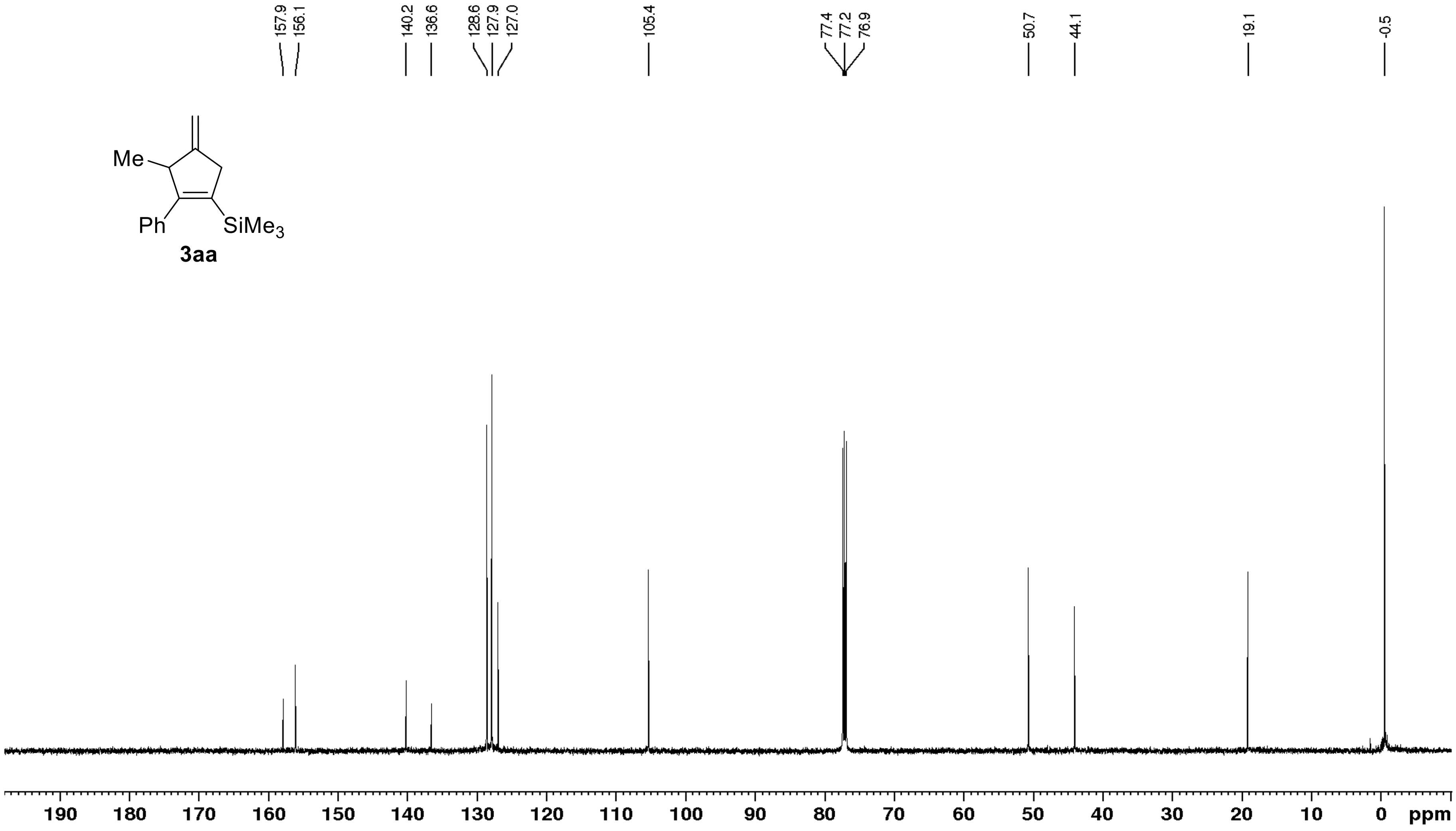

Figure S28. ^{29}Si DEPT NMR spectrum (99 MHz, $CDCl_3$, 298 K, optimized for J = 7.0 Hz) of **3aa**

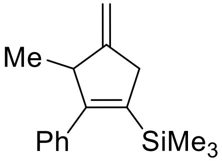

3aa

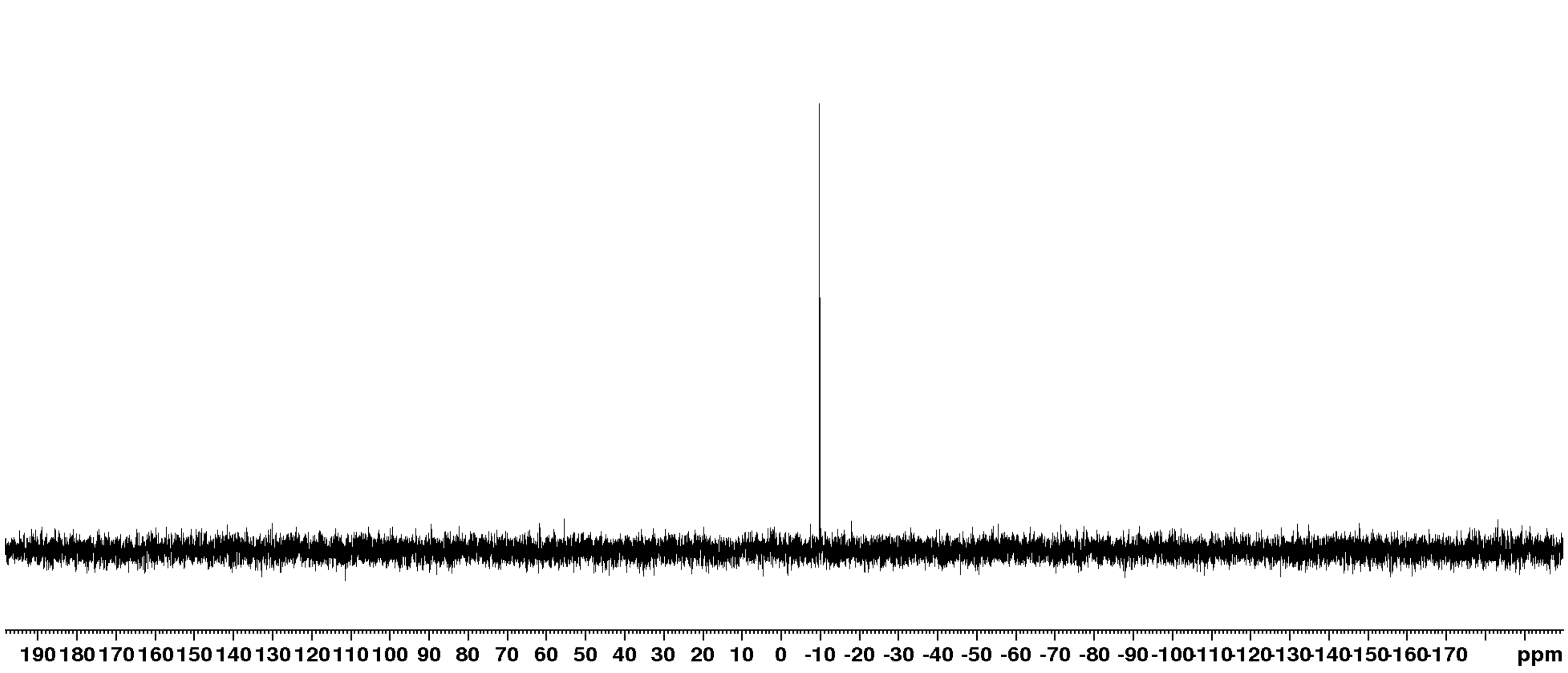

Figure S29. ¹H NMR spectrum (400 MHz, CDCl3, 298 K) of **3ab**

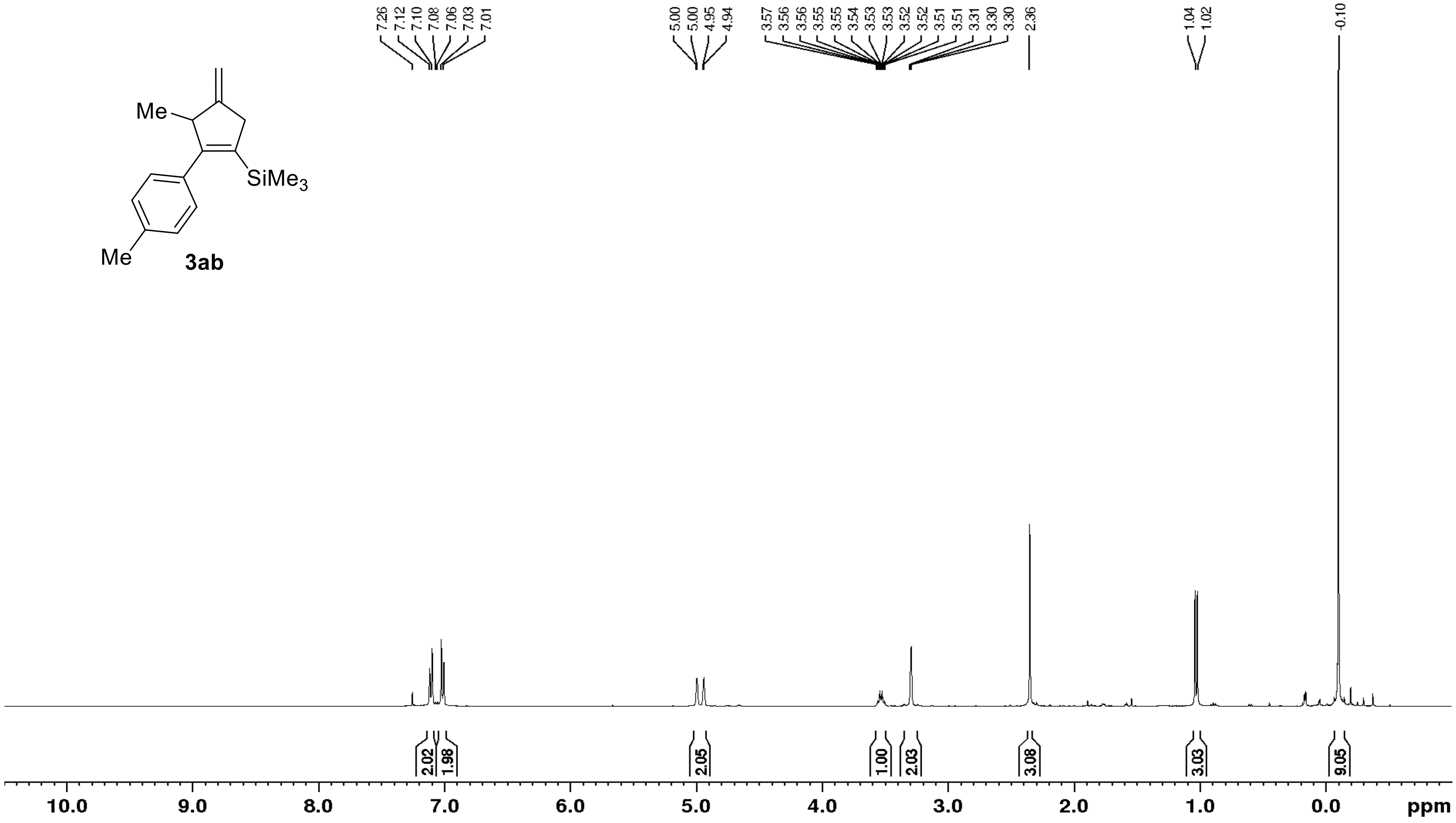

Figure S30. $^{13}C\{^{1}H\}$ NMR spectrum (101 MHz, $CDCl_3$, 298 K) of **3ab**

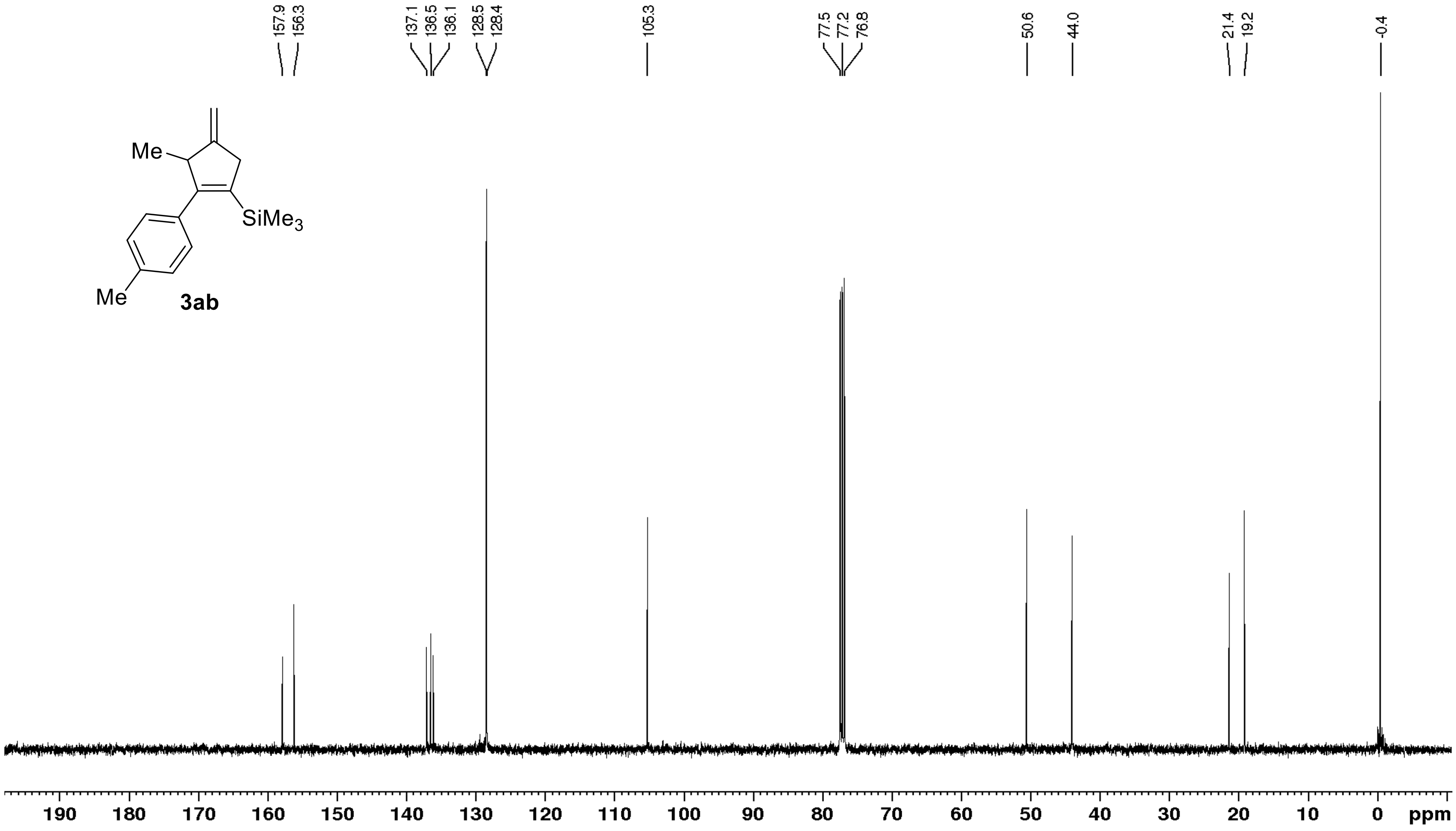

Figure S31. ^{29}Si DEPT NMR spectrum (79 MHz, $CDCl_3$, 298 K, optimized for J = 7.0 Hz) of **3ab**

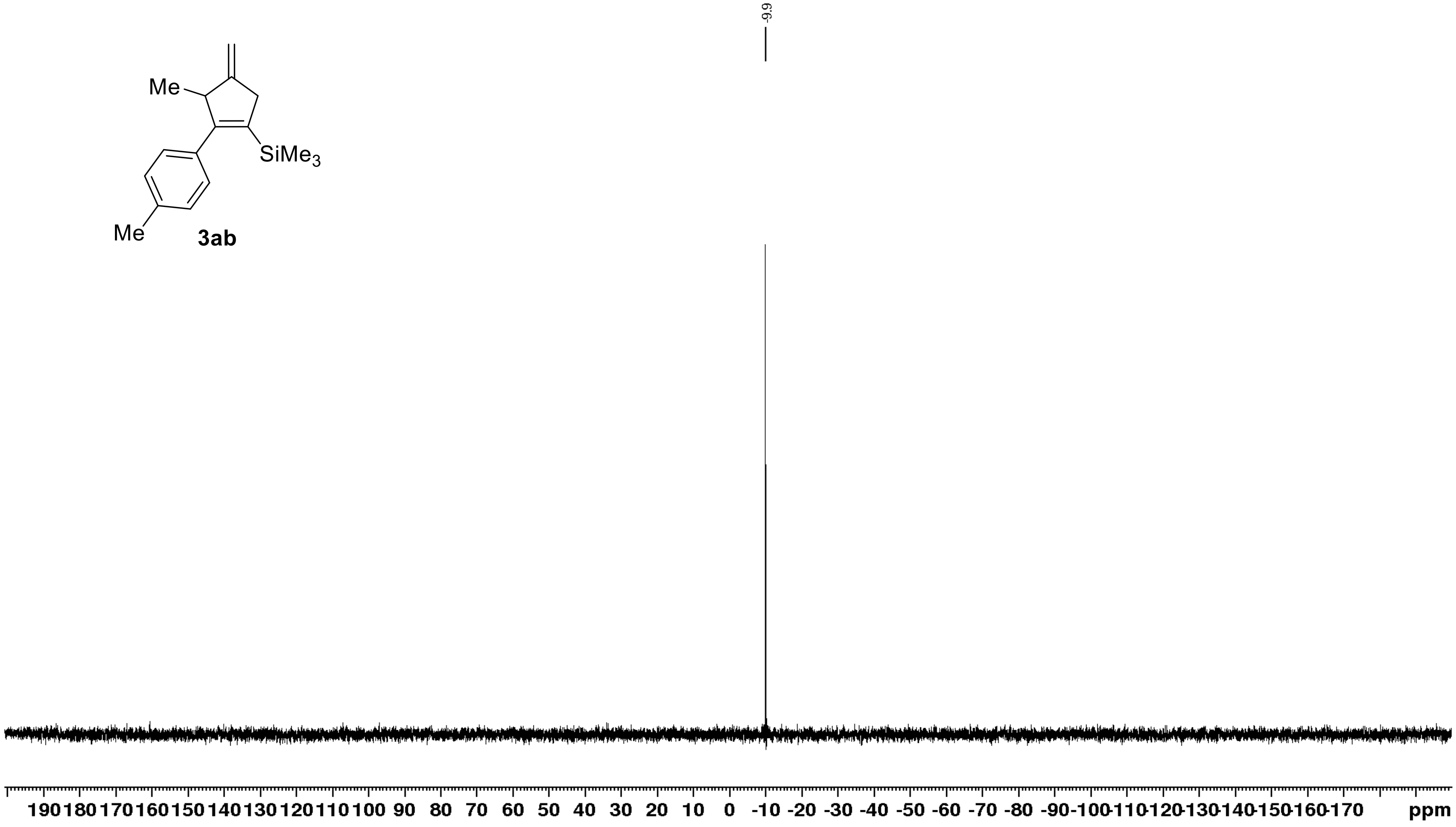

Figure S32. ^{1}H NMR spectrum (500 MHz, $CDCl_3$, 298 K) of **3ac**

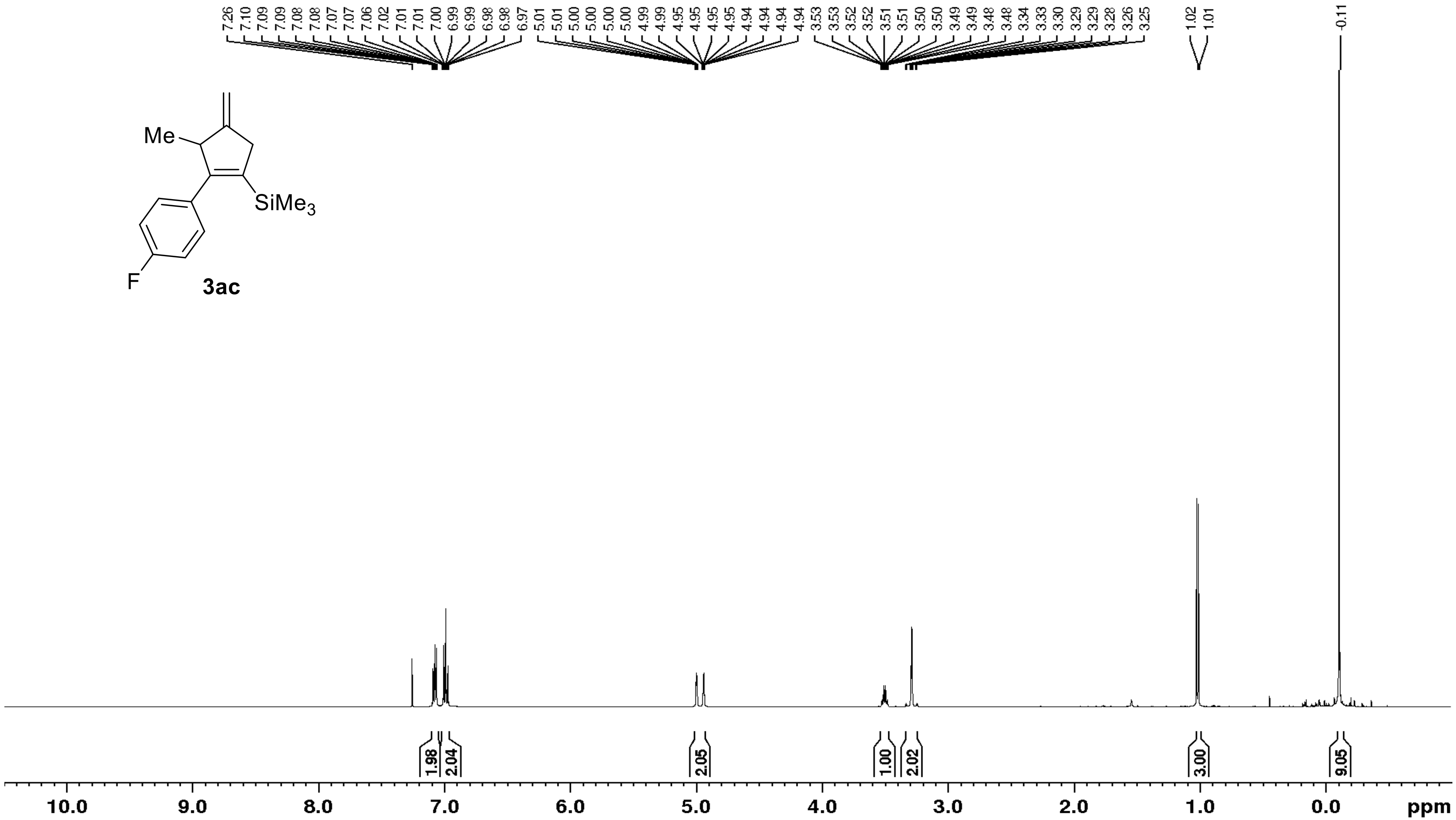

3ac

Figure S33. $^{13}C\{^{1}H\}$ NMR spectrum (101 MHz, $CDCl_3$, 298 K) of **3ac**

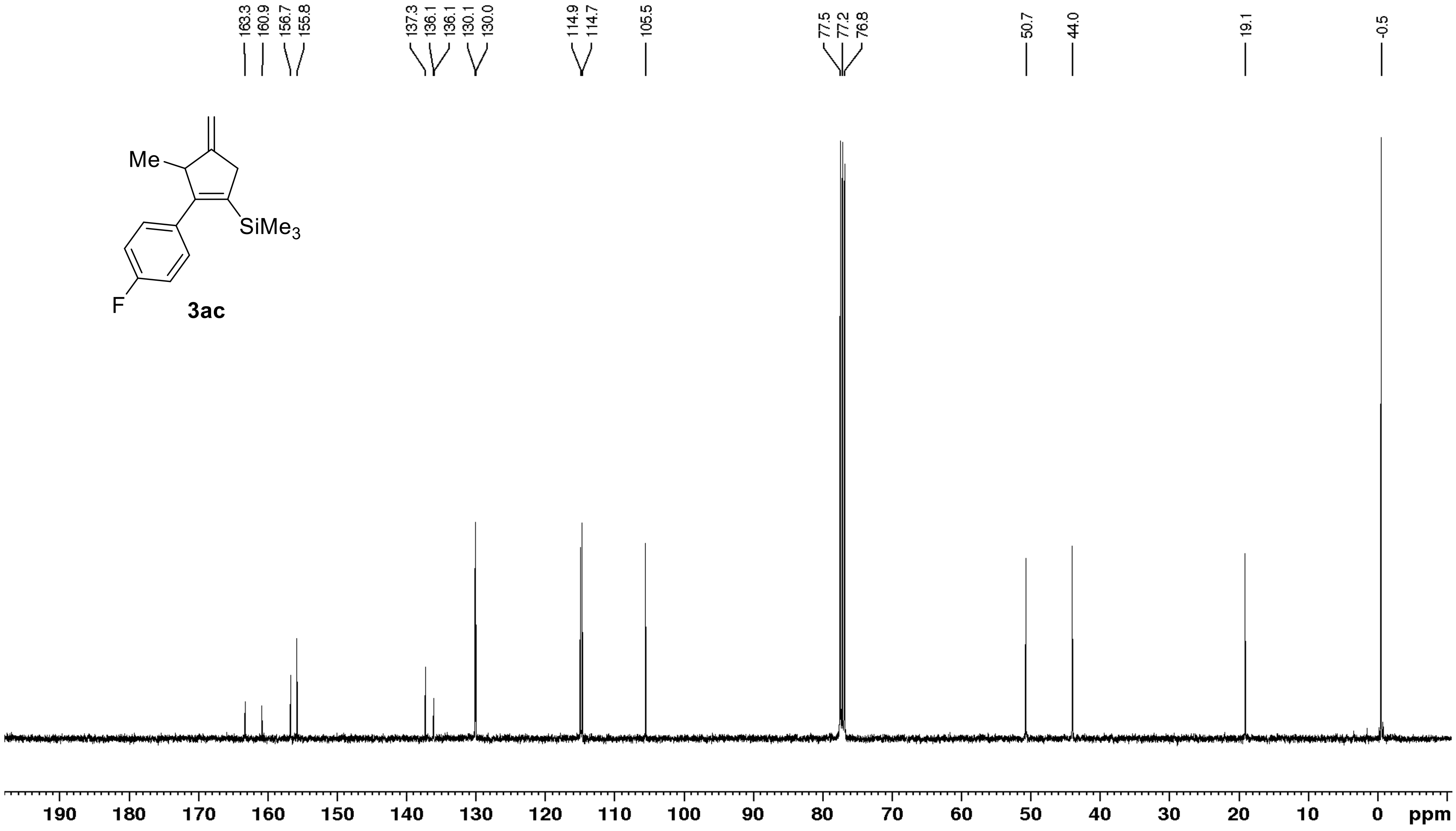

Figure S34. ^{19}F NMR spectrum (471MHz, $CDCl_3$, 298 K) of **3ac**

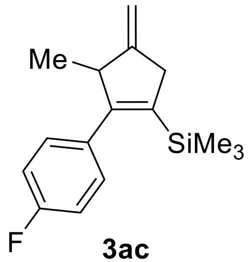

3ac

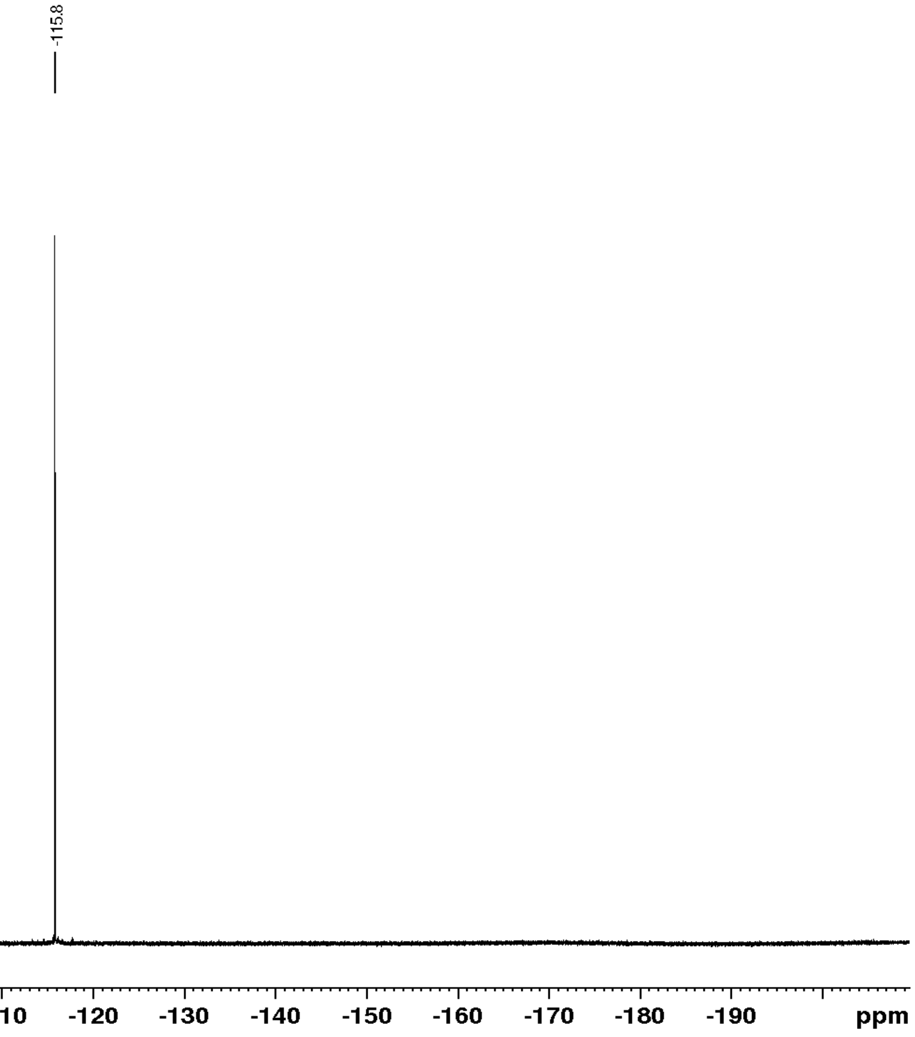

Figure S35. ^{29}Si DEPT NMR spectrum (79 MHz, $CDCl_3$, 298 K, optimized for *J* = 7.0 Hz) of **3ac**

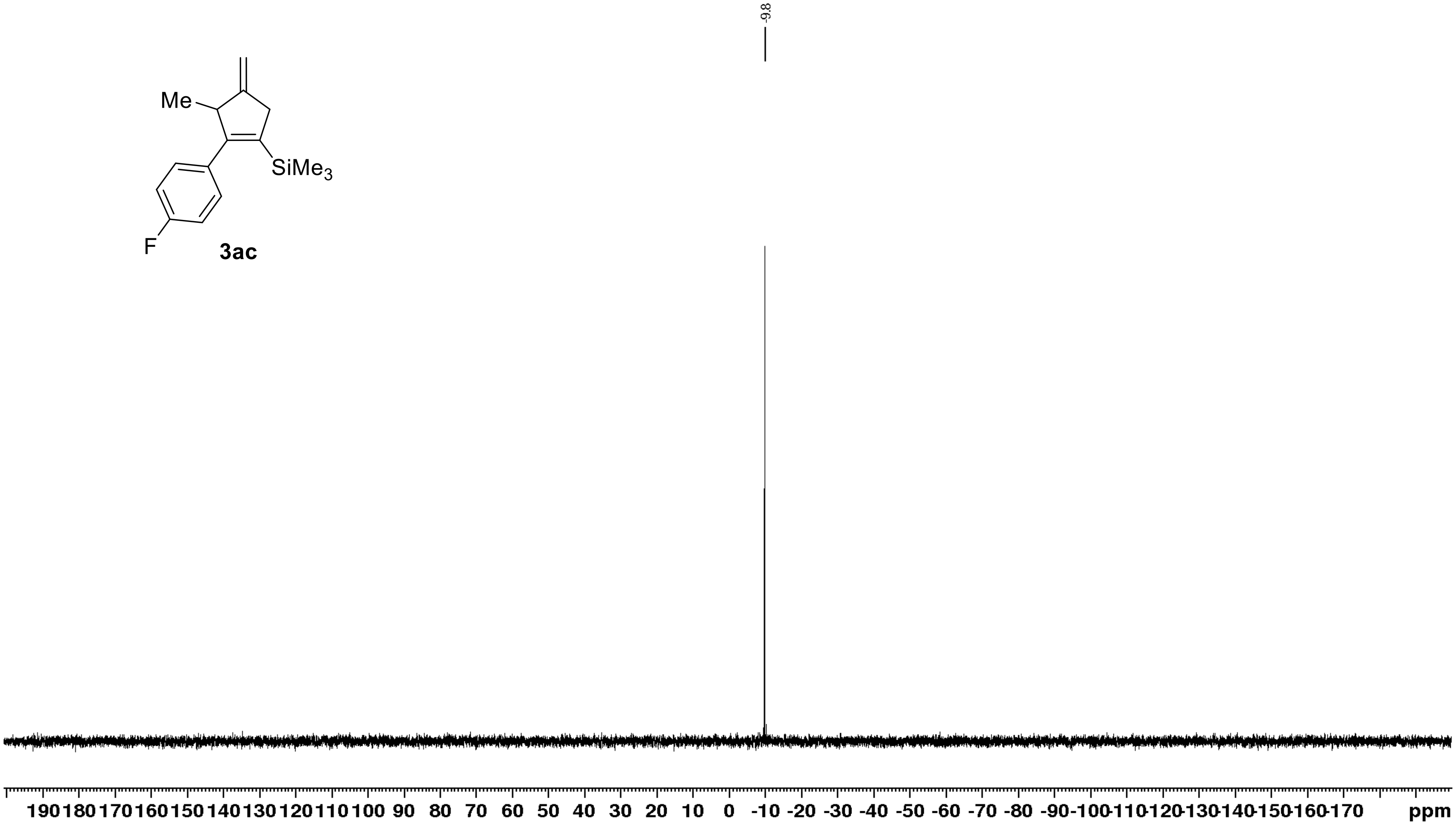

Figure S36. ^{1}H NMR spectrum (400 MHz, $CDCl_3$, 298 K) of **3ad**

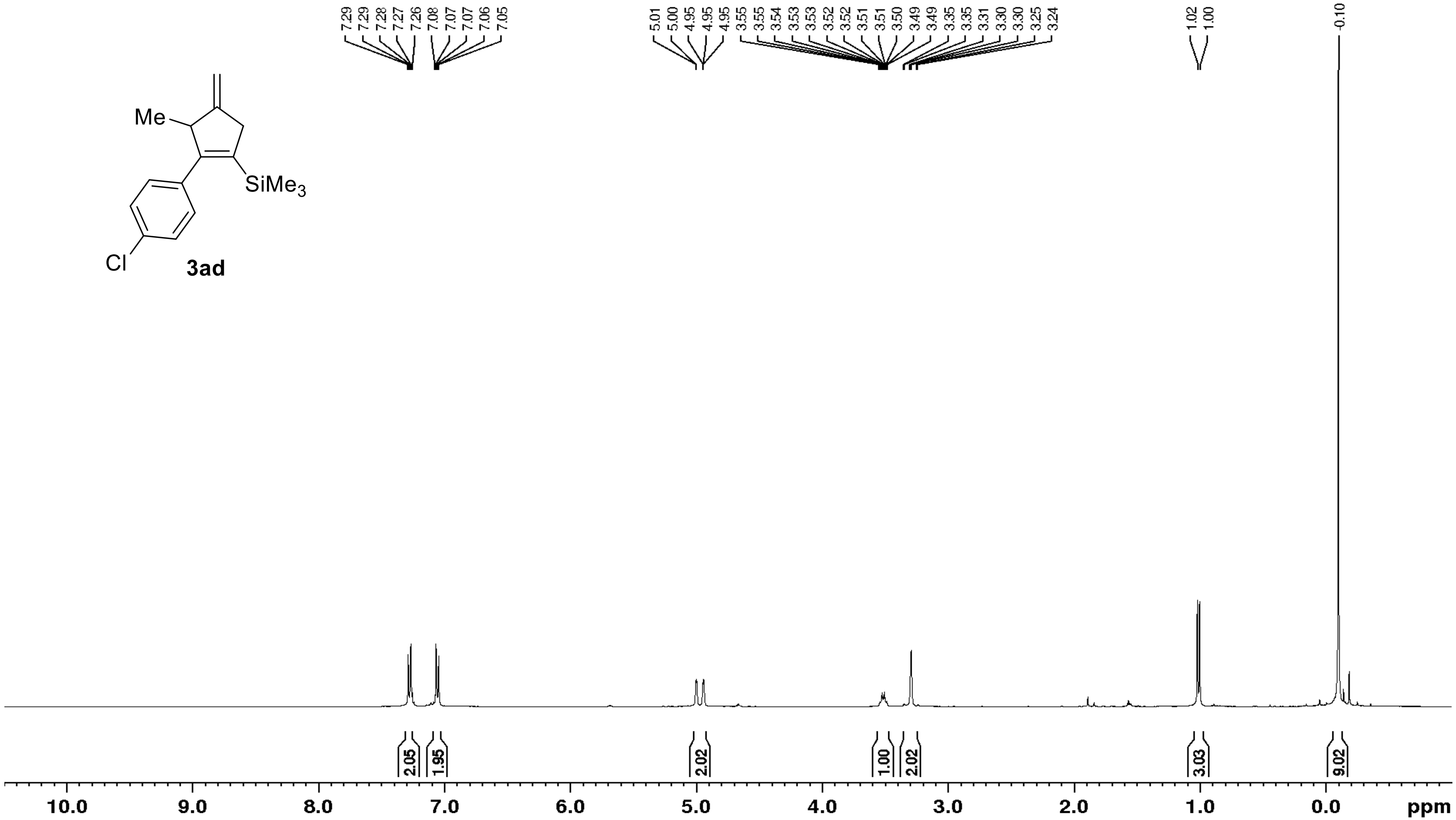

Figure S37. $^{13}C\{^{1}H\}$ NMR spectrum (101 MHz, $CDCl_3$, 298 K) of **3ad**

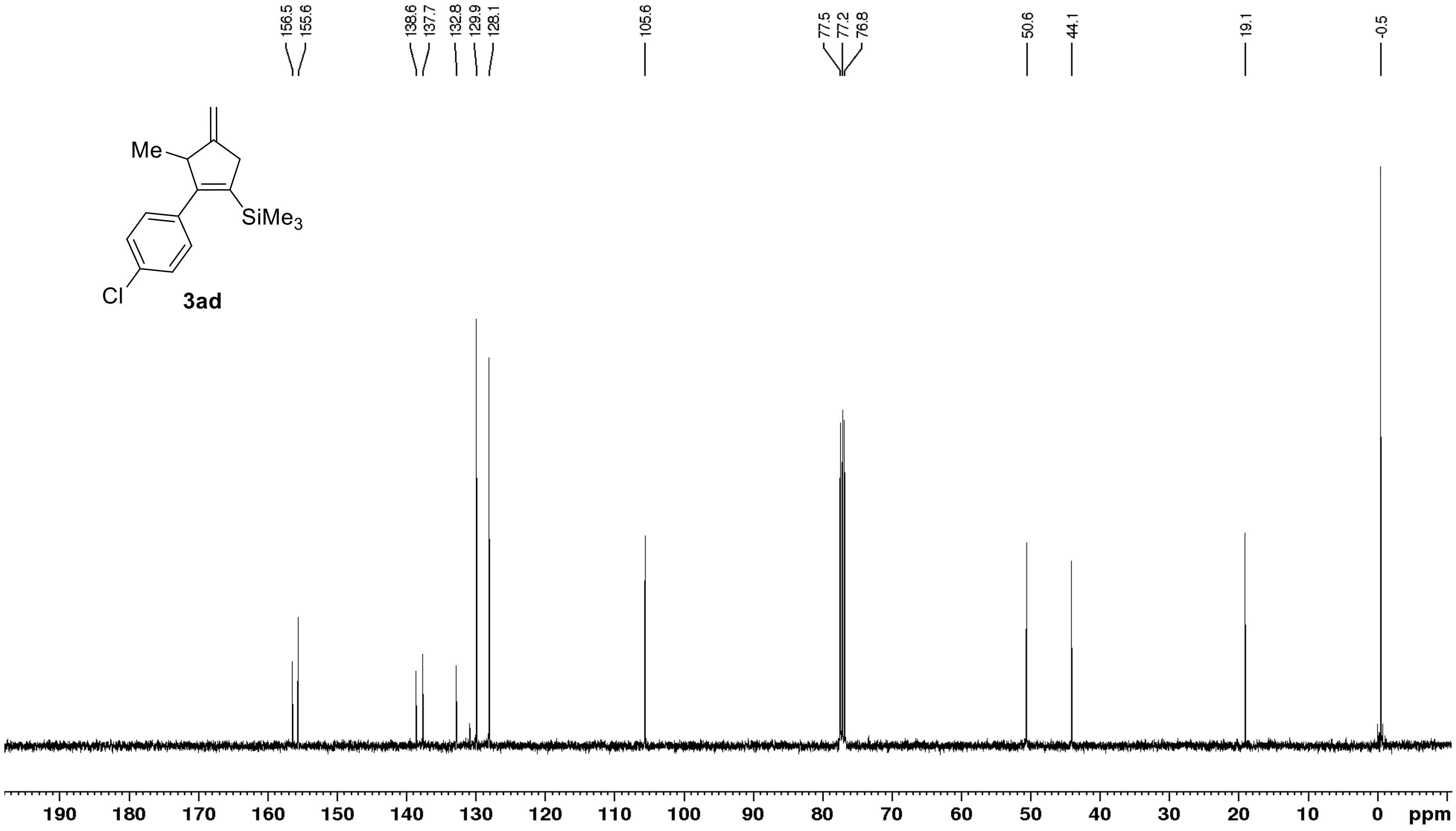

Figure S38. ^{29}Si DEPT NMR spectrum (79 MHz, $CDCl_3$, 298 K, optimized for J = 7.0 Hz) of **3ad**

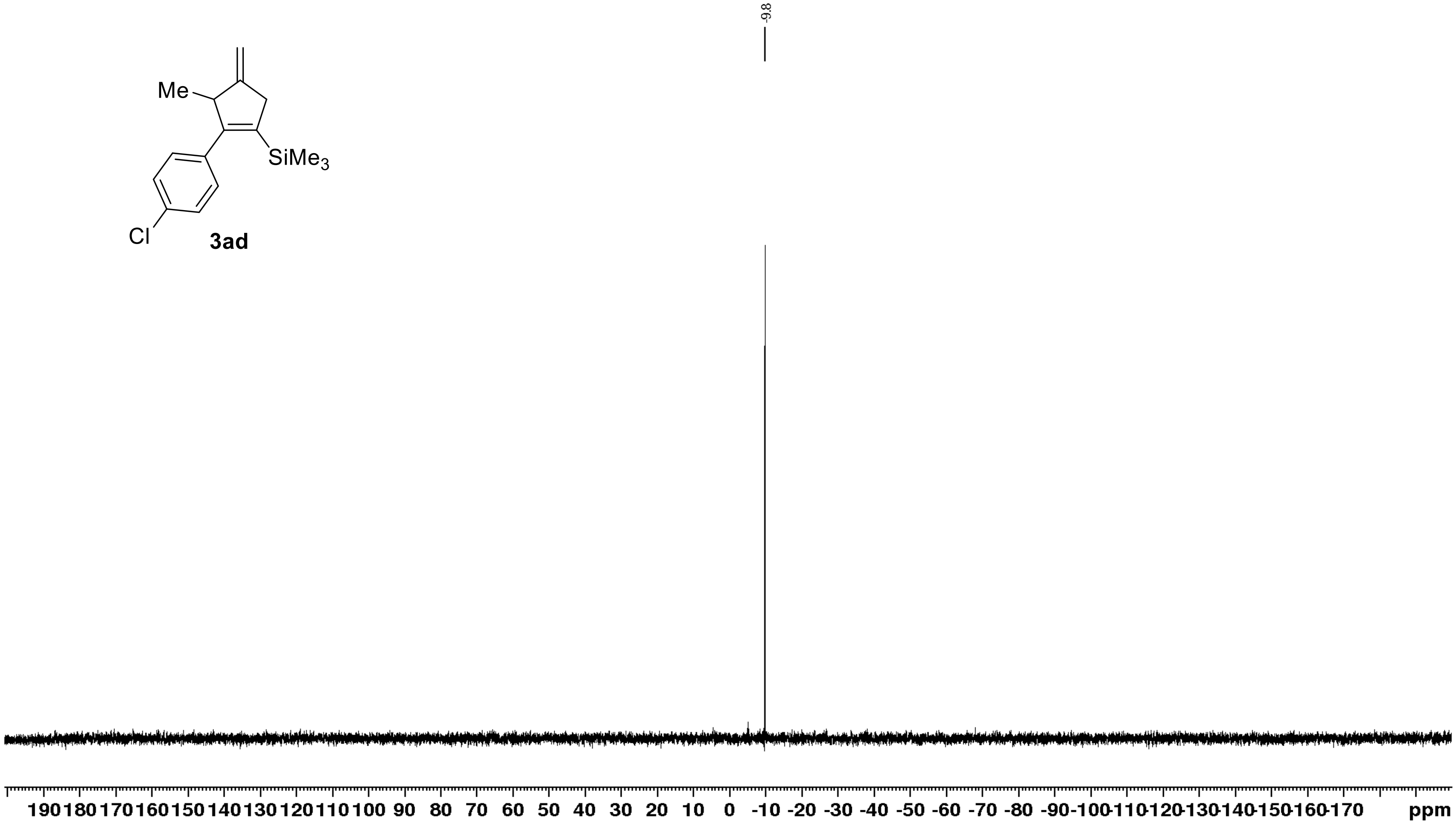

Figure S39. ^{1}H NMR spectrum (500 MHz, $CDCl_3$, 298 K) of **3ae**

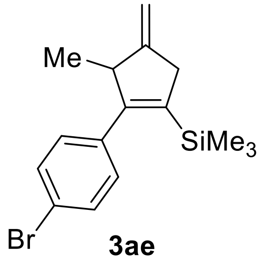

3ae

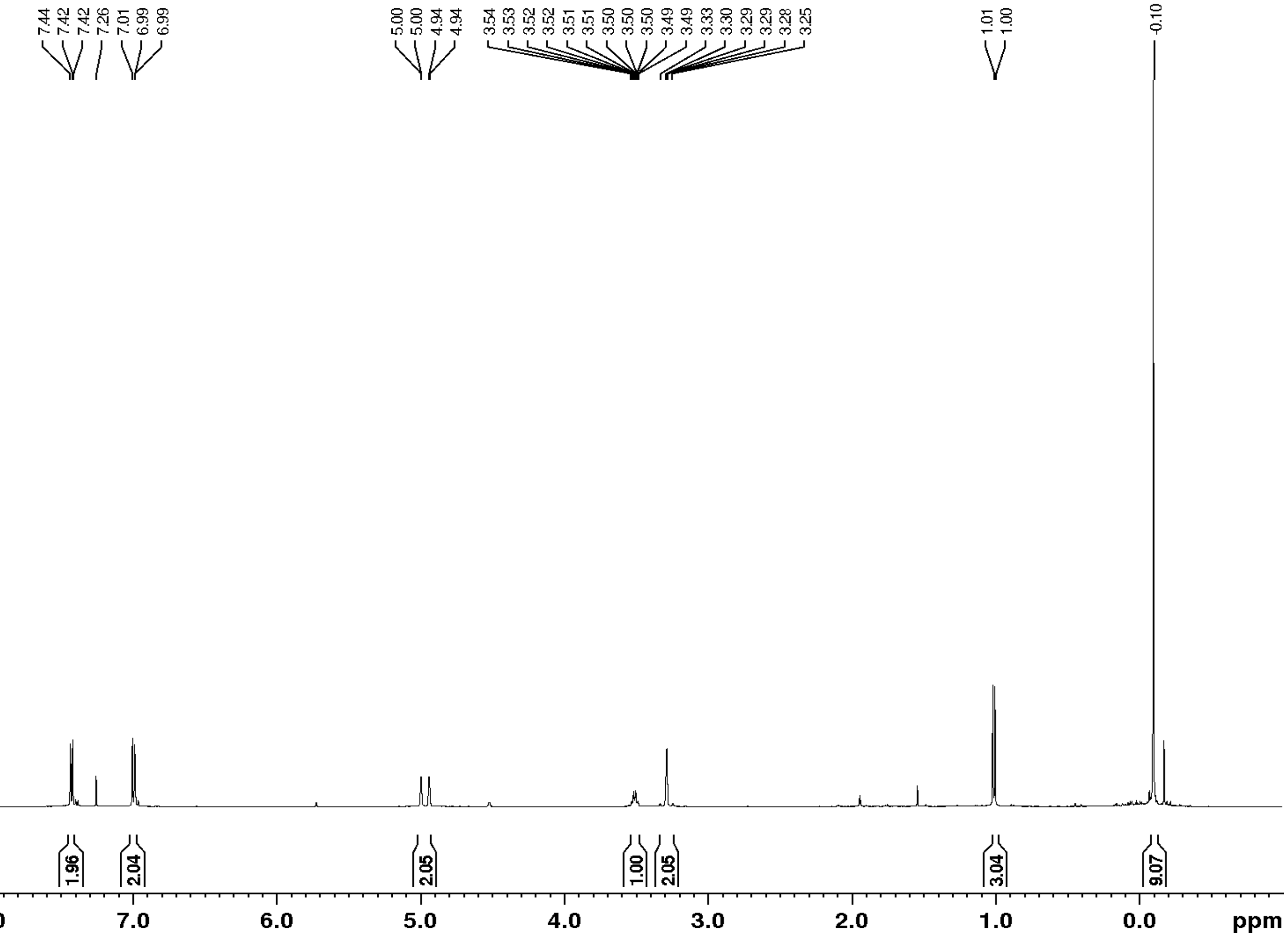

Figure S40. $^{13}C\{^1H\}$ NMR spectrum (126 MHz, $CDCl_3$, 298 K) of **3ae**

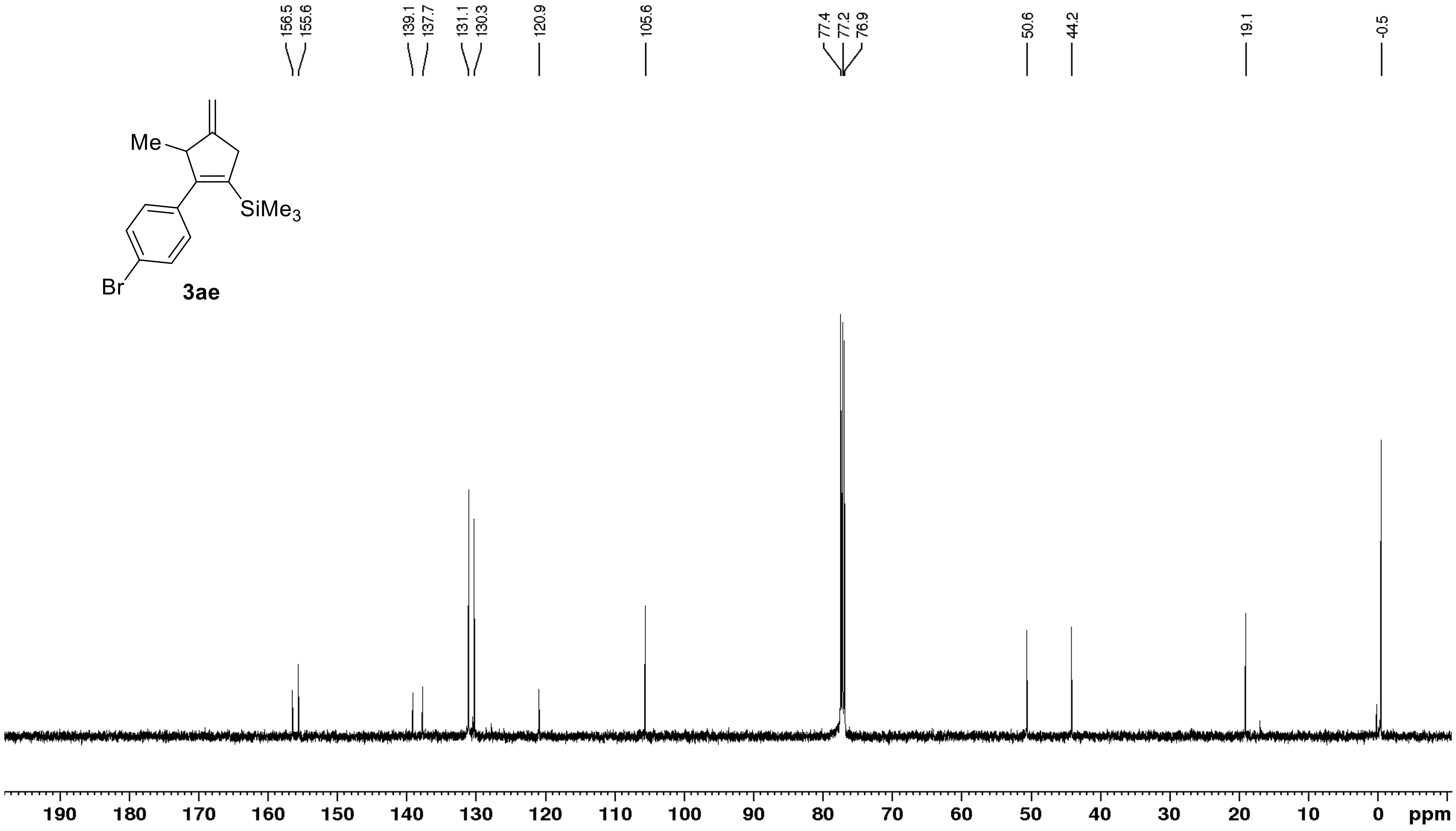

Figure S41. ^{29}Si DEPT NMR spectrum (99 MHz, $CDCl_3$, 298 K, optimized for J = 7.0 Hz) of **3ae**

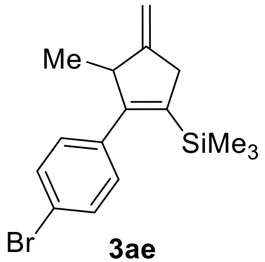

3ae

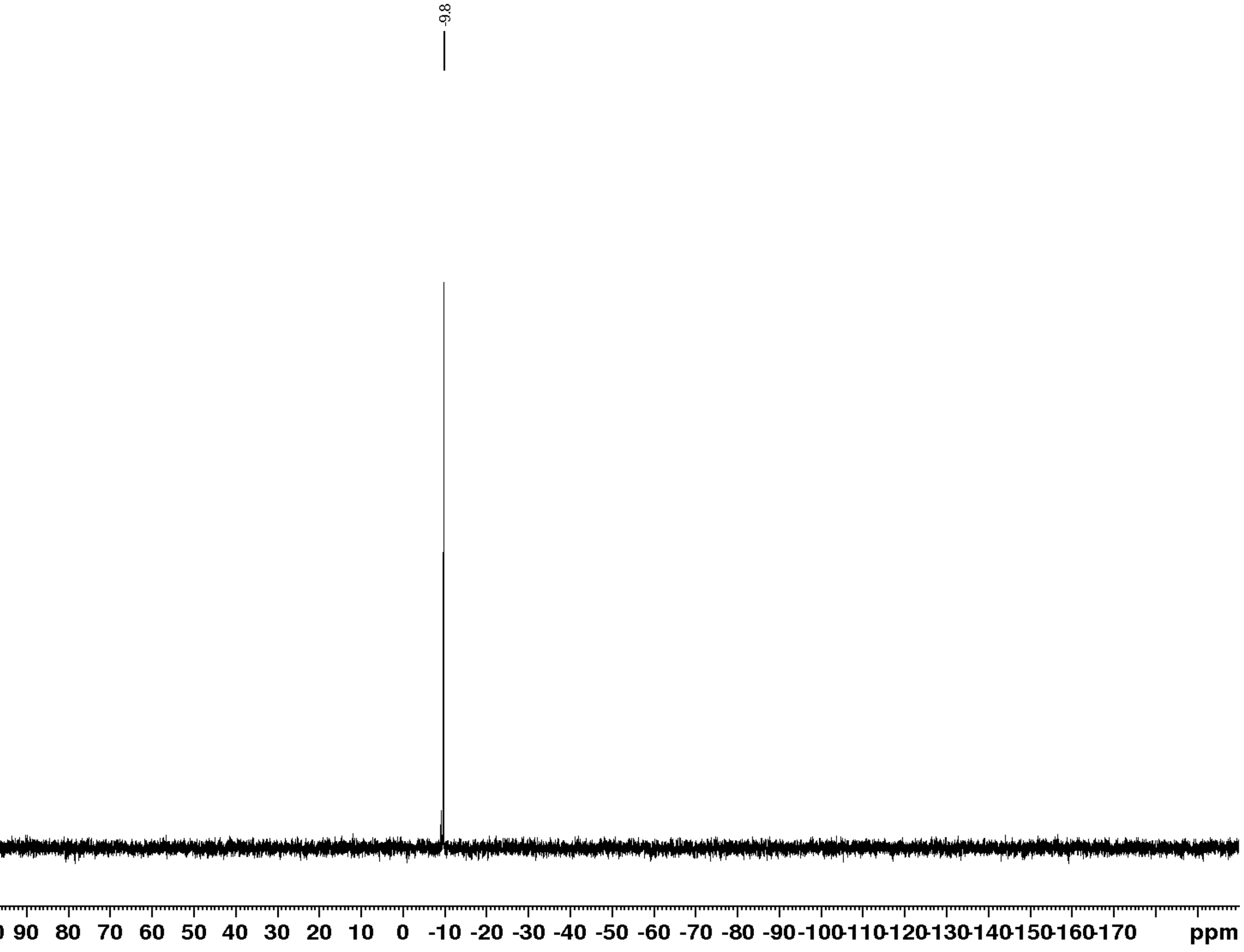

Figure S42. ^{1}H NMR spectrum (500 MHz, $CDCl_3$, 298 K) of **3ag**

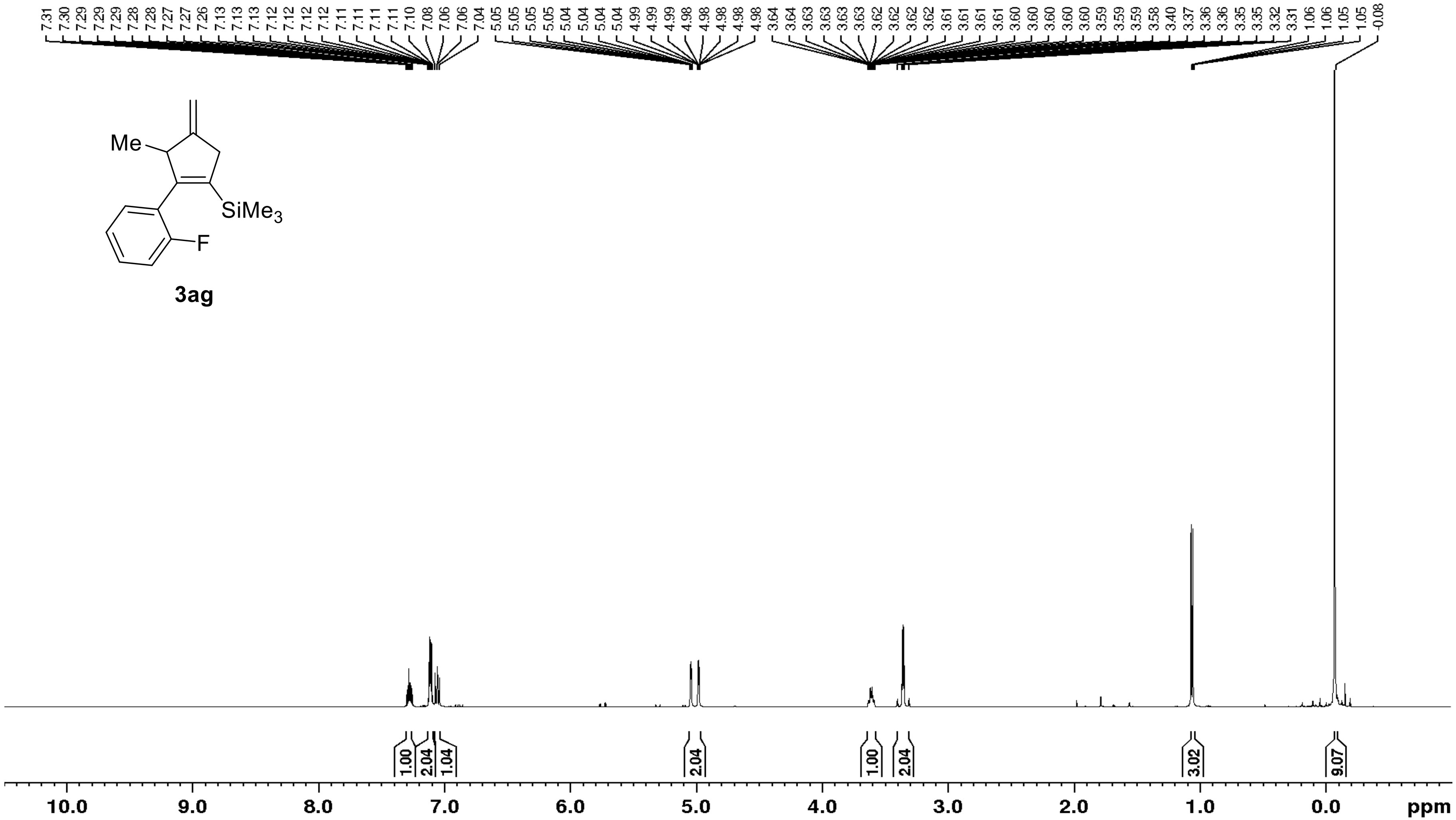

Figure S43. $^{13}C\{^{1}H\}$ NMR spectrum (126 MHz, $CDCl_3$, 298 K) of **3ag**

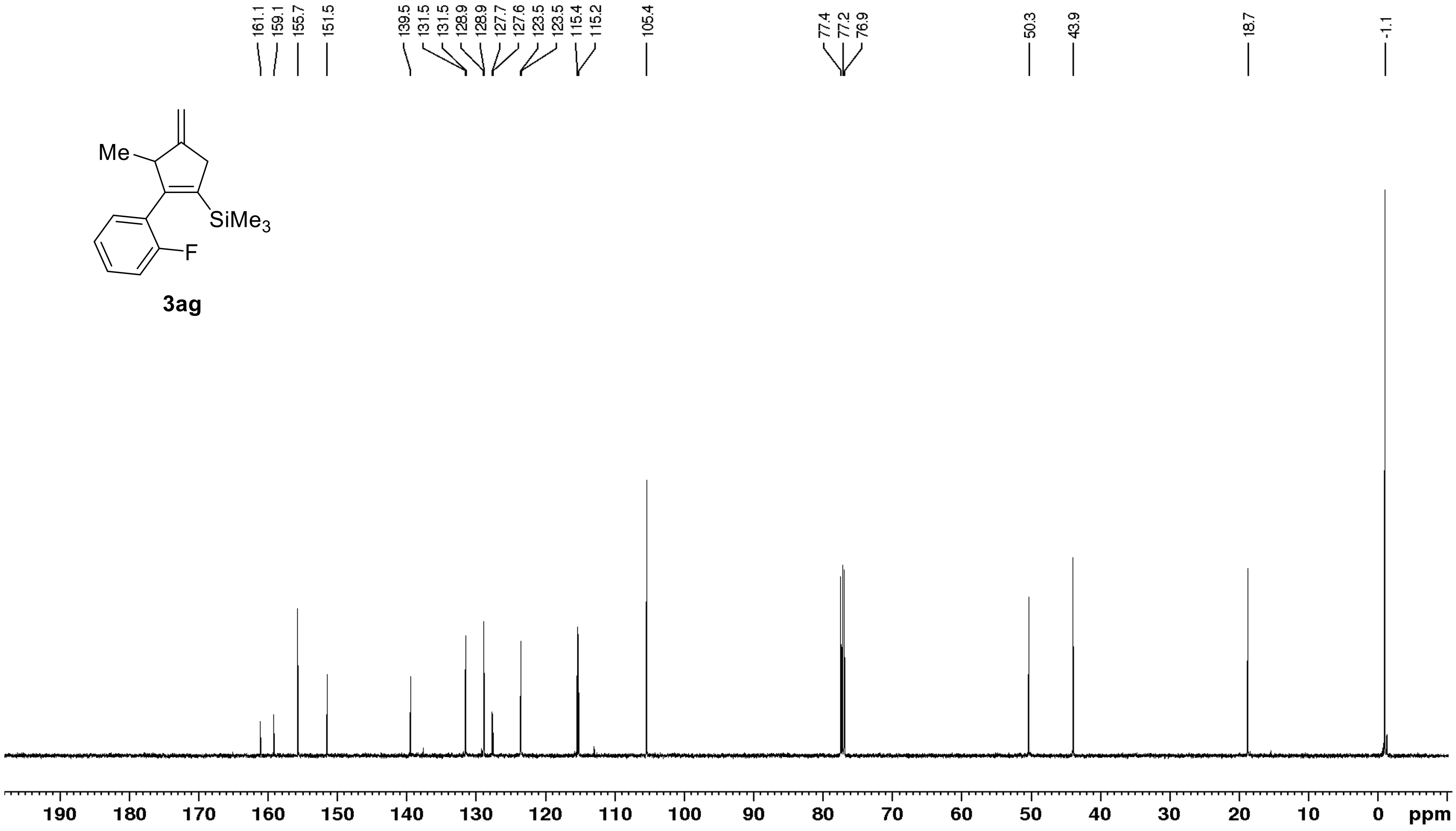

Figure S44. ^{19}F NMR spectrum (471 MHz, $CDCl_3$, 298 K) of **3ag**

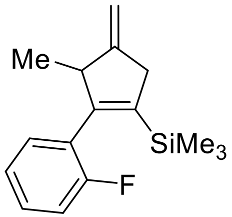

3ag

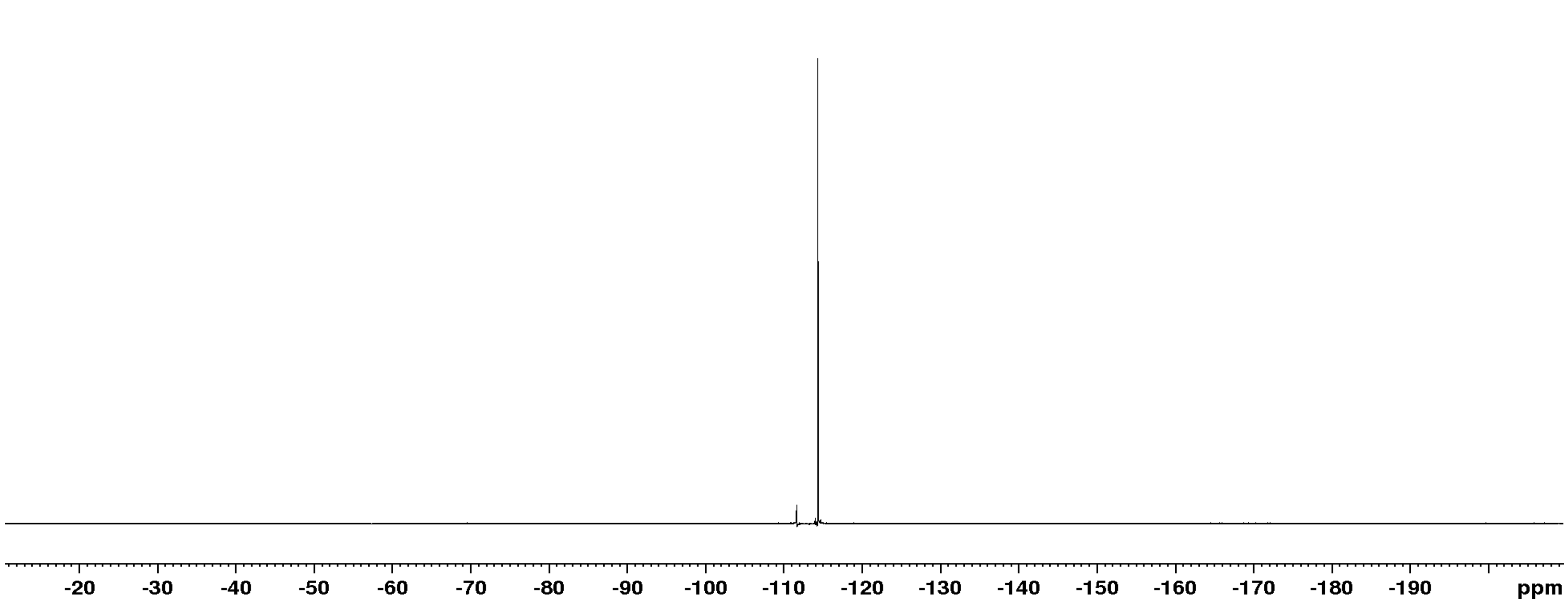

Figure S45. ^{29}Si DEPT NMR spectrum (99 MHz, $CDCl_3$, 298 K, optimized for *J* = 7.0 Hz) of **3ag**

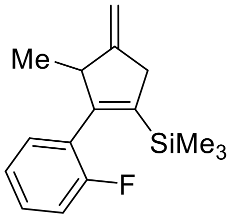

3ag

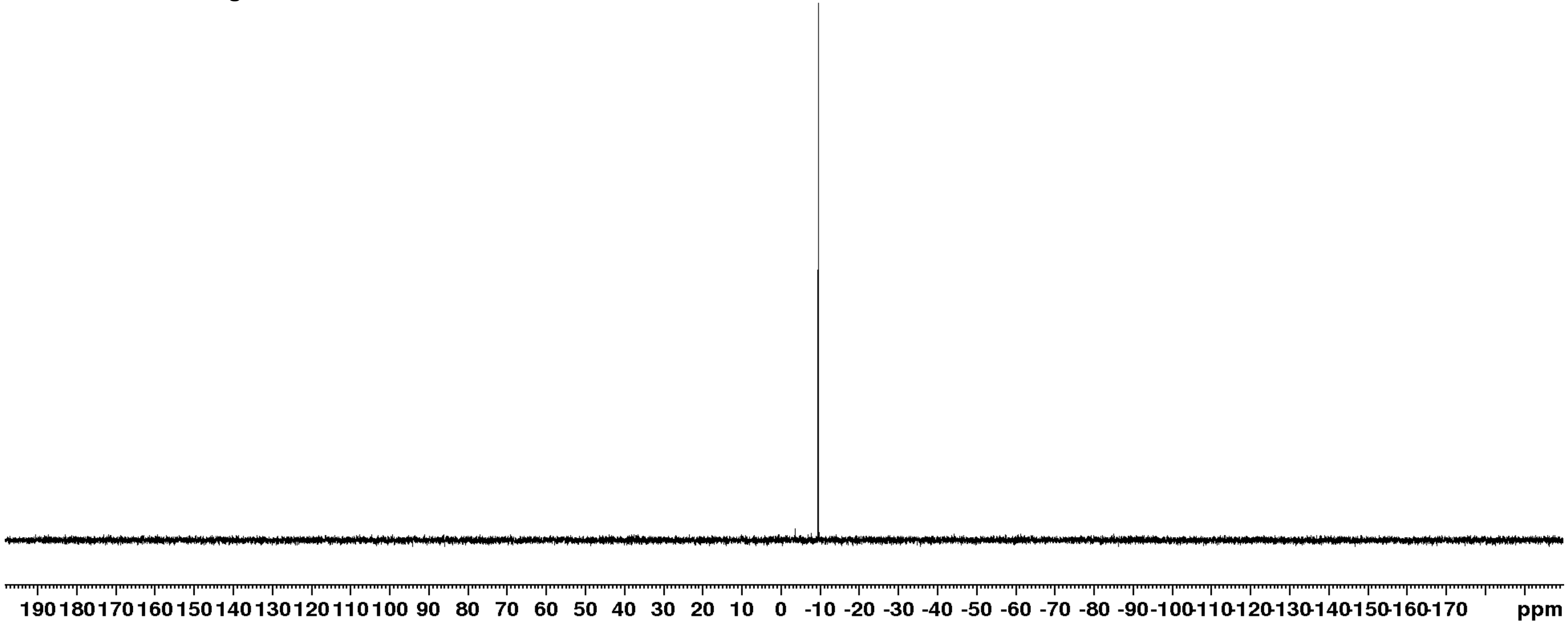

Figure S46. ^{1}H NMR spectrum (400 MHz, $CDCl_3$, 298 K) of **3ah**

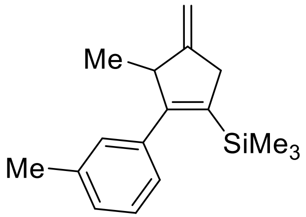

3ah

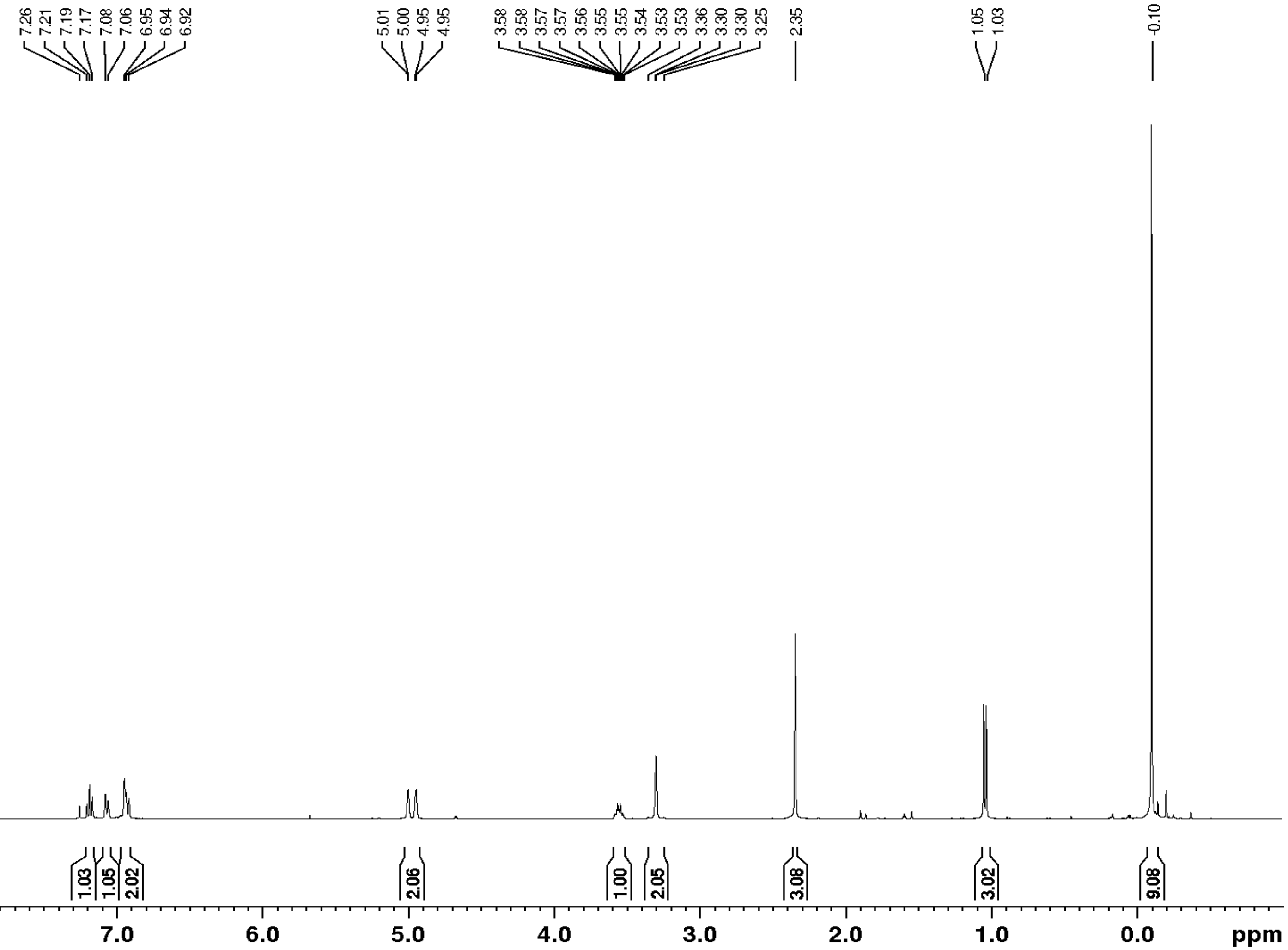

Figure S47. $^{13}C\{^{1}H\}$ NMR spectrum (101 MHz, $CDCl_3$, 298 K) of **3ah**

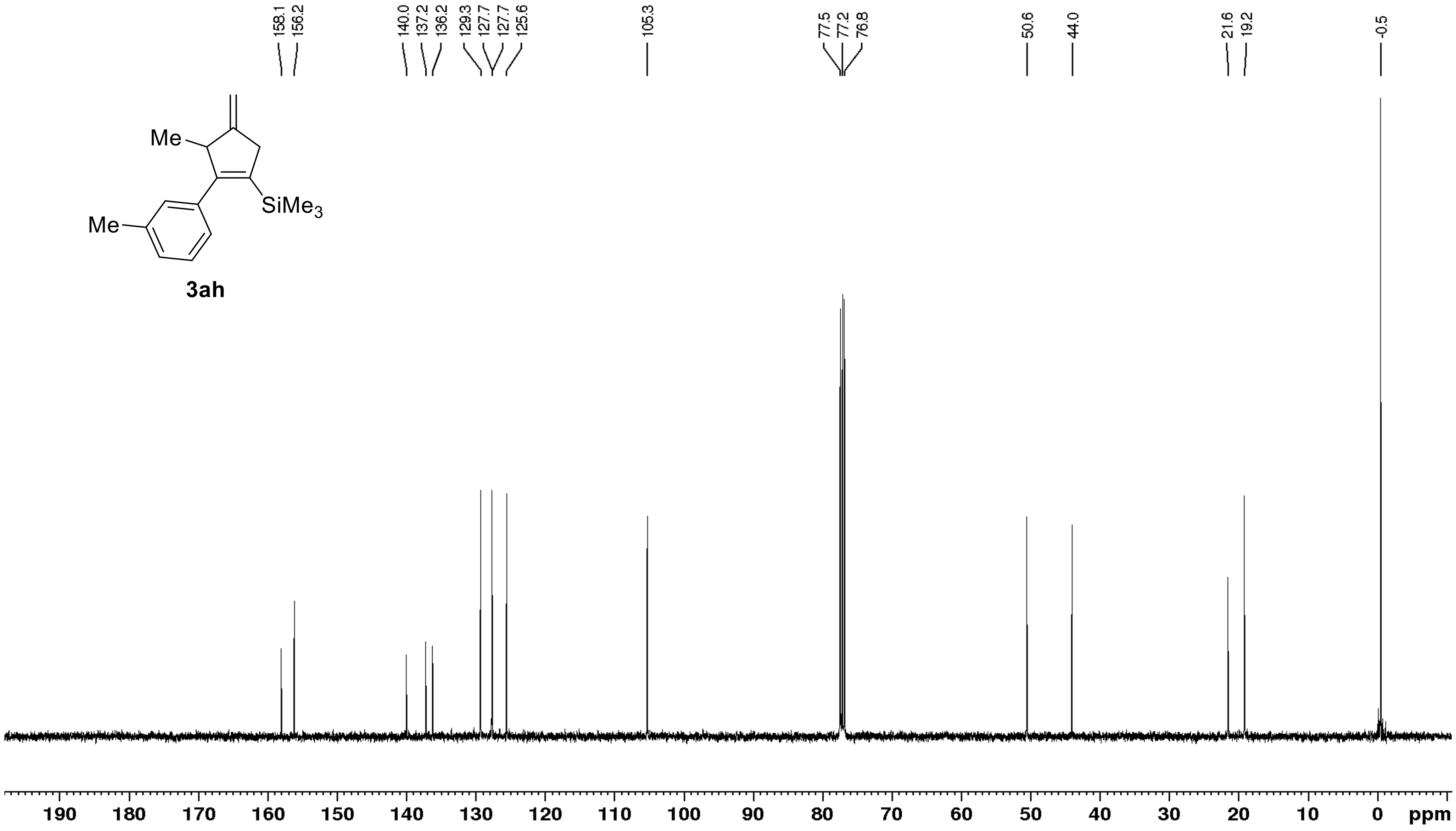

Figure S48. ^{29}Si DEPT NMR spectrum (79 MHz, $CDCl_3$, 298 K, optimized for J = 7.0 Hz) of **3ah**

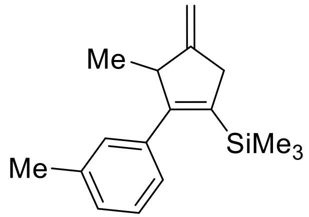

3ah

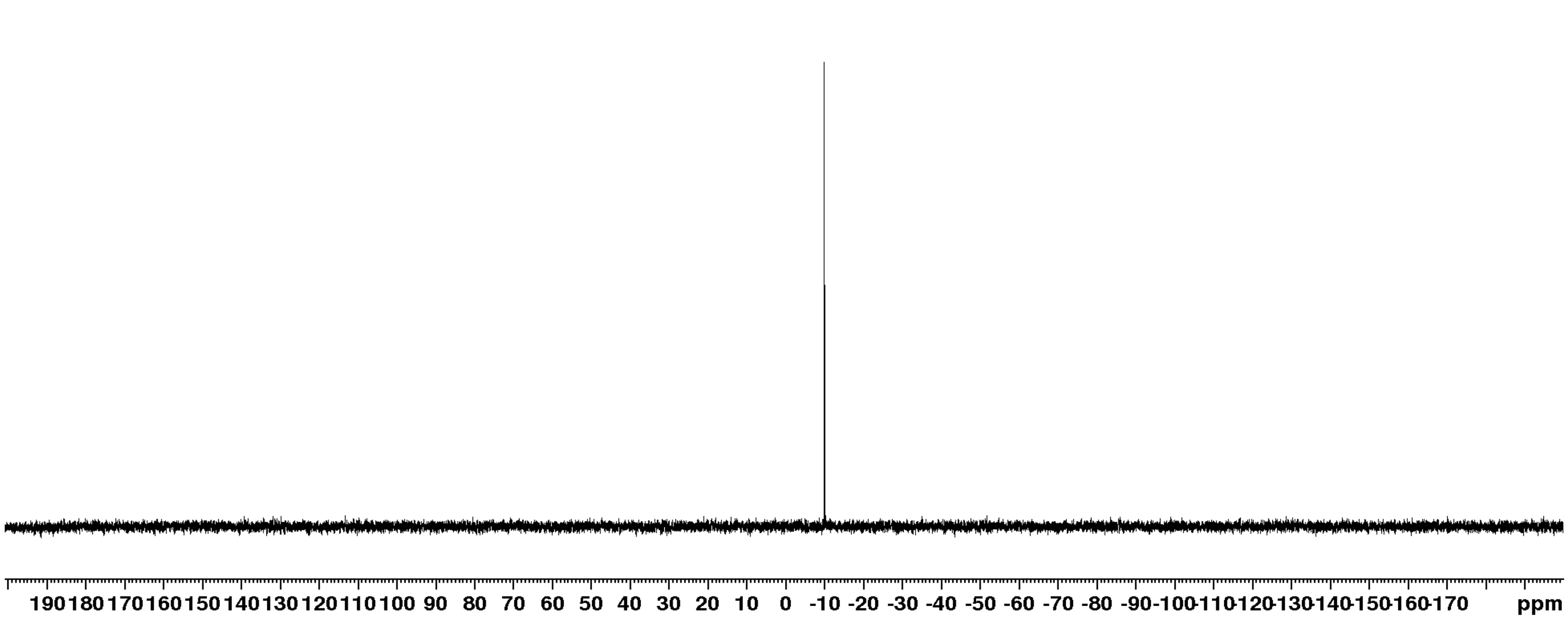

Figure S49. ^{1}H NMR spectrum (500 MHz, $CDCl_3$, 298 K) of **3ai**

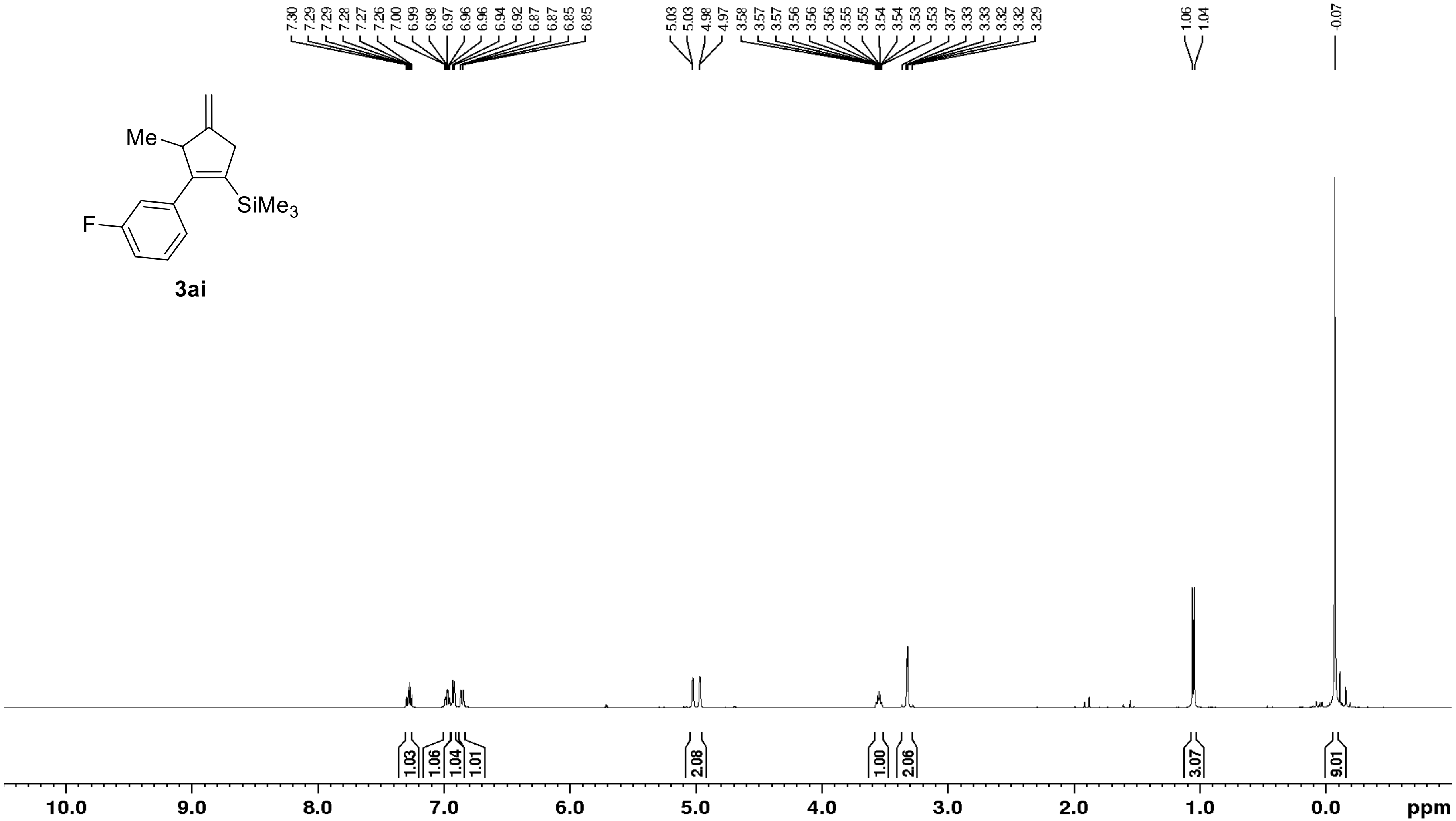

3ai

Figure S50. $^{13}C\{^1H\}$ NMR spectrum (126 MHz, $CDCl_3$, 298 K) of **3ai**

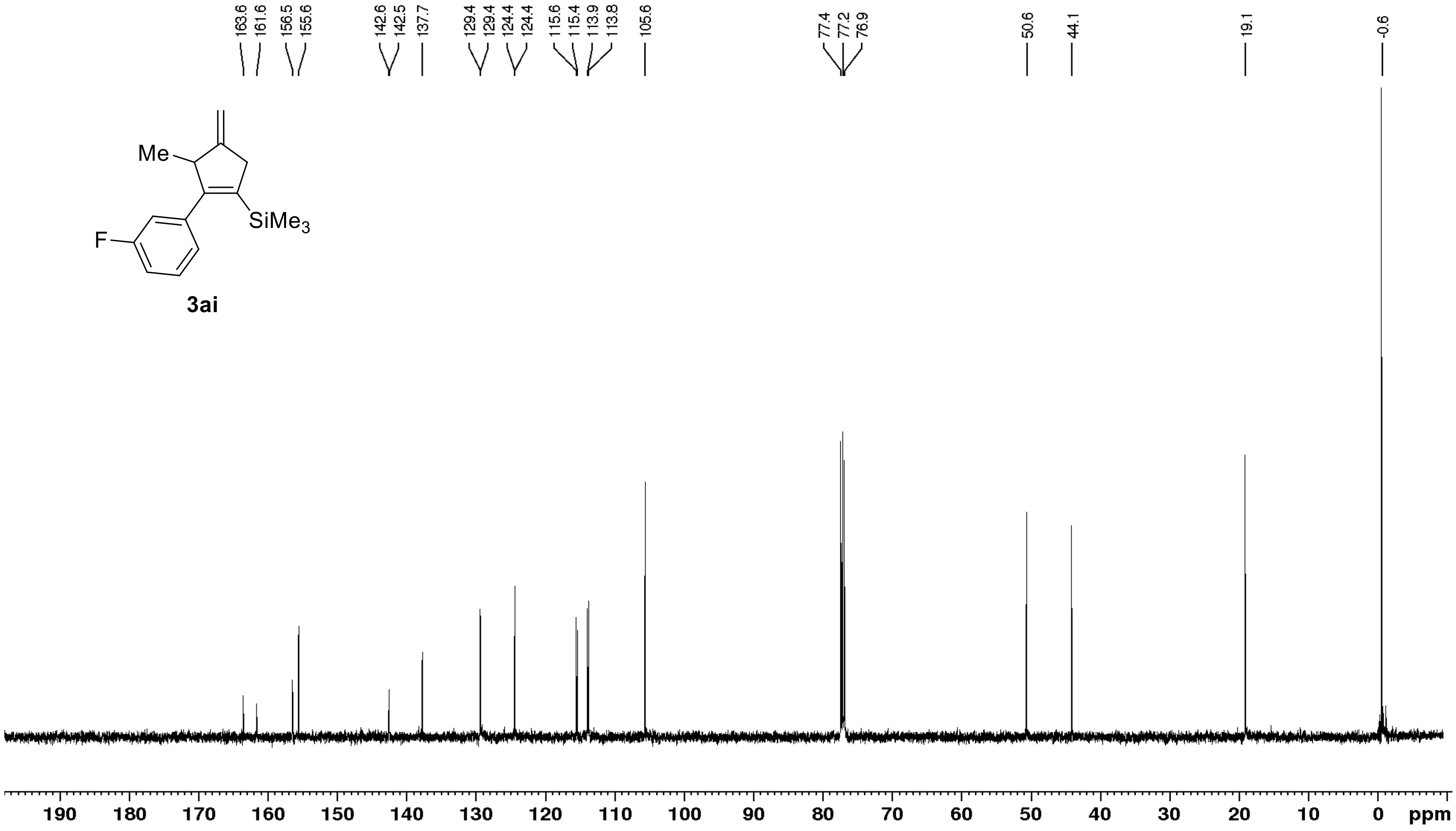

Figure S51. ^{19}F NMR spectrum (471 MHz, $CDCl_3$, 298 K) of **3ai**

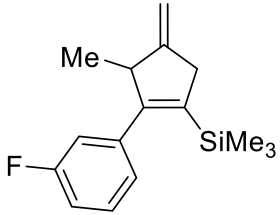

3ai

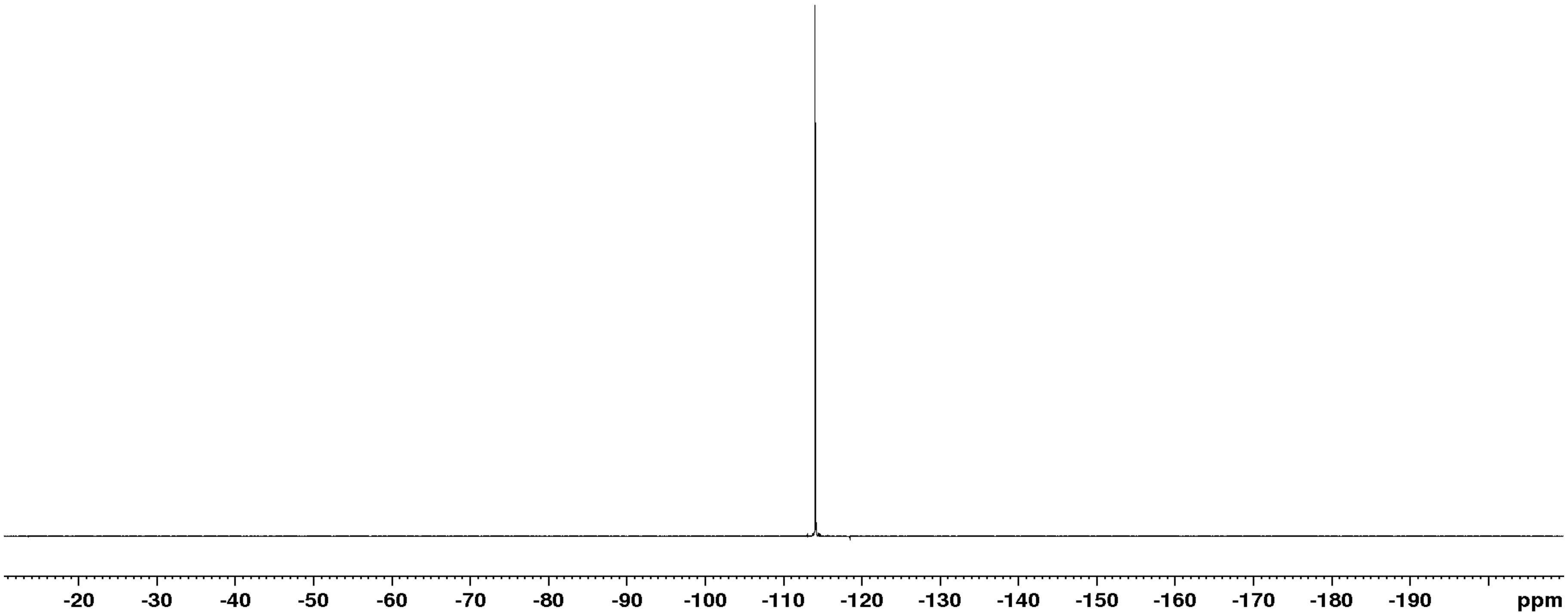

Figure S52. ^{29}Si DEPT NMR spectrum (79 MHz, $CDCl_3$, 298 K, optimized for *J* = 7.0 Hz) of **3ai**

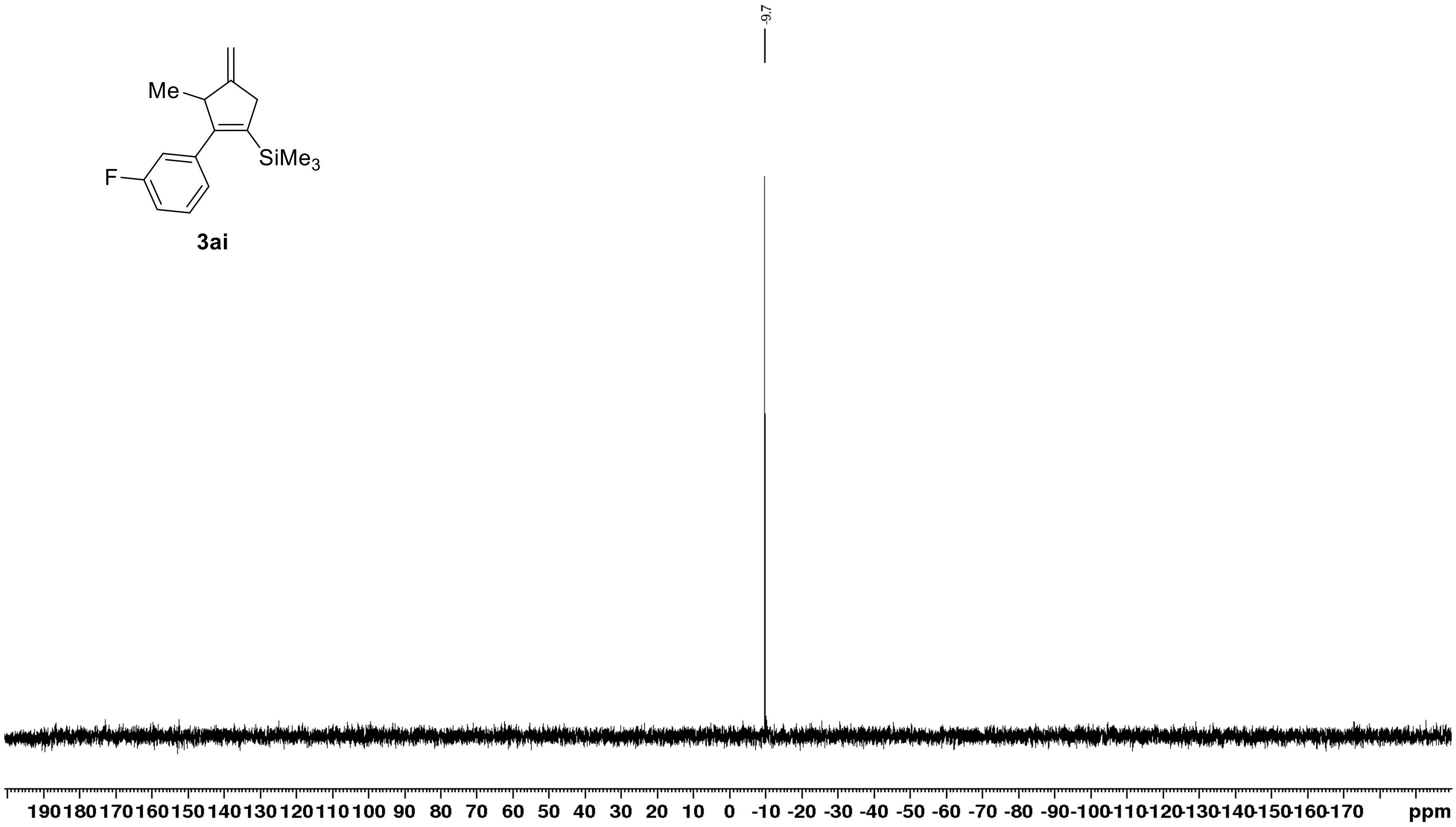

Figure S53. ^{1}H NMR spectrum (500 MHz, $CDCl_3$, 298 K) of **3aj**

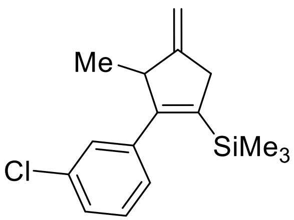

3aj

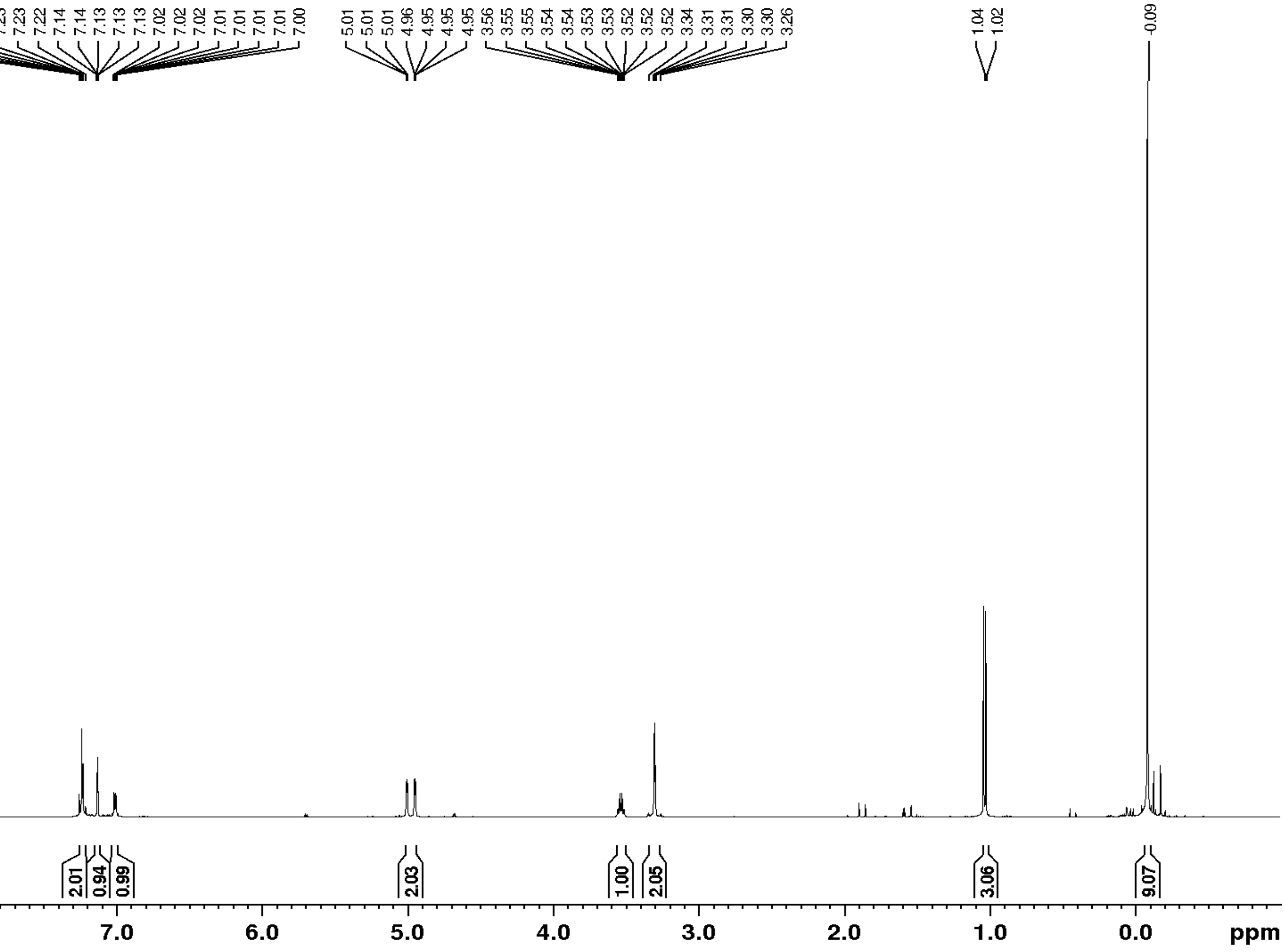

Figure S54. $^{13}C\{^{1}H\}$ NMR spectrum (126 MHz, $CDCl_3$, 298 K) of **3aj**

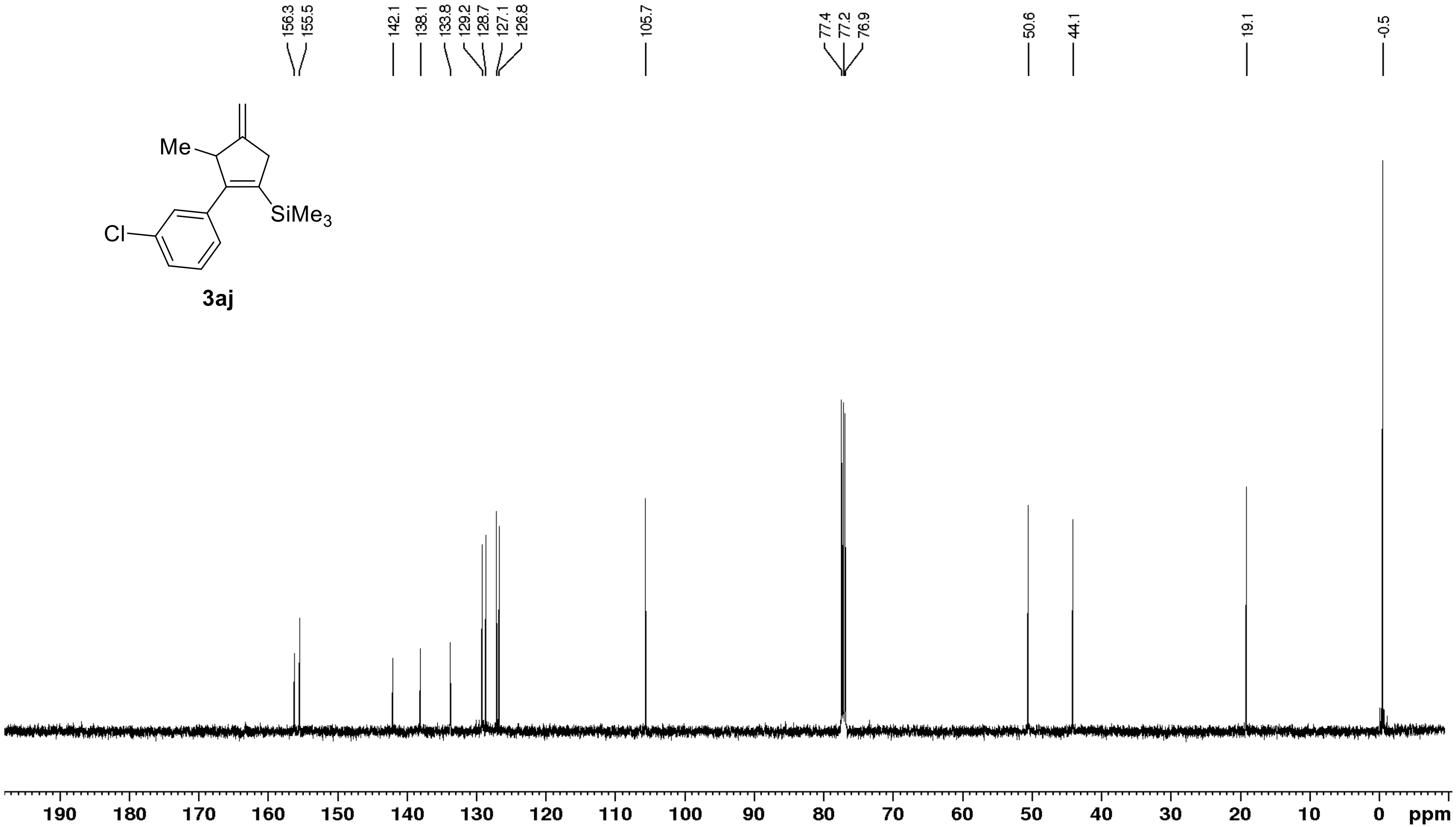

Figure S55. ^{29}Si DEPT NMR spectrum (99 MHz, $CDCl_3$, 298 K, optimized for J = 7.0 Hz) of **3aj**

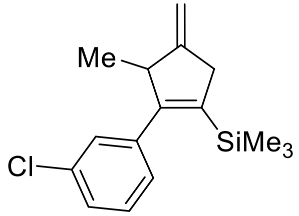

3aj

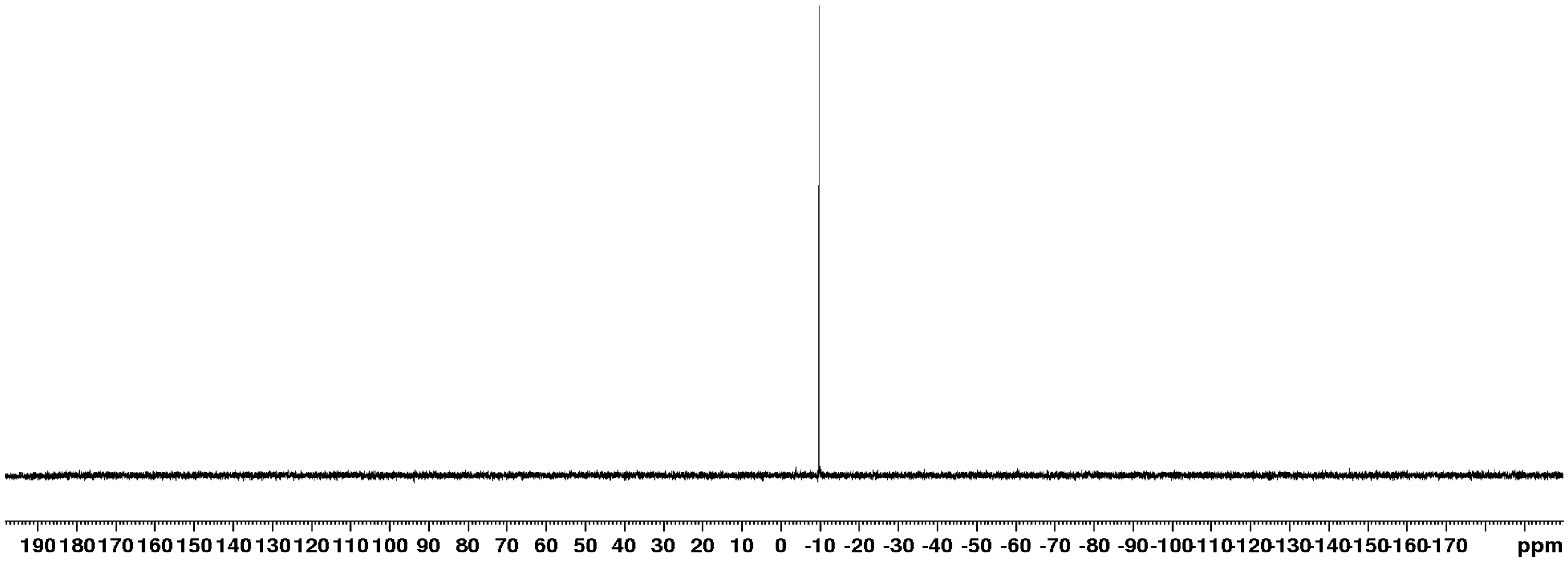

Figure S56. ^1H NMR spectrum (500 MHz, $CDCl_3$, 298 K) of **3ak**

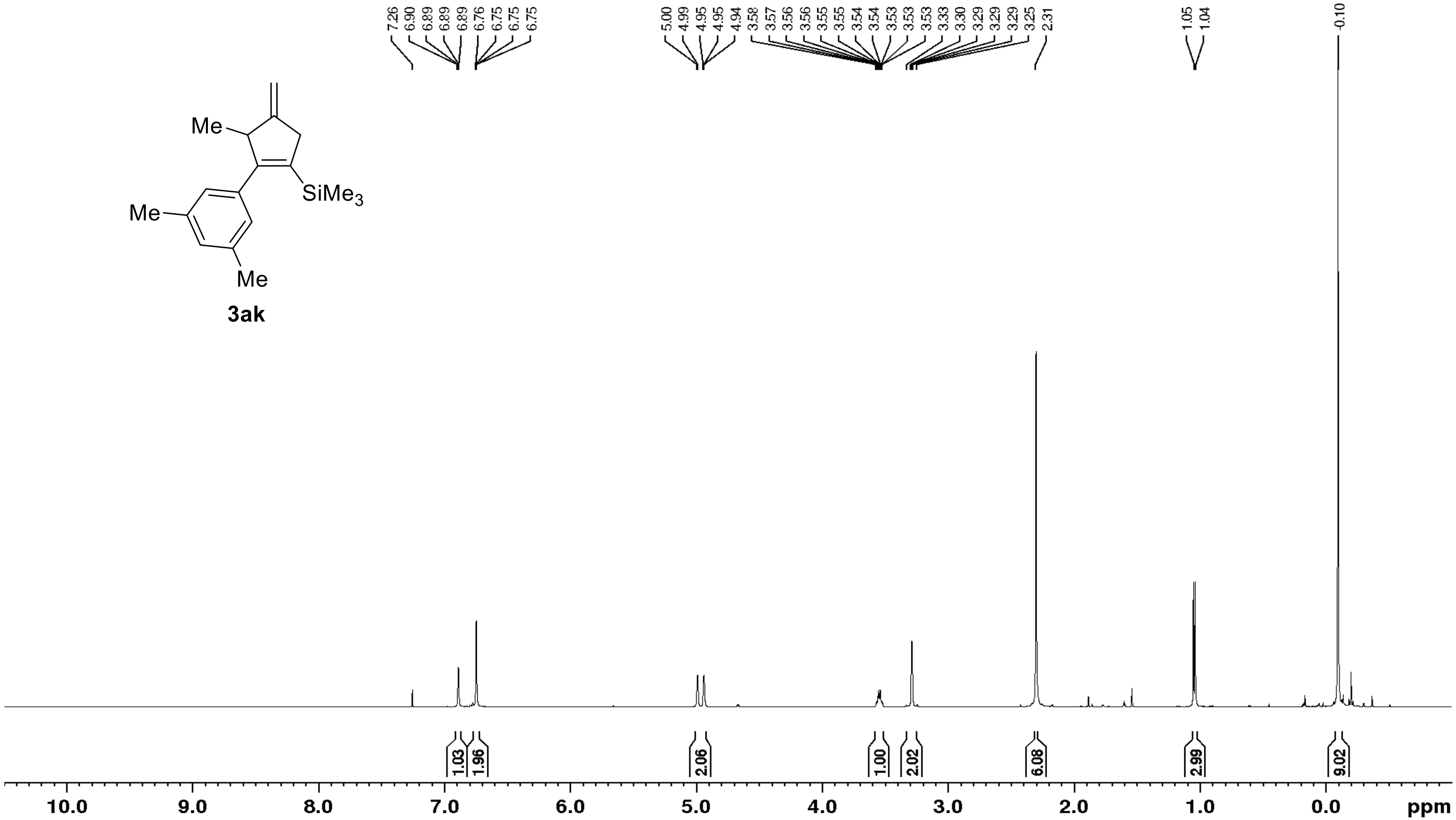

Figure S57. $^{13}C\{^{1}H\}$ NMR spectrum (101 MHz, $CDCl_3$, 298 K) of **3ak**

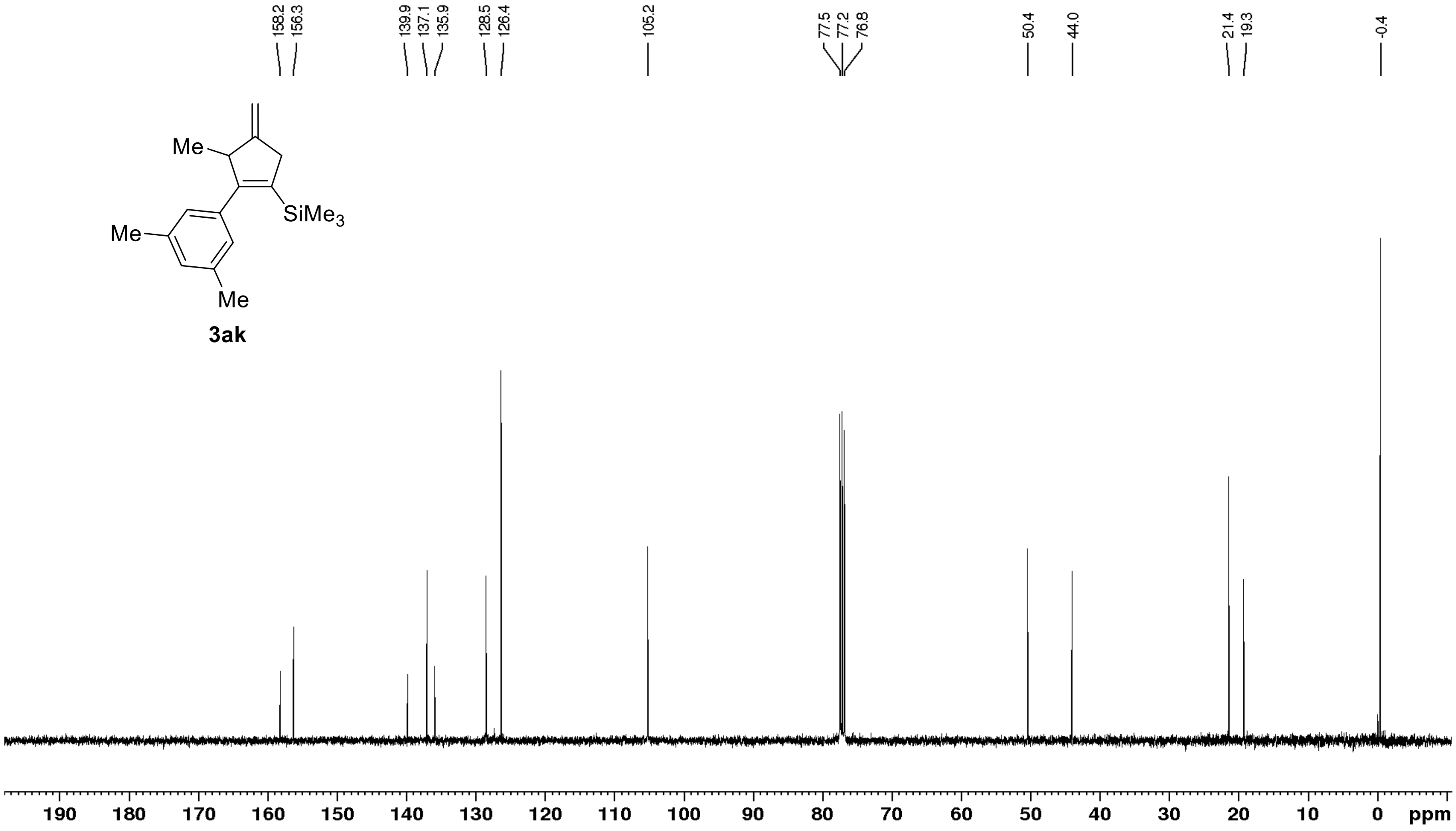

Figure S58. ^{29}Si DEPT NMR spectrum (79 MHz, $CDCl_3$, 298 K, optimized for J = 7.0 Hz) of **3ak**

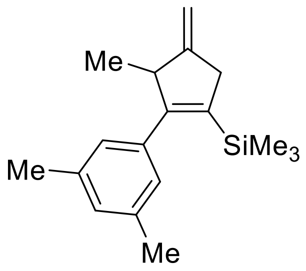

3ak

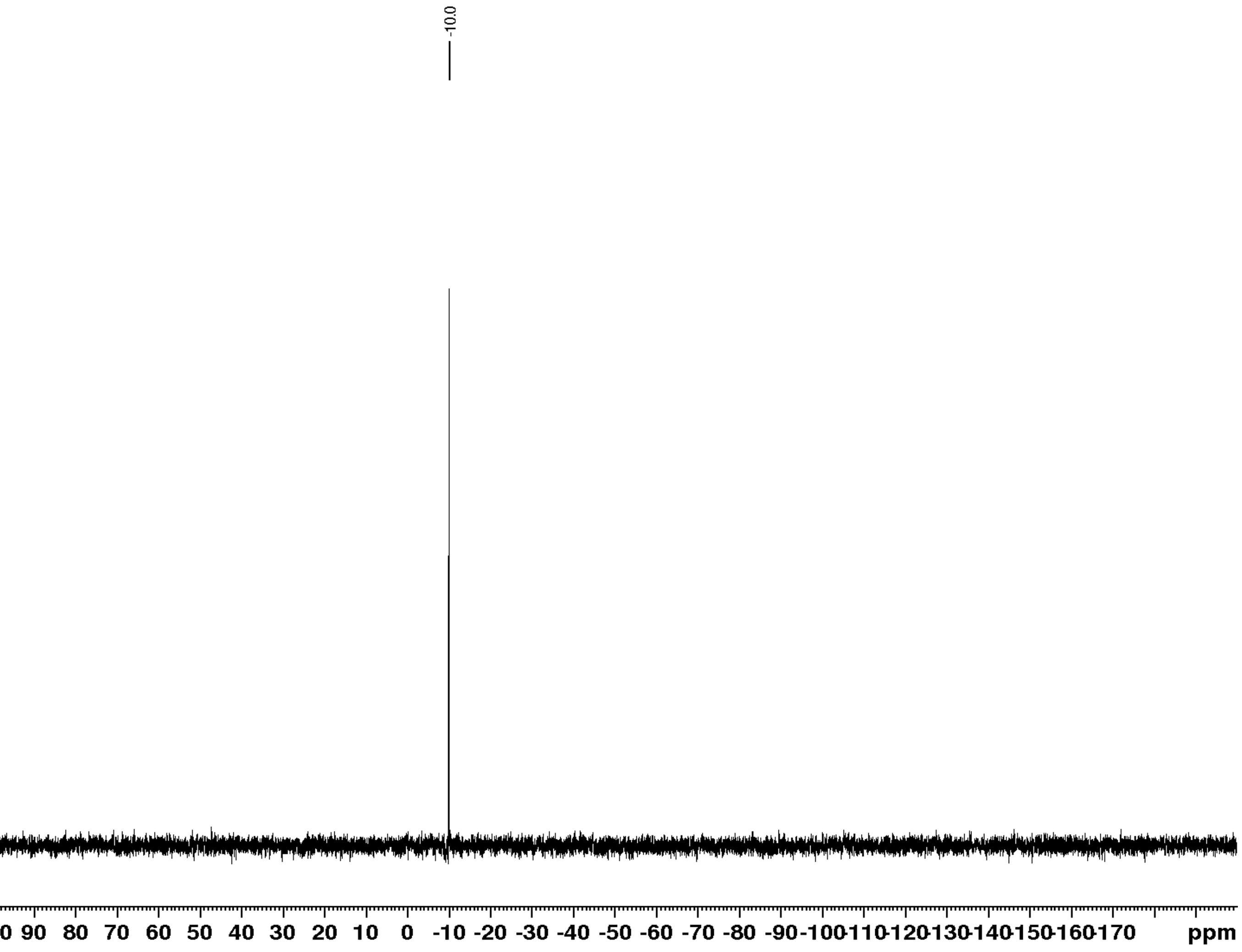

Figure S59. ^{1}H NMR spectrum (400 MHz, $CDCl_3$, 298 K) of **3al**

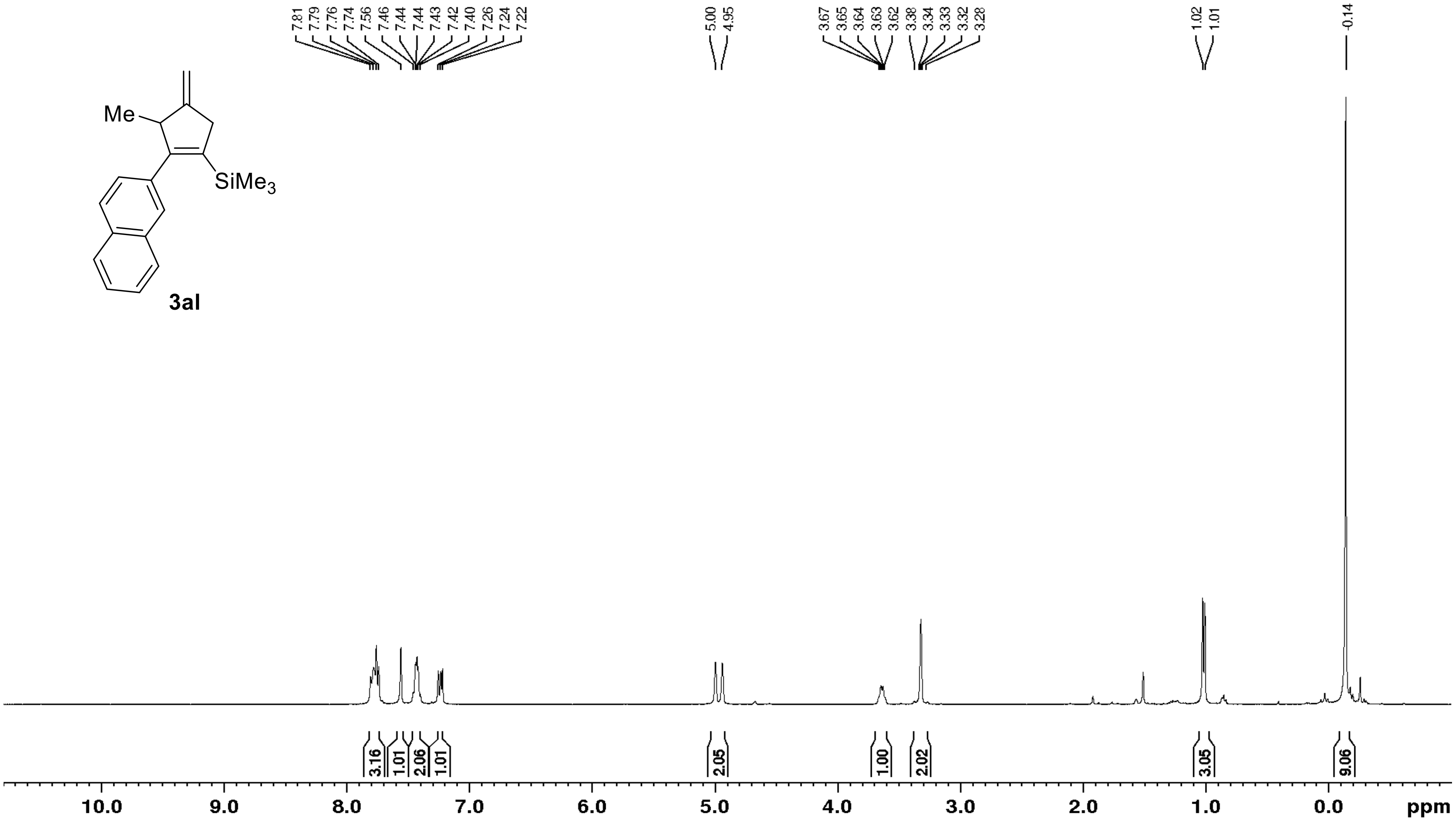

Figure S60. $^{13}C\{^{1}H\}$ NMR spectrum (101 MHz, $CDCl_3$, 298 K) of **3al**

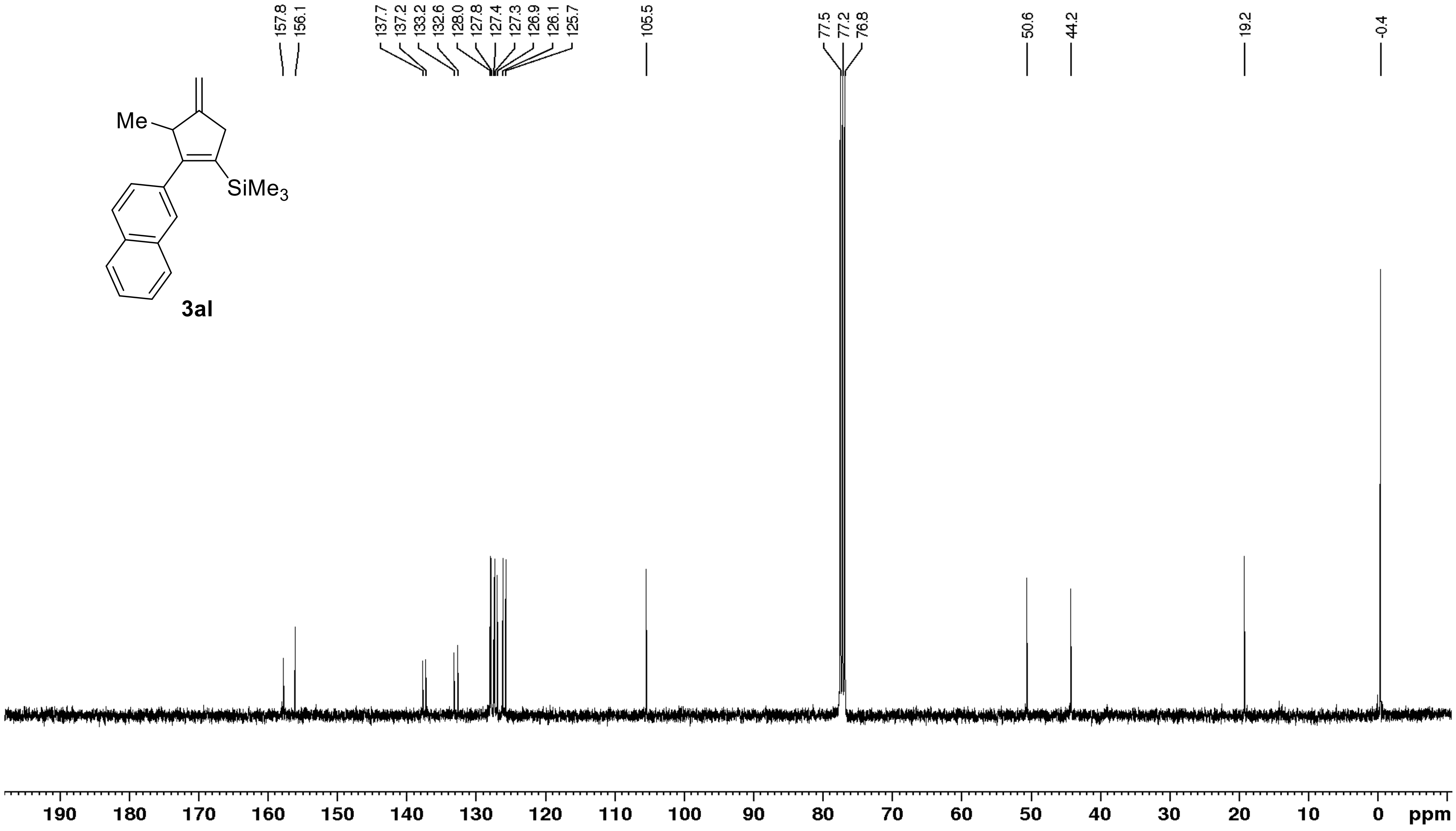

Figure S61. ^{29}Si DEPT NMR spectrum (79 MHz, $CDCl_3$, 298 K, optimized for J = 7.0 Hz) of **3al**

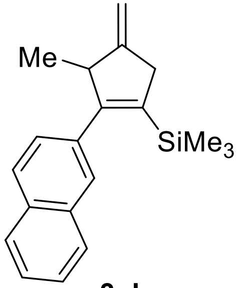

3al

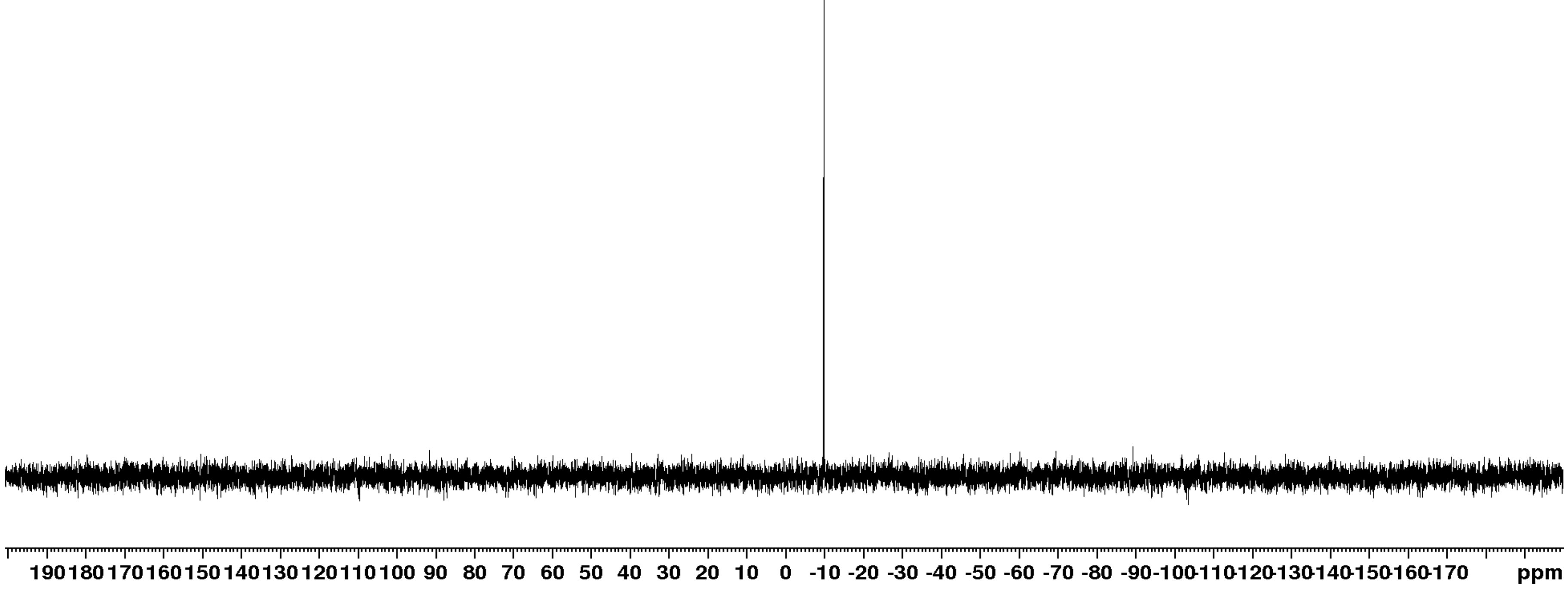

Figure S62. ^{1}H NMR spectrum (500 MHz, $CDCl_3$, 298 K) of **3am**

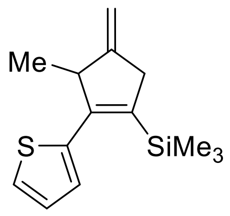

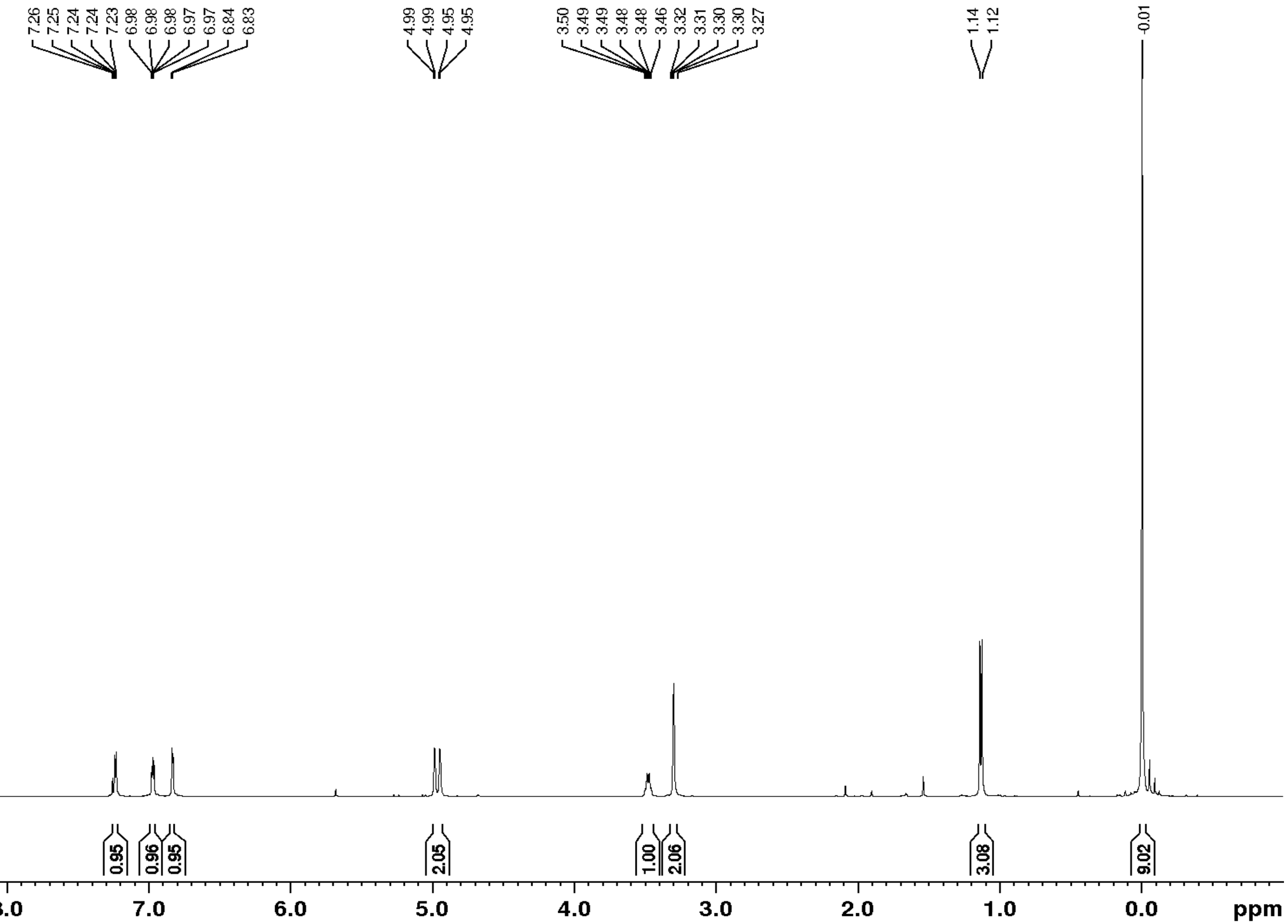

Figure S63. $^{13}C\{^{1}H\}$ NMR spectrum (101 MHz, $CDCl_3$, 298 K) of **3am**

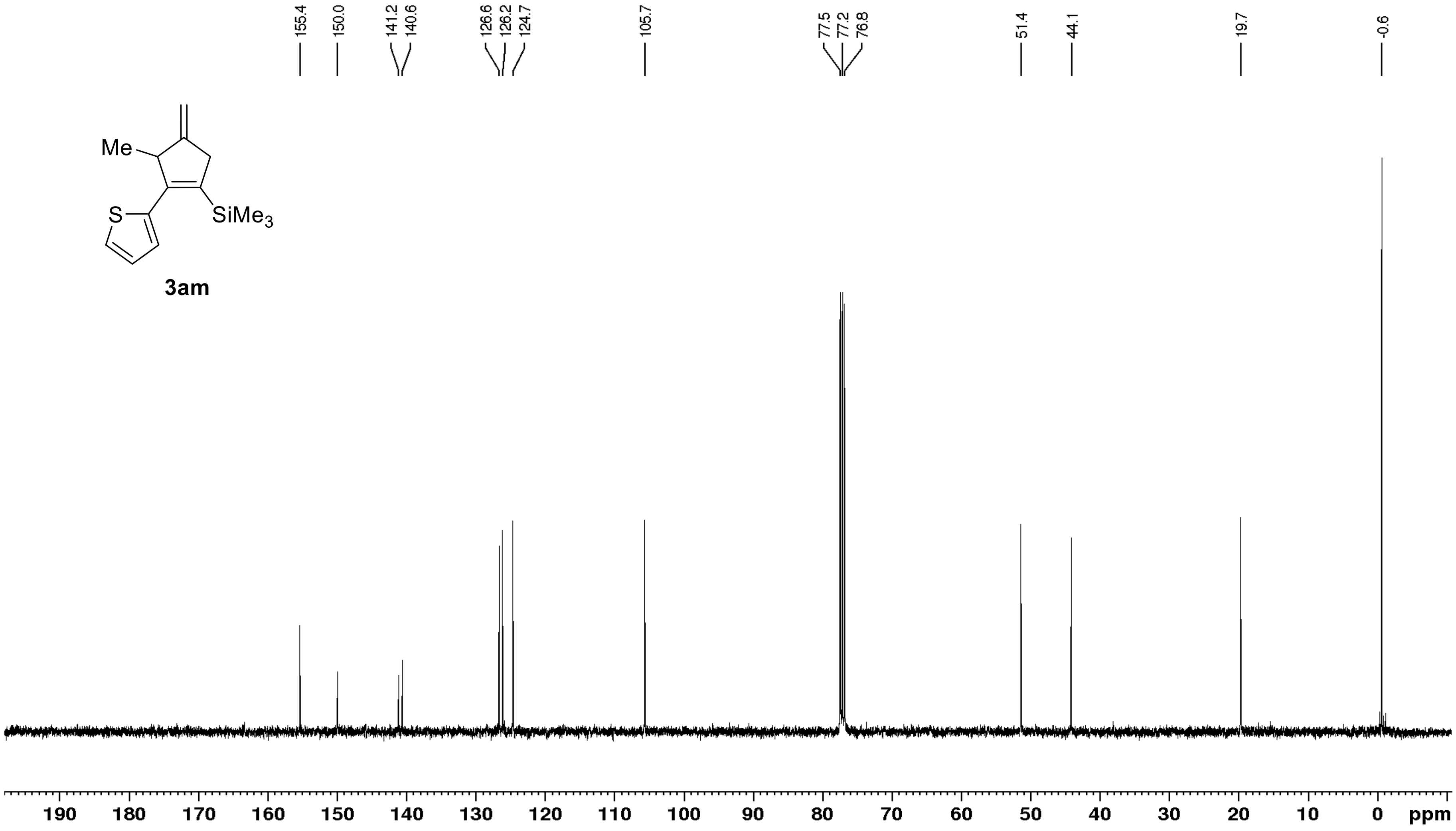

Figure S64. ^{29}Si DEPT NMR spectrum (79 MHz, $CDCl_3$, 298 K, optimized for J = 7.0 Hz) of **3am**

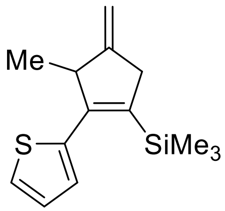

3am

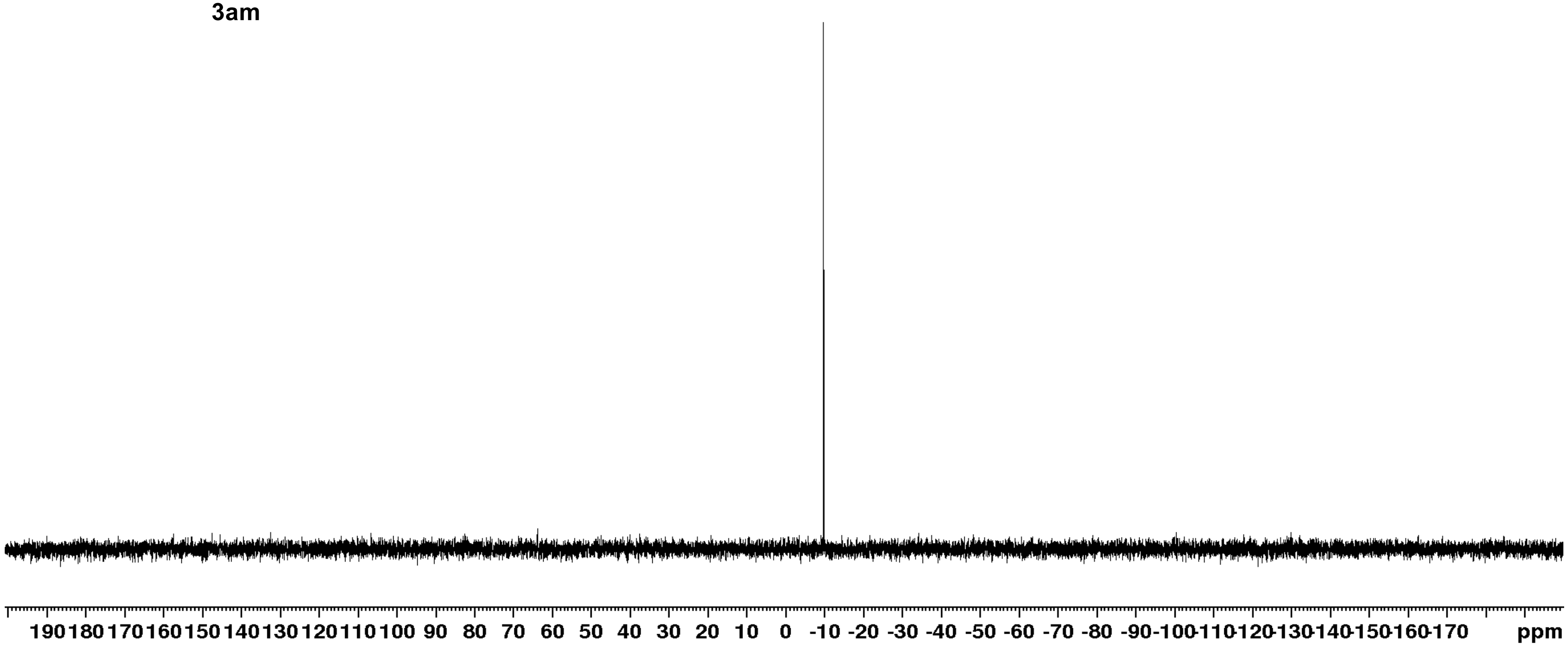

Figure S65. ^{1}H NMR spectrum (500 MHz, $CDCl_3$, 298 K) of **3an**

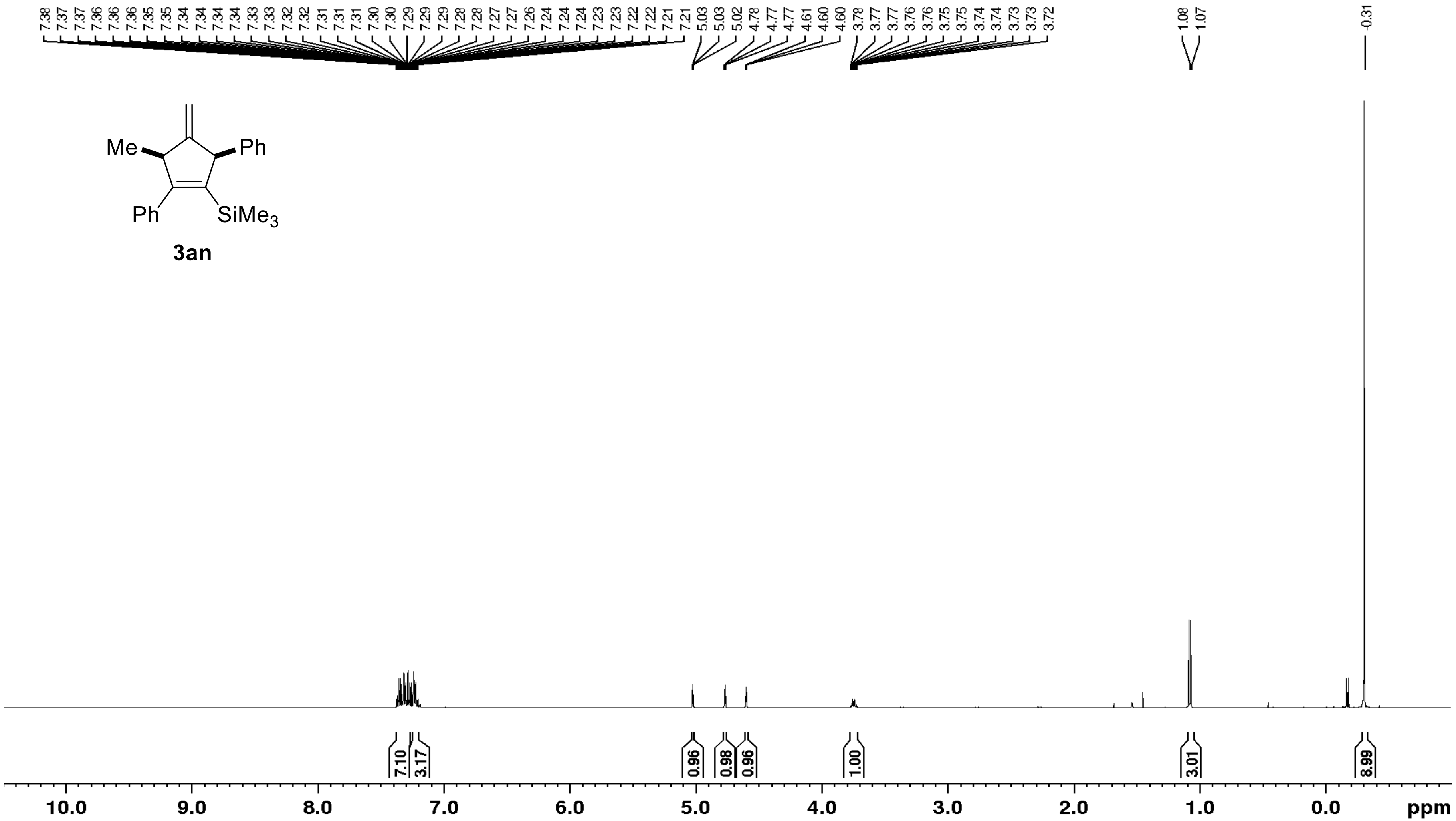

Figure S66. $^{13}C\{^1H\}$ NMR spectrum (101 MHz, $CDCl_3$, 298 K) of **3an**

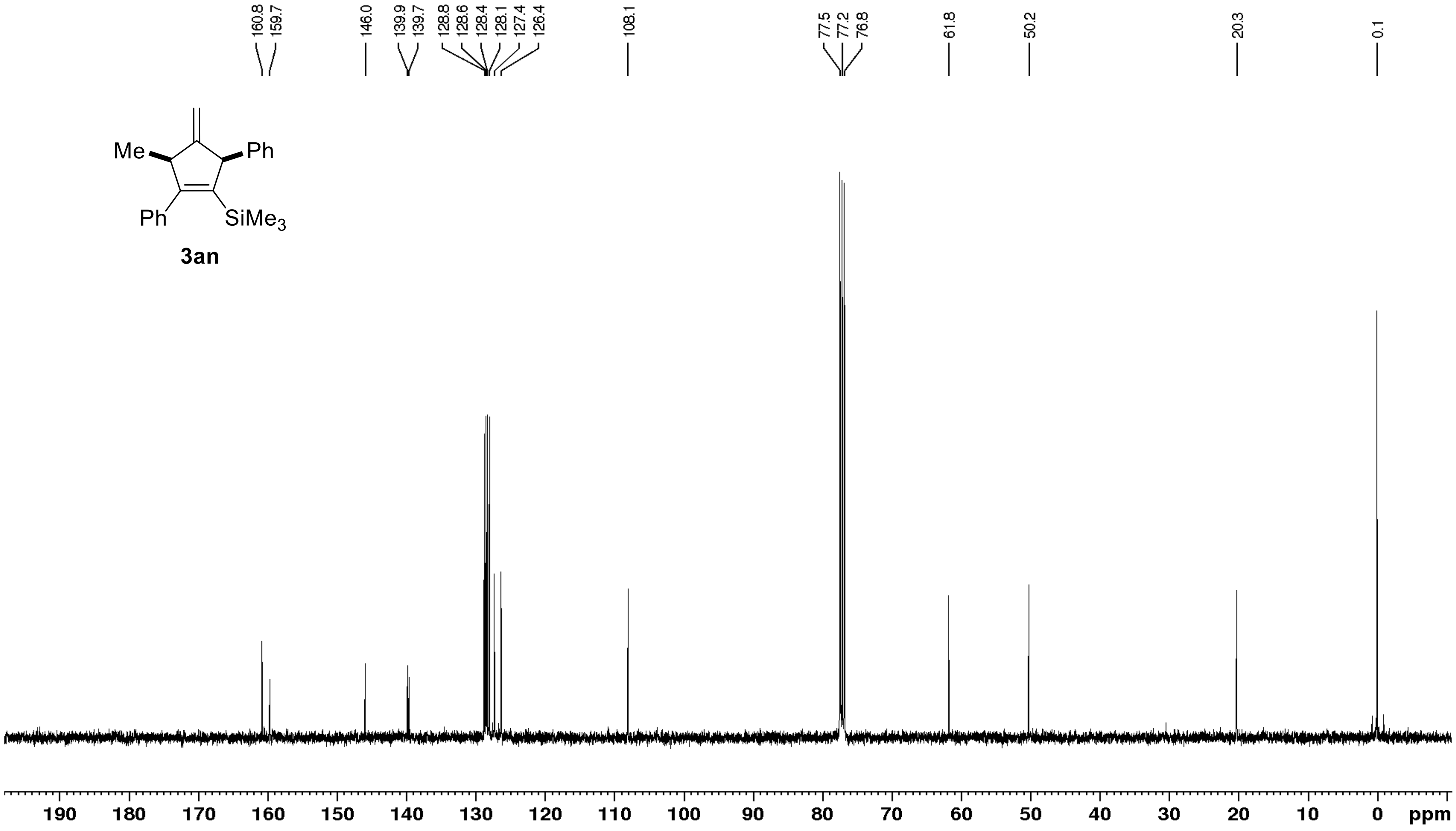

Figure S67. ^{29}Si DEPT NMR spectrum (79 MHz, $CDCl_3$, 298 K, optimized for J = 7.0 Hz) of **3an**

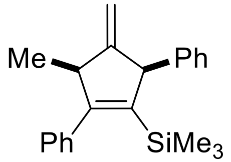

3an

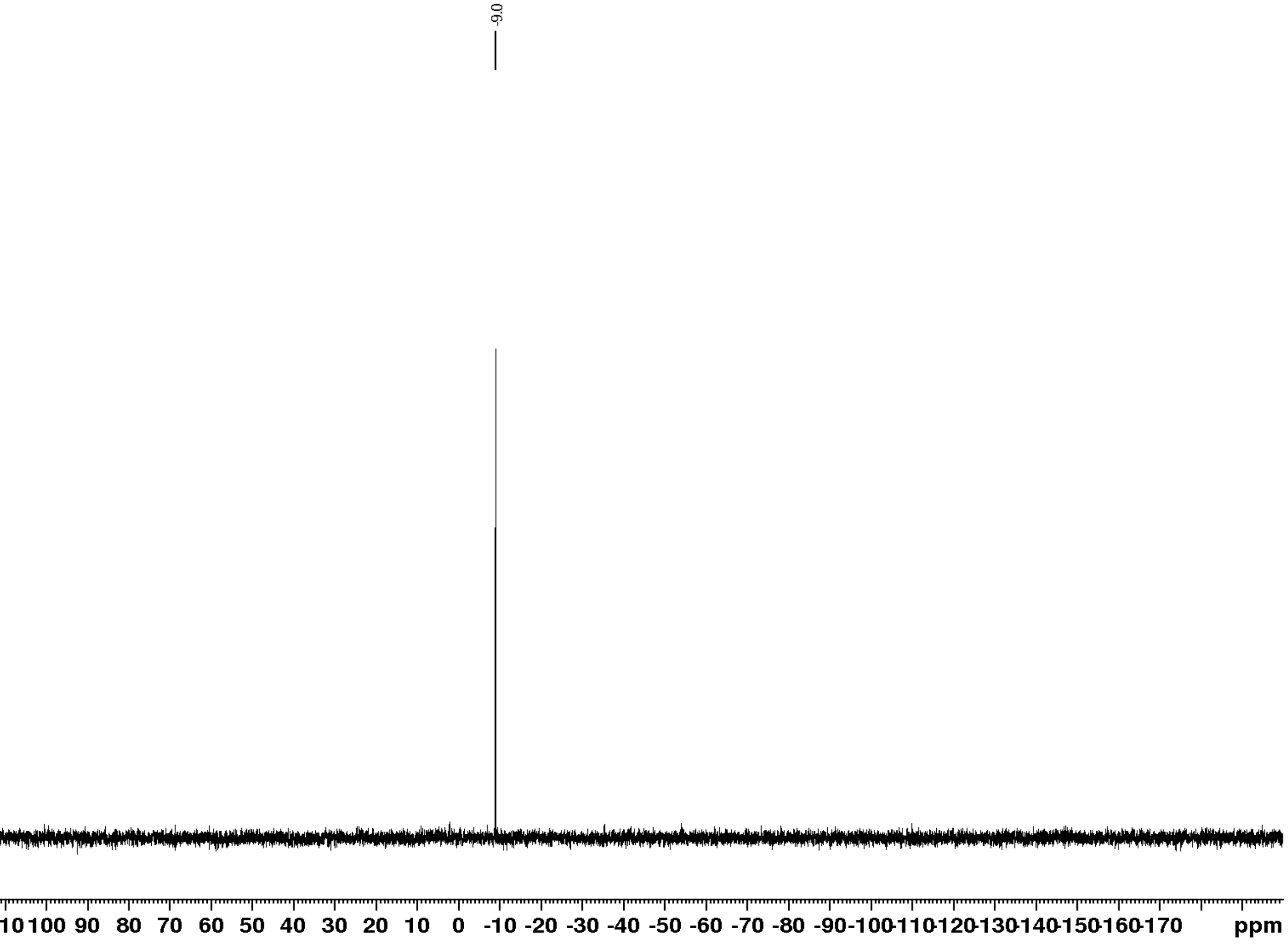

Figure S68. ^{1}H NMR spectrum (500 MHz, $CDCl_3$, 298 K) of **3ao** (*minor *trans*-diastereomer)

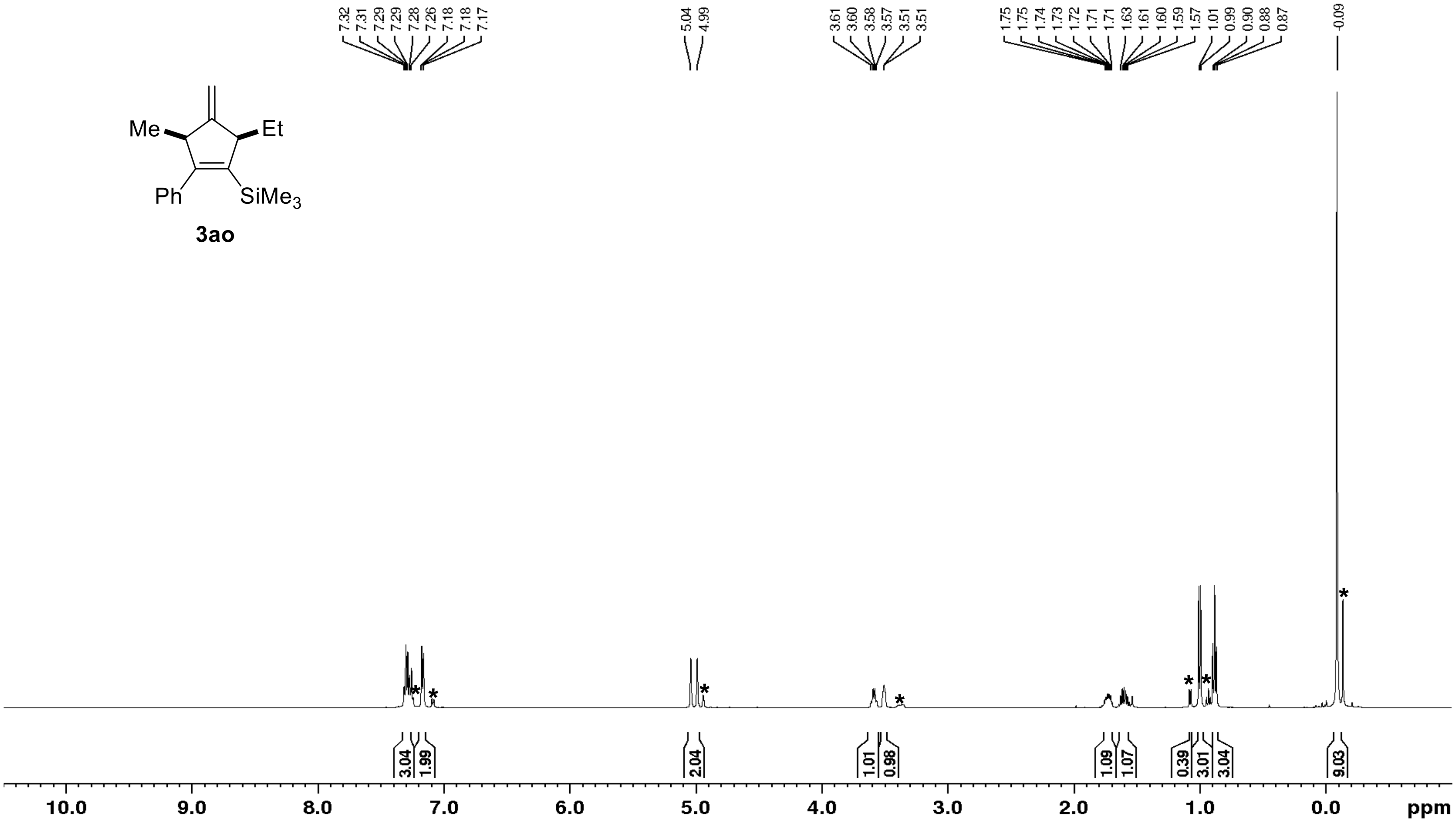

Figure S69. $^{13}C\{^{1}H\}$ NMR spectrum (126 MHz, $CDCl_3$, 298 K) of **3ao**

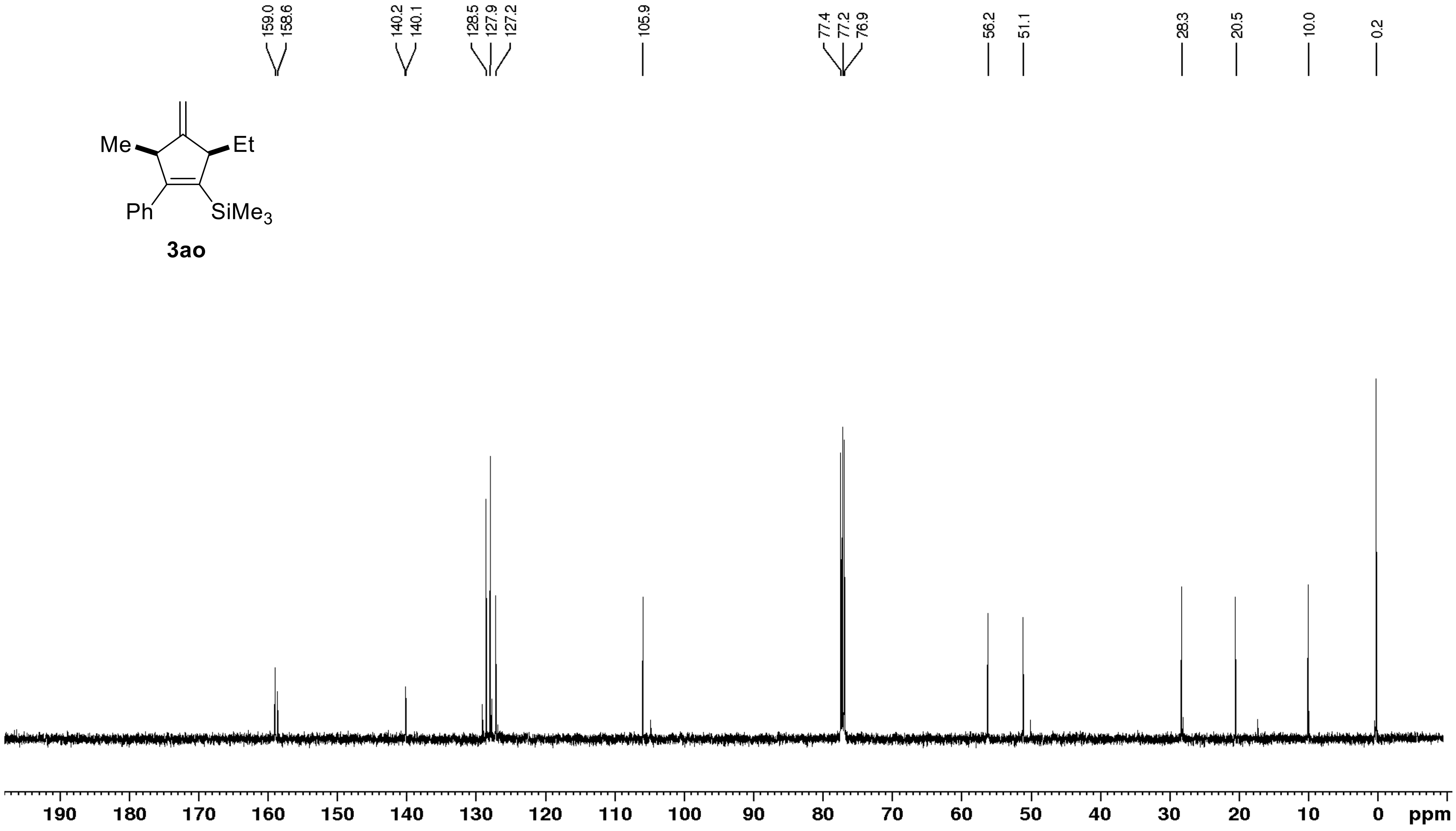

Figure S70. ^{29}Si DEPT NMR spectrum (99 MHz, $CDCl_3$, 298 K, optimized for J = 7.0 Hz) of **3ao**

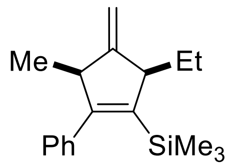

3ao

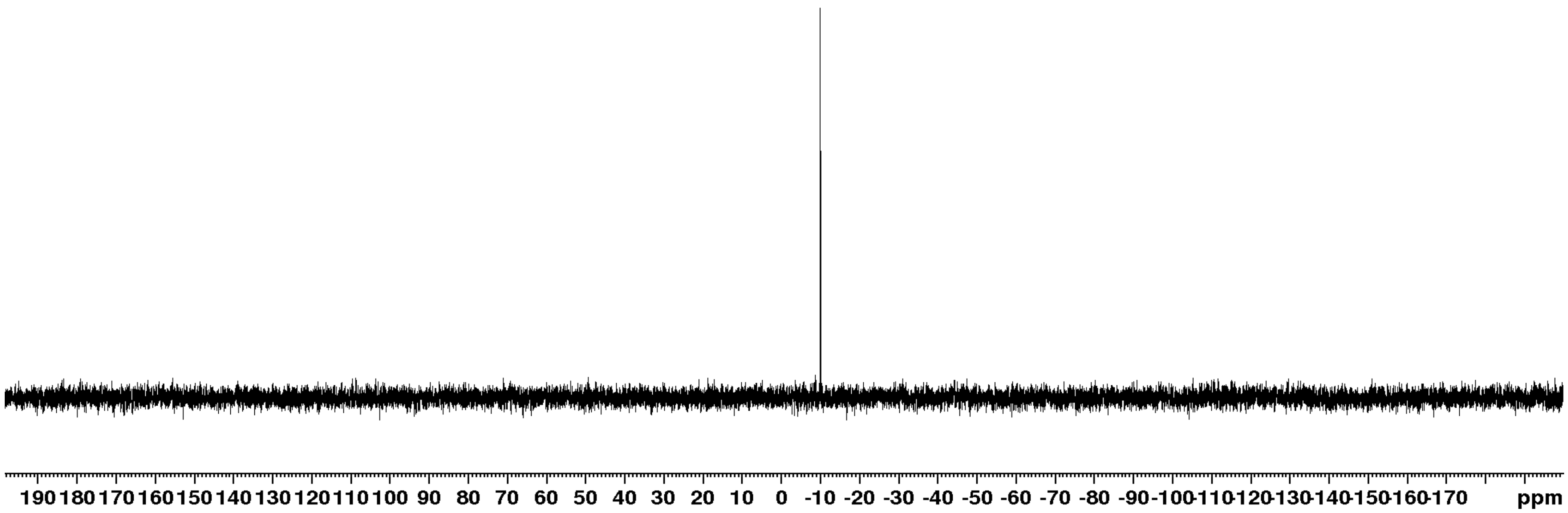

Figure S71. ^{1}H NMR spectrum (400 MHz, $CDCl_3$, 298 K) of **3ba**

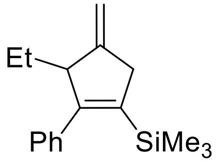

3ba

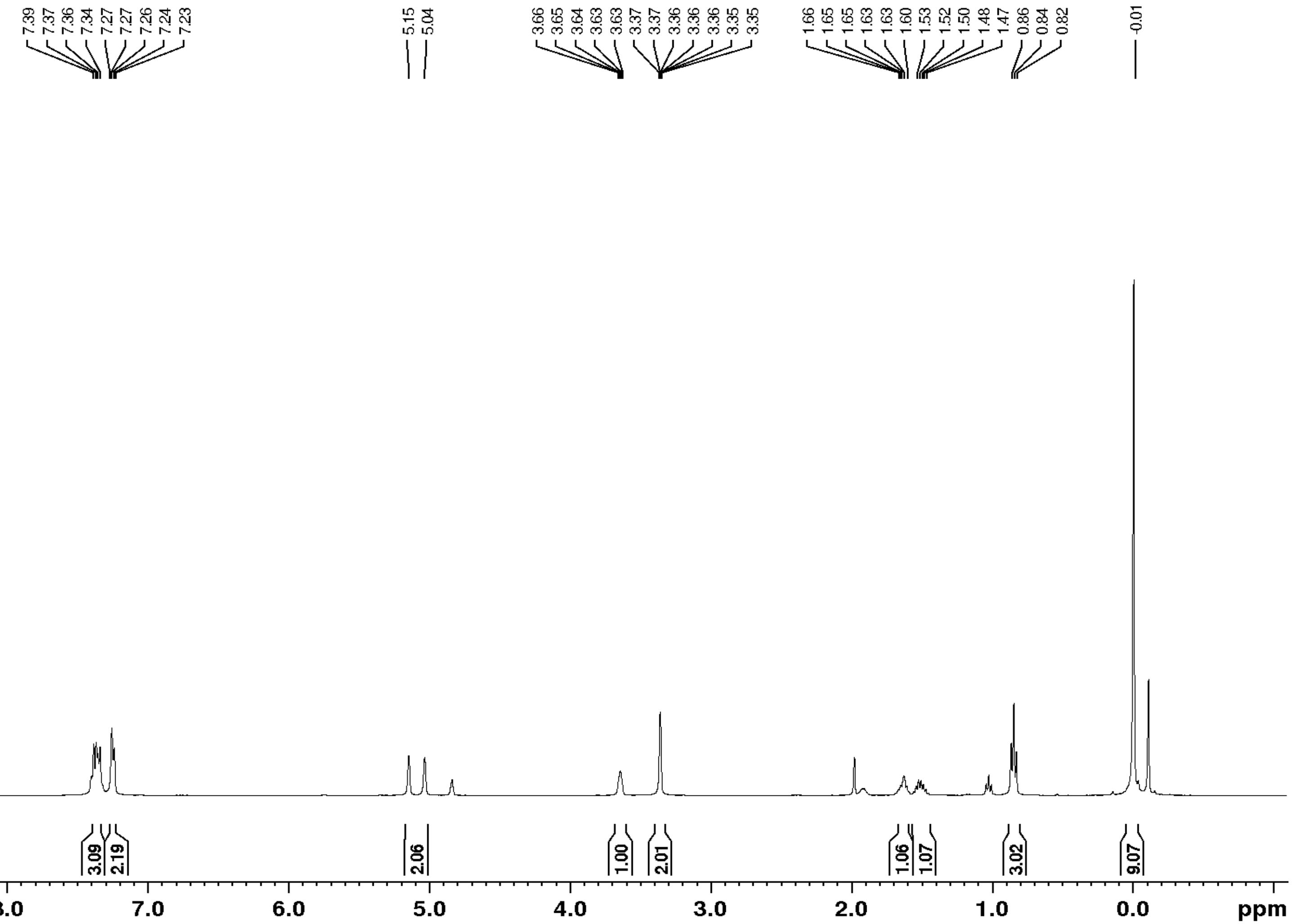

Figure S72. $^{13}C\{^1H\}$ NMR spectrum (101 MHz, $CDCl_3$, 298 K) of **3ba**

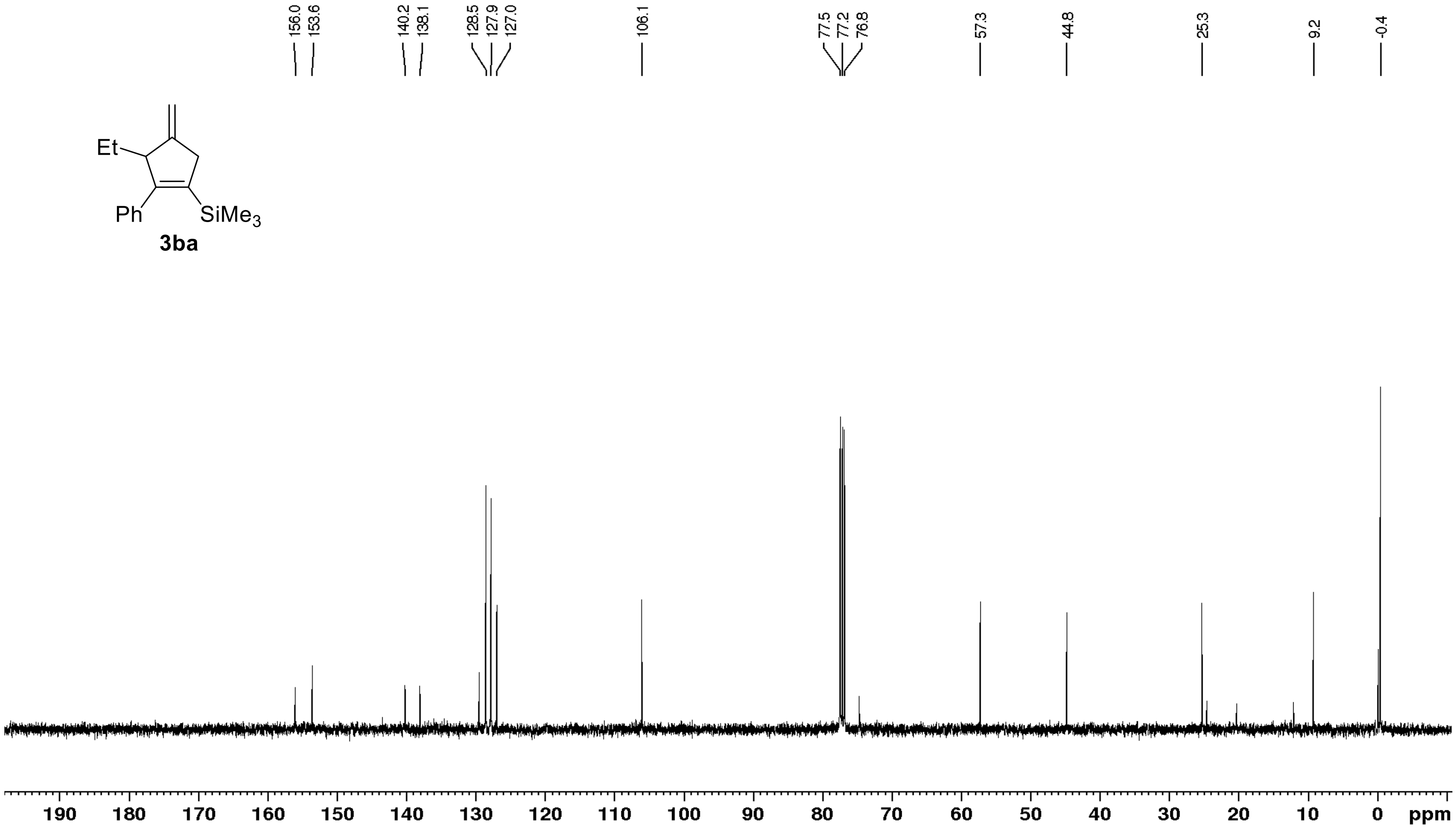

Figure S73. ^{29}Si DEPT NMR spectrum (79 MHz, $CDCl_3$, 298 K, optimized for J = 7.0 Hz) of **3ba**

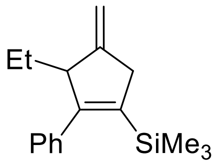

3ba

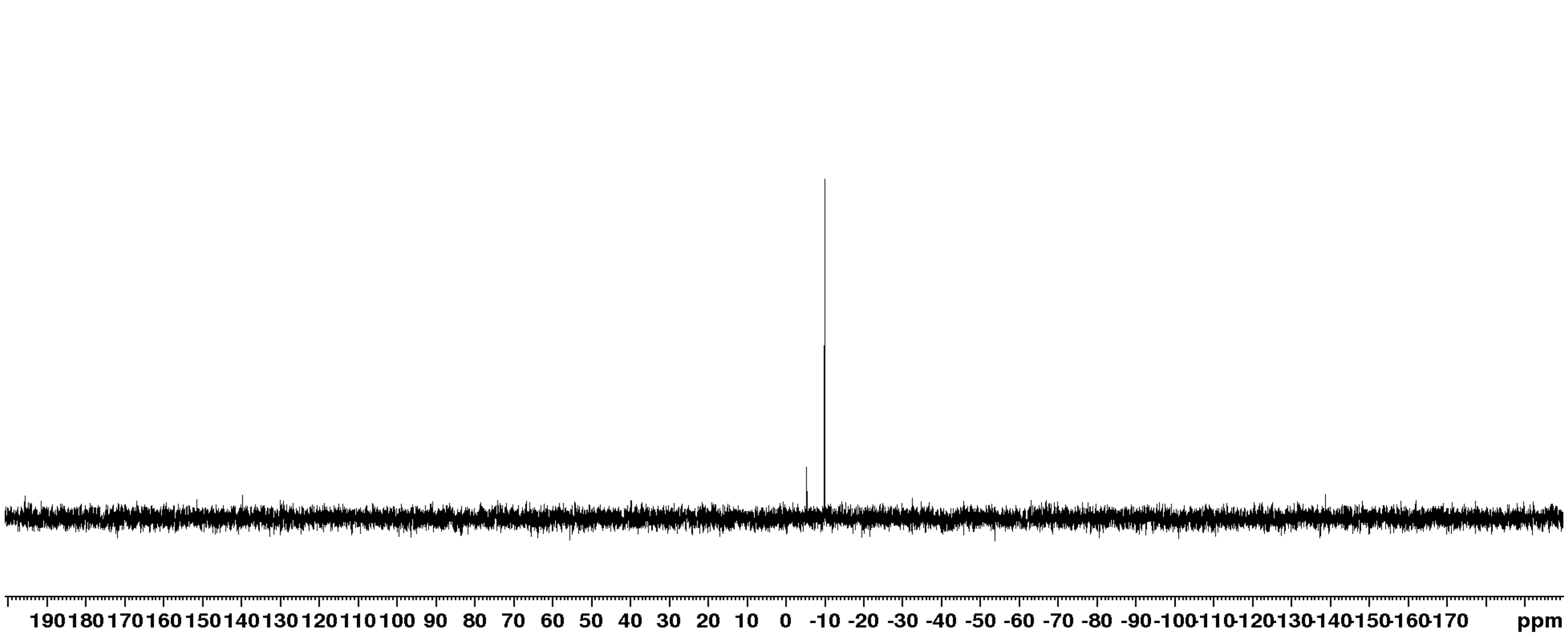

Figure S74. ¹H NMR spectrum (500 MHz, $CDCl_3$, 298 K) of **3ca**

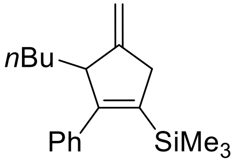

3ca

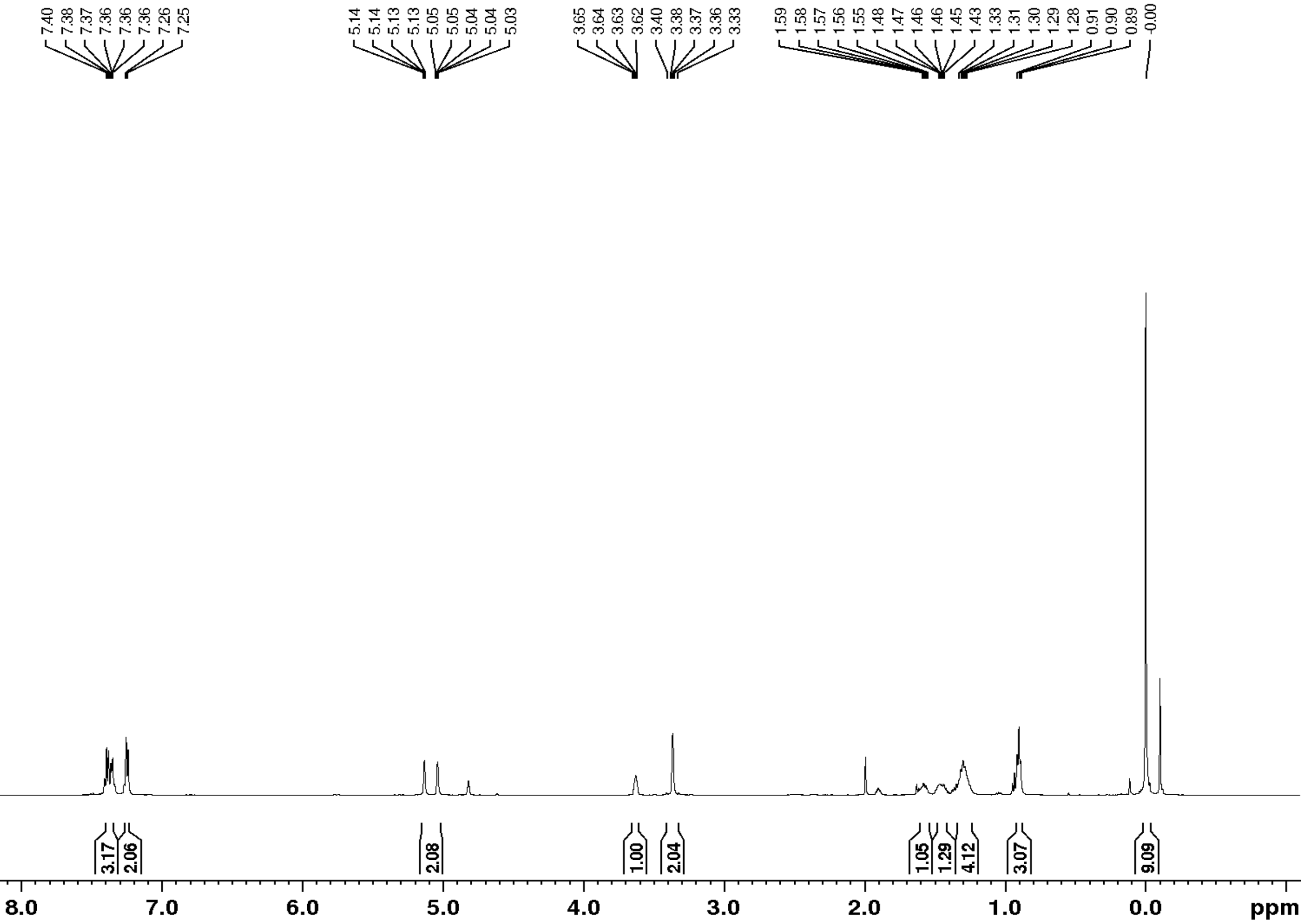

Figure S75. $^{13}C\{^{1}H\}$ NMR spectrum (101 MHz, $CDCl_3$, 298 K) of **3ca**

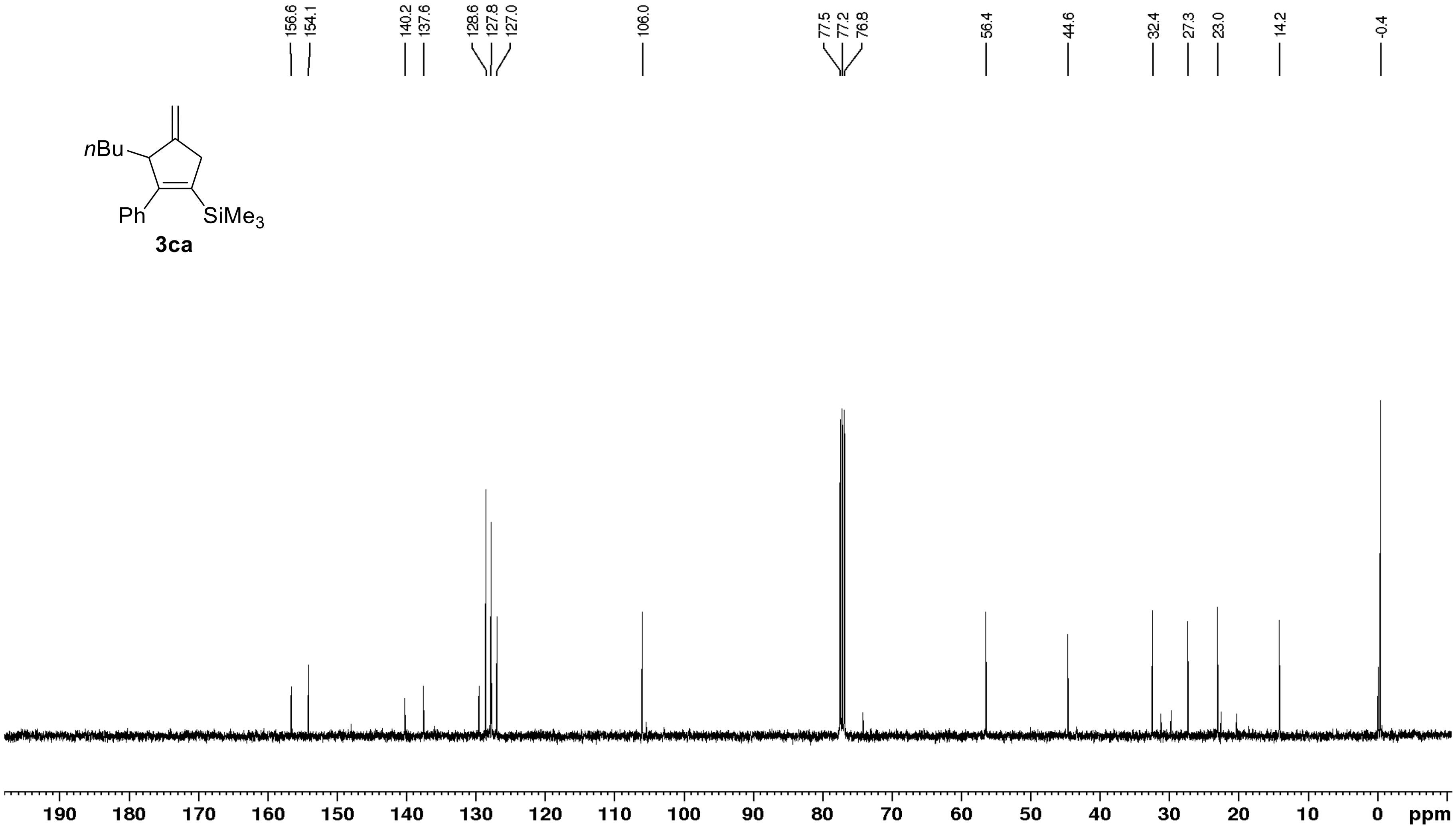

Figure S76. ^{29}Si DEPT NMR spectrum (79 MHz, $CDCl_3$, 298 K, optimized for J = 7.0 Hz) of **3ca**

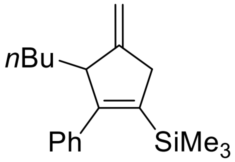

3ca

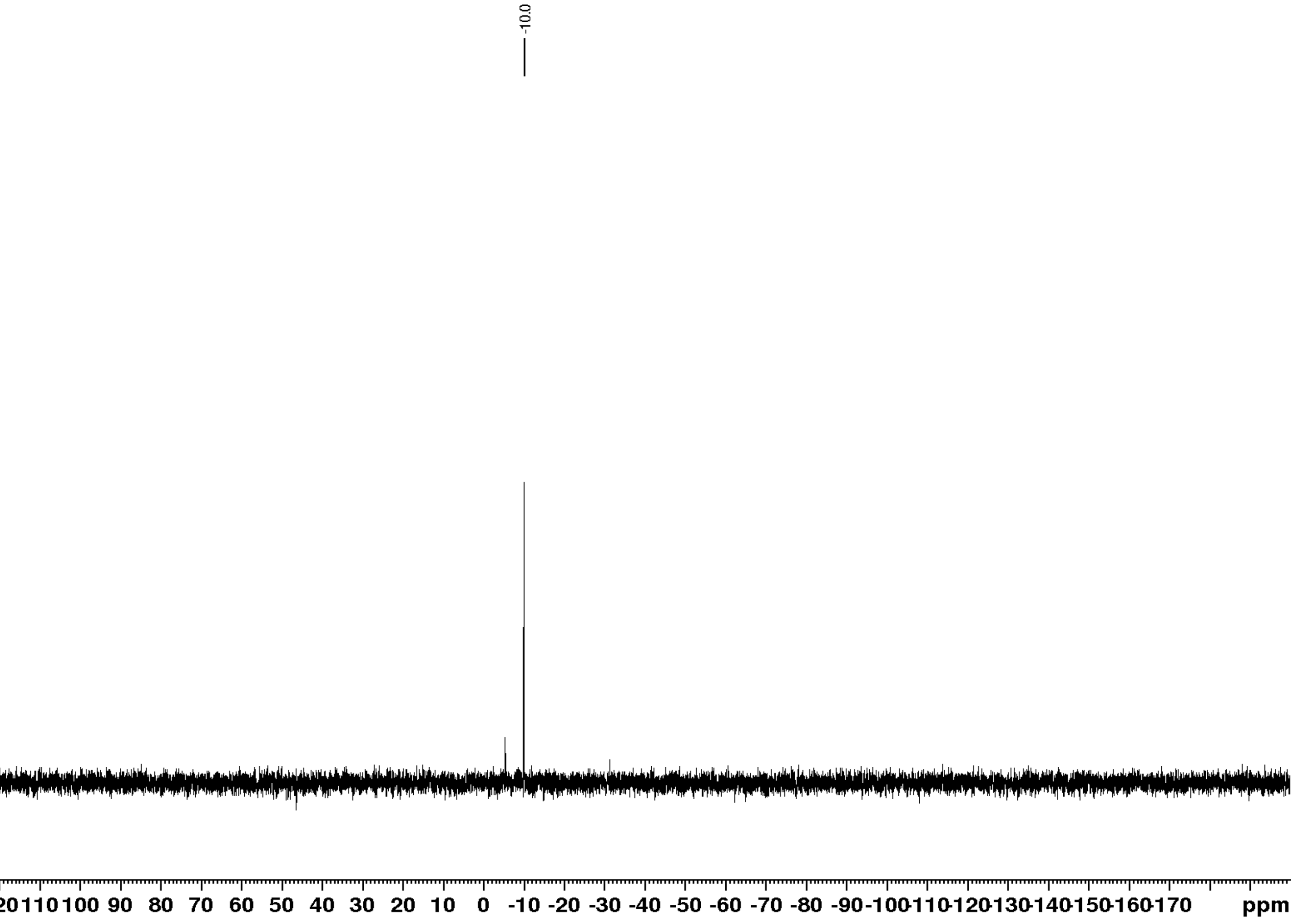

Figure S77. ^{1}H NMR spectrum (500 MHz, $CDCl_3$, 298 K) of **3da**

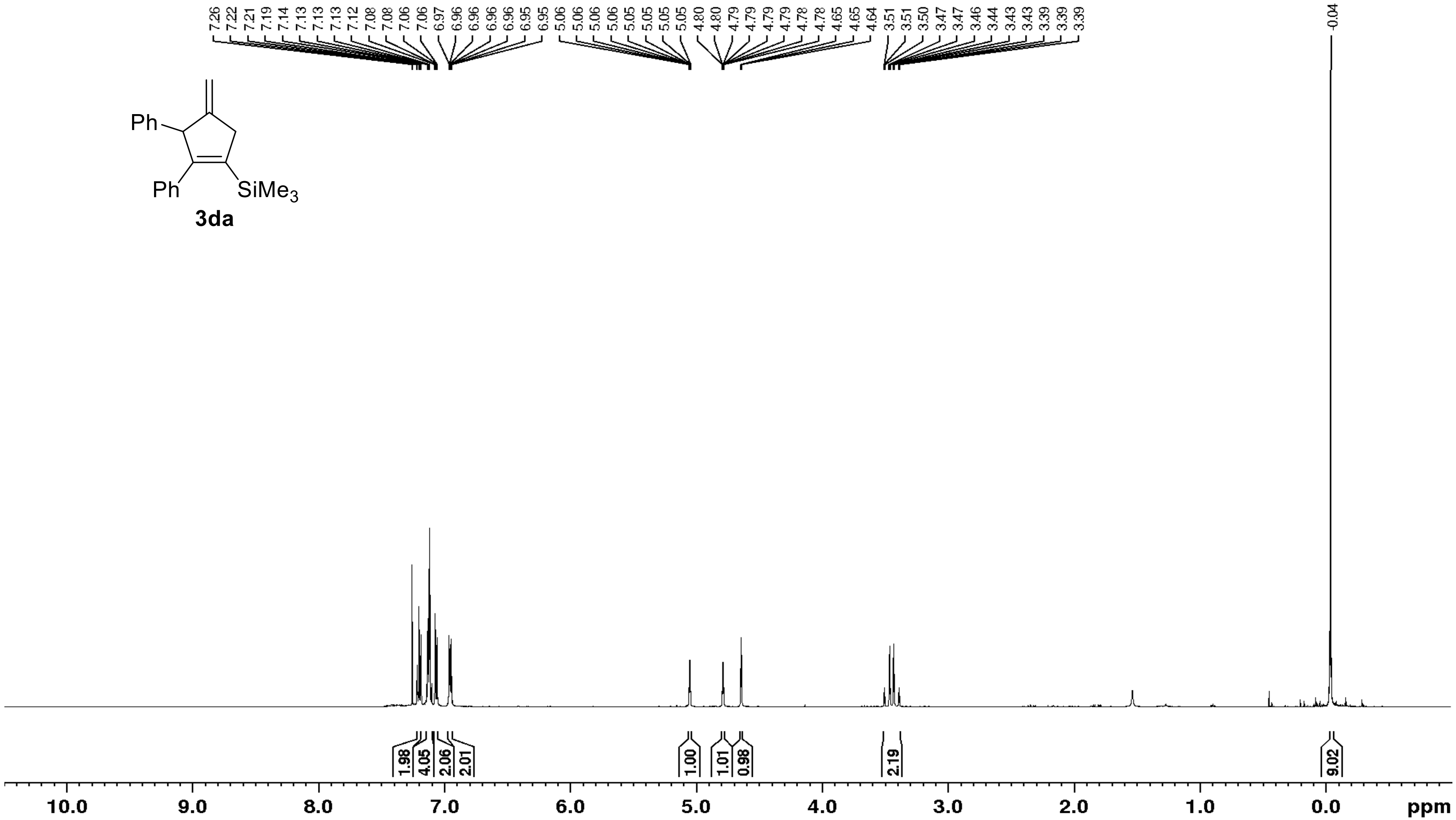

Figure S78. $^{13}C\{^1H\}$ NMR spectrum (126 MHz, $CDCl_3$, 298 K) of **3da**

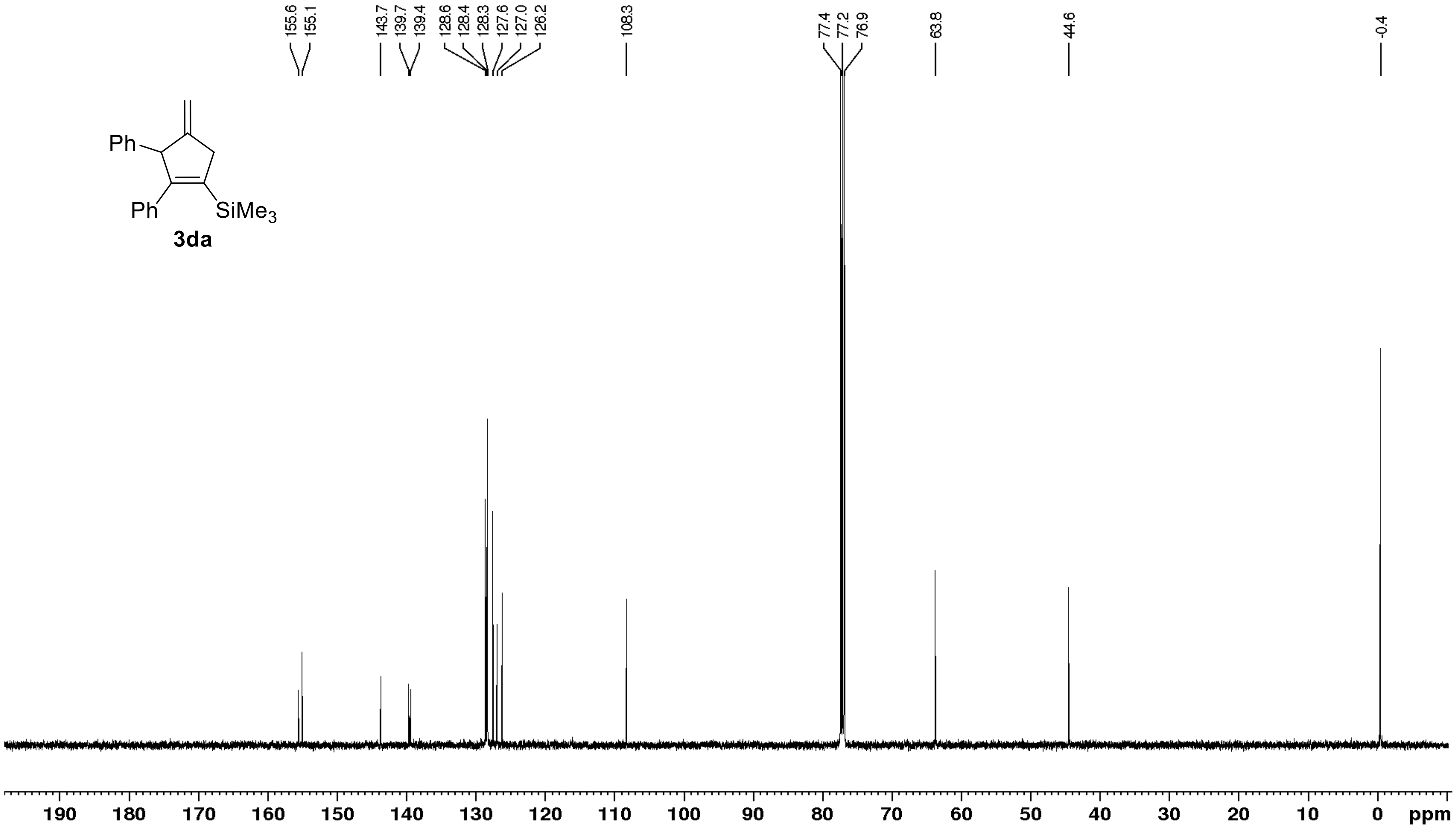

Figure S79. ^{29}Si DEPT NMR spectrum (79 MHz, $CDCl_3$, 298 K, optimized for *J* = 7.0 Hz) of **3da**

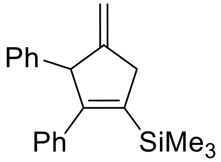

3da

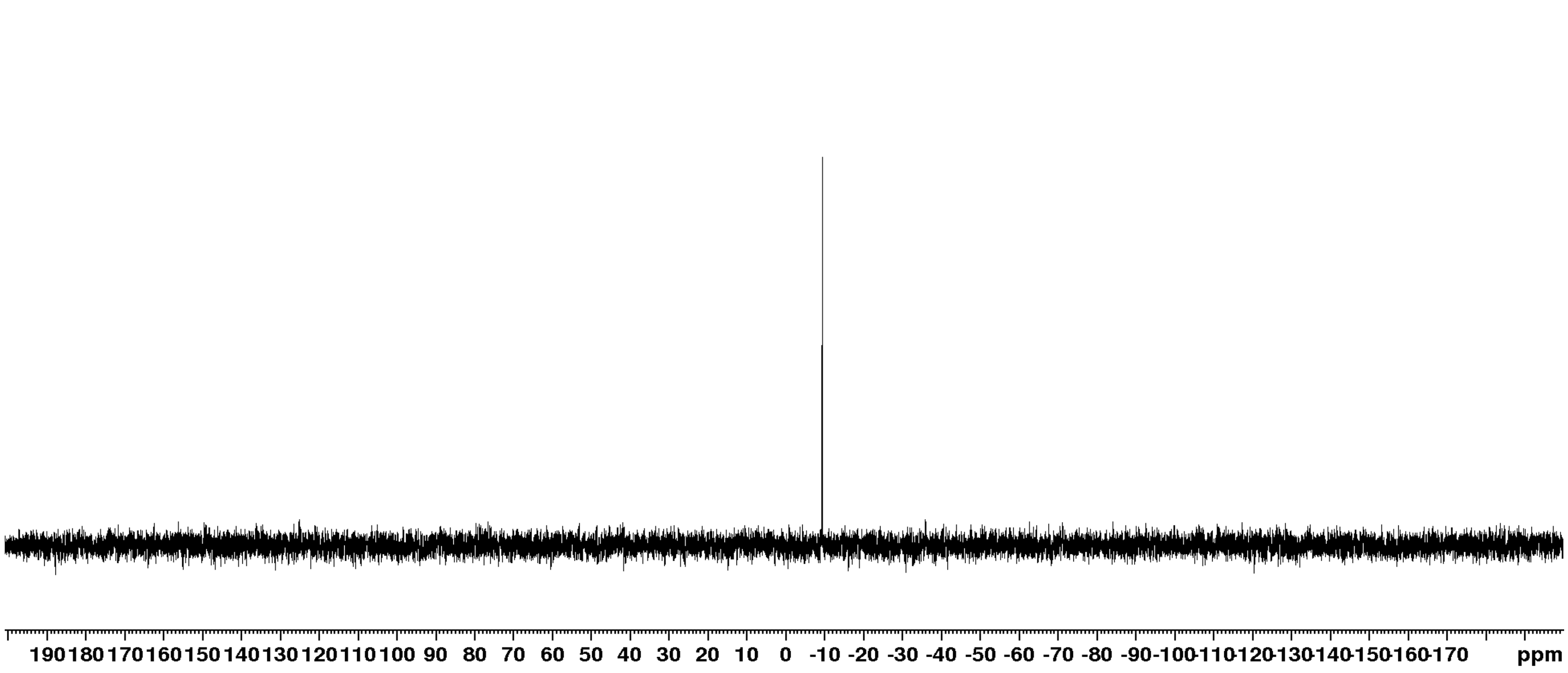

Figure S80. ^{1}H NMR spectrum (500 MHz, $CDCl_3$, 298 K) of **3fa**

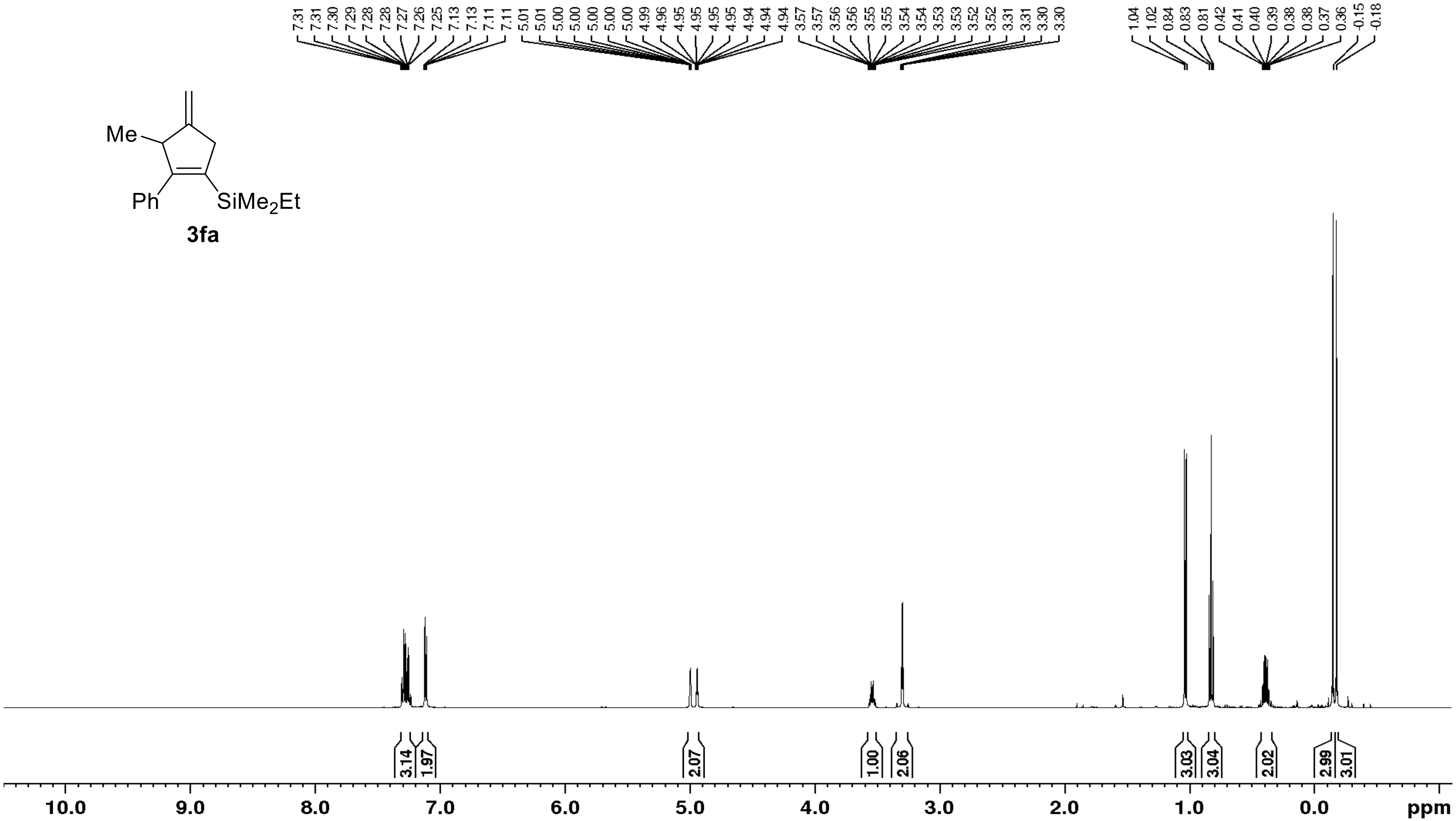

Figure S81. $^{13}C\{^1H\}$ NMR spectrum (126 MHz, $CDCl_3$, 298 K) of **3fa**

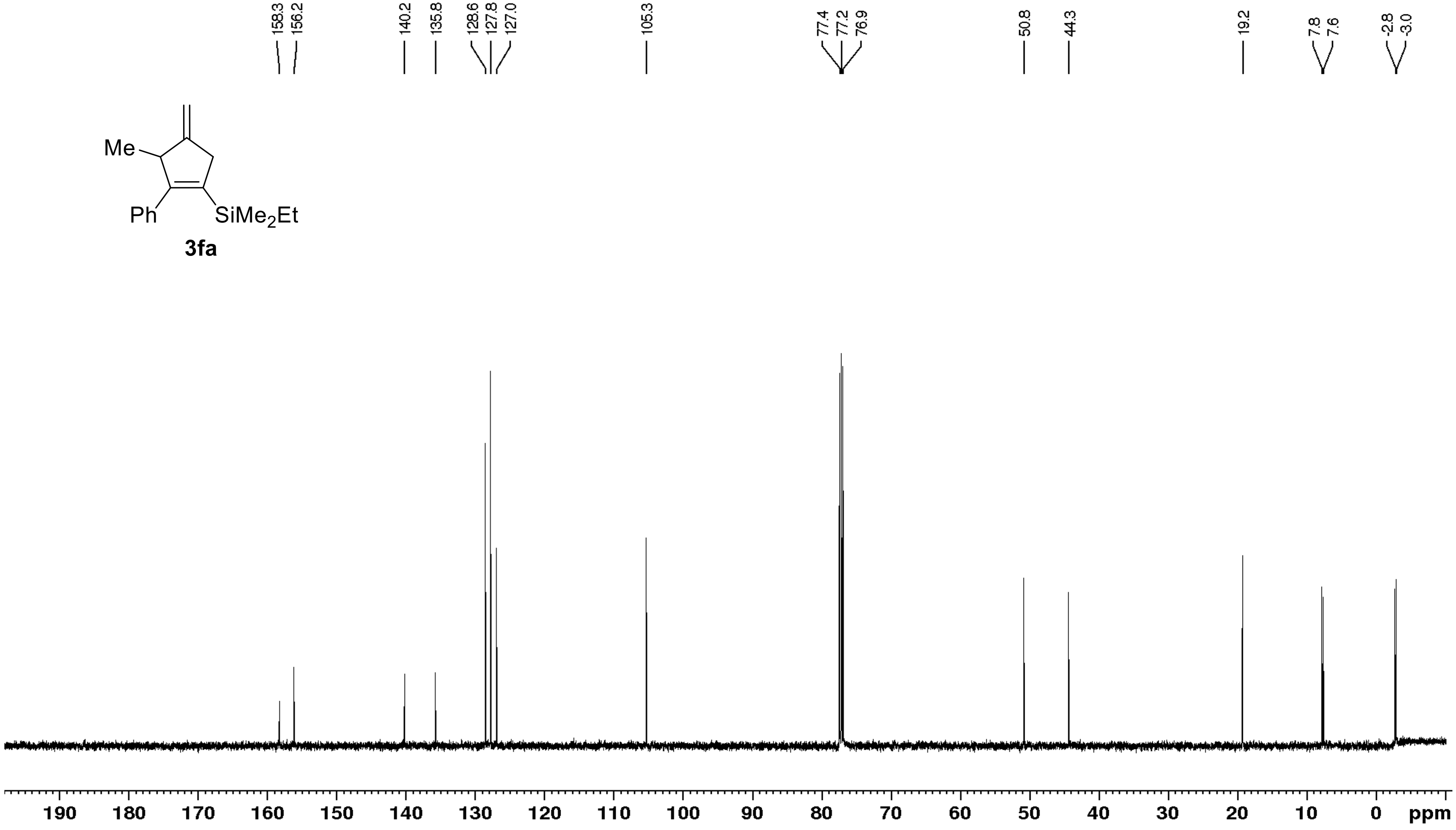

Figure S82. ^{29}Si DEPT NMR spectrum (99 MHz, $CDCl_3$, 298 K, optimized for *J* = 7.0 Hz) of **3fa**

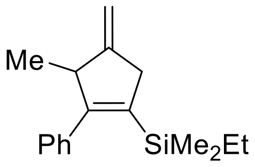

3fa

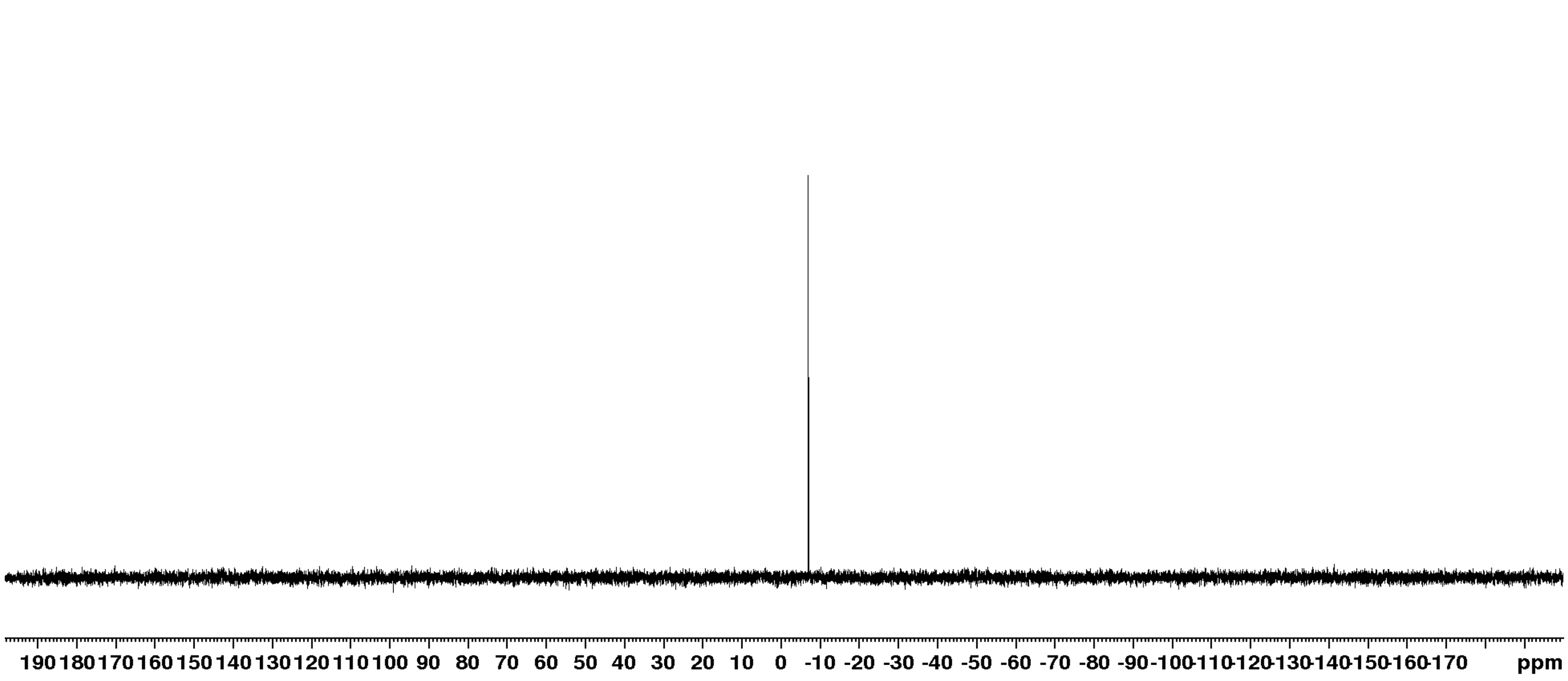

Figure S83. ^{1}H NMR spectrum (500 MHz, $CDCl_3$, 298 K) of **3ga**

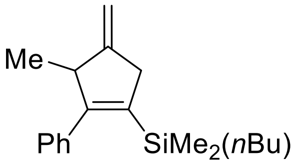

3ga

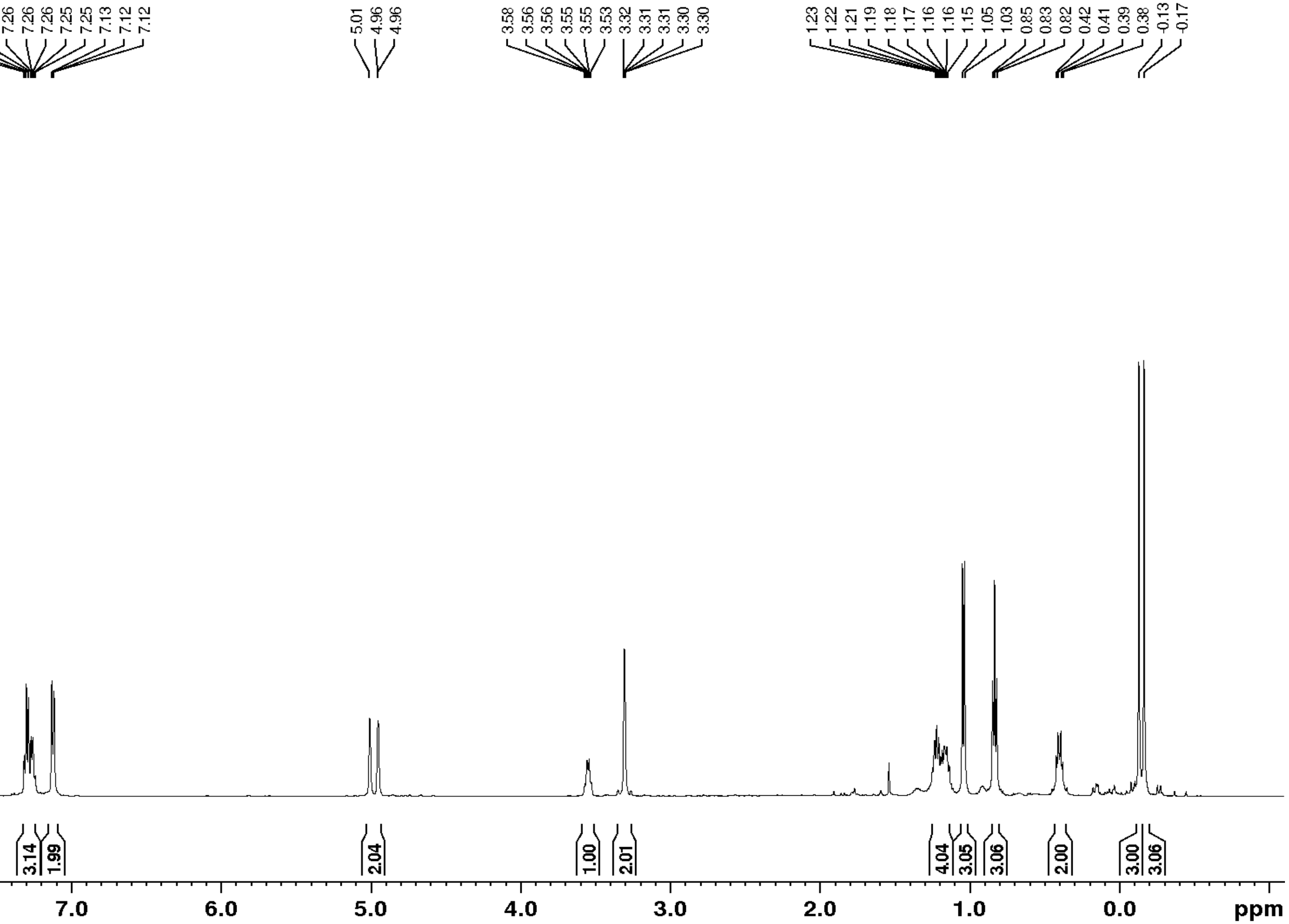

Figure S84. $^{13}C\{^{1}H\}$ NMR spectrum (101 MHz, $CDCl_3$, 298 K) of **3ga**

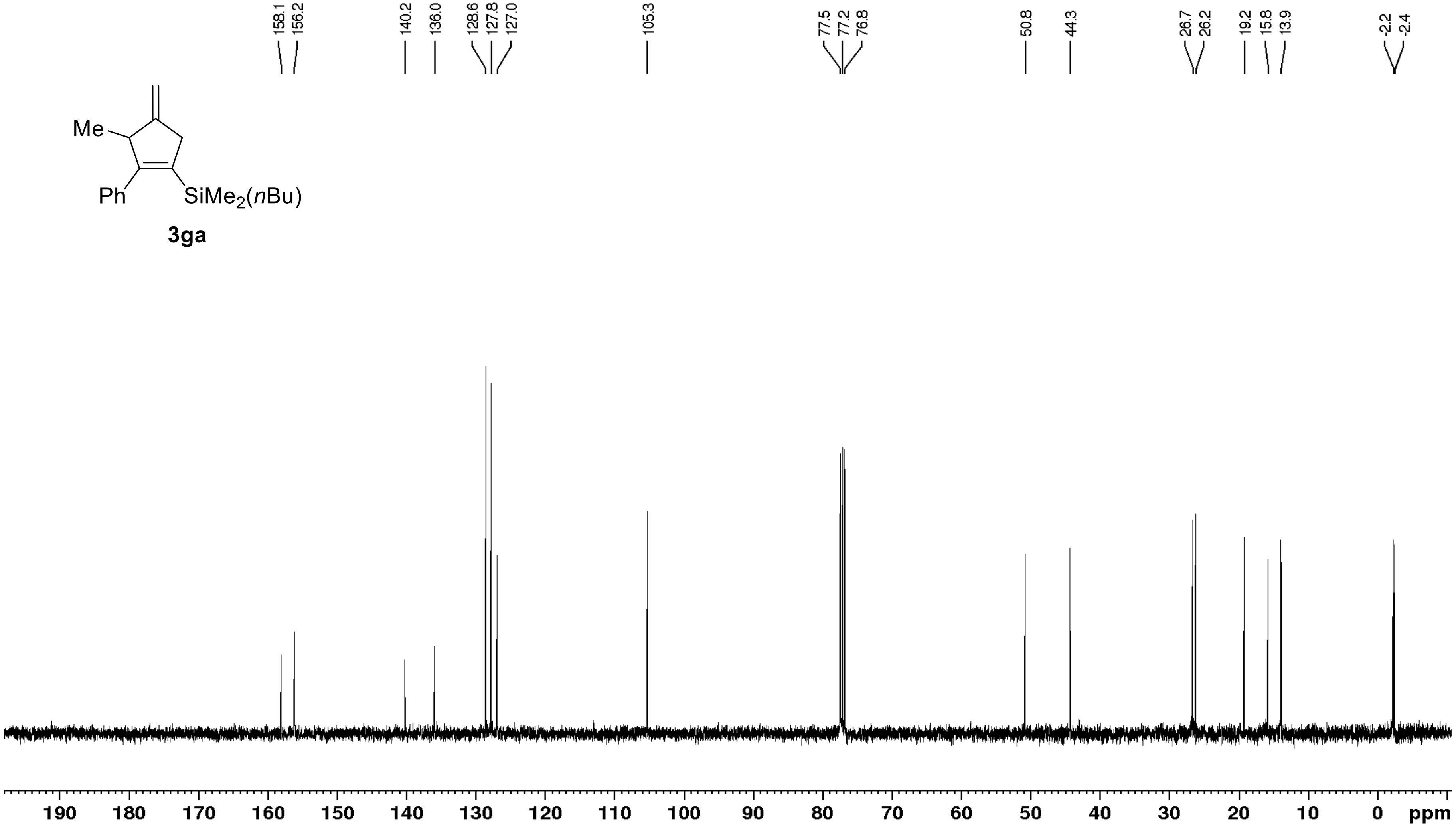

Figure S85. ^{29}Si DEPT NMR spectrum (79 MHz, $CDCl_3$, 298 K, optimized for J = 21.0 Hz) of **3ga**

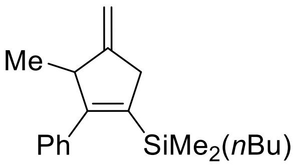

3ga

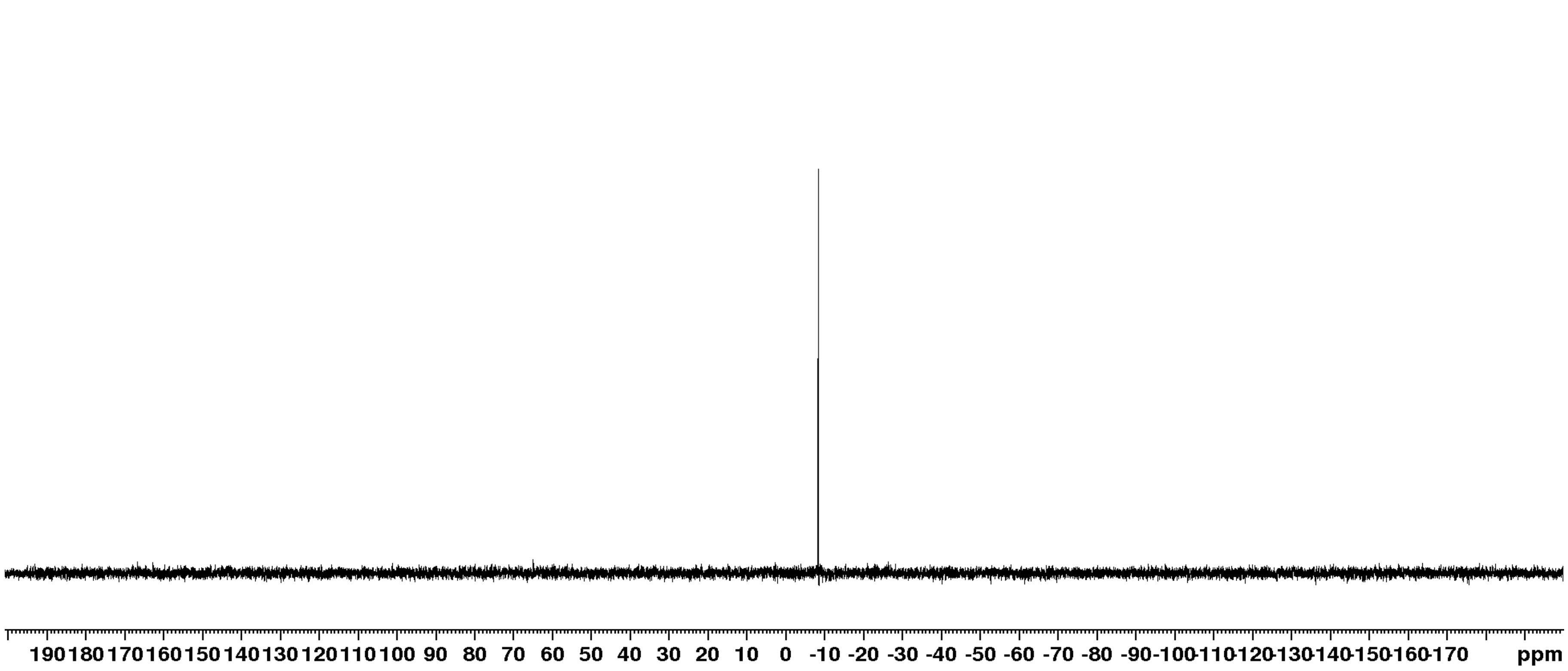

Figure S86. ^{1}H NMR spectrum (500 MHz, $CDCl_3$, 298 K) of **3ha**

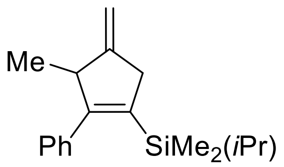

3ha

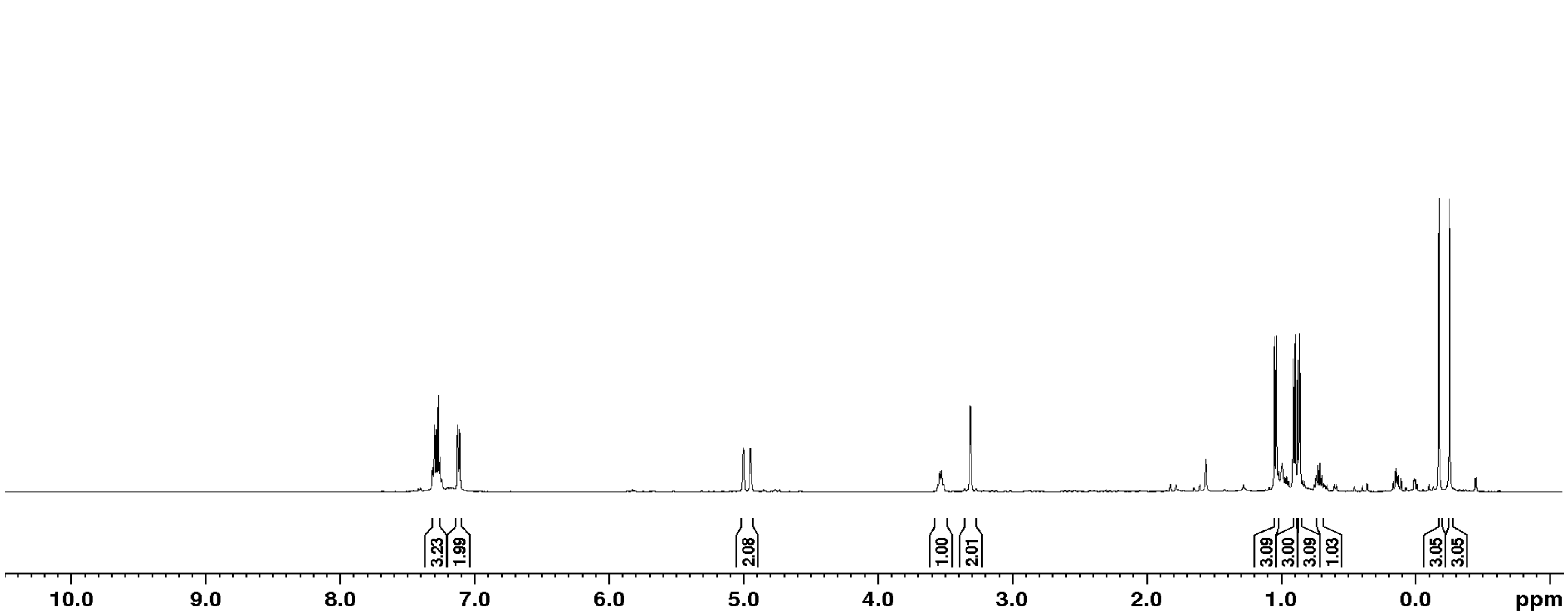

Figure S87. $^{13}C\{^{1}H\}$ NMR spectrum (101 MHz, $CDCl_3$, 298 K) of **3ha**

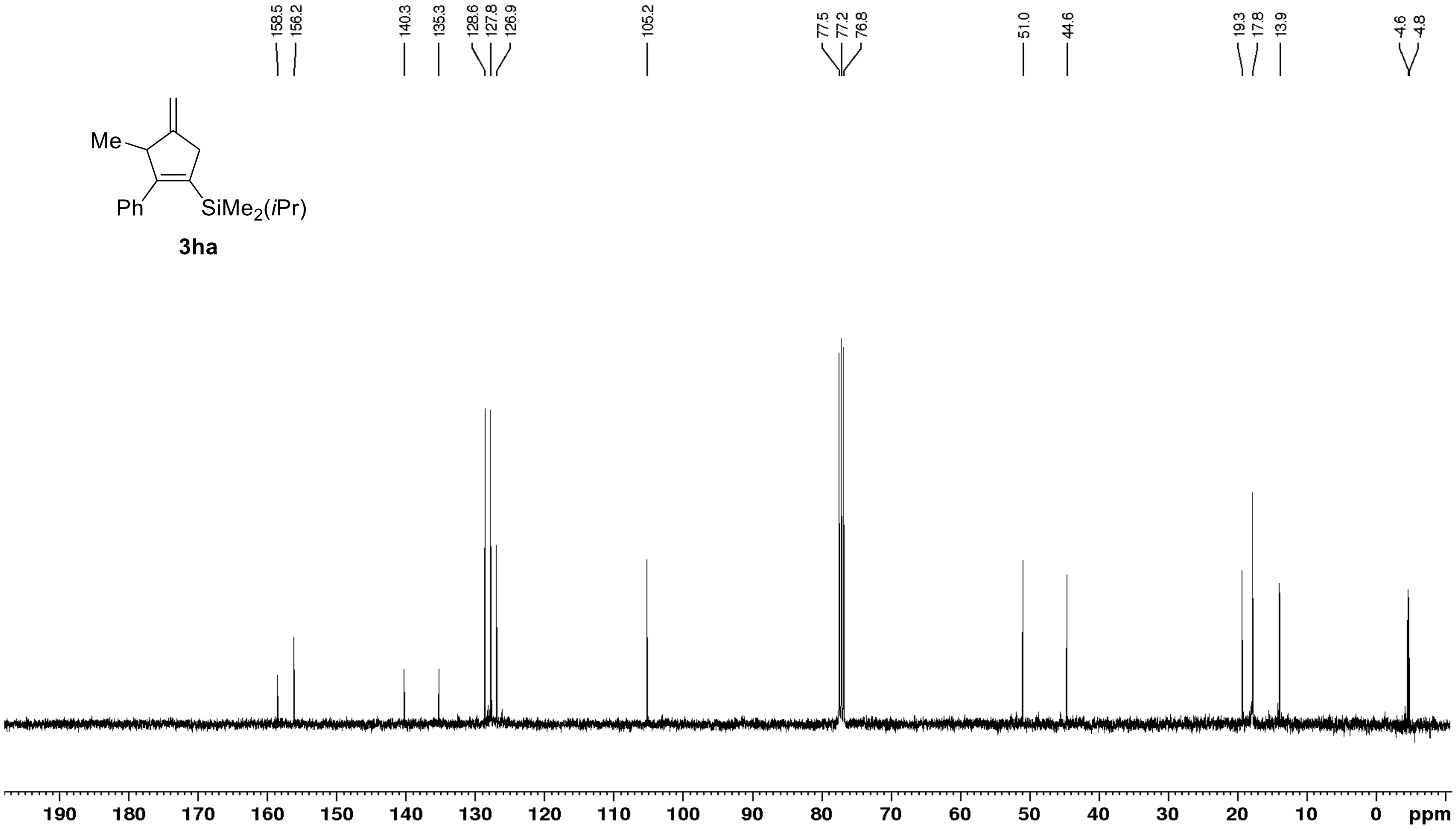

Figure S88. ^{29}Si DEPT NMR spectrum (79 MHz, $CDCl_3$, 298 K, optimized for J = 27.0 Hz) of **3ha**

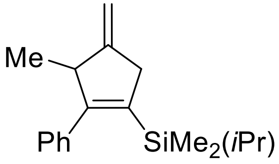

3ha

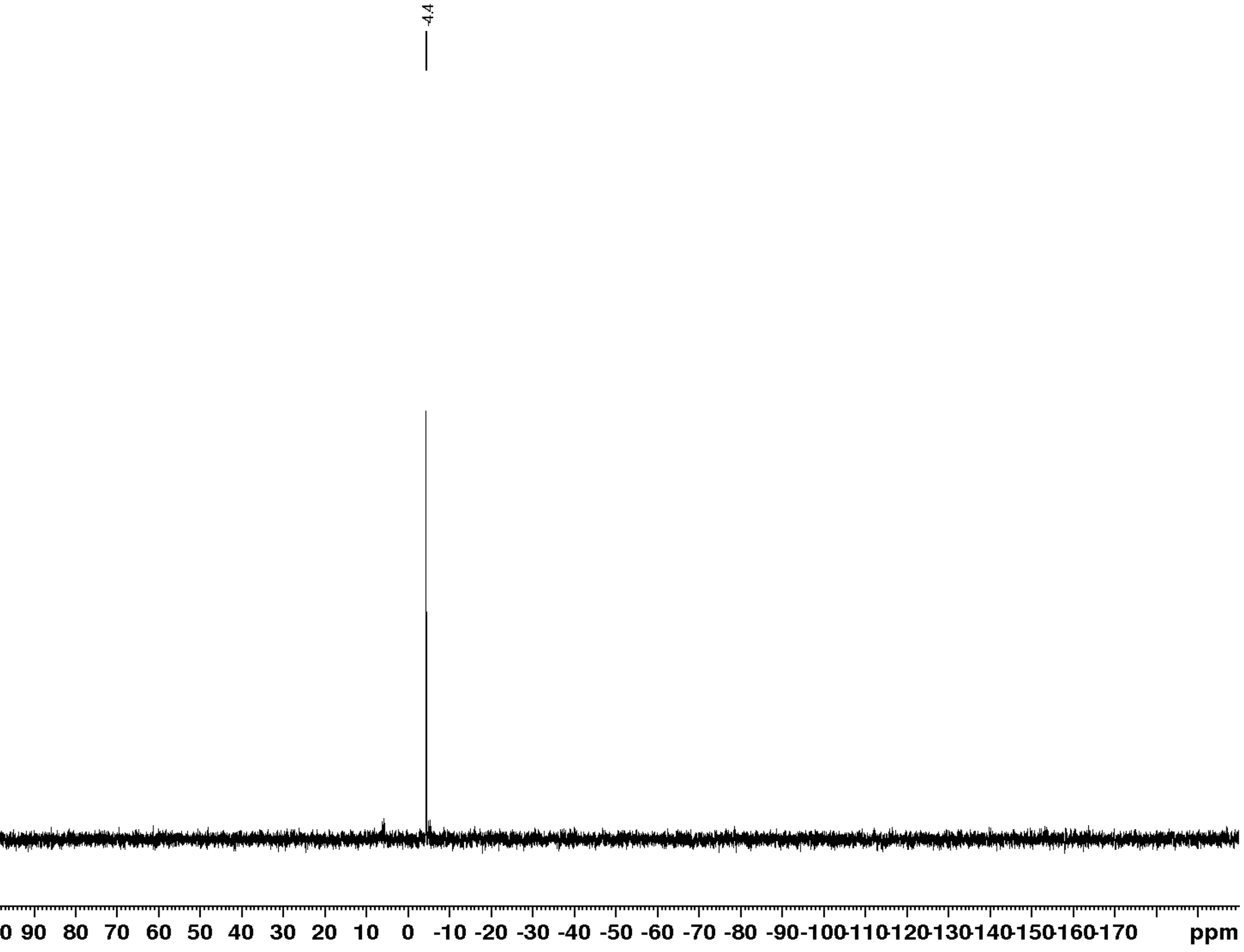

Figure S89. ^{1}H NMR spectrum (500 MHz, $CDCl_3$, 298 K) of **3ia**

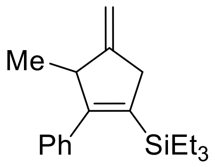

3ia

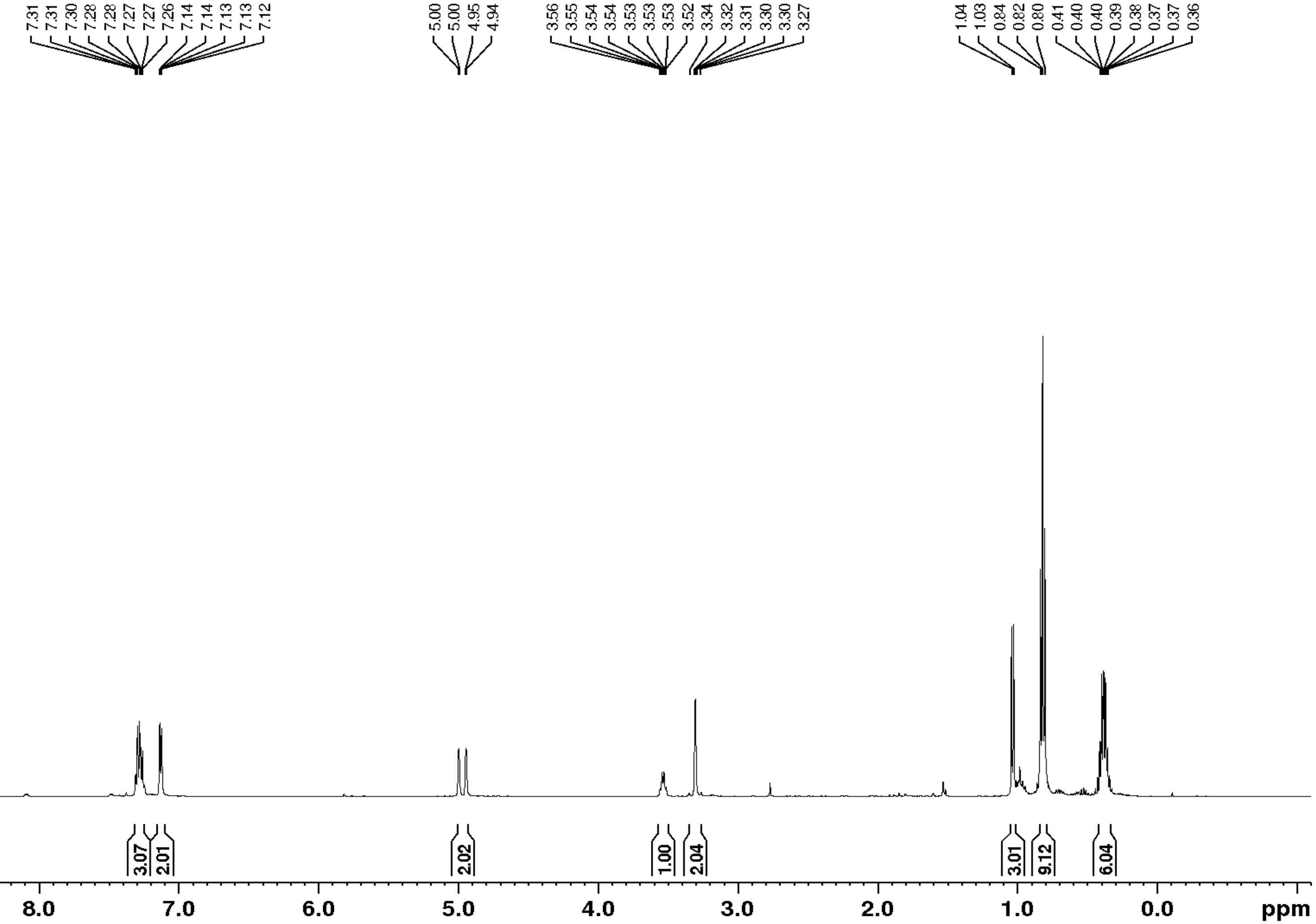

Figure S90. $^{13}C\{^1H\}$ NMR spectrum (126 MHz, $CDCl_3$, 298 K) of **3ia**

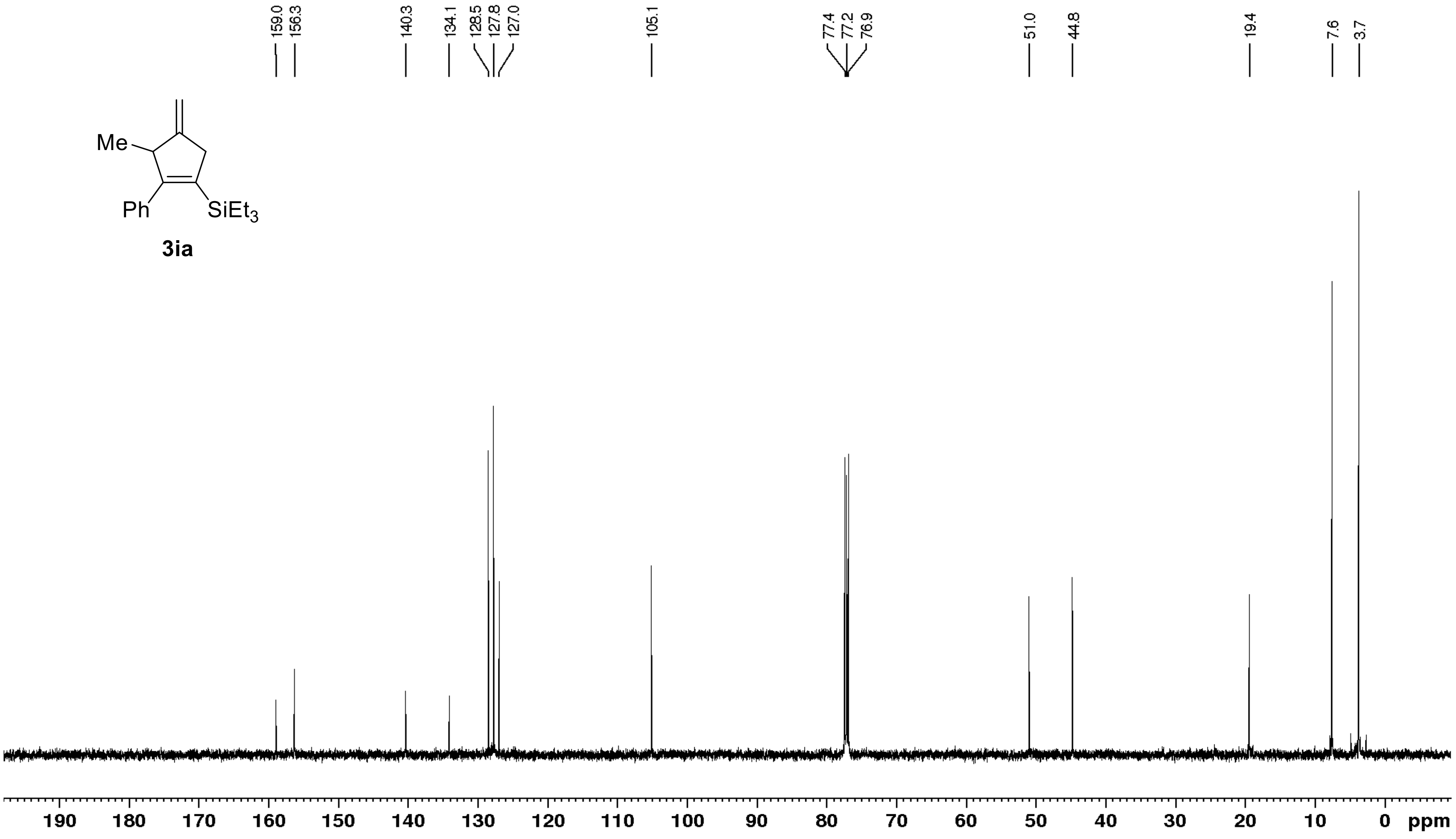

Figure S91. ^{29}Si DEPT NMR spectrum (99 MHz, $CDCl_3$, 298 K, optimized for *J* = 31.0 Hz) of **3ia**

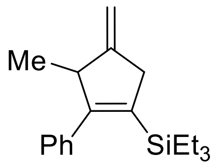

3ia

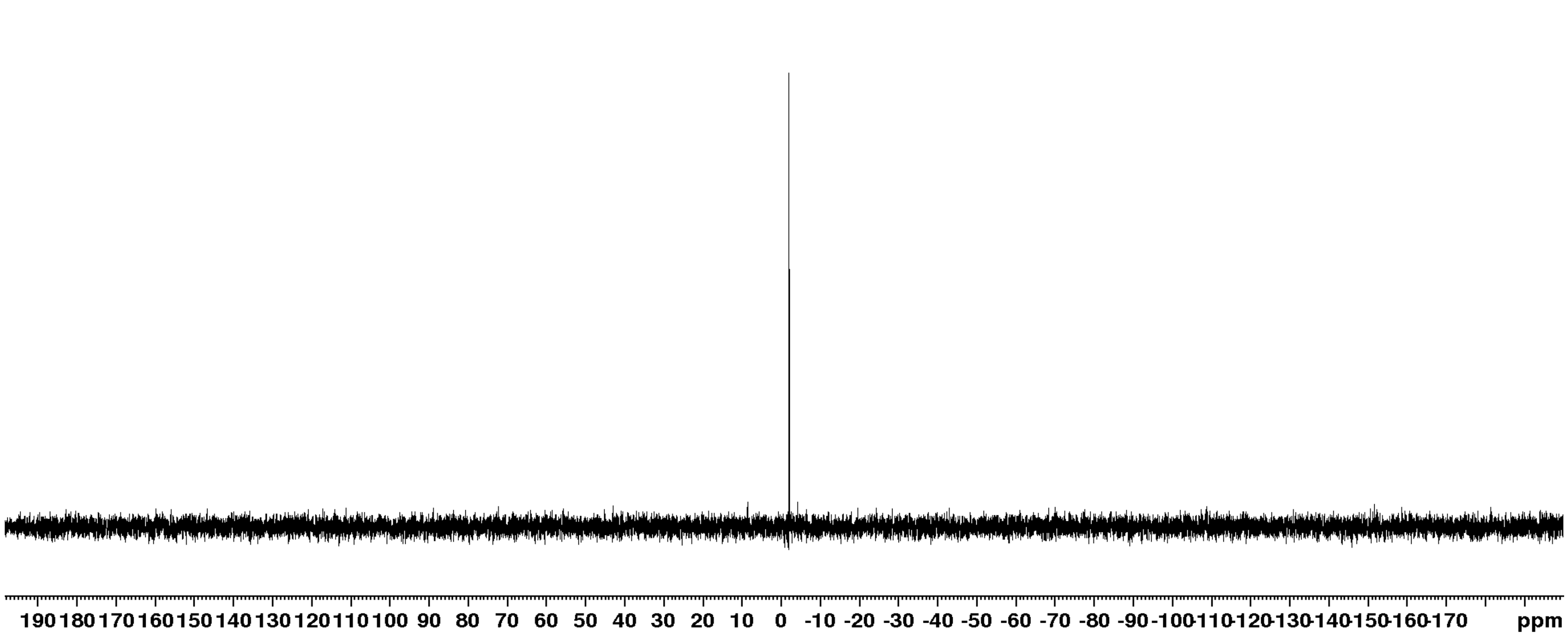

Figure S92. ^{1}H NMR spectrum (400 MHz, $CDCl_3$, 298 K) of **3ap**

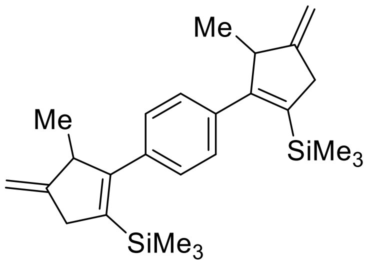

3ap

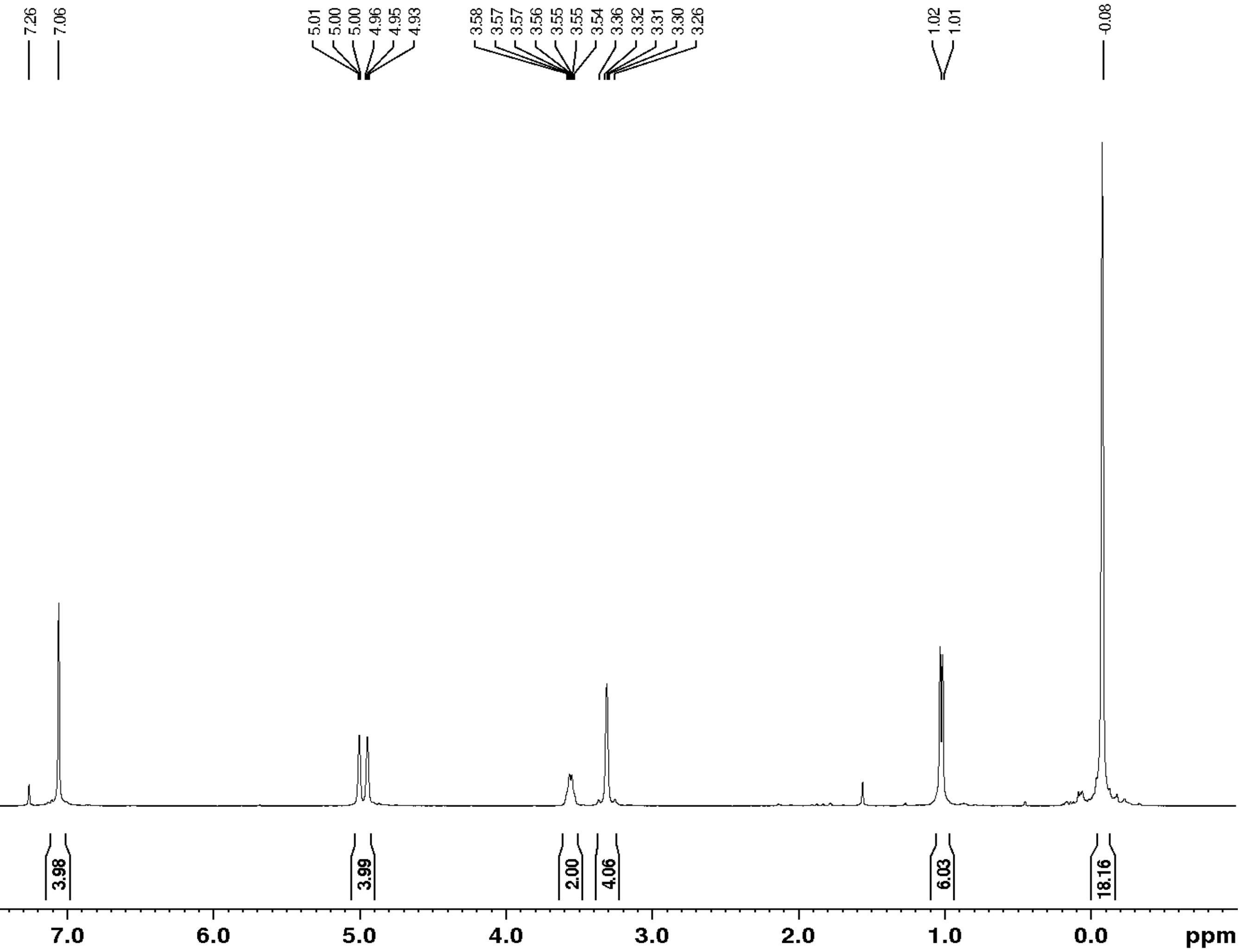

Figure S93. $^{13}C\{^{1}H\}$ NMR spectrum (101 MHz, $CDCl_3$, 298 K) of **3ap**

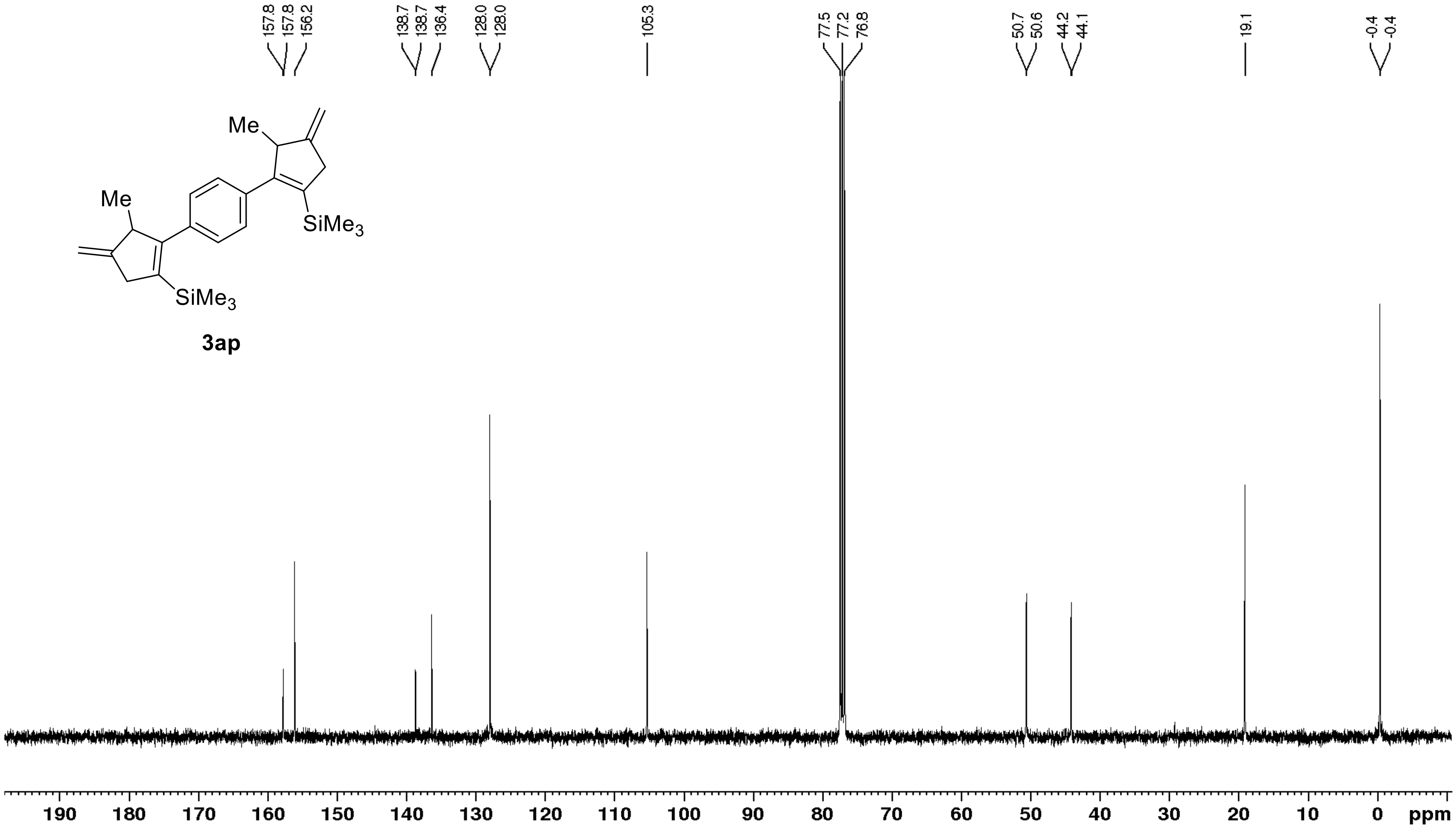

Figure S94. ^{29}Si DEPT NMR spectrum (79 MHz, $CDCl_3$, 298 K, optimized for J = 7.0 Hz) of **3ap**

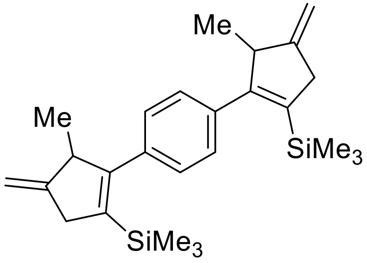

3ap

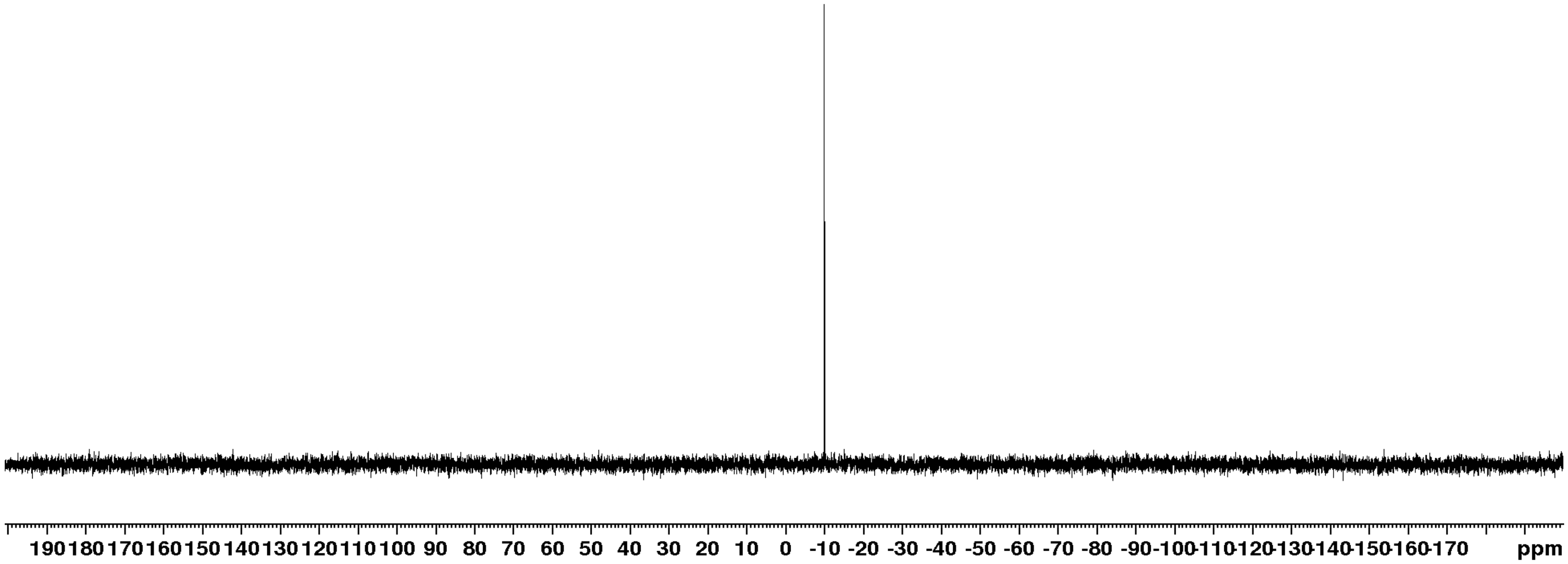

Figure S95. ^{1}H NMR spectrum (400 MHz, $CDCl_3$, 298 K) of **3aq**

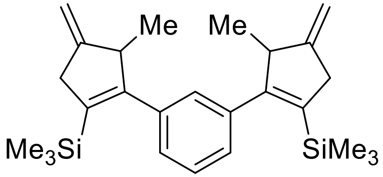

3aq

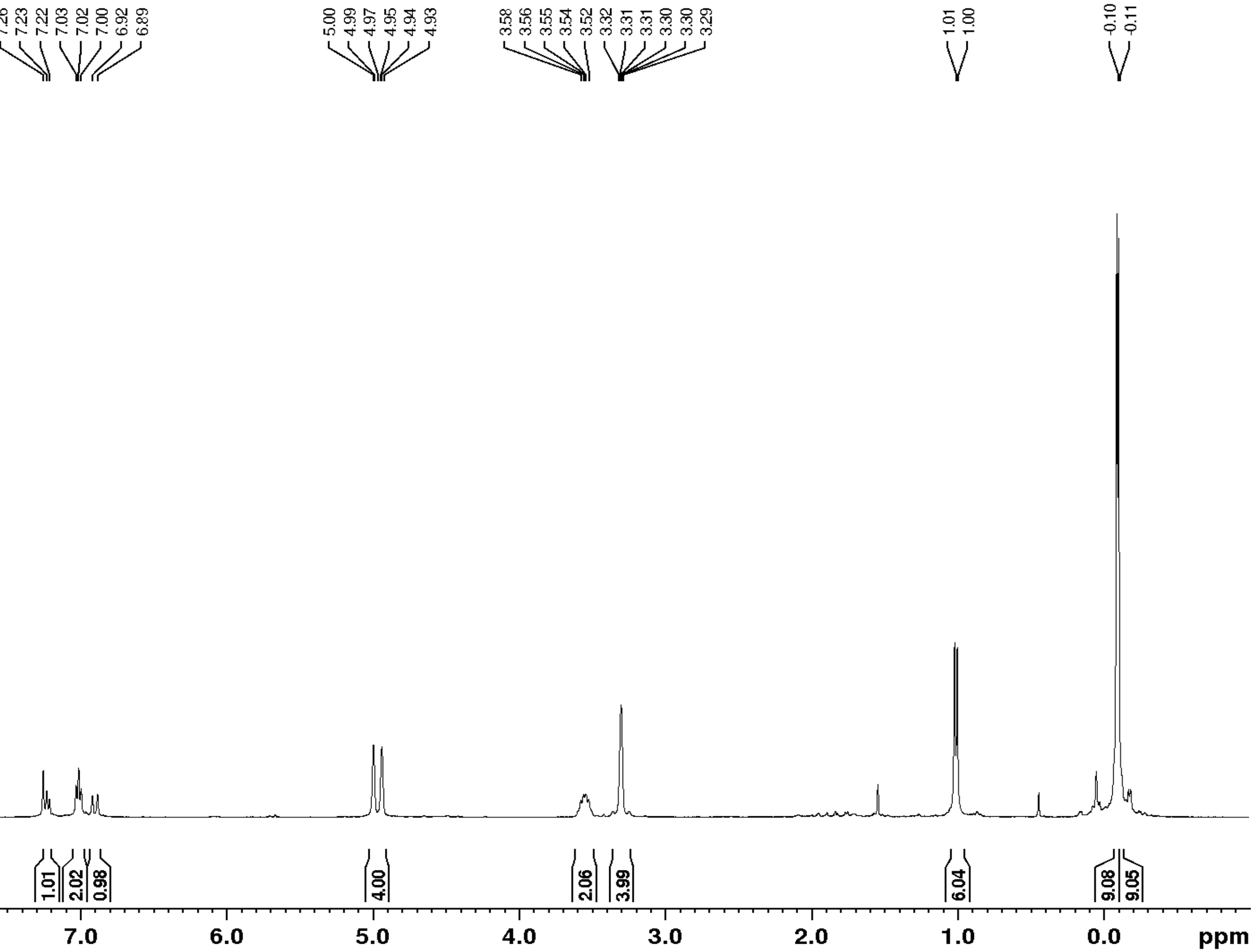

Figure S96. $^{13}C\{^{1}H\}$ NMR spectrum (101 MHz, $CDCl_3$, 298 K) of **3aq**

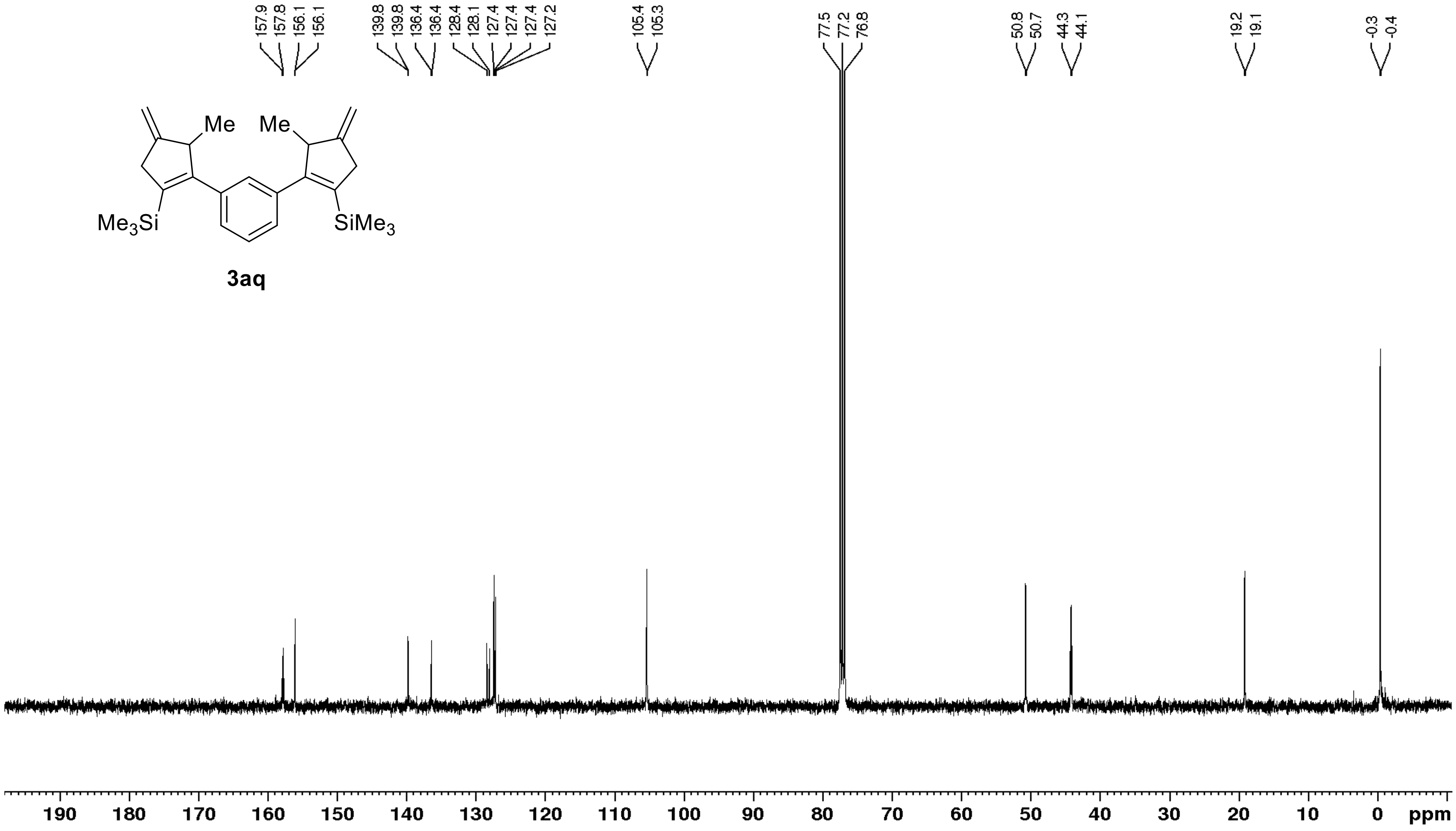

Figure S97. ^{29}Si DEPT NMR spectrum (79 MHz, $CDCl_3$, 298 K, optimized for *J* = 7.0 Hz) of **3aq**

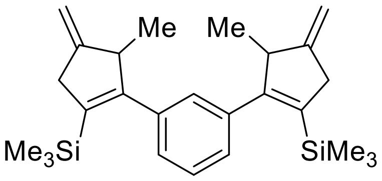

3aq

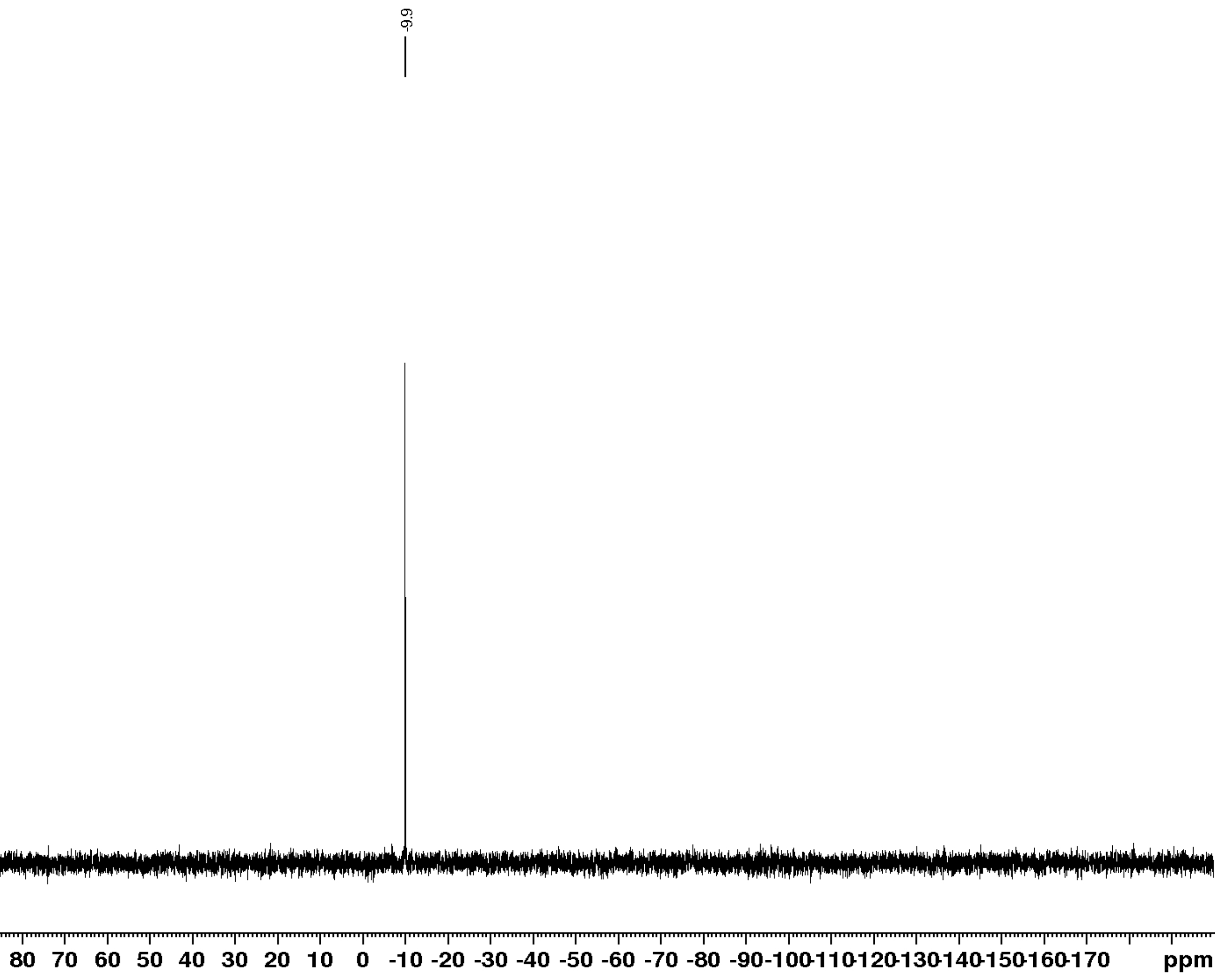

Figure S98. ^{1}H NMR spectrum (500 MHz, $CDCl_3$, 298 K) of **3aa**-d_3

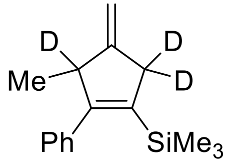

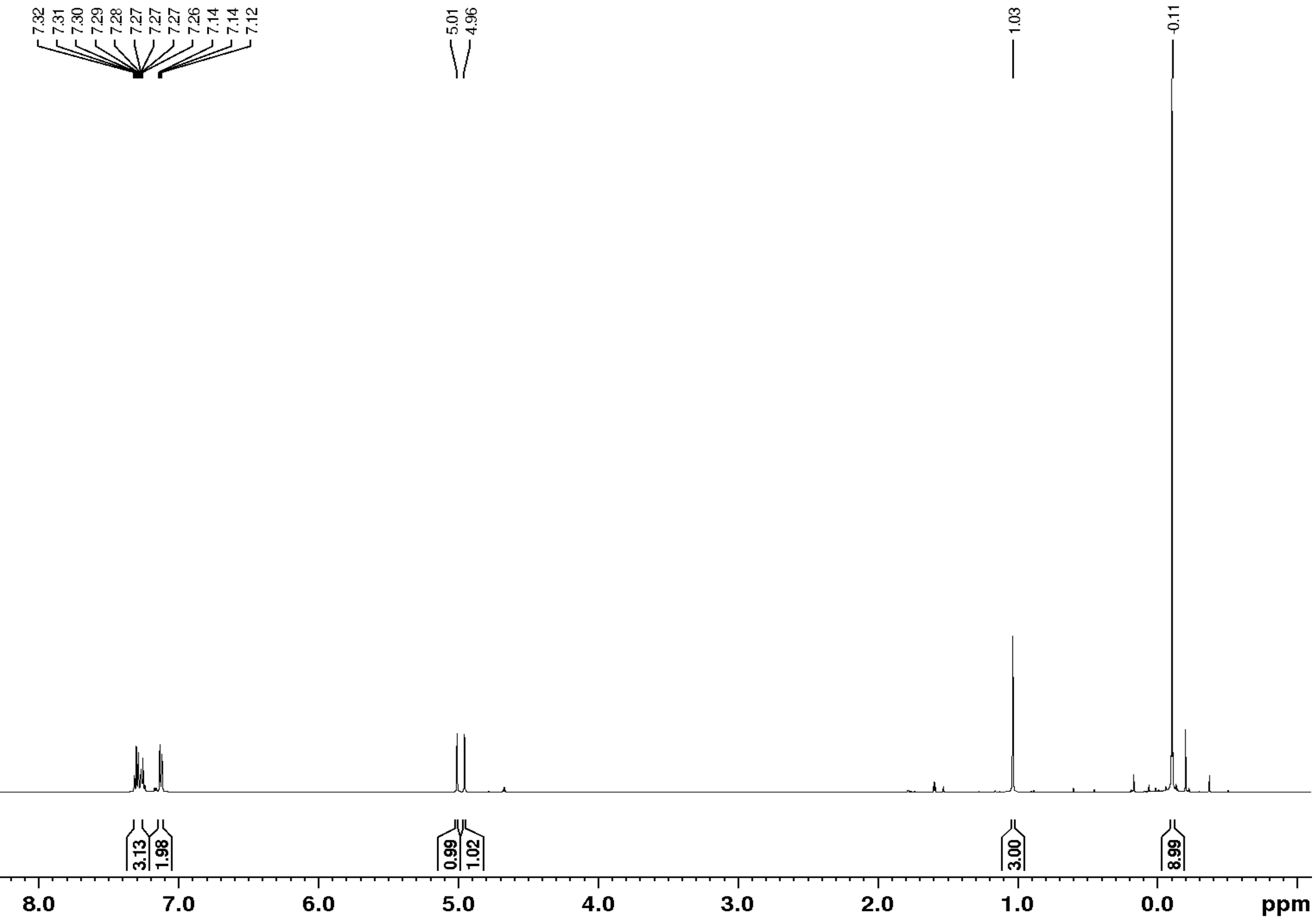

Figure S99. $^{13}C\{^{1}H\}$ NMR spectrum (126 MHz, $CDCl_3$, 298 K) of **3aa**-d_3

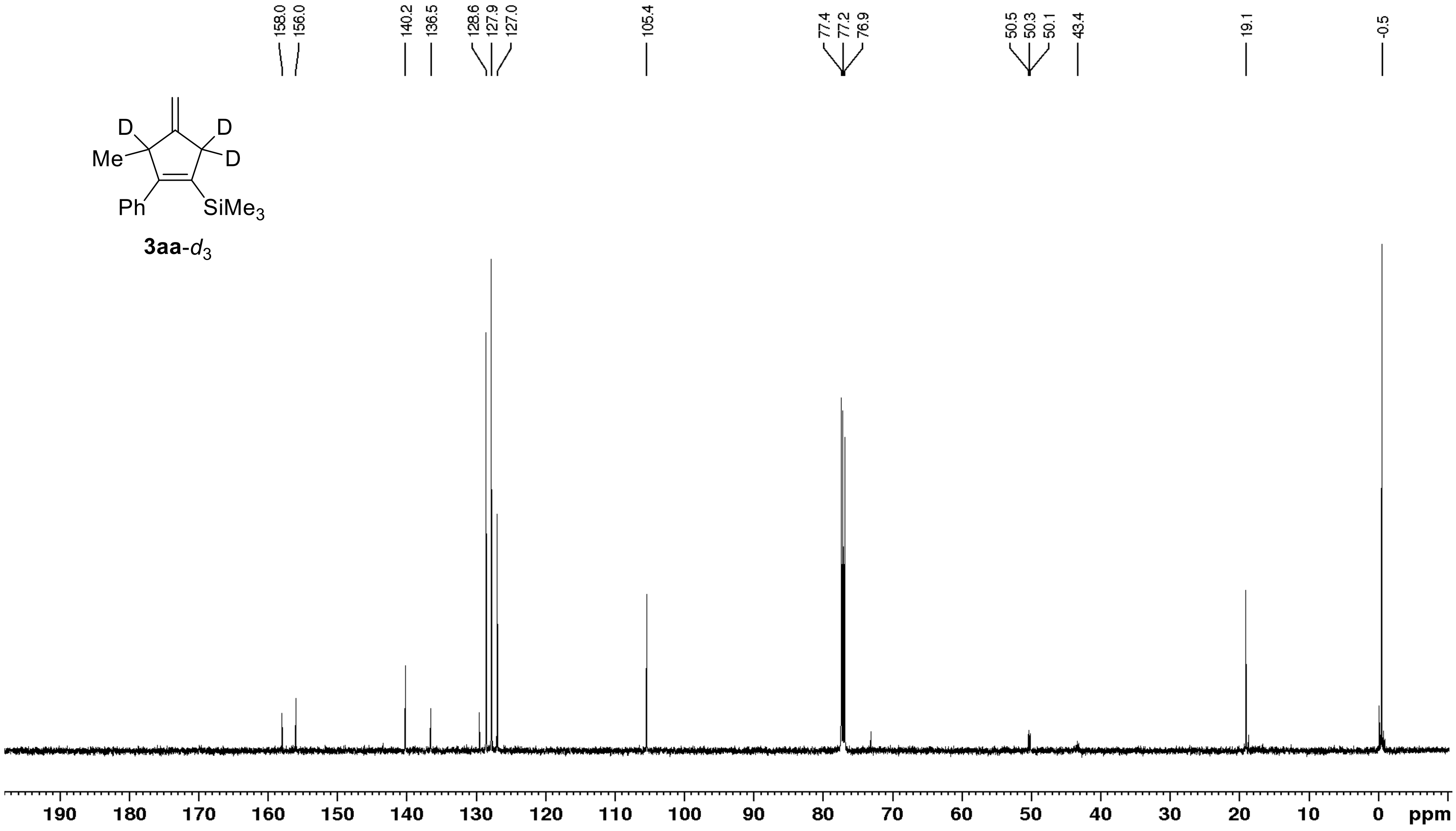

Figure S100. ^{2}H NMR spectrum (77 MHz, $CDCl_3$, 298 K) of **3aa**-*d*$_3$

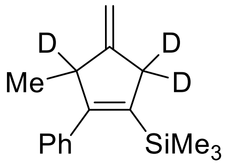

3aa-*d*$_3$

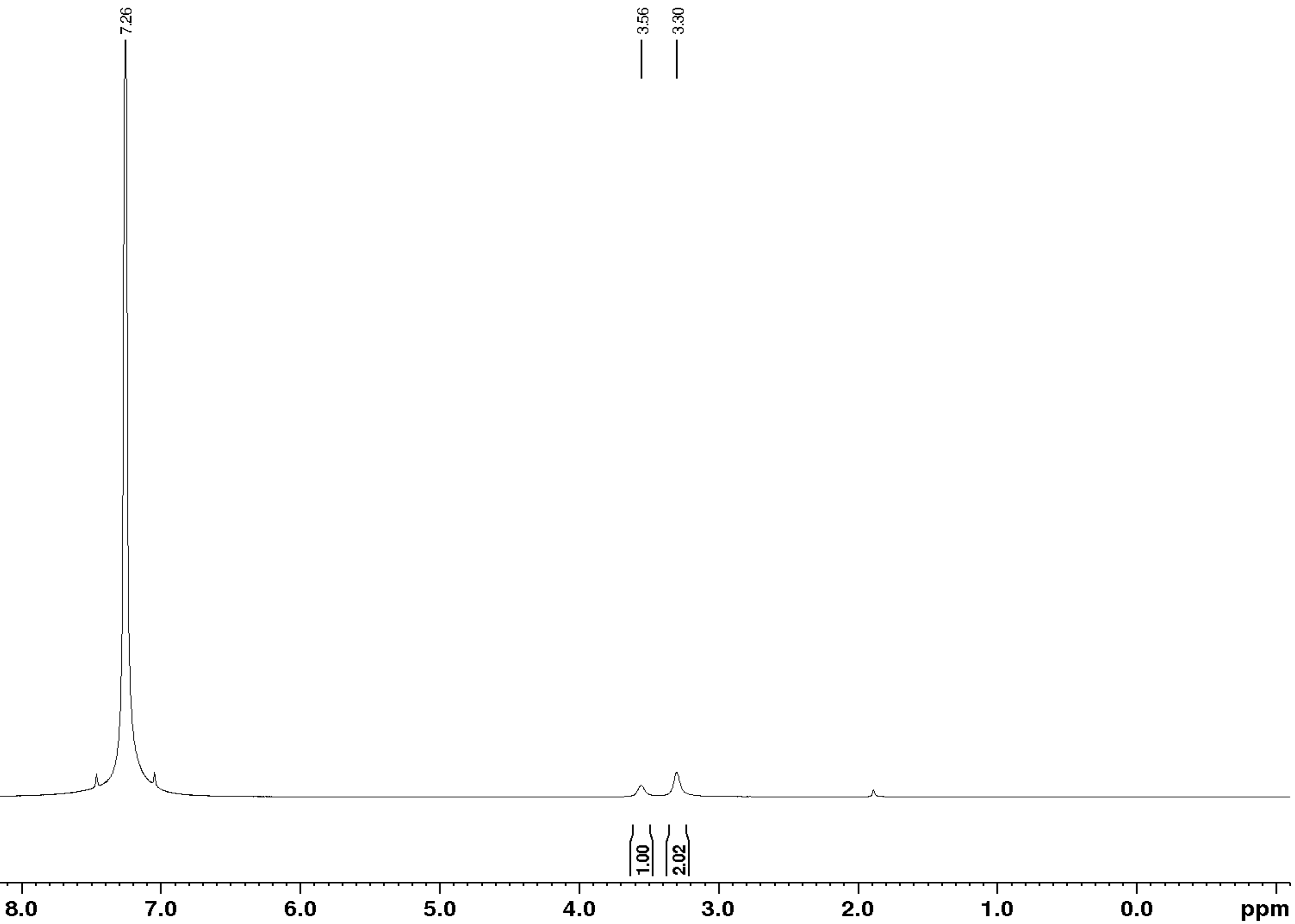

Figure S101. ^{29}Si DEPT NMR spectrum (99 MHz, $CDCl_3$, 298 K, optimized for J = 7.0 Hz) of **3aa**-d_3

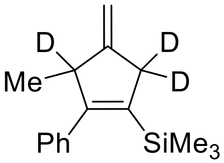

3aa-d_3

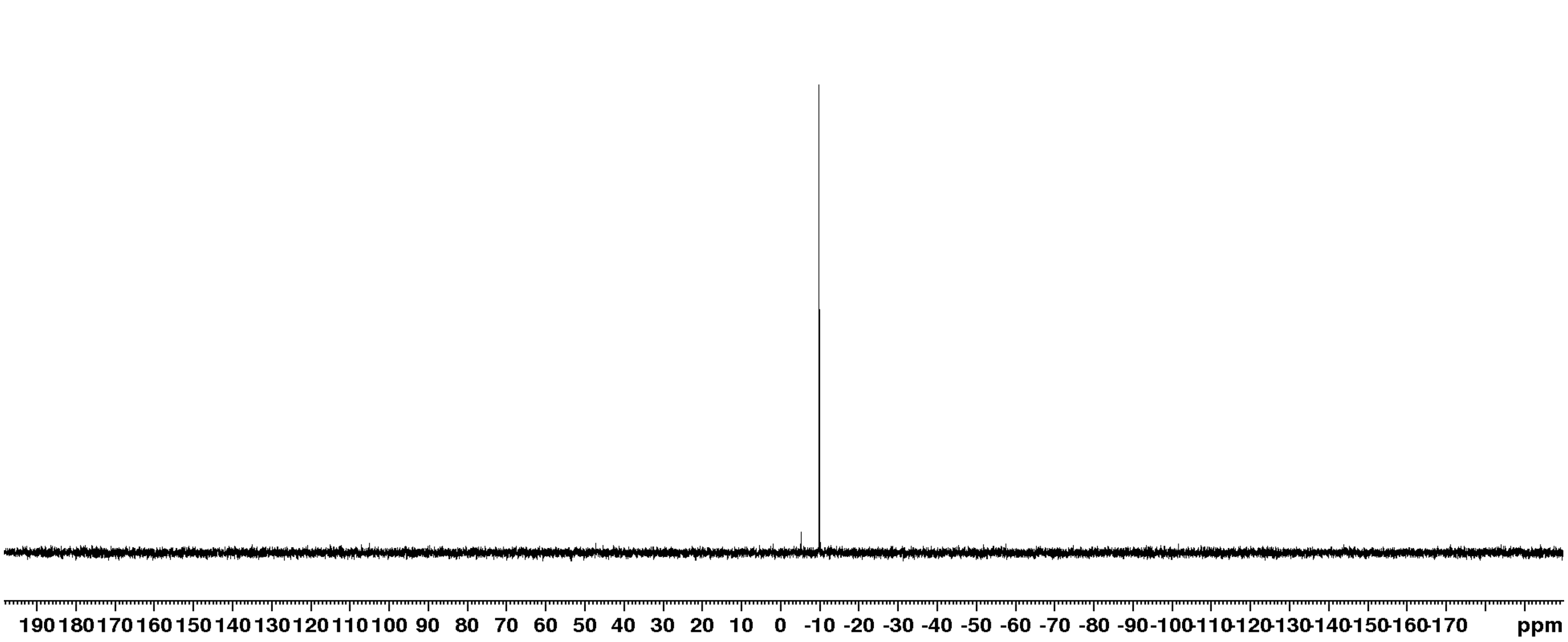

Figure S102. ^{1}H NMR spectrum (400 MHz, $CDCl_3$, 298 K) of **4a**

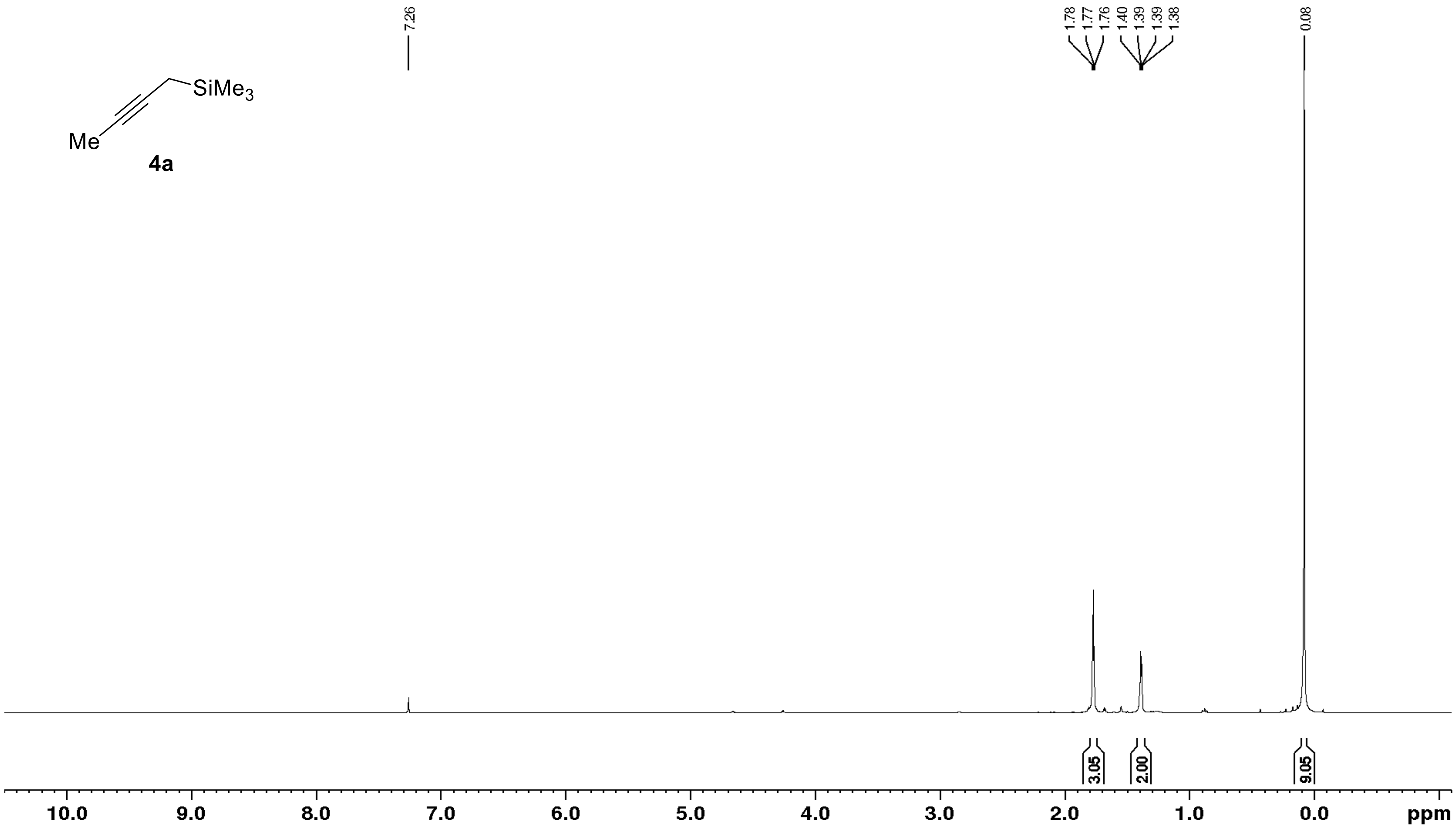

Figure S103. $^{13}C\{^{1}H\}$ NMR spectrum (101 MHz, $CDCl_3$, 298 K) of **4a**

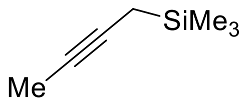

4a

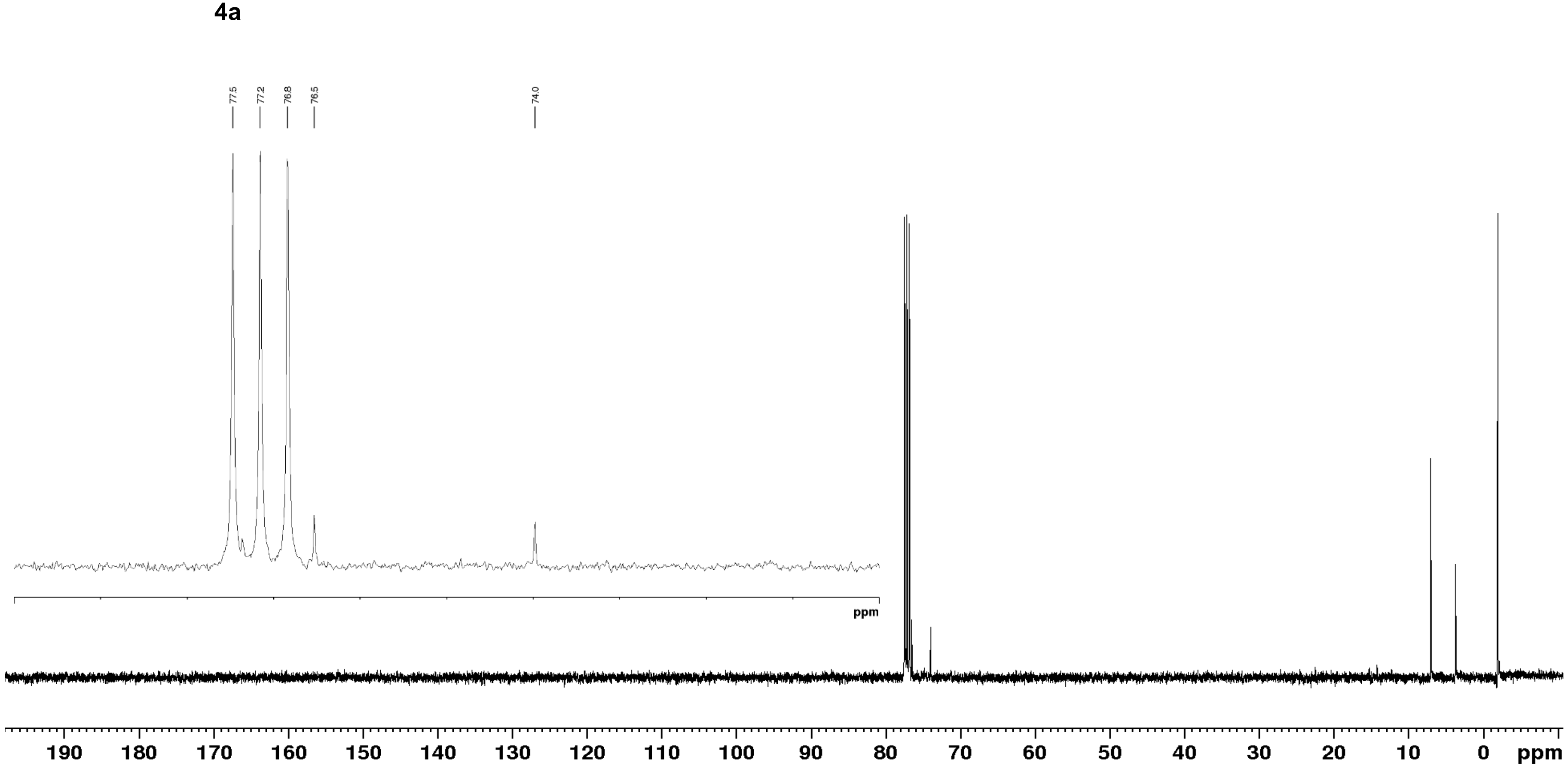

Figure S104. ^{29}Si DEPT NMR spectrum (79 MHz, $CDCl_3$, 298 K, optimized for J = 7.0 Hz) of **4a**

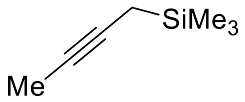

4a

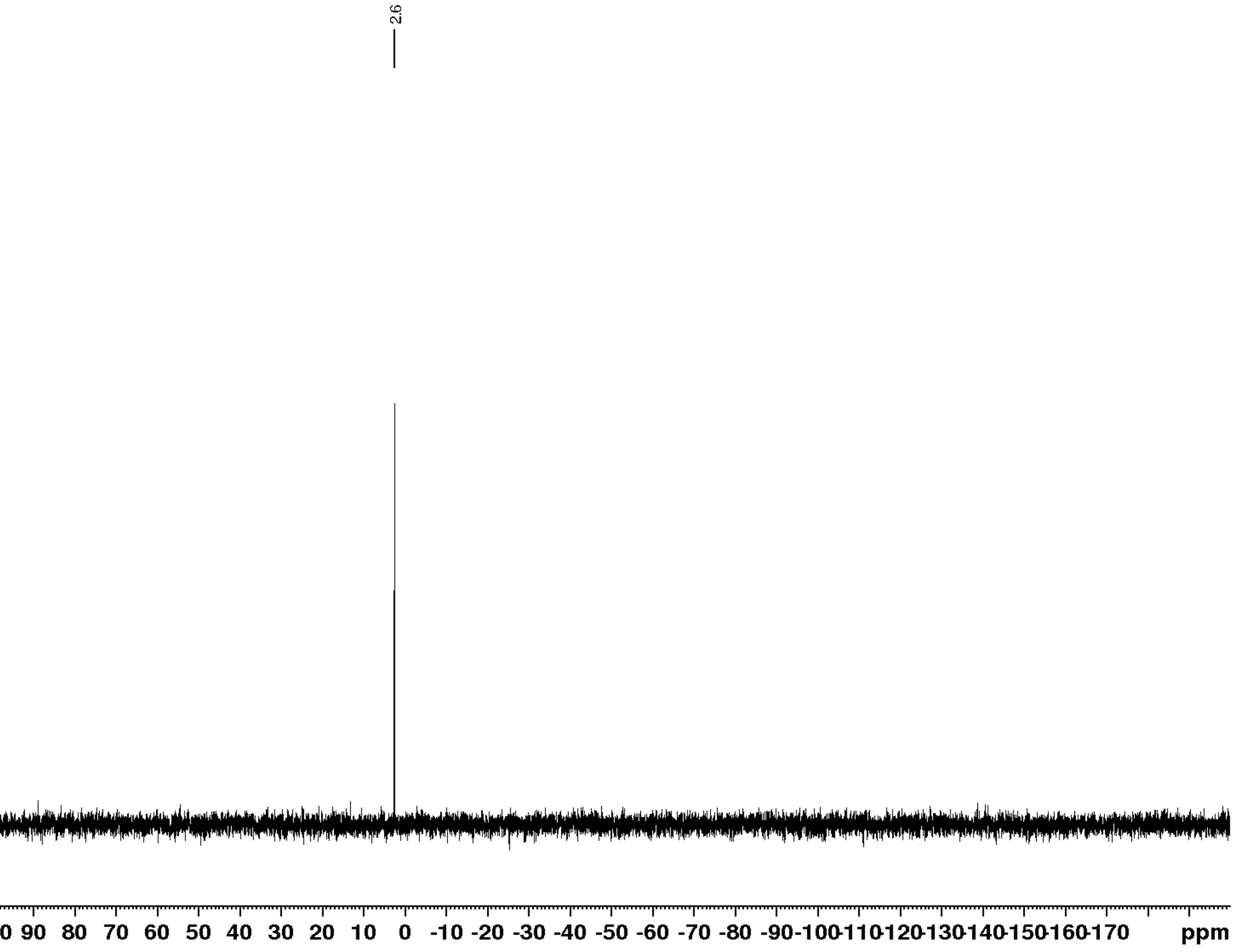

Figure S105. ^{1}H NMR spectrum (400 MHz, $CDCl_3$, 298 K) of **11**

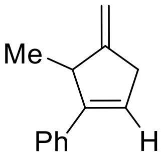

11

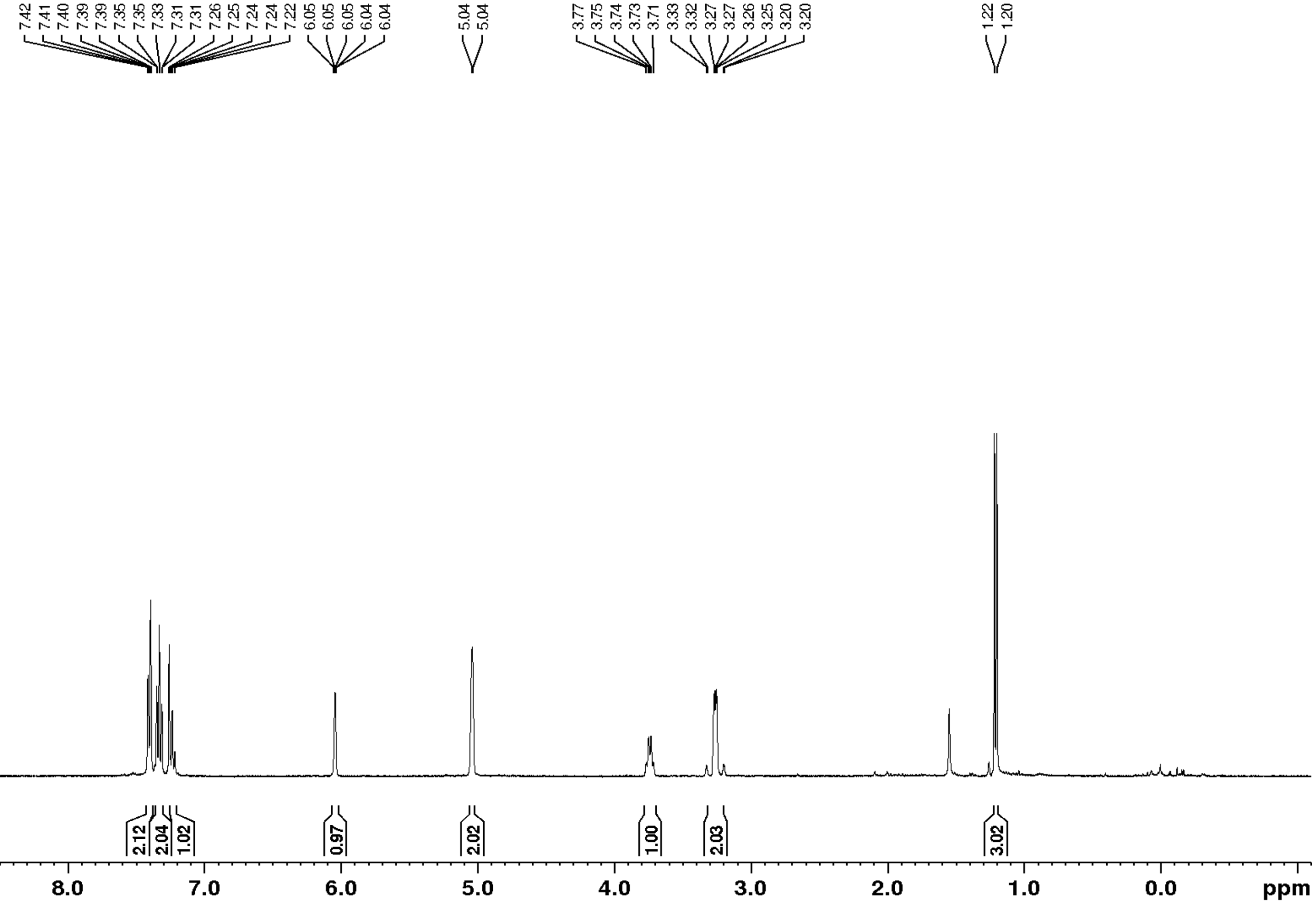

Figure S106. $^{13}C\{^1H\}$ NMR spectrum (101 MHz, $CDCl_3$, 298 K) of **11**

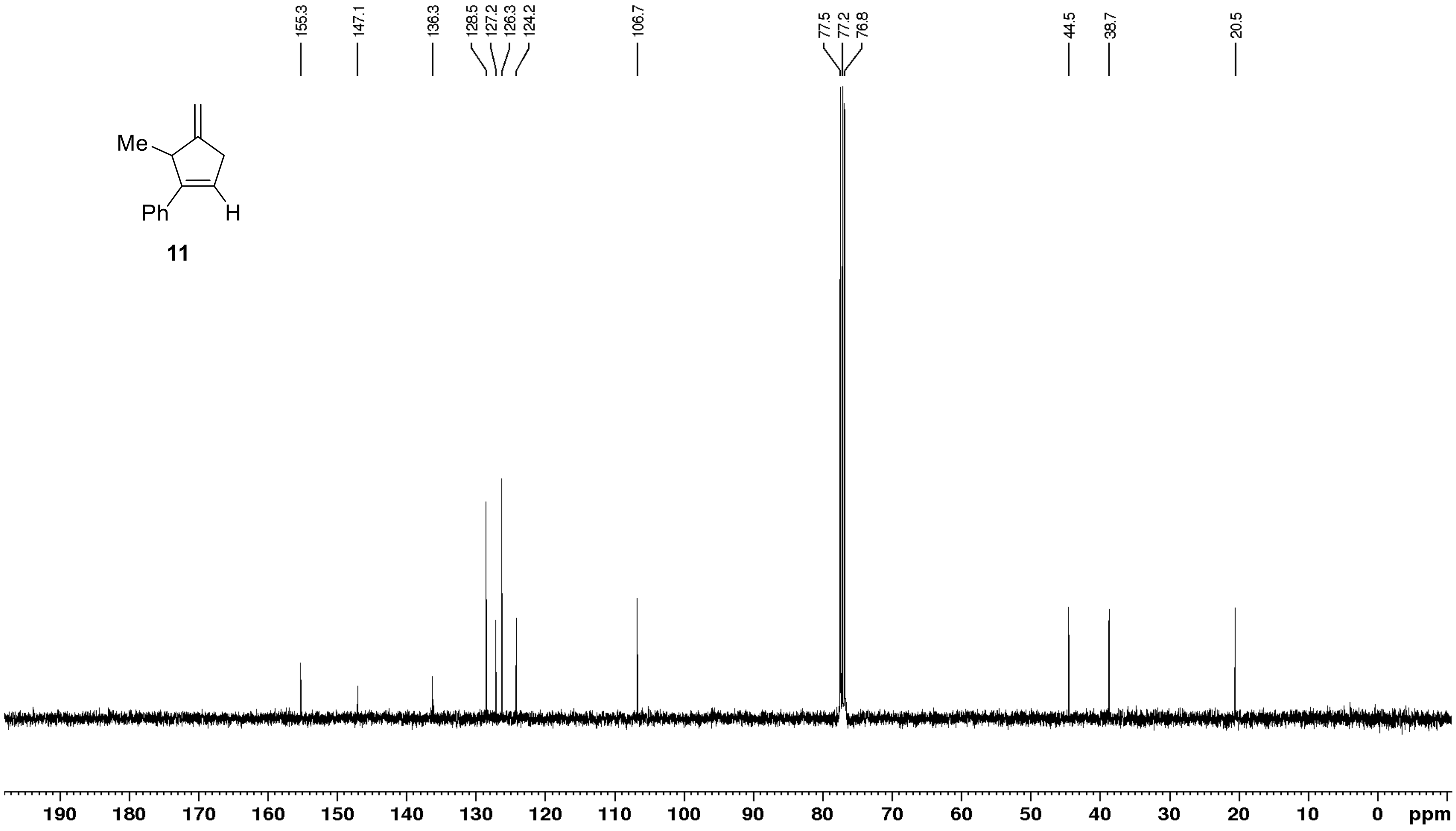

Figure S107. ^{1}H NMR spectrum (500 MHz, $CDCl_3$, 298 K) of **12** (*minor *trans*-diastereomer)

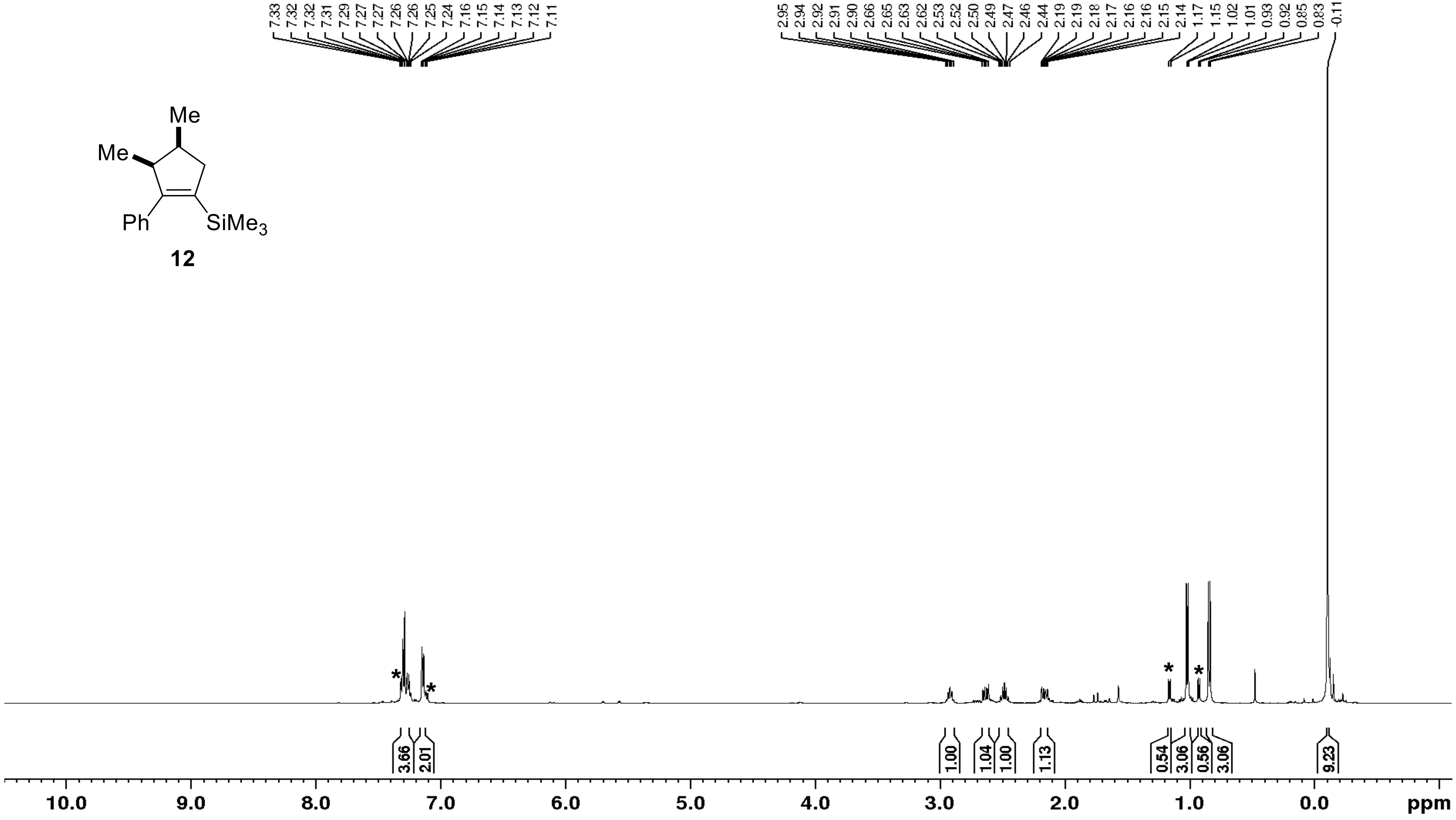

Figure S108. $^{13}C\{^1H\}$ NMR spectrum (101 MHz, $CDCl_3$, 298 K) of **12**

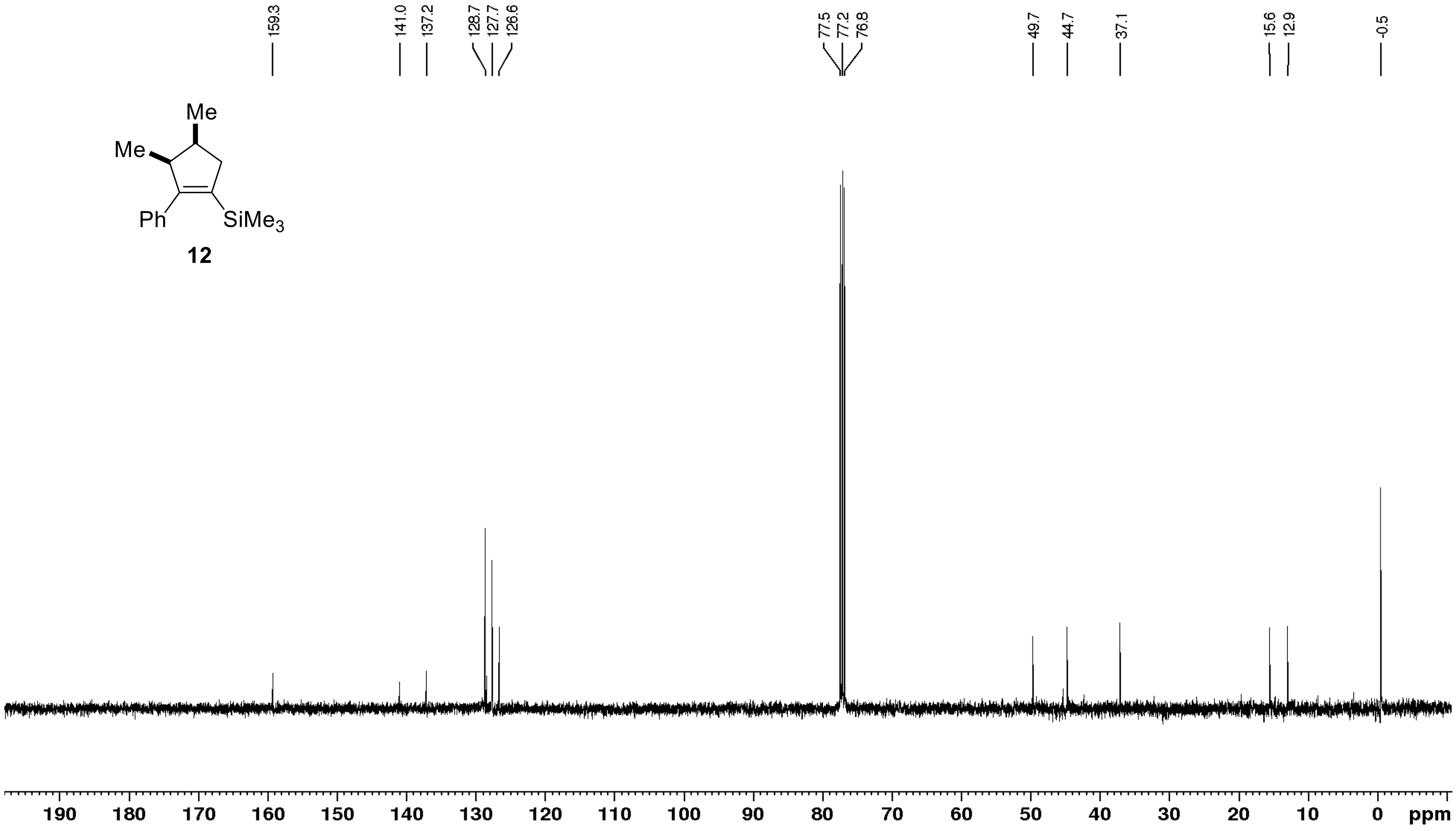

Figure S109. ^{29}Si DEPT NMR spectrum (79 MHz, $CDCl_3$, 298 K, optimized for *J* = 7.0 Hz) of **12**

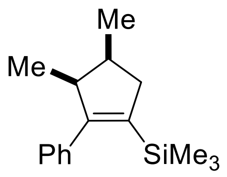

12

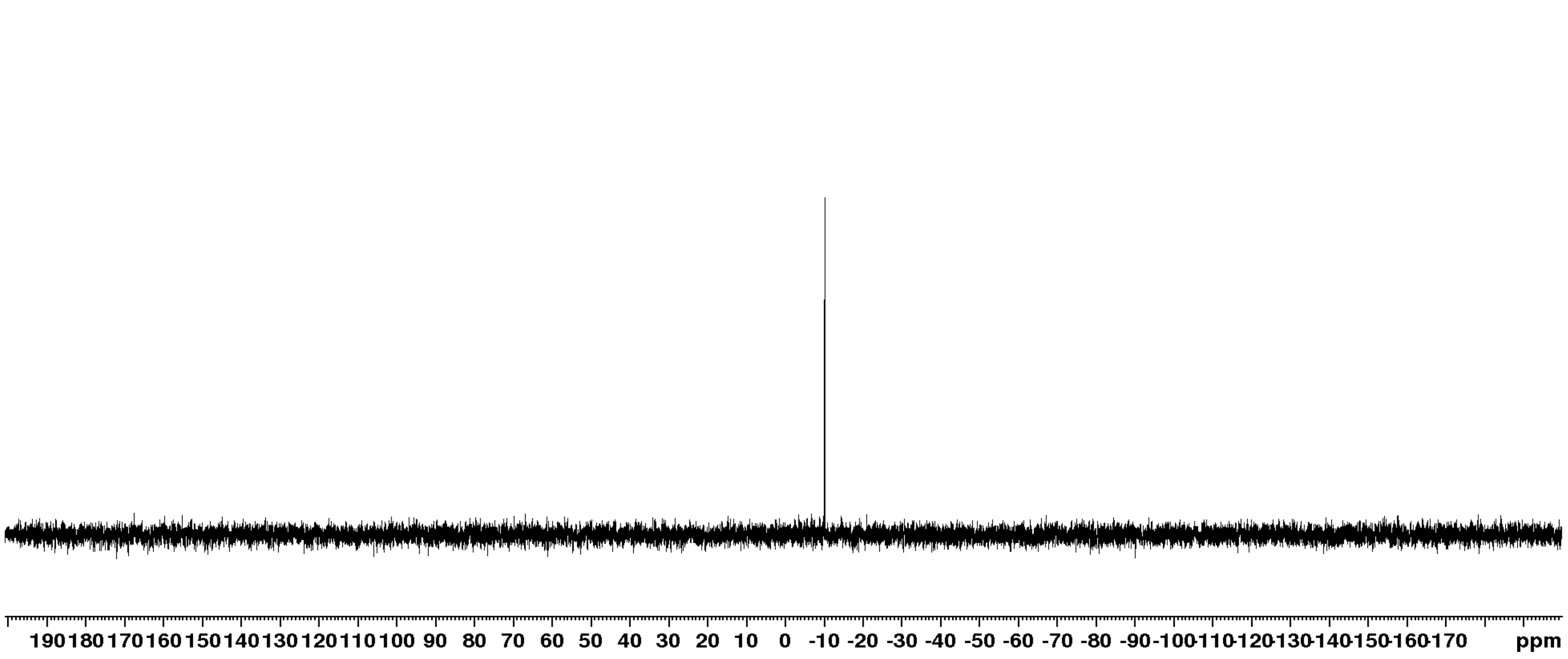

Figure S110. ^{1}H NMR spectrum (400 MHz, $CDCl_3$, 298 K) of **13**

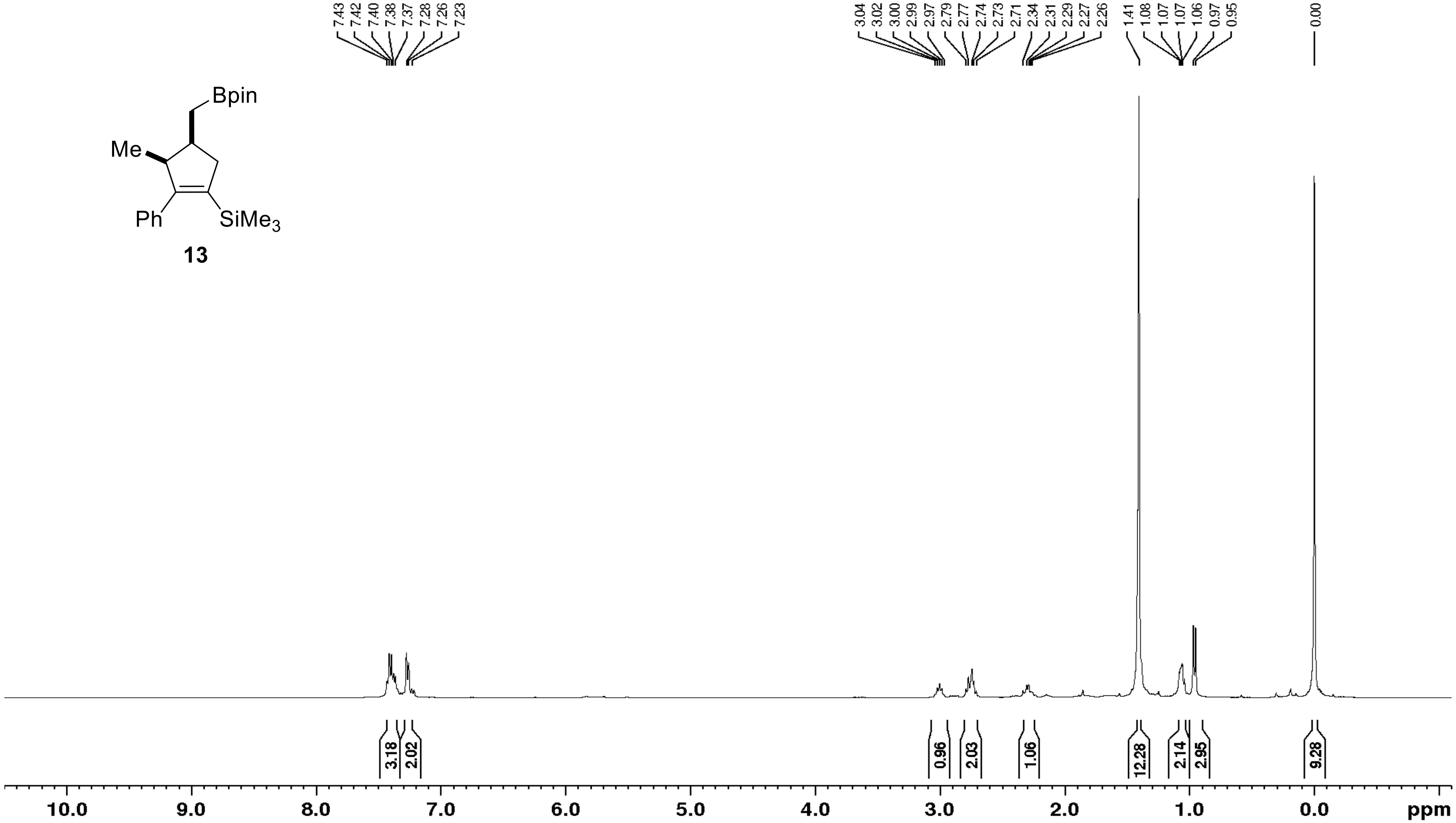

Figure S111. $^{13}C\{^{1}H\}$ NMR spectrum (101 MHz, $CDCl_3$, 298 K) of **13**

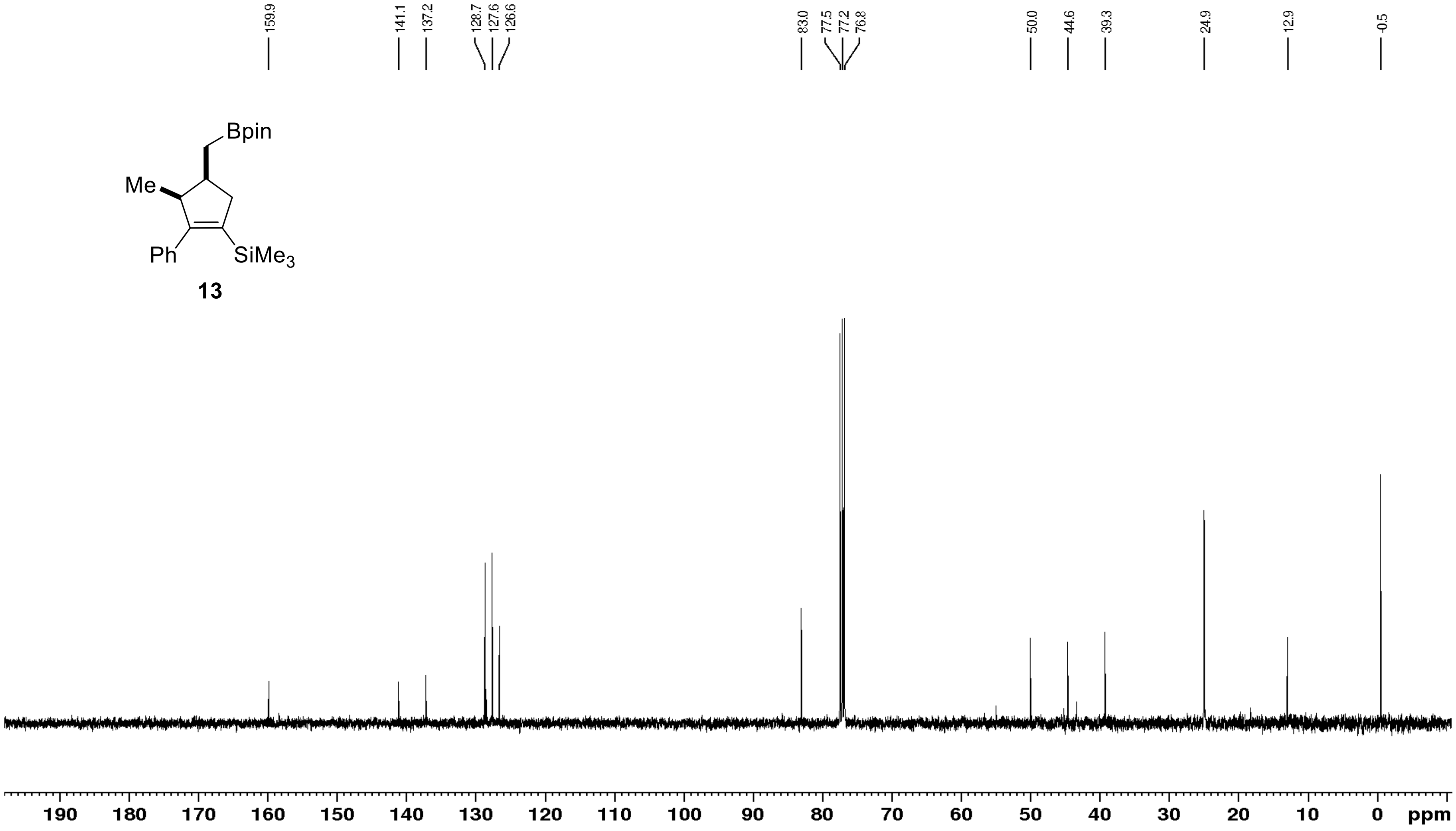

Figure S112. ^{11}B NMR spectrum (128 MHz, $CDCl_3$, 298 K) of **13**

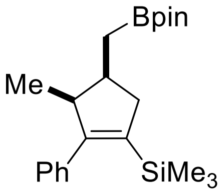

13

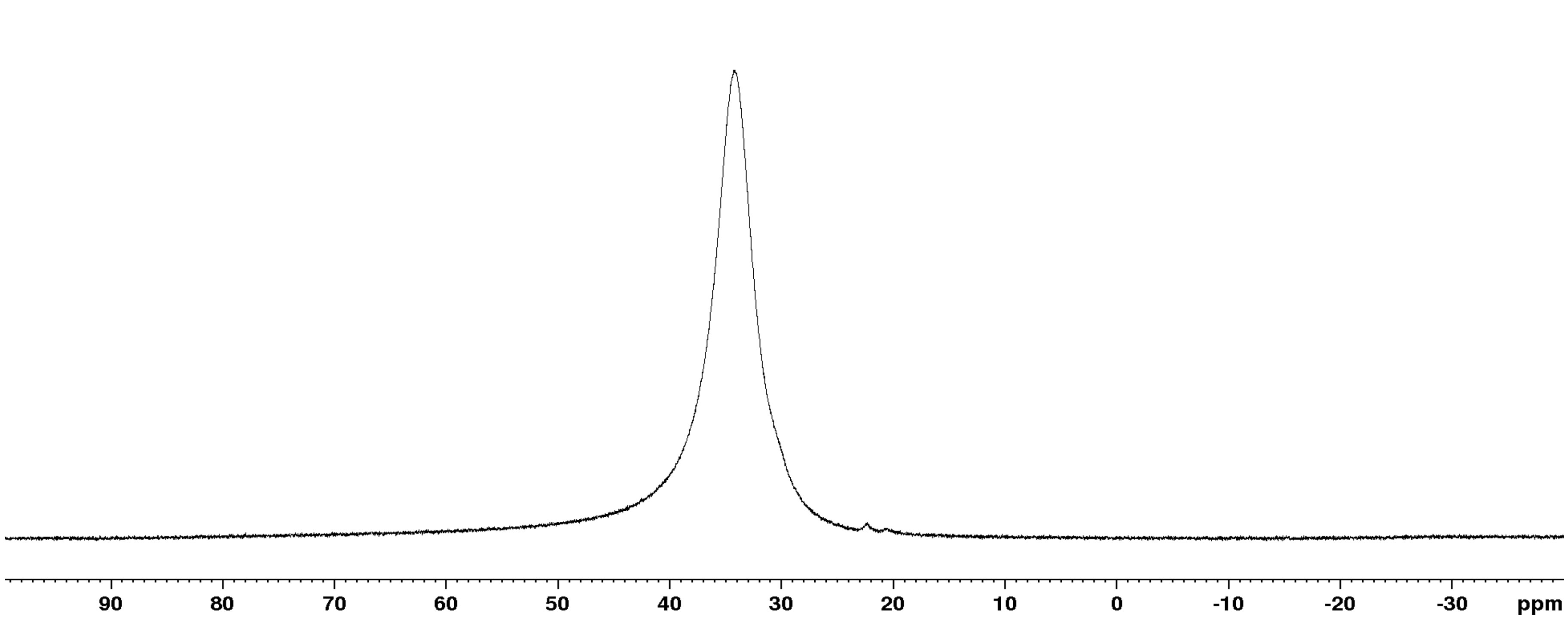

Figure S113. ^{29}Si DEPT NMR spectrum (79 MHz, $CDCl_3$, 298 K, optimized for J = 7.0 Hz) of **13**

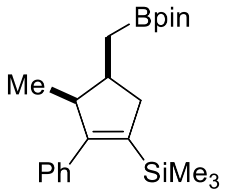

13

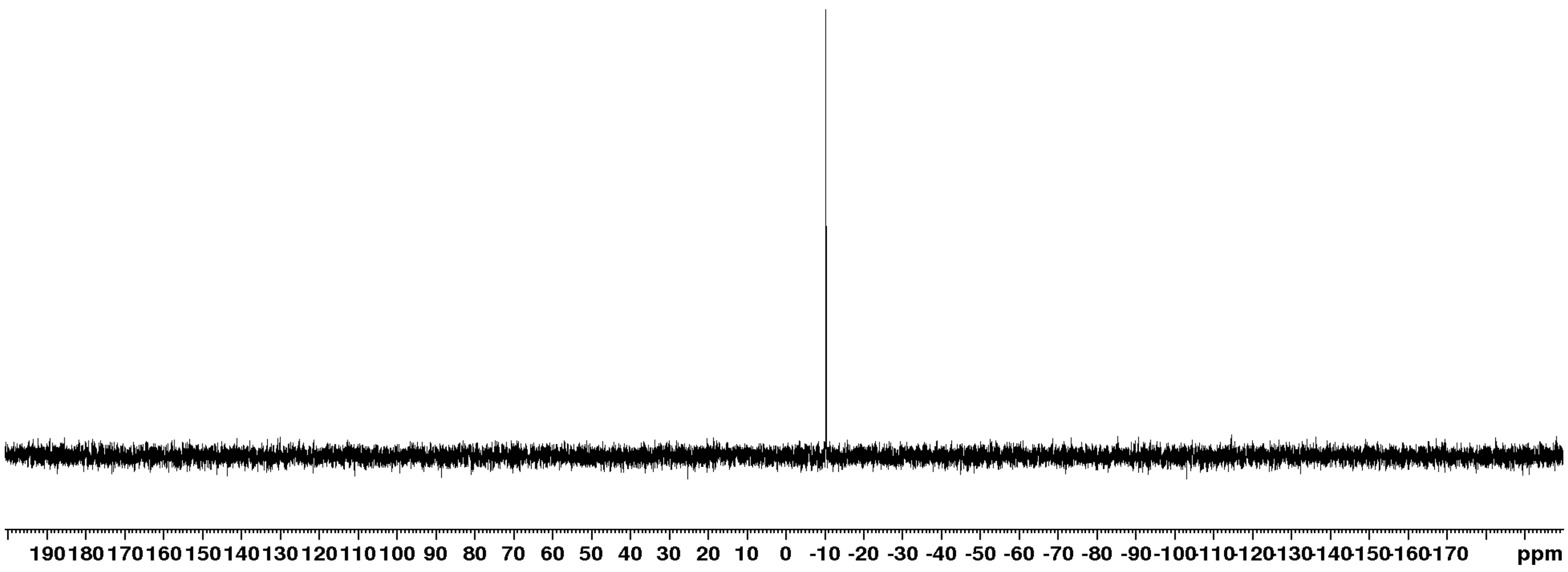

Figure S114. ^{1}H NMR spectrum (500 MHz, $CDCl_3$, 298 K) of **14**

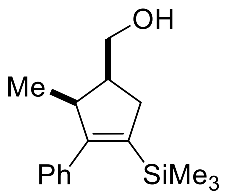

14

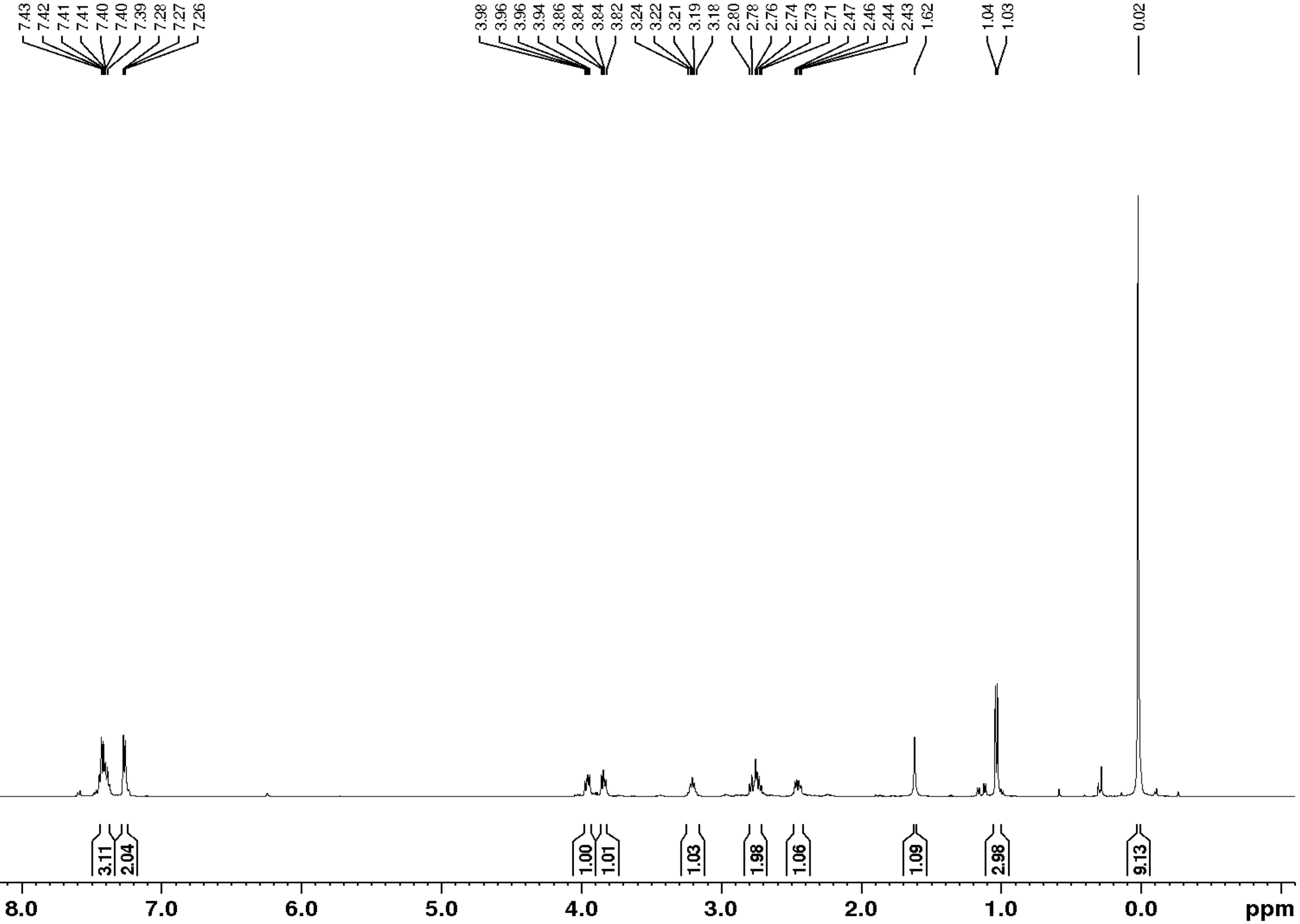

Figure S115. $^{13}C\{^{1}H\}$ NMR spectrum (101 MHz, $CDCl_3$, 298 K) of **14**

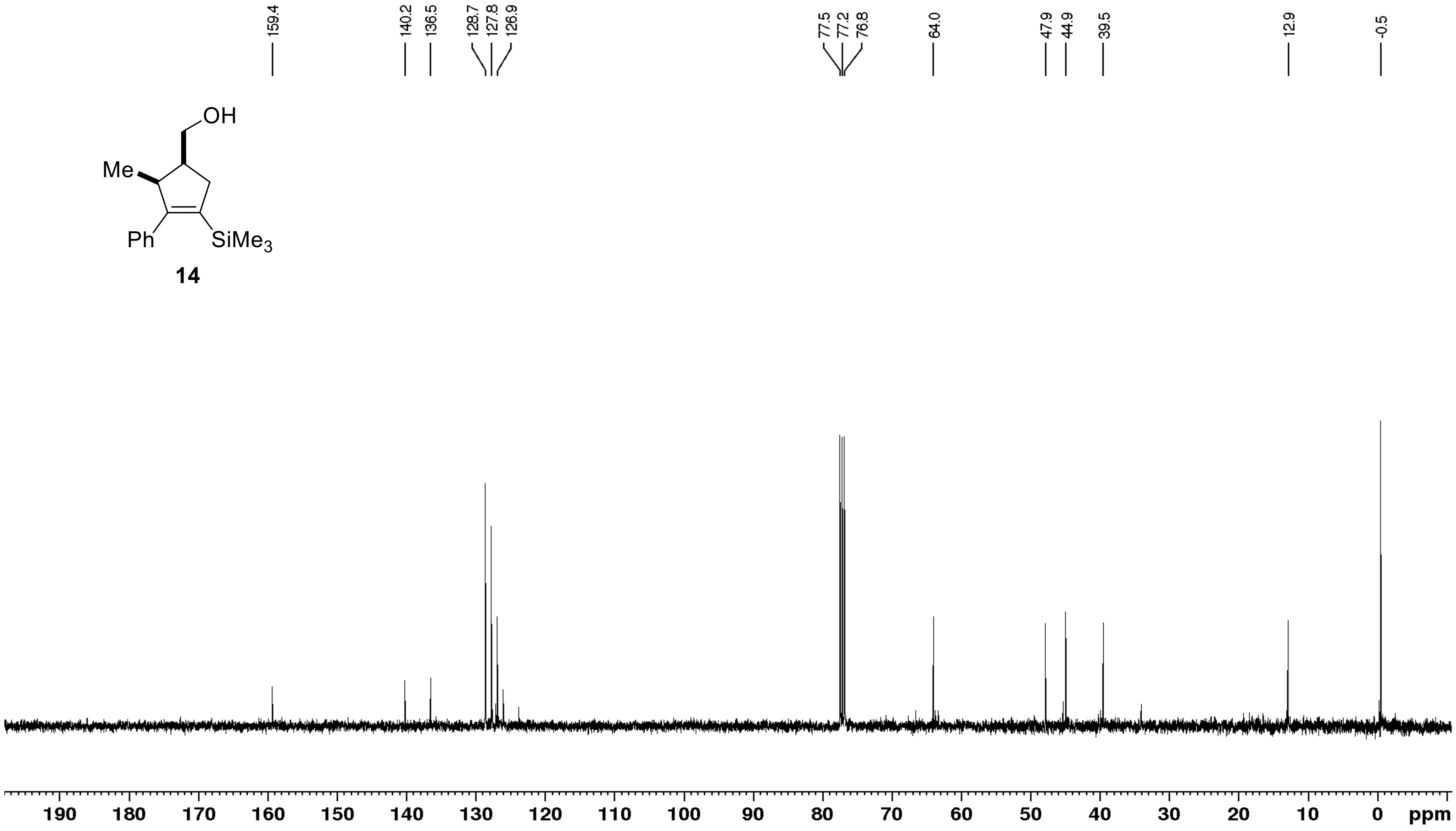

Figure S116. ^{29}Si DEPT NMR spectrum (79 MHz, $CDCl_3$, 298 K, optimized for J = 7.0 Hz) of **14**

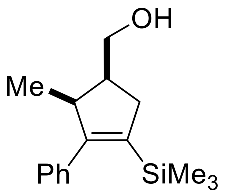

14

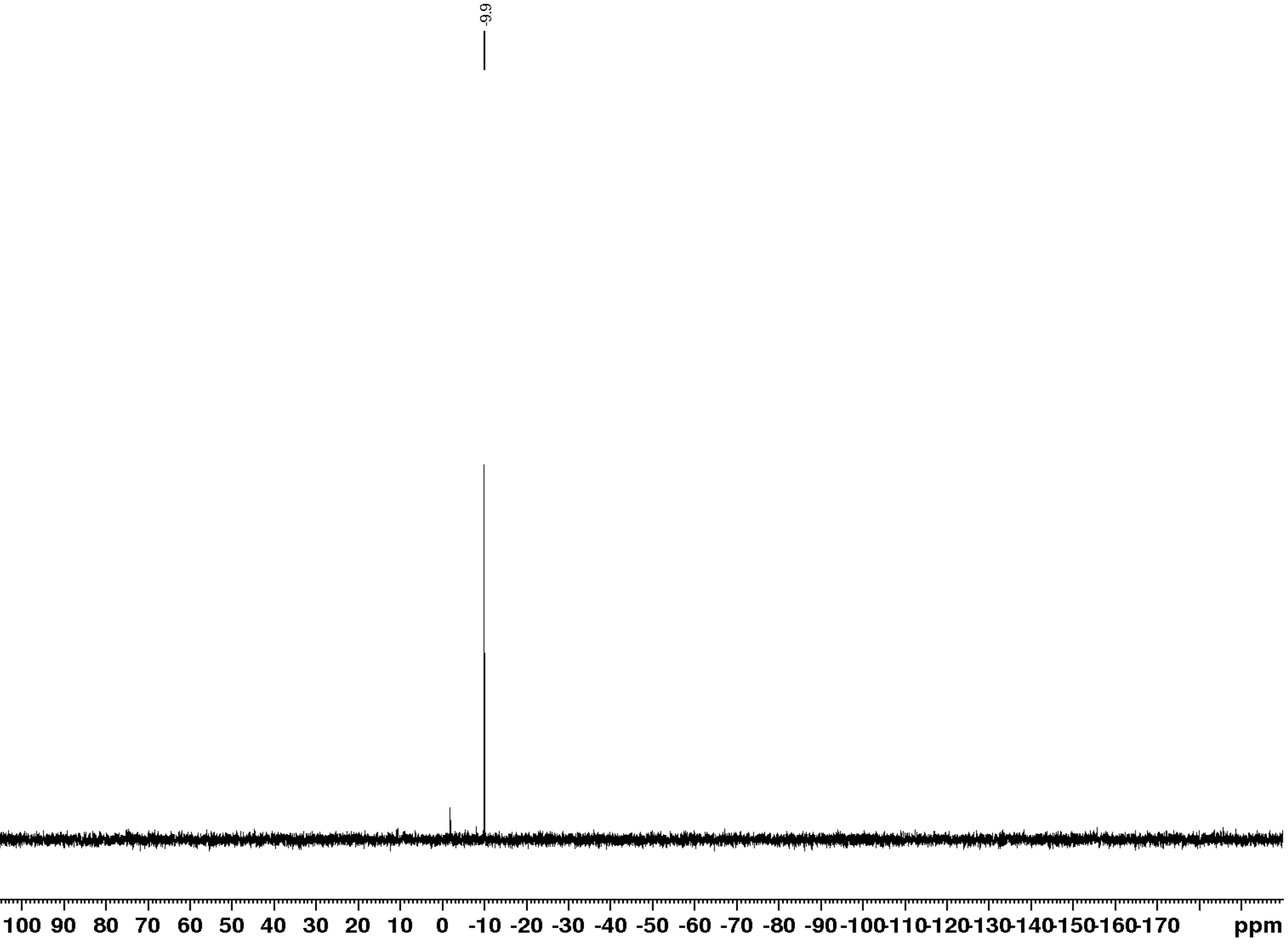

Figure S117. ^{1}H NMR spectrum (400 MHz, $CDCl_3$, 298 K) of **15**

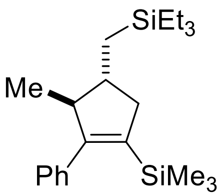

15

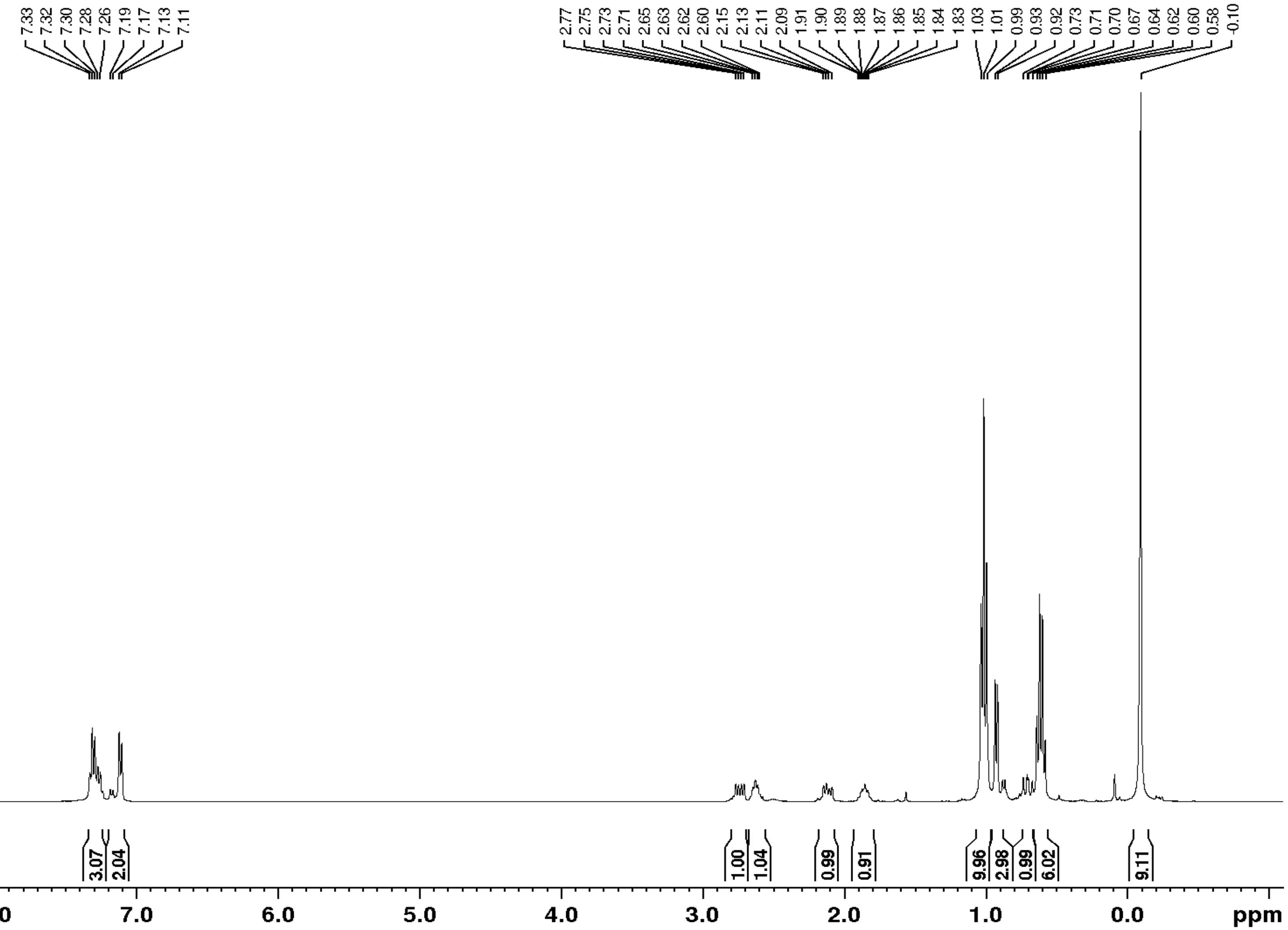

Figure S118. $^{13}C\{^1H\}$ NMR spectrum (101 MHz, $CDCl_3$, 298 K) of **15**

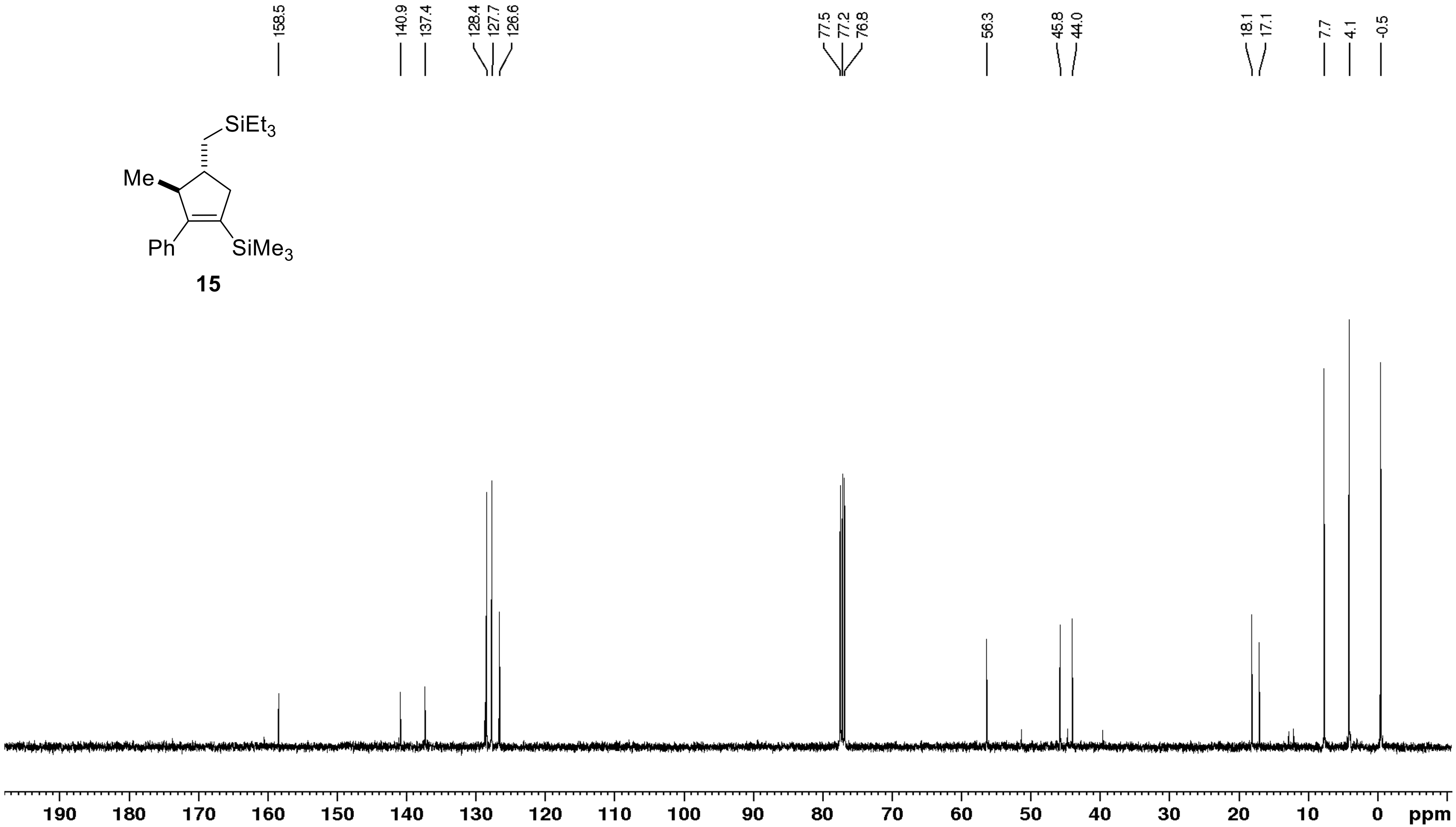

Figure S119. ^{29}Si DEPT NMR spectrum (79 MHz, $CDCl_3$, 298 K, optimized for J = 35.0 Hz) of **15**

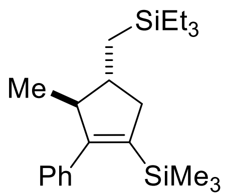

15

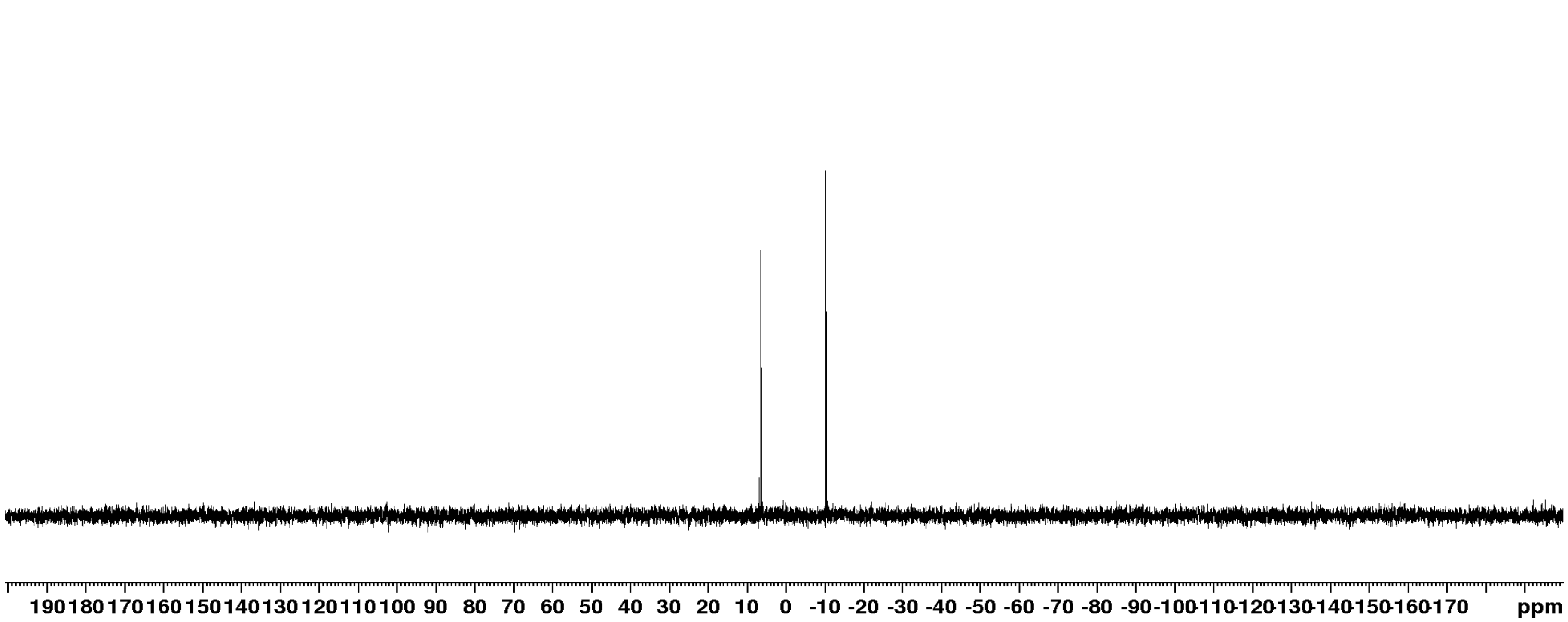

Figure S120. ^{1}H NMR spectrum (500 MHz, $CDCl_3$, 298 K) of **16**

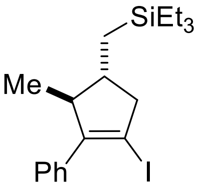

16

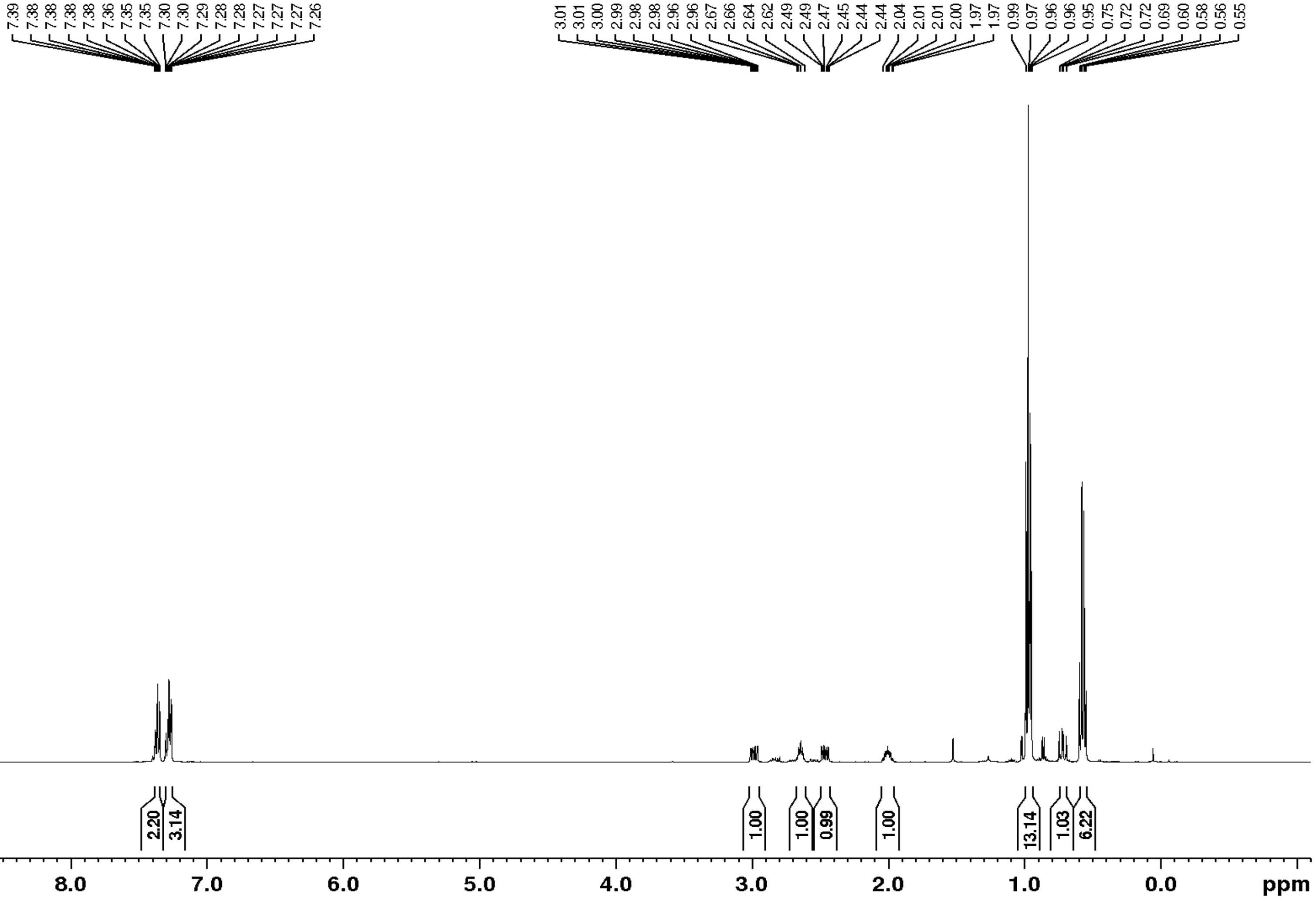

Figure S121. $^{13}C\{^{1}H\}$ NMR spectrum (101 MHz, $CDCl_3$, 298 K) of **16**

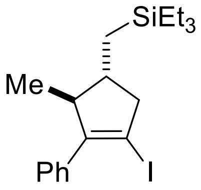

16

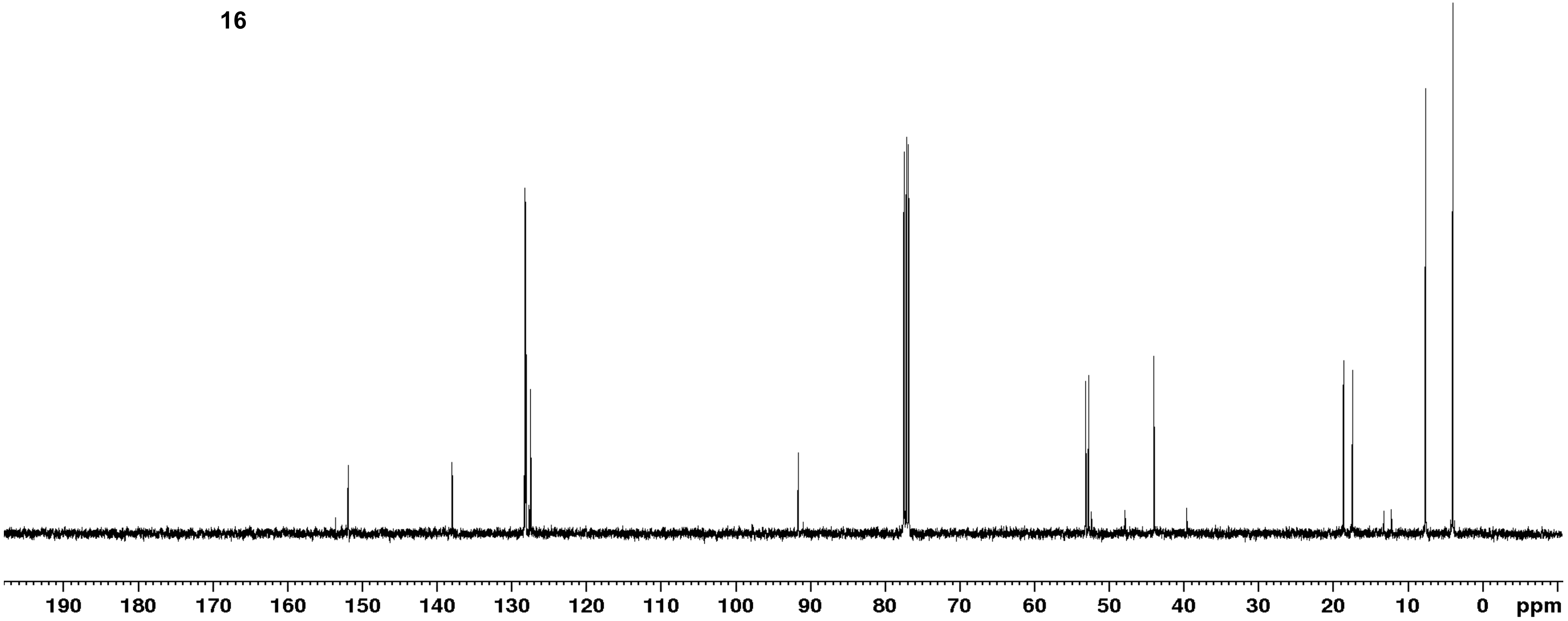

Figure S122. ^{29}Si DEPT NMR spectrum (79 MHz, $CDCl_3$, 298 K, optimized for J = 35.0 Hz) of **16**

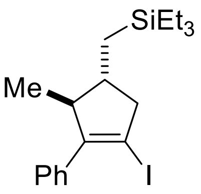

16

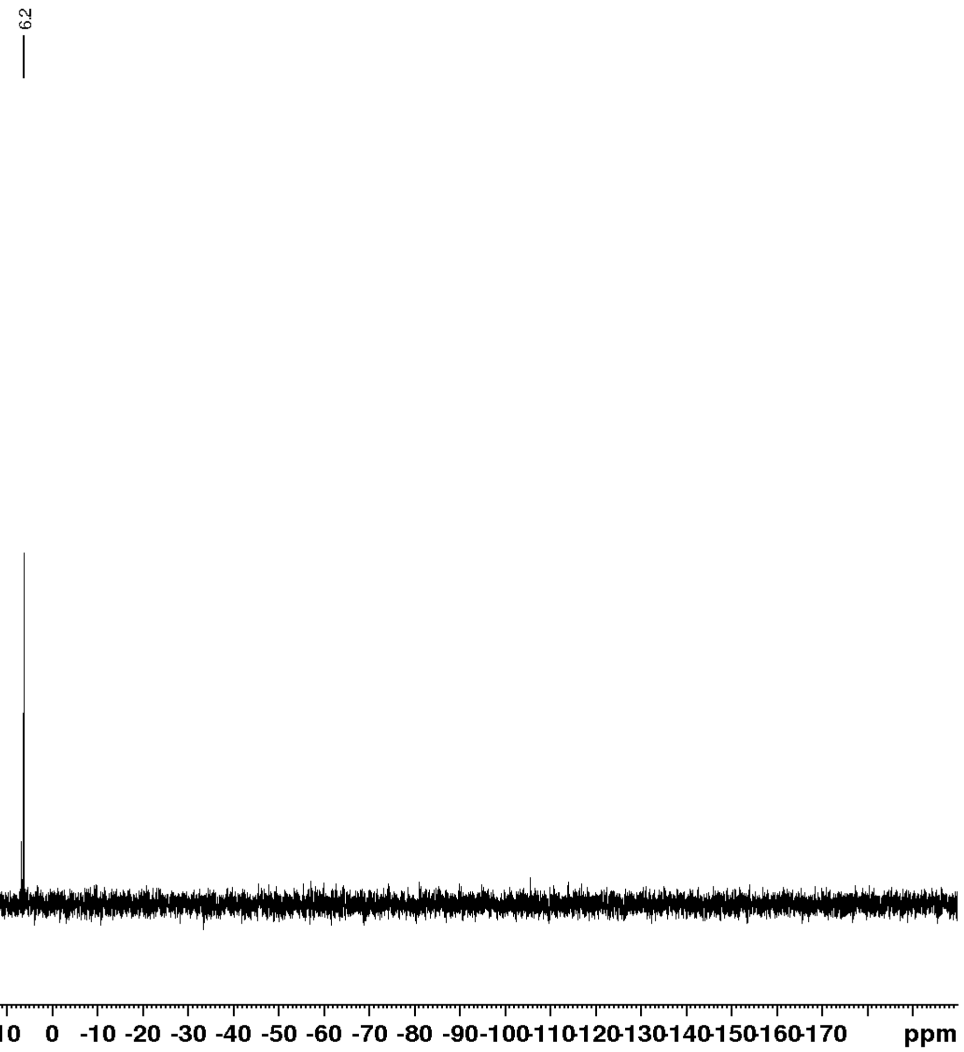

11 Computational Data

Computational Details: The quantum chemical DFT calculations have been performed with the TURBOMOLE 7.4 suite of programs[S19] The structures are fully optimized at the TPSS-D3/def2-TZVP + COSMO level of theory, which combines the TPSS meta-GGA density functional[S20] with the BJ-damped DFT-D3 dispersion correction[S21,S22] and the def2-TZVP basis set,[S23,S24] using the Conductor-like Screening Model (COSMO) continuum solvation model[S25] for benzene solvent (dielectric constant ε = 2.30 and solvent radius R_{solv} = 3.28 Å). The density-fitting RI-J approach[S23,S26,S27] is used to accelerate the geometry optimization and numerical harmonic frequency calculations[S28] in solution. The optimized structures are characterized by frequency analysis to identify the nature of located stationary points (no imaginary frequency for true minima and only one imaginary frequency for transition state) and to provide thermal corrections (at 298.15 K and 1 atm) according to the modified ideal gas–rigid rotor–harmonic oscillator model.[S29] This choice of dispersion-corrected meta-GGA functional makes the efficient exploration of all potential reaction paths possible.

The final solvation free energies in benzene are computed with the COSMO-RS solvation model[S30] (parameter file: BP_TZVP_C30_1601.ctd) using the COSMOtherm program package[S31] on the above TPSS-D3 optimized structures, and corrected by +1.89 kcal·mol^{-1} to account for higher reference solute concentration of 1 mol·L^{-1} usually used in solution. To check the effects of the chosen DFT functional on the reaction energies and barriers, single-point calculations at the meta-GGA TPSS-D3[S20] and hybrid-meta-GGA PW6B95-D3[S32] levels are performed using a larger def2-QZVP basis set.[S14,S33] The final reaction Gibbs free energies (ΔG) are determined from the electronic single-point energies plus TPSS-D3 thermal corrections and COSMO-RS solvation free energies. In our discussion, higher-level PW6B95-D3 Gibbs free energies (in kcal mol^{-1}, at 298.15 K and 1 mol L^{-1} standard state concentration) will be used unless specified otherwise.

Meta-GGA functionals like TPSS tend to underestimate reaction barriers that can be evidently improved by using hybrid meta-GGA functionals like PW6B95. The applied DFT methods in combination with the large AO basis set provide usually accurate electronic energies leading to errors for chemical energies (including barriers) on the order of typically 1–2 kcal mol^{-1}. This has been tested thoroughly for the huge data base GMTKN55[S34] which is the common standard in the field of DFT benchmarking.

Table S2. TPSS-D3/def2-TZVP + COSMO computed lowest imaginary frequency (ImF), zero-point energies (ZPE), gas-phase enthalpic (Hc) and Gibbs free-energy (Gc) corrections; the COSMO-RS computed solvation enthalpic (Hsol) and Gibbs free-energy (Gsol) corrections in benzene solution; TPSS-D3/def2-QZVP and PW6B95-D3/def2-QZVP single-point energies (TPSS-D3 and PW6B95-D3); the relative electronic energies (ΔE_T and ΔE_P) and Gibbs free-energies (ΔG_T and ΔG_P) at the TPSS-D3 and PW6B95-D3 levels. Each structure is labeled either by its molecular formula or a specific name in bold. Transition structures (with only one imaginary frequency) are indicated by the "**TS**" prefix.

Reactions in benzene	ImF cm^{-1}	ZPE kcal/mol	Hc kca/mol	Gc kcal/mol	Hsol kcal/mol	Gsol kcal/mol	TPSS-D3 E_h	PW6B95-D3 E_h	G_P E_h	ΔE_T kcal/mol	ΔE_P kcal/mol	*ΔG_P kcal/mol*	ΔG_T kcal/mol
***A^+* catalyzed allenylsilane *1a* isomerization is kinetically facile**													
**1a** + **A^+**	0	299.84	322.21	247.45	-54.19	-45.25	-1538.98469	-1540.39506	-1540.06681	0.00	0.00	*0.00*	0.00
**$TS0^+$**	118i	300.92	323.55	262.49	-45.98	-39.11	-1538.98694	-1540.39670	-1540.03771	-1.41	-1.03	*18.26*	17.88
**A^+** + **4a**	0	300.18	322.54	247.95	-54.77	-45.67	-1538.98023	-1540.39364	-1540.06526	2.80	0.89	*0.97*	2.88
*$SiMe_3^+$ transfer from **A^+** to alkyne **2a** is kinetically facile but 5.3 kcal/mol endergonic*													
**A^+** + **2a**	0	270.28	289.82	220.73	-57.45	-48.29	-1322.03626	-1323.30339	-1323.02257	0.00	0.00	*0.00*	0.00
**$TS1^+$**	124i	271.37	291.04	235.80	-47.96	-41.00	-1322.03715	-1323.30393	-1322.99049	-0.56	-0.34	*20.13*	19.92
**B^+** + **4a**	0	269.99	289.63	220.11	-55.33	-46.34	-1322.03400	-1323.29711	-1323.01416	1.42	3.94	*5.28*	2.76
*...followed by C..C addition between **B^+** and **4a***													
**$TS2^+$**	82i	271.02	290.51	236.24	-47.14	-40.61	-1322.04812	-1323.30795	-1322.99318	-7.44	-2.86	*18.44*	13.86
**C^+**	0	272.59	291.61	238.49	-48.80	-42.22	-1322.07594	-1323.33919	-1323.02341	-24.90	-22.47	*-0.52*	-2.96
...and 1,5-H-shift													
**$TS3^+$**	107i	272.09	290.27	238.85	-49.05	-42.18	-1322.06609	-1323.32623	-1323.00981	-18.72	-14.33	*8.01*	3.62
**D^+**	0	273.82	292.37	239.95	-47.44	-40.81	-1322.11958	-1323.38255	-1323.06220	-52.29	-49.67	*-24.87*	-27.48
...and 1,5-cyclization													
**$TS4^+$**	341i	273.50	291.39	240.43	-48.49	-41.76	-1322.10259	-1323.36461	-1323.04499	-41.62	-38.41	*-14.07*	-17.27
**E^+**	0	274.64	292.57	241.61	-49.74	-43.14	-1322.13063	-1323.39893	-1323.07964	-59.22	-59.96	*-35.81*	-35.07
...and 1,2-H-shift													
**$TS5^+$**	717i	272.09	290.11	238.47	-51.00	-44.09	-1322.10295	-1323.37260	-1323.05984	-41.85	-43.43	*-23.39*	-21.80
**F^+**	0	274.46	292.54	241.22	-51.88	-44.88	-1322.11902	-1323.38875	-1323.07285	-51.94	-53.57	*-31.55*	-29.92
*Finally, the intermolecular $SiMe_3^+$ transfer from **F^+** to propargylsilane **4a** leads to annulation product **3aa** and regenerated cation **A^+***													
**4a** + **F^+**	0	389.49	416.17	333.01	-60.44	-49.75	-1887.01162	-1888.81114	-1888.35370	0.00	0.00	*-31.55*	0.00
**$TS6^+$**	123i	390.45	417.43	348.08	-52.70	-44.19	-1887.02288	-1888.82020	-1888.33291	-7.07	-5.68	*-18.50*	11.66
**A^+** + **3aa**	0	389.61	416.35	332.26	-62.10	-51.54	-1887.02076	-1888.81675	-1888.36337	-5.73	-3.52	*-37.62*	-8.28

**F**$^+$ + **1a**	0	389.15	415.84	332.52	-59.85	-49.33	-1887.01609	-1888.81256	-1888.35525	0.00	0.00	*-31.55*	0.00
**TS6a**$^+$	137i	390.47	417.33	348.20	-52.42	-43.91	-1887.02461	-1888.81835	-1888.33043	-5.35	-3.63	*-15.97*	13.86
**A**$^+$ + **3aa**	0	389.61	416.35	332.26	-62.10	-51.54	-1887.02076	-1888.81675	-1888.36337	-2.93	-2.63	*-36.65*	-5.40
Computed Me_3Si^+ binding free energies													
**1a** + $SiMe_3^+$	0	182.44	196.39	140.53	-63.14	-54.68	-974.00746	-974.89432	-974.75149	0.00	0.00	*0.00*	0.00
**4a** + $SiMe_3^+$	0	182.77	196.72	141.03	-63.73	-55.10	-974.00299	-974.89290	-974.74994	2.80	0.89	*0.97*	2.88
**A**$^+$	0	185.15	198.91	156.16	-46.21	-40.80	-974.08763	-974.97125	-974.78441	-50.31	-48.27	*-20.66*	-22.69
**2a** + $SiMe_3^+$	0	152.88	163.99	113.80	-66.41	-57.72	-757.05902	-757.80265	-757.70725	0.00	0.00	*0.00*	0.00
**B**$^+$	0	155.30	166.33	128.82	-47.36	-41.89	-757.13694	-757.87330	-757.73175	-48.89	-44.33	*-15.38*	-19.94
**3aa** + $SiMe_3^+$	0	272.20	290.53	225.34	-71.06	-60.98	-1322.04352	-1323.31601	-1323.04805	0.00	0.00	*0.00*	0.00
**F**$^+$	0	274.46	292.54	241.22	-51.88	-44.88	-1322.11902	-1323.38875	-1323.07285	-47.38	-45.65	*-15.56*	-17.29
**cb**$^-$ + $SiMe_3^+$	0	144.01	161.24	96.48	-115.99	-102.16	-16169.75395	-16177.85434	-16177.85736	0.00	0.00	*0.00*	0.00
**Scb**	0	145.99	163.31	111.96	-34.48	-25.21	-16169.92076	-16178.01837	-16177.87711	-104.68	-102.93	*-12.39*	-14.14
Very facile $SiMe_3^+$ transfer													
**1a** + **Scb**	0	260.69	286.61	203.25	-42.45	-29.66	-16734.81782	-16743.44218	-16743.15951	0.00	0.00	*0.00*	0.00
**TS7**	28i	262.00	287.94	219.31	-44.80	-33.52	-16734.82088	-16743.44230	-16743.14320	-1.92	-0.08	*10.23*	8.39
**A**$^+$ + **cb**$^-$	0	261.42	287.07	203.40	-107.03	-92.73	-16734.73118	-16743.35508	-16743.17269	54.37	54.66	*-8.27*	-8.55
**TS8**	25i	261.96	287.98	219.25	-42.03	-30.95	-16734.82260	-16743.44833	-16743.14523	-3.00	-3.86	*8.96*	9.82
**4a** + **Scb**	0	261.02	286.94	203.75	-43.03	-30.07	-16734.81335	-16743.44076	-16743.15796	2.80	0.89	*0.97*	2.88

Table S3. The TPSS-D3/def2-TZVP + COSMO optimized atomic Cartesian coordinates (in Å) in benzene solution. Each structure is labeled by the specific name (see also **Table S2**), followed by the number of atoms, the total energy, and the detailed atomic coordinates (in double-column text list)

A^+ : $(Me_3Si)MeCCCH_2SiMe_3^+$ cation
35
Energy = -974.0680803321

C	1.0401713	1.0951335	-0.0080765
H	1.1042226	1.6898410	-0.9246055
H	1.1064402	1.7010524	0.9009231
C	0.0371760	0.1405908	-0.0011255
C	-0.8892732	-0.7179395	0.0051113
C	-2.2306462	2.1543482	-0.0341604
C	-3.4516788	-0.2651329	-1.5476919
C	-3.4262634	-0.2083185	1.5852493
H	-1.6732125	2.4894539	0.8467132
H	-1.6839735	2.4559525	-0.9337192
H	-3.1888245	2.6885106	-0.0382823
H	-3.6555537	-1.3402651	-1.5262517
H	-4.4142022	0.2554215	-1.6300836
H	-2.8681513	-0.0367124	-2.4455710
H	-4.3899929	0.3108937	1.6618625
H	-3.6253349	-1.2842637	1.6092013
H	-2.8305238	0.0574515	2.4646348
Si	-2.5863839	0.3208247	0.0018289
C	-0.9912058	-2.2215681	0.0120244
H	-1.5645645	-2.5493477	0.8828616
H	-1.5168259	-2.5579892	-0.8854412
H	-0.0009289	-2.6777077	0.0410383
C	2.7504771	-1.0034302	-1.5578023
H	1.9558746	-1.7572024	-1.5512246
H	2.6272803	-0.3834809	-2.4526685
H	3.7069179	-1.5319624	-1.6499509
Si	2.7561612	0.0304821	-0.0034748
C	4.0477289	1.3788107	-0.0166088
C	2.7567076	-0.9788660	1.5669032
H	5.0426401	0.9163124	-0.0153071
H	3.9698067	2.0060839	-0.9107584
H	3.9737619	2.0196957	0.8681697
H	2.6371888	-0.3448699	2.4523766
H	1.9620005	-1.7325130	1.5755252
H	3.7134709	-1.5060184	1.6635400

B^+ : $(Me_3Si)MeCCPh^+$ cation
30
Energy = -757.1277345603

C	-0.1264136	0.9282142	0.0008261
C	1.0985317	1.2213584	0.0242609
C	-1.4348144	0.4677012	-0.0240978
C	-2.1230456	0.2242627	1.1950349
C	-2.0846682	0.2468790	-1.2682988
C	-3.4285375	-0.2333673	1.1598395
H	-1.6141067	0.4018782	2.1363536
C	-3.3904813	-0.2109681	-1.2821722
H	-1.5467634	0.4416538	-2.1899118
C	-4.0580362	-0.4507774	-0.0733669
H	-3.9639730	-0.4232078	2.0838985
H	-3.8967633	-0.3838315	-2.2257867
H	-5.0823558	-0.8104030	-0.0926349
C	1.9561758	2.4570996	0.0470155
H	1.3282118	3.3479421	0.0927655
H	2.6204109	2.4336428	0.9144329
H	2.5738906	2.4921908	-0.8541060
C	3.1228435	-0.4413837	-1.4954636
H	2.5245563	-0.3306794	-2.4056876
H	3.8370374	0.3857564	-1.4409984
H	3.6951330	-1.3741109	-1.5798167
Si	2.0455778	-0.5472242	0.0276141
C	2.9876139	-0.5134743	1.6406869
C	0.8358569	-1.9658763	-0.0569854
H	2.3117832	-0.4535209	2.4999647
H	3.5608103	-1.4454561	1.7272515
H	3.6950092	0.3197436	1.6912682
H	1.4128672	-2.8990739	-0.0597738
H	0.1638886	-1.9968194	0.8064487
H	0.2328078	-1.9487407	-0.9703431

C⁺ : $(Me_3Si)MeCC(Ph)C(Me)CCH_2SiMe_3^+$ cation
52
Energy = -1322.035199914

C	-3.0244500	-2.0382702	-0.3527580
C	-3.3254406	1.4287117	-1.0960863
C	-2.2748000	-1.2524432	-1.0444531
C	-3.8136105	-2.8202298	0.4371871
C	-1.9687562	1.1470583	-0.4861698
H	-3.8578277	2.1740642	-0.4925917
H	-3.2305781	1.8622120	-2.0984236
C	-1.5210624	-0.1226430	-0.3354815
H	-3.9555924	0.5366156	-1.1591837
C	-2.1230140	-1.3794751	-2.5491438
H	-4.8934716	-2.6820817	0.3263041
H	-3.4961601	-3.8620128	0.5519176
Si	-0.9526860	2.7356781	-0.1358872
C	-0.3430550	-0.5568929	0.4398875
H	-2.7144481	-2.2016777	-2.9544967
H	-2.4388198	-0.4374786	-3.0067610
H	-1.0663390	-1.5343997	-2.7867012
C	-1.4207817	3.9224571	-1.5228055
C	-1.5044603	3.4906050	1.5005708
C	0.9058753	2.4708983	-0.2167196
C	0.4757692	-1.6148241	0.0083718
C	-0.0555783	0.0500852	1.6727727
H	-1.1862519	3.5063285	-2.5096463
H	-2.4818842	4.1925775	-1.5098195
H	-0.8429940	4.8478641	-1.4100030
H	-1.1812256	2.9126879	2.3725979
H	-1.0794983	4.4972875	1.5973375
H	-2.5955102	3.5881686	1.5444410
H	1.3826206	3.4452566	-0.3818049
H	1.3317918	2.0376738	0.6908129
H	1.1758398	1.8233742	-1.0587073
C	1.5665701	-2.0244235	0.7699817
H	0.2663574	-2.1150003	-0.9326197
C	1.0348564	-0.3594917	2.4357774
H	-0.7117433	0.8315796	2.0413074
C	1.8524423	-1.3963334	1.9851430
H	2.1961398	-2.8349315	0.4155172
H	1.2372868	0.1211399	3.3882676
H	2.6993646	-1.7223961	2.5808952
Si	-3.5261359	-2.2099210	2.3933206
C	-4.9302651	-3.1384217	3.2009830
C	-1.8491152	-2.8561978	2.8719544
C	-3.7526734	-0.3640930	2.4628566
H	-4.7478657	-0.0754285	2.1079447
H	-3.6631137	-0.0376333	3.5064332
H	-3.0036991	0.1717855	1.8738539
H	-4.8537702	-4.2182325	3.0379642
H	-4.8827311	-2.9572560	4.2826866
H	-5.9070496	-2.7948157	2.8458377
H	-1.6545358	-2.5937437	3.9193421
H	-1.8148193	-3.9480835	2.7907673
H	-1.0450920	-2.4317494	2.2655155

D⁺ :
$CH_2C(SiMe_3)C(Ph)C(Me)CHCH_2SiMe_3^+$
52
Energy = -1322.077312676

C	-3.0389784	-0.9119437	0.0444967
C	-2.0320729	1.1569508	2.0397480
C	-1.7240754	-1.3021632	0.3063696
C	-4.1626829	-1.7861205	-0.1463089
C	-1.1909368	1.0388432	0.9884439
H	-2.3358083	0.2930953	2.6288518
H	-2.3800900	2.1255684	2.3909230
C	-0.7656797	-0.2926583	0.5671436
H	-3.2326867	0.1542838	-0.0297815
C	-1.3660420	-2.7707797	0.2764910
H	-4.8995830	-1.3836363	-0.8491017
H	-3.9157333	-2.8254613	-0.3743802
Si	-0.5932779	2.6164935	0.0567091
C	0.6688886	-0.5127122	0.4277353
H	-2.0737482	-3.3299783	0.8964246
H	-1.4229471	-3.1846155	-0.7363535
H	-0.3643015	-2.9469556	0.6682975
C	-2.1787418	3.5435904	-0.3476202
C	0.5060984	3.6425236	1.1785636
C	0.2949713	2.1264592	-1.5206048
C	1.1988328	-1.3176065	-0.6027023
C	1.5571278	0.1539810	1.2980139
H	-2.8394950	2.9481840	-0.9881692
H	-2.7362147	3.8168240	0.5551894
H	-1.9421089	4.4704164	-0.8838372
H	1.4576381	3.1422428	1.3864325
H	0.7332944	4.6020366	0.6983126

H	0.0149126	3.8546230	2.1349996
H	0.4635435	3.0216988	-2.1312669
H	1.2699262	1.6693400	-1.3254207
H	-0.2967756	1.4251711	-2.1204239
C	2.5727924	-1.4380066	-0.7616444
H	0.5296625	-1.7990619	-1.3075659
C	2.9304709	0.0045044	1.1542925
H	1.1534950	0.7581549	2.1042450
C	3.4407163	-0.7851972	0.1198853
H	2.9720064	-2.0362002	-1.5744819
H	3.6052220	0.5025060	1.8432319
H	4.5144153	-0.8908102	-0.0014819
Si	-5.2074501	-1.8451464	1.5345154
C	-6.6579625	-2.9595932	1.1371552
C	-4.1220932	-2.5588646	2.8842777
C	-5.7486975	-0.0931881	1.9155579
H	-6.3323129	0.3321922	1.0912574
H	-6.3835007	-0.0858770	2.8096386
H	-4.9001328	0.5716257	2.1105140
H	-6.3267868	-3.9636049	0.8507907
H	-7.2995465	-3.0561619	2.0215731
H	-7.2661816	-2.5512546	0.3228541
H	-4.6641810	-2.5447148	3.8375417
H	-3.8505795	-3.5996196	2.6759911
H	-3.1986019	-1.9858498	3.0229690

E⁺ : *c*-$CH_2C(SiMe_3)C(Ph)C(Me)CH$-$CH_2SiMe_3^+$

52

Energy = -1322.090287443

C	-3.3151606	-1.0103118	-0.8160147
C	-3.2142340	0.5153681	-0.6914969
C	-1.9319567	-1.4713073	-0.5423366
C	-4.3727239	-1.6782167	0.1248052
C	-1.8202159	0.8021127	-0.2505732
H	-3.9446690	0.9496345	0.0035956
H	-3.4088026	1.0167917	-1.6510361
C	-1.1085741	-0.3998381	-0.1393837
H	-3.5802827	-1.2979074	-1.8450391
C	-1.4973091	-2.8705093	-0.7058403
H	-5.3408419	-1.2258487	-0.1249211
H	-4.4605367	-2.7356365	-0.1571136
Si	-1.1602023	2.5912061	-0.0254993
C	0.2876111	-0.5387627	0.3284964
H	-2.3069913	-3.5713861	-0.4867501
H	-1.2353794	-3.0204007	-1.7657010
H	-0.6083261	-3.0977422	-0.1139425
C	-2.2545073	3.6649805	-1.1063968
C	-1.3727358	3.0519863	1.7852797
C	0.6293730	2.6728244	-0.5732482
C	1.2946031	-1.0125722	-0.5222693
C	0.6077804	-0.1841614	1.6461532
H	-2.1822203	3.3855282	-2.1636803
H	-3.3072653	3.6118273	-0.8070912
H	-1.9400438	4.7116361	-1.0199245
H	-0.7558084	2.4368795	2.4464034
H	-1.0566090	4.0957839	1.9051605
H	-2.4158483	2.9788564	2.1081734
H	0.9382678	3.7252624	-0.5941063
H	1.2987492	2.1352766	0.1036445
H	0.7612408	2.2675493	-1.5820926
C	2.6079682	-1.1082758	-0.0652291
H	1.0567399	-1.2794543	-1.5482723
C	1.9203442	-0.2887393	2.1028541
H	-0.1757407	0.1595514	2.3152629
C	2.9226799	-0.7455457	1.2457727
H	3.3861534	-1.4626657	-0.7340901
H	2.1591634	-0.0156140	3.1260212
H	3.9462721	-0.8220126	1.5991778
Si	-4.1430334	-1.6138695	2.0227295
C	-5.6968969	-2.3856741	2.7386132
C	-2.6296009	-2.6076798	2.5440602
C	-3.9517943	0.1534625	2.6466961
H	-4.7595109	0.8081678	2.2986923
H	-3.9781379	0.1575853	3.7430976
H	-2.9971300	0.5997600	2.3475004
H	-5.8317456	-3.4155431	2.3877359
H	-5.6467794	-2.4112815	3.8334921
H	-6.5912174	-1.8163311	2.4593499
H	-2.5843064	-2.6437308	3.6395945
H	-2.6753196	-3.6419635	2.1847836
H	-1.6845598	-2.1703488	2.2021622

F⁺ : *c*-$CH_2C(SiMe_3)C(Ph)CH(Me)C$-$CH_2SiMe_3^+$

52

Energy = -1322.079755201

C	-3.2686491	-0.8693959	-0.3426772

C	-3.0856070	0.6122856	-0.3588180
C	-1.9370764	-1.5126122	-0.5299015
C	-4.4646273	-1.5298748	-0.0874988
C	-1.5992698	0.8159919	-0.0757506
H	-3.7741553	1.1265354	0.3246210
H	-3.3635203	0.9790062	-1.3630599
C	-0.9722672	-0.3816494	-0.1409012
H	-1.8758869	-1.6338349	-1.6327278
C	-1.7241022	-2.9023133	0.0802895
H	-5.3859106	-0.9825668	-0.2904432
H	-4.5050349	-2.5996159	-0.2864429
Si	-0.9054079	2.5633019	0.1442467
C	0.4500229	-0.6664227	0.0927405
H	-1.7926481	-2.8698005	1.1701322
H	-2.4570495	-3.6182770	-0.3020852
H	-0.7292309	-3.2678543	-0.1801878
C	-2.0285152	3.6818298	-0.8749012
C	-1.0352639	3.0513618	1.9593237
C	0.8550634	2.7051708	-0.4881271
C	1.2049312	-1.4064610	-0.8323238
C	1.0728661	-0.2170717	1.2672837
H	-2.0162540	3.4070204	-1.9364430
H	-3.0687648	3.6530634	-0.5290434
H	-1.6897811	4.7219658	-0.8009796
H	-0.3985066	2.4302739	2.5985907
H	-0.7238429	4.0943927	2.0931739
H	-2.0655829	2.9677530	2.3261541
H	1.1424238	3.7635516	-0.5148243
H	1.5767720	2.1751850	0.1389247
H	0.9390903	2.3098462	-1.5066987
C	2.5554162	-1.6574465	-0.6029540
H	0.7402425	-1.7620483	-1.7486765
C	2.4216378	-0.4769039	1.5008849
H	0.4855785	0.3270947	2.0009983
C	3.1675598	-1.1933482	0.5638971
H	3.1321252	-2.2147760	-1.3350904
H	2.8891096	-0.1239480	2.4153506
H	4.2189548	-1.3958477	0.7438432
Si	-4.5387175	-1.4210033	2.0033444
C	-4.8590148	-3.2007717	2.4560777
C	-2.9949758	-0.7261400	2.7919628
C	-5.9917085	-0.2793550	2.2588294
H	-6.9057854	-0.6762038	1.8058606
H	-6.1647830	-0.1659762	3.3364682
H	-5.8054966	0.7195541	1.8491467
H	-4.0086188	-3.8440819	2.2098338
H	-5.0227409	-3.2584565	3.5398493
H	-5.7515754	-3.5899644	1.9562505
H	-3.1822528	-0.7105604	3.8741236
H	-2.0985581	-1.3274122	2.6234740
H	-2.7825454	0.3005798	2.4816644

4a : MeCC-CH_2SiMe_3

22

Energy = -564.8641975279

C	1.0415742	1.0319775	-0.0092492
H	0.9976303	1.6807841	-0.8955281
H	0.9999707	1.6935611	0.8676365
C	-0.0765626	0.1005578	-0.0009909
C	-0.9528016	-0.7374765	0.0063495
C	-2.0228486	-1.7307665	0.0150819
H	-2.6810468	-1.5934220	0.8810048
H	-2.6390678	-1.6535137	-0.8883508
H	-1.6192324	-2.7493416	0.0591607
C	2.7923301	-0.9817623	-1.5419471
H	1.9471997	-1.6792463	-1.5672321
H	2.7563901	-0.3766318	-2.4558811
H	3.7181521	-1.5691260	-1.5647637
Si	2.7095804	0.0981968	-0.0046802
C	4.0979147	1.3720202	-0.0149443
C	2.7956828	-0.9605626	1.5470750
H	5.0795407	0.8826643	-0.0131534
H	4.0458737	2.0101572	-0.9054018
H	4.0483747	2.0217417	0.8672358
H	2.7607804	-0.3430121	2.4526978
H	1.9511185	-1.6582624	1.5833119
H	3.7219898	-1.5468709	1.5763830

3aa : *c*-$CH_2C(SiMe_3)C(Ph)CH(Me)C{=}CH_2$

39

Energy = -912.8854961936

C	-2.2585336	-1.9227684	-0.0956598
C	-2.5033208	-0.4326375	0.0651418
C	-0.7875326	-2.1441539	-0.4184340
C	-3.1720368	-2.8806472	0.0708016
C	-1.1106884	0.1870701	0.0782484
H	-3.0745156	-0.2073223	0.9761900
H	-3.0994425	-0.0335873	-0.7690888

C	-0.1821203	-0.7639884	-0.1748993
H	-0.6776073	-2.3954977	-1.4868315
C	-0.1253489	-3.2671100	0.3956391
H	-4.2021300	-2.6445228	0.3272644
H	-2.9280420	-3.9339858	-0.0427362
Si	-0.9107416	2.0501086	0.2586618
C	1.2787436	-0.5777196	-0.2333183
H	-0.2102872	-3.0601633	1.4674012
H	-0.6066791	-4.2284860	0.1909222
H	0.9358960	-3.3547181	0.1418860
C	-2.5082345	2.8256033	-0.3848798
C	-0.7213735	2.5359611	2.0737292
C	0.5202473	2.7312441	-0.7596714
C	2.0268060	-1.0630504	-1.3191992
C	1.9624687	0.0639169	0.8105963
H	-2.6816599	2.5715959	-1.4375576
H	-3.3819175	2.4931041	0.1880525
H	-2.4584496	3.9188799	-0.3102238
H	0.2263496	2.1851311	2.4967562
H	-0.7515841	3.6274203	2.1831477
H	-1.5334827	2.1168409	2.6798294
H	0.4821957	3.8281352	-0.7568728
H	1.4971864	2.4239303	-0.3766996
H	0.4490951	2.3955091	-1.8008238
C	3.4060056	-0.8748917	-1.3764386
H	1.5184007	-1.5728662	-2.1334172
C	3.3434608	0.2465949	0.7590675
H	1.3983373	0.4051934	1.6733841
C	4.0703311	-0.2167775	-0.3384981
H	3.9650858	-1.2427081	-2.2325817
H	3.8529399	0.7455058	1.5790476
H	5.1465730	-0.0757615	-0.3814063

1a : allenylsilane $(Me_3Si)C(Me)CCCH_2$

22

Energy = -564.8681002726

C	2.9886835	-0.8692458	-0.0018853
H	3.4463538	-1.2068934	0.9259735
H	3.4455889	-1.2053929	-0.9306619
C	1.9358066	-0.0876782	-0.0008290
C	0.8584410	0.6545674	0.0001779
C	-0.5620158	-2.0793887	-0.0003258
C	-1.7709456	0.3129796	1.5375934
C	-1.7710199	0.3135248	-1.5372868
H	-0.0020365	-2.4026055	-0.8849017
H	-0.0025329	-2.4030029	0.8844237
H	-1.5279897	-2.5991135	-0.0007182
H	-1.9208679	1.3988490	1.5609785
H	-2.7607973	-0.1594137	1.5630090
H	-1.2371631	0.0269963	2.4513900
H	-2.7611770	-0.1582419	-1.5624507
H	-1.9202571	1.3994871	-1.5607145
H	-1.2376458	0.0271823	-2.4512092
Si	-0.8177041	-0.2188525	0.0000395
C	0.9227855	2.1756491	0.0014963
H	0.4130340	2.5831242	-0.8797656
H	0.4150682	2.5814612	0.8847031
H	1.9563244	2.5326528	0.0006409

2a : PhCCMe

17

Energy = -347.9310559635

C	-1.3905612	0.0001018	-0.0007277
C	-2.6043513	0.0006443	-0.0010849
C	0.0347982	-0.0000556	-0.0005131
C	0.7525451	-1.2125984	-0.0016028
C	0.7526471	1.2124332	0.0010187
C	2.1446528	-1.2078440	-0.0010760
H	0.2055367	-2.1503108	-0.0025720
C	2.1447267	1.2076425	0.0012809
H	0.2057436	2.1502050	0.0021239
C	2.8465453	-0.0001303	0.0003207
H	2.6842006	-2.1508030	-0.0017172
H	2.6843057	2.1505817	0.0023830
H	3.9326963	-0.0001506	0.0006752
C	-4.0613245	0.0002215	-0.0000268
H	-4.4529626	-0.7878839	-0.6532576
H	-4.4518670	-0.1739705	1.0096029
H	-4.4533938	0.9606383	-0.3529300

$SiMe_3^+$: silyl cation

13

Energy = -409.1329784800

Si	-0.0000309	0.0000890	0.0129885
C	-1.4383352	-1.1343878	0.0049688
C	1.7016352	-0.6783392	0.0187503
C	-0.2633626	1.8128615	0.0192801
H	0.6712112	2.3778799	-0.0039963

H	-0.8869091	2.0927536	-0.8411103
H	-0.8340839	2.0864763	0.9184856
H	-1.3676972	-1.8016227	-0.8655116
H	-1.3914346	-1.7786460	0.8945602
H	-2.3948993	-0.6072591	-0.0137117
H	2.2481322	-0.2795533	0.8847942
H	1.7235126	-1.7702331	0.0414789
H	2.2322476	-0.3199970	-0.8751135

cb⁻: anion $HCB_{11}H_5Br_6^-$
24
Energy = -15760.33649373

B	0.6911862	-1.3347825	0.7226185
B	1.4940278	0.2613477	0.7011189
B	-1.0750422	-1.0646143	0.7085422
B	-0.0007890	0.0004176	-0.2502753
B	-0.2427567	-1.4537719	2.2213874
B	1.3360545	-0.6388976	2.2168322
Br	1.5068342	-2.9179556	-0.0982172
B	0.2239770	1.5180167	0.6739517
B	1.0491292	1.1142677	2.1868576
Br	3.2424382	0.5317253	-0.1444788
B	-1.3638166	0.6984969	0.6786077
B	-1.5055181	-0.2042829	2.1943718
Br	-2.3128625	-2.3342071	-0.1288047
Br	0.0096371	-0.0308913	-2.2014030
C	-0.0181882	0.0527046	2.9783821
H	-0.3978359	-2.4343009	2.8625320
H	2.2282666	-1.0791121	2.8547096
B	-0.7071194	1.3828308	2.1730920
Br	0.4943420	3.2507974	-0.2032238
H	1.7510170	1.8367872	2.8048541
Br	-2.9372432	1.4784518	-0.1939184
H	-2.4980495	-0.3560676	2.8175949
H	-0.0240938	0.0701041	4.0603312
H	-1.1702277	2.2836521	2.7820332

Scb : $Me_3Si(HCB_{11}H_5Br_6)$
37
Energy = -16169.57484574

B	-0.0412689	0.3048307	0.4398975
B	-0.7439445	1.6402083	1.3616670
B	-0.1645888	0.6052365	-1.2968129
B	0.1789584	1.9551065	-0.1556591
B	-1.2772375	-0.5178743	-0.4845602
B	-1.6340962	0.1194277	1.1499252
Br	1.6072545	-0.5859336	1.2053422
B	-1.3593003	2.8127932	0.1596394
B	-2.4654753	1.6758134	0.9572982
Br	-0.0496176	2.1548124	3.1135704
B	-1.0037879	2.1730725	-1.4809610
B	-1.8916278	0.6481549	-1.6732315
Br	1.1795817	-0.0391673	-2.5610081
Br	1.9196560	2.8234948	-0.1132819
C	-2.6615650	0.4107003	-0.1684168
H	-1.3574129	-1.6772553	-0.6934404
H	-1.9360080	-0.6200098	2.0187558
B	-2.6232555	2.0002808	-0.7819840
Br	-1.4138276	4.7247975	0.5348508
H	-3.3376369	1.9638813	1.6980987
Br	-0.6455127	3.3462885	-2.9958847
H	-2.3855902	0.2554713	-2.6704833
Si	1.2889738	-2.9642905	1.0746482
H	-3.6140313	-0.1027804	-0.1769330
H	-3.6074016	2.5040700	-1.1943791
C	2.7997102	-3.4808983	2.0316520
C	-0.3171595	-3.3020412	1.9431367
C	1.3442359	-3.3838413	-0.7308666
H	2.8332880	-4.5778351	2.0602180
H	3.7205571	-3.1296478	1.5554469
H	2.7719273	-3.1166906	3.0632731
H	-1.1738267	-2.9122634	1.3884997
H	-0.4319570	-4.3910567	2.0274968
H	-0.3244730	-2.8792199	2.9520225
H	0.5320049	-2.9138727	-1.2902004
H	2.2943120	-3.0794733	-1.1791591
H	1.2472730	-4.4735190	-0.8297240

TS0⁺ : **A⁺** catalyzed isomerization of **1a**
57
Energy = -1538.933262070

C	-3.3177214	0.8487608	-0.5483221
H	-2.7954545	1.7408496	-0.1820050
H	-3.2474282	0.8294375	-1.6425582
C	-2.8001654	-0.3543064	0.0412744
C	-2.4553384	-1.4013907	0.5774164
C	-0.1653784	0.4773690	-1.1811787
C	0.3099941	-1.1376106	1.5969158

C	-0.4202576	-2.6961933	-1.2318924
H	-0.9060879	0.4128516	-1.9836275
H	-0.4368682	1.3153490	-0.5328051
H	0.8035058	0.7006249	-1.6355750
H	0.3331982	-2.1615915	1.9834586
H	1.2922538	-0.6929164	1.7762193
H	-0.4358159	-0.5731864	2.1632583
H	0.0168719	-2.5832782	-2.2290065
H	0.0299833	-3.5757989	-0.7620524
H	-1.4920177	-2.8724491	-1.3549069
Si	-0.0959456	-1.1348842	-0.2361171
C	-2.5142260	-2.6391600	1.3704666
H	-1.7900838	-3.3858742	1.0341684
H	-2.3173573	-2.4235955	2.4251663
H	-3.5147361	-3.0755678	1.2885025
C	-5.2942963	1.1202796	1.7967425
H	-4.8950165	0.2134270	2.2652932
H	-4.7331633	1.9781208	2.1854137
H	-6.3360415	1.2307989	2.1199625
Si	-5.1921775	1.0244159	-0.0733909
C	-5.7872984	2.6053949	-0.8876155
C	-6.0896786	-0.4833023	-0.7350722
H	-6.8455623	2.7761397	-0.6568752
H	-5.2243223	3.4761122	-0.5322322
H	-5.6878367	2.5571922	-1.9780957
H	-5.9667349	-0.5786888	-1.8201748
H	-5.7204668	-1.4045690	-0.2701002
H	-7.1637953	-0.4129661	-0.5270587
C	2.2732022	-1.6157324	-0.7847662
H	2.1371475	-1.7293027	-1.8586263
H	2.3144338	-2.5325469	-0.1977776
C	2.8169682	-0.4948087	-0.3045605
C	3.3689554	0.5802238	0.1505781
C	5.8638992	-0.9382957	-0.8752321
C	5.6824568	2.1527975	-1.0490542
C	5.9779268	0.7683607	1.7110158
H	5.5823601	-1.8334063	-0.3097821
H	5.4510493	-1.0284606	-1.8860491
H	6.9569987	-0.9288625	-0.9611950
H	5.3498813	3.0722596	-0.5547493
H	6.7666753	2.2295297	-1.1960063
H	5.2126661	2.1053610	-2.0377475
H	7.0728509	0.8200606	1.6718259
H	5.6228122	1.6707597	2.2209118
H	5.7045571	-0.0999251	2.3207140
Si	5.2856575	0.6286587	-0.0279637
C	2.6647131	1.7630354	0.7904022
H	3.0685407	1.9381850	1.7924209
H	2.8471351	2.6662939	0.1997997
H	1.5872676	1.6098485	0.8695909

TS1$^+$: $SiMe_3^+$ transfer from **A$^+$** to **2a**

52

Energy = -1321.994187679

C	-3.1892047	-0.0993939	-0.8521325
H	-3.1392954	0.6846411	-1.6170228
H	-2.9695093	-1.0601531	-1.3365217
C	-2.2801187	0.1528485	0.2364103
C	-1.5933216	0.3351368	1.2319388
C	-0.2228373	0.6182220	-1.9413244
C	1.2945115	1.2145340	0.6783114
C	0.1863655	-1.8583443	0.2475504
H	-0.8048028	-0.0940647	-2.5295591
H	-0.8572791	1.4776555	-1.7022984
H	0.6108289	0.9765436	-2.5531001
H	0.5745040	1.9744950	0.9946217
H	1.7977867	0.8177003	1.5632876
H	2.0445501	1.7112677	0.0555591
H	-0.6881568	-2.2971329	-0.2429958
H	1.0558480	-2.4891715	0.0479532
H	-0.0069881	-1.8627040	1.3248731
Si	0.4309009	-0.0922958	-0.3387019
C	-1.1034305	0.5137271	2.6018663
H	-0.2013952	-0.0789219	2.7863101
H	-0.8662186	1.5631688	2.7998242
H	-1.8742442	0.1932900	3.3104446
C	-5.3832702	1.4710988	0.6301565
H	-4.7095480	1.6701339	1.4714978
H	-5.2854522	2.2964810	-0.0846481
H	-6.4098249	1.4844200	1.0148661
Si	-5.0022677	-0.1798072	-0.1733688
C	-6.1048102	-0.5048354	-1.6556847
C	-5.0758354	-1.5823119	1.0704192
H	-7.1545054	-0.5628254	-1.3441559
H	-6.0225295	0.2961556	-2.3993854
H	-5.8505416	-1.4518790	-2.1456009
H	-4.8145863	-2.5415842	0.6083867
H	-4.3879149	-1.4102492	1.9062309

H	-6.0861566	-1.6778013	1.4851649
C	2.2614648	-1.0353128	-1.7278079
C	3.1169059	-0.6006413	-0.9641011
C	1.6307759	-1.7789832	-2.8245394
H	0.7091242	-2.2726399	-2.5012689
H	1.3843122	-1.1093520	-3.6531950
H	2.3234953	-2.5445448	-3.1874196
C	4.0815596	-0.1070768	-0.0500475
C	4.7941137	1.0748389	-0.3375795
C	4.3348121	-0.8014077	1.1506841
C	5.7391162	1.5498509	0.5645080
H	4.6007873	1.6014428	-1.2666369
C	5.2789156	-0.3135487	2.0464391
H	3.7907267	-1.7164142	1.3625254
C	5.9798715	0.8605226	1.7560726
H	6.2901000	2.4575639	0.3398271
H	5.4740967	-0.8498661	2.9695458
H	6.7183634	1.2363164	2.4575536

TS2$^+$: C-C addition of **B$^+$** and **4a**

52

Energy = -1322.005356544

C	0.2033824	1.7003637	1.7760544
C	-2.3433004	-0.2153535	1.6468796
C	0.8419160	0.8072583	2.3177730
C	-0.5443423	2.8018365	1.2140869
C	-1.3232390	-0.8369493	0.7373154
H	-2.9888529	0.4602300	1.0772975
H	-2.9842352	-0.9892430	2.0793979
C	-0.0538497	-0.6863009	0.6095214
H	-1.8489447	0.3374211	2.4474478
C	1.6779238	-0.0553571	3.1457370
H	-1.6201829	2.6589997	1.3823696
H	-0.2640148	3.7027443	1.7825297
Si	-2.0252040	-2.1570641	-0.5411572
C	1.2170360	-1.0929441	0.1453595
H	2.1353333	0.5384696	3.9441422
H	1.0890944	-0.8546740	3.6077338
H	2.4810806	-0.5125966	2.5579637
C	-2.3326413	-3.6925434	0.4854118
C	-3.6084910	-1.3797539	-1.1744137
C	-0.8343131	-2.4747087	-1.9460864
C	1.7619880	-2.2958050	0.6556251
C	1.9292240	-0.3642130	-0.8315923
H	-1.3946049	-4.1100139	0.8670213
H	-2.9926072	-3.4940267	1.3363414
H	-2.8114147	-4.4584972	-0.1372426
H	-3.4159545	-0.4301212	-1.6846625
H	-4.0672585	-2.0616946	-1.9010974
H	-4.3348931	-1.2058194	-0.3744887
H	-1.3436780	-3.1042709	-2.6864232
H	-0.5375135	-1.5488205	-2.4499480
H	0.0700279	-3.0018155	-1.6292339
C	2.9762922	-2.7660941	0.1730356
H	1.2110725	-2.8475993	1.4101324
C	3.1434958	-0.8401457	-1.2995929
H	1.5146451	0.5602681	-1.2127150
C	3.6674191	-2.0395383	-0.8005277
H	3.3870807	-3.6948565	0.5552153
H	3.6874194	-0.2837277	-2.0558807
H	4.6190430	-2.4065111	-1.1723051
Si	-0.2492549	3.2648461	-0.6310038
C	-1.1225289	4.9047379	-0.8890267
C	1.6016508	3.4432532	-0.8966877
C	-1.0265875	1.9670082	-1.7502386
H	-2.1072833	1.9140545	-1.5720245
H	-0.8797729	2.2398837	-2.8019572
H	-0.6181319	0.9631280	-1.6047396
H	-0.7200312	5.6810616	-0.2281955
H	-0.9965862	5.2494559	-1.9222070
H	-2.1986028	4.8210638	-0.6966416
H	1.8357653	3.5833112	-1.9583850
H	1.9779653	4.3200719	-0.3565001
H	2.1627579	2.5745148	-0.5361109

TS3$^+$: hydride transfer within **C$^+$**

52

Energy = -1322.024742072

C	-1.6453522	-1.1273799	-0.8470264
C	-0.9654679	1.3640736	-0.4755408
C	-0.3809019	-1.3686637	-0.6057945
C	-3.0201556	-1.3783998	-0.8809145
C	0.4333379	0.9267668	-0.2499100
H	-1.5440594	1.3859419	0.4539990
H	-1.0702965	2.3303776	-0.9753658
C	0.6916490	-0.4084494	-0.2573574
H	-1.4753601	0.6089039	-1.1513908
C	-0.0253037	-2.8532678	-0.7640224

H	-3.4421971	-1.4119751	-1.8921433
H	-3.2251386	-2.3061982	-0.3229451
Si	1.7585103	2.2929250	0.0024794
C	2.0268251	-0.9779630	0.0646835
H	-0.9087166	-3.4702971	-0.9324391
H	0.6601899	-2.9717906	-1.6061968
H	0.4791515	-3.1727254	0.1525278
C	1.1331544	3.8103050	-0.9193560
C	1.8388800	2.6654173	1.8450073
C	3.4443831	1.8223573	-0.6706978
C	2.8610906	-1.4945665	-0.9372891
C	2.4745183	-0.9751524	1.3925872
H	0.9621142	3.6039358	-1.9826095
H	0.2054589	4.2092756	-0.4934353
H	1.8866607	4.6045322	-0.8571268
H	2.2514133	1.8237620	2.4102910
H	2.4861573	3.5342799	2.0167069
H	0.8492398	2.9005017	2.2530594
H	4.0742911	2.7212625	-0.6635316
H	3.9444642	1.0558054	-0.0730057
H	3.3847899	1.4665360	-1.7046035
C	4.1285390	-1.9757704	-0.6182519
H	2.5312654	-1.4821019	-1.9724760
C	3.7421483	-1.4612142	1.7115975
H	1.8244886	-0.5894805	2.1723950
C	4.5720581	-1.9586041	0.7061552
H	4.7750073	-2.3545580	-1.4040942
H	4.0814125	-1.4487362	2.7429693
H	5.5615882	-2.3314593	0.9523845
Si	-4.2638039	-0.2161608	0.1552505
C	-5.8596116	-1.1846296	0.0665839
C	-3.6110710	-0.1156750	1.9043525
C	-4.4317148	1.4230452	-0.7264694
H	-4.7268956	1.2766164	-1.7713224
H	-5.2323751	1.9899861	-0.2339768
H	-3.5321178	2.0418754	-0.7045477
H	-5.7700462	-2.1632840	0.5507567
H	-6.6523496	-0.6288659	0.5817853
H	-6.1826429	-1.3395232	-0.9687717
H	-4.3155259	0.4575132	2.5192274
H	-3.5264342	-1.1156636	2.3446004
H	-2.6344152	0.3713473	1.9820607

TS4$^+$: ring-closing of **D$^+$**

52

Energy = -1322.060933908

C	-1.5125782	-0.0772989	-1.1940624
C	-0.8682415	1.3531155	0.1672812
C	-0.5098563	-0.9949378	-0.7381702
C	-2.9443338	-0.4353819	-1.3396810
C	0.4871175	0.9738551	0.0688911
H	-1.5078992	0.8335275	0.8761215
H	-1.1817362	2.3717777	-0.0524785
C	0.6516142	-0.4096162	-0.1848832
H	-1.1586303	0.6924983	-1.8760428
C	-0.7458334	-2.4709591	-0.7851835
H	-3.3975870	0.1254451	-2.1628515
H	-3.0272157	-1.5043640	-1.5795963
Si	1.8864954	2.2934999	0.0100592
C	1.9049312	-1.1303993	0.0785122
H	-1.5962358	-2.7191834	-0.1376654
H	-1.0094860	-2.8030088	-1.7957701
H	0.1230595	-3.0256340	-0.4316981
C	1.1056088	3.7683034	-0.8573436
C	2.3574645	2.7435842	1.7704342
C	3.3452314	1.6543220	-0.9757794
C	2.4968711	-1.9525999	-0.8939400
C	2.5451644	-0.9614056	1.3167798
H	0.7162283	3.5053812	-1.8475120
H	0.2942555	4.2162974	-0.2734110
H	1.8697919	4.5416062	-1.0002279
H	2.8588670	1.9181736	2.2850889
H	3.0492405	3.5946004	1.7516605
H	1.4805024	3.0360515	2.3581885
H	4.0396162	2.4847153	-1.1541594
H	3.8930951	0.8650765	-0.4535011
H	3.0344454	1.2666752	-1.9522841
C	3.7143833	-2.5759192	-0.6369701
H	2.0194770	-2.0716229	-1.8625035
C	3.7561663	-1.5973242	1.5767224
H	2.0750995	-0.3470258	2.0792586
C	4.3450259	-2.3998944	0.5975944
H	4.1756622	-3.1950444	-1.4000773
H	4.2397901	-1.4684694	2.5400009
H	5.2931893	-2.8900333	0.7960842
Si	-4.1055588	-0.2390223	0.1930516
C	-5.7145228	-1.0752151	-0.2788151
C	-3.3181739	-1.1150474	1.6622898

C	-4.3977258	1.5834791	0.5417762
H	-4.7790854	2.0951029	-0.3494243
H	-5.1545821	1.6839761	1.3292732
H	-3.5050434	2.1175473	0.8819748
H	-5.5668752	-2.1401934	-0.4915233
H	-6.4406115	-0.9954138	0.5387667
H	-6.1609611	-0.6109699	-1.1656873
H	-3.8991683	-0.9084984	2.5690658
H	-3.3179444	-2.2013354	1.5144928
H	-2.2849164	-0.8112028	1.8708184

TS5$^+$: 1,2-H-shift within **E$^+$**

52

Energy = -1322.063101369

C	-1.5402016	-0.1455867	-1.0622048
C	-0.9287044	1.2090225	-0.8111090
C	-0.5072816	-1.1091130	-0.8130882
C	-2.9622040	-0.3994096	-1.3136513
C	0.4950128	0.9566289	-0.3731302
H	-1.5165845	1.7020865	-0.0240226
H	-1.0046720	1.8742004	-1.6807280
C	0.7160188	-0.3874406	-0.3710807
H	-0.7344997	-0.6770958	-2.0097667
C	-0.6741279	-2.5953063	-0.7783909
H	-3.3835808	0.3321509	-2.0114243
H	-3.1321067	-1.4060236	-1.7089503
Si	1.7132951	2.3752219	-0.0226165
C	1.9476632	-1.1050389	0.0123209
H	-1.6001129	-2.9192976	-1.2564662
H	0.1758945	-3.0998412	-1.2416702
H	-0.6985906	-2.8997487	0.2738729
C	1.1083473	3.8339703	-1.0444516
C	1.6159447	2.7934346	1.8080669
C	3.4488217	1.8889805	-0.5386433
C	2.6536705	-1.8821635	-0.9180134
C	2.4315823	-1.0024389	1.3241804
H	1.1056702	3.6059873	-2.1169552
H	0.0965482	4.1457649	-0.7588883
H	1.7690889	4.6955984	-0.8933577
H	1.9826882	1.9722197	2.4324405
H	2.2311549	3.6759325	2.0213137
H	0.5889575	3.0227421	2.1151736
H	4.1064206	2.7634923	-0.4611304
H	3.8650712	1.0970385	0.0904282
H	3.4726994	1.5445194	-1.5784794
C	3.8321402	-2.5257438	-0.5463553
H	2.2991414	-1.9548850	-1.9431084
C	3.6076831	-1.6519659	1.6956302
H	1.8765923	-0.4174346	2.0515775
C	4.3113973	-2.4118011	0.7604553
H	4.3805945	-3.1107472	-1.2784219
H	3.9729353	-1.5654535	2.7144692
H	5.2291869	-2.9153513	1.0481437
Si	-4.0249457	-0.2942651	0.3197440
C	-5.6915897	-1.0072795	-0.1480519
C	-3.1620668	-1.3119683	1.6393845
C	-4.1793253	1.4993633	0.8492887
H	-4.5136979	2.1414155	0.0266187
H	-4.9273790	1.5721447	1.6484143
H	-3.2478249	1.9128559	1.2524670
H	-5.6049028	-2.0480685	-0.4795924
H	-6.3657275	-0.9860625	0.7163263
H	-6.1622531	-0.4308262	-0.9524444
H	-3.7321363	-1.2678079	2.5748681
H	-3.0815875	-2.3666151	1.3540250
H	-2.1548887	-0.9361859	1.8579792

TS6$^+$: $SiMe_3^+$ from **F$^+$** to **4a**

74

Energy = -1886.949468039

C	1.5366990	-2.1577154	-1.1653619
C	1.8916478	-1.0748258	-2.1489965
C	2.3864856	-1.9716977	0.0669027
C	0.5869389	-3.1188256	-1.3387873
C	2.6217484	-0.0251869	-1.3166820
H	1.0135020	-0.6896713	-2.6835671
H	2.5442148	-1.4925069	-2.9318885
C	2.8701598	-0.5251988	-0.0835876
H	3.2562097	-2.6402787	-0.0748123
C	1.7430567	-2.3442924	1.4076351
H	0.1761227	-3.3070273	-2.3271537
H	0.4866533	-3.9190312	-0.6118308
Si	3.1157017	1.6170508	-2.1104327
C	3.5272999	0.1567522	1.0423253
H	0.8720276	-1.7163131	1.6134189
H	1.4363291	-3.3943889	1.4180254
H	2.4597839	-2.1966298	2.2183931
C	3.3577104	1.2502478	-3.9434431

C	1.6873615	2.8353633	-1.9227516
C	4.7116880	2.3081446	-1.4046220
C	4.5810852	-0.4540079	1.7422054
C	3.0907906	1.4237228	1.4574110
H	4.1505550	0.5100889	-4.1044967
H	2.4423501	0.8727125	-4.4145284
H	3.6466799	2.1641473	-4.4758991
H	1.5183049	3.1141704	-0.8768864
H	1.8947996	3.7556164	-2.4821320
H	0.7530196	2.4150014	-2.3145091
H	5.0393852	3.1571592	-2.0173081
H	4.6071284	2.6555035	-0.3734418
H	5.5061285	1.5535521	-1.4262046
C	5.2024720	0.2021146	2.8021700
H	4.9327901	-1.4364475	1.4374852
C	3.7053873	2.0765996	2.5241439
H	2.2546613	1.8874898	0.9420449
C	4.7670679	1.4695594	3.1963349
H	6.0282103	-0.2751358	3.3216869
H	3.3540679	3.0569208	2.8328034
H	5.2487639	1.9770803	4.0266152
H	-0.9314934	-1.3916696	-3.1659633
C	-1.8079141	-1.6502910	-2.5622818
Si	-1.3377418	-1.7466114	-0.7546896
H	-2.2032037	-2.6008372	-2.9317012
H	-2.5576667	-0.8689334	-2.7144898
C	-1.8625660	-3.1234286	0.3986180
C	-0.7079863	-0.1466304	-0.0327239
H	-1.0716793	-3.3060607	1.1323160
H	-2.7718510	-2.8448040	0.9375621
H	-2.0426557	-4.0533069	-0.1491256
H	-0.8856648	-0.1018086	1.0451019
H	0.3611417	0.0048964	-0.2074624
H	-1.2407190	0.6845289	-0.5048162
H	-4.7386181	-2.0566182	-2.4225977
C	-4.7659323	-2.2830992	-1.3521429
H	-5.8141516	-2.4069458	-1.0593760
H	-4.2593431	-3.2409772	-1.1932286
C	-4.1582845	-1.2091460	-0.5673300
C	-3.8459608	-0.2699210	0.1444977
C	-3.6594858	0.8592708	1.0303356
H	-3.0658487	0.5794945	1.9103302
H	-3.1364739	1.6823149	0.5265643
Si	-5.3747753	1.5055110	1.6365200
C	-6.2349938	0.0950681	2.5266604
C	-5.0339692	2.9440572	2.7941157
C	-6.3463482	2.0524997	0.1272821
H	-6.3892990	-0.7591205	1.8576605
H	-5.6510070	-0.2503324	3.3879396
H	-7.2171601	0.4120716	2.8964609
H	-4.4904516	3.7488941	2.2854058
H	-5.9741445	3.3651376	3.1696889
H	-4.4413617	2.6295553	3.6612119
H	-7.3352138	2.4264995	0.4174288
H	-5.8311916	2.8574437	-0.4101516
H	-6.4950662	1.2209416	-0.5711309

TS6a⁺ : $SiMe_3^+$ from **F⁺** to allenylsilane **1a**

74

Energy = -1886.951706839

C	1.5818996	-1.9001369	-1.5883756
C	1.5536270	-0.5016533	-2.1437900
C	2.6731381	-1.9751754	-0.5521988
C	0.7383014	-2.9156689	-1.9311460
C	2.3397586	0.3286880	-1.1340087
H	0.5299466	-0.1436514	-2.3160482
H	2.0331381	-0.4921039	-3.1352759
C	2.9357294	-0.4970143	-0.2423126
H	3.5532542	-2.3723474	-1.0927733
C	2.4249140	-2.9010451	0.6451576
H	0.1505397	-2.8447246	-2.8422465
H	0.9146487	-3.9157436	-1.5462182
Si	2.4333478	2.2042279	-1.3407372
C	3.7597893	-0.1176516	0.9158541
H	1.5640674	-2.5662220	1.2296210
H	2.2540636	-3.9302488	0.3160015
H	3.2968843	-2.8994860	1.3026065
C	2.2948896	2.5248803	-3.1930938
C	0.9435397	2.9825888	-0.4831486
C	4.0474206	2.9242556	-0.7092600
C	5.0174804	-0.7043301	1.1329242
C	3.2860333	0.8200164	1.8455877
H	3.1169201	2.0542492	-3.7453964
H	1.3515933	2.1509158	-3.6088534
H	2.3367822	3.6019761	-3.3939086
H	0.9699104	2.8364464	0.6021810
H	0.9147468	4.0622926	-0.6732003
H	0.0045639	2.5568406	-0.8567829

H	4.1233060	3.9713715	-1.0276787
H	4.1306521	2.8951139	0.3802946
H	4.9054460	2.3848024	-1.1261649
C	5.7924642	-0.3336767	2.2293786
H	5.4012918	-1.4347668	0.4251597
C	4.0563575	1.1835986	2.9483915
H	2.2998553	1.2520896	1.7019924
C	5.3146820	0.6115175	3.1402839
H	6.7709565	-0.7821472	2.3740406
H	3.6732689	1.9096973	3.6595206
H	5.9175573	0.8943924	3.9978970
Si	-1.1448825	-2.2267317	-0.6166772
C	-1.2806422	-3.9899292	0.0023395
C	-0.5322324	-0.9154209	0.5701837
C	-2.0381148	-1.7320592	-2.1888321
H	-2.2122007	-2.6211528	-2.8044963
H	-3.0022912	-1.2663678	-1.9760404
H	-1.4431833	-1.0264667	-2.7787595
H	-0.3568843	-4.2703275	0.5182875
H	-2.1081641	-4.1198124	0.7031882
H	-1.4293794	-4.6757679	-0.8379027
H	-0.5419545	-1.3211126	1.5873993
H	0.4786092	-0.5604626	0.3540585
H	-1.2033258	-0.0538888	0.5521668
C	-3.5605180	-1.9263158	0.7339998
H	-3.1040766	-2.1906038	1.6857133
H	-4.0211842	-2.7295500	0.1616166
C	-3.7807000	-0.6592978	0.4181868
C	-4.0478217	0.5792183	0.1336518
C	-6.5073523	-0.0658357	1.8866083
C	-5.2065239	2.7475590	1.9329962
C	-6.7527886	1.8000706	-0.5748003
H	-6.7528514	-0.9421598	1.2766670
H	-5.8720922	-0.3966823	2.7156014
H	-7.4418658	0.3181100	2.3127881
H	-4.7083982	3.5281984	1.3469153
H	-6.1052712	3.1916492	2.3778341
H	-4.5377022	2.4548541	2.7503025
H	-7.6974042	2.2176382	-0.2056053
H	-6.2677344	2.5708073	-1.1844541
H	-6.9928135	0.9514554	-1.2252464
Si	-5.6748810	1.2699677	0.8702009
C	-3.1833062	1.4979100	-0.7130625
H	-2.3203803	0.9835167	-1.1412588
H	-3.7733318	1.9186903	-1.5334935
H	-2.8235898	2.3372163	-0.1080722

TS7 Me3Si+ transfer from **Scb** to **1a**

59

Energy = -16734.44902929

B	-0.1057938	0.2581734	0.5191761
B	-1.1896631	1.5352484	1.1151761
B	0.4045739	0.6443631	-1.1377172
B	0.2179152	1.9517434	0.0782180
B	-0.8657269	-0.5570420	-0.8399597
B	-1.8482682	-0.0047327	0.5417777
Br	1.1342198	-0.6494147	1.7867183
B	-1.3686132	2.7325083	-0.2011641
B	-2.6296178	1.5206878	0.0854473
Br	-1.2676058	2.0151828	3.0090421
B	-0.3850506	2.1800808	-1.5935198
B	-1.0467216	0.6308429	-2.1477932
Br	2.1623585	0.1402947	-1.8406669
Br	1.7562368	2.9131320	0.7836252
C	-2.3155382	0.2999763	-1.0605947
H	-0.8011795	-1.7101226	-1.0920581
H	-2.4335898	-0.7848478	1.2071962
B	-2.1352472	1.9132228	-1.5741328
Br	-1.6708586	4.6255715	0.1671938
H	-3.7328146	1.7391251	0.4445876
Br	0.4534980	3.4322484	-2.8351165
H	-1.1010960	0.2627373	-3.2684568
Si	1.0079659	-3.3825060	1.4694255
H	-3.1649007	-0.2505635	-1.4431783
H	-2.9140982	2.3949962	-2.3190690
C	2.3013465	-3.4065212	2.8304683
C	-0.7950559	-3.2857274	1.9613766
C	1.6217370	-3.3083473	-0.2916684
C	1.0541364	-5.8250280	1.4410607
H	2.7063135	-4.4044052	3.0154341
H	3.1343438	-2.7525613	2.5506655
H	1.8692463	-3.0313702	3.7639337
H	-1.3682516	-2.7383816	1.2085251
H	-1.2435149	-4.2744138	2.0897706
H	-0.8819372	-2.7339724	2.9022692
H	0.8784890	-3.6948819	-0.9934380
H	1.8807667	-2.2899648	-0.5926153
H	2.5209026	-3.9294059	-0.3754431

H	0.8090624	-5.9541201	2.4945638
H	2.1028628	-5.9196560	1.1646685
C	-0.8137425	-6.1674377	-0.3738203
C	0.1060722	-5.9820868	0.5139267
C	-1.6018624	-5.0997014	-1.1108483
H	-2.6742914	-5.2899416	-1.0042058
H	-1.3889084	-4.0967210	-0.7393322
H	-1.3648261	-5.1368426	-2.1791025
Si	-1.1722522	-8.0132220	-0.7642412
C	-2.9710701	-8.3180551	-0.3189416
H	-3.6465482	-7.6793640	-0.8987790
H	-3.2372753	-9.3608258	-0.5309238
H	-3.1555222	-8.1332312	0.7451567
C	-0.8807712	-8.2371630	-2.6056159
H	0.1563229	-8.0090017	-2.8756447
H	-1.0833417	-9.2761192	-2.8931600
H	-1.5380691	-7.5938588	-3.2011163
C	-0.0258083	-9.0956479	0.2485111
H	1.0265245	-8.8969765	0.0174718
H	-0.1728996	-8.9457433	1.3237859
H	-0.2228579	-10.1516918	0.0283771

TS8 : Me_3Si^+ transfer from **Scb** to **4a**
59
Energy = -16734.44856224

B	1.1116585	-0.0774252	1.5994953
B	2.0746071	-1.3834348	0.8739860
B	1.6231792	1.4777538	0.9085701
B	1.5491854	0.0120597	-0.1209977
B	2.1856283	0.9830098	2.5133846
B	2.4634191	-0.7766206	2.4921108
Br	-0.7235023	-0.3771672	2.3058957
B	3.2121856	-0.6304604	-0.2813917
B	3.7638443	-1.1120657	1.3310916
Br	1.3572557	-3.1874408	0.6125982
B	2.9325413	1.1399051	-0.2603275
B	3.3150325	1.7303079	1.3650677
Br	0.3816249	2.9786666	0.6891935
Br	0.2590246	-0.1716370	-1.5795141
C	3.7279340	0.3282003	2.2375061
H	1.9187562	1.5219859	3.5295736
H	2.3795018	-1.3948276	3.4946873
B	4.2890585	0.4361253	0.6343818
Br	3.8085428	-1.5635600	-1.8912294
H	4.5552379	-1.9562084	1.5652208
Br	3.2072576	2.2490297	-1.8454551
H	3.8082934	2.7716958	1.6217068
Si	-7.9485195	0.4835163	-0.6380716
H	4.4589646	0.4341318	3.0283846
H	5.4326522	0.6194095	0.4063073
C	-9.1889993	1.2270166	0.5579187
C	-8.6474135	-1.0220209	-1.5128585
C	-7.3157243	1.7567201	-1.8616866
C	-6.4427795	-0.0924615	0.4292532
H	-8.7674560	2.0916583	1.0835889
H	-10.0828736	1.5662662	0.0209893
H	-9.5086241	0.4955098	1.3091225
H	-7.9113647	-1.4655822	-2.1932695
H	-8.9535917	-1.7938815	-0.7971660
H	-9.5278968	-0.7491773	-2.1065355
H	-6.5582234	1.3276299	-2.5272782
H	-8.1338501	2.1345908	-2.4860434
H	-6.8655593	2.6139628	-1.3476042
H	-6.7877774	-0.8038287	1.1889878
H	-6.0464484	0.7897855	0.9484991
C	-4.6597330	-1.1086313	-1.2664396
C	-5.4416126	-0.6731894	-0.4304348
C	-3.9864079	-1.5426234	-2.4937280
H	-3.7755859	-2.6152521	-2.4758407
H	-4.6313309	-1.3256157	-3.3514406
H	-3.0394595	-1.0110272	-2.6332133
Si	-3.0166738	-0.7882105	0.5512517
C	-2.2557176	-2.3862186	-0.0343806
H	-1.6794263	-2.2778690	-0.9538419
H	-1.5716528	-2.7571227	0.7343902
H	-3.0526345	-3.1249035	-0.1757768
C	-2.7721525	0.9344723	-0.1171700
H	-3.6366326	1.5347966	0.1922951
H	-1.8614429	1.4104040	0.2517534
H	-2.7326412	0.9264655	-1.2092956
C	-3.9197200	-1.0085569	2.1770536
H	-4.2916171	-0.0619509	2.5777662
H	-4.7621929	-1.6951215	2.0474139
H	-3.2473130	-1.4552205	2.9163809

12 References

[S1] Harris, R. K.; Becker, E. D.; Cabral de Menezes, S. M.; Goodfellow, R.; Granger, P. NMR nomenclature. Nuclear spin properties and conventions for chemical shifts (IUPAC Recommendations 2001). *Pure Appl. Chem*. **2001**, *73*, 1795–1818.

[S2] Reed, C. A. H^+, CH_3^+, and R_3Si^+ Carborane Reagents: When Triflates Fail. *Acc. Chem. Res.* **2010**, *43*, 121–128.

[S3] (a) Wu, Q.; Roy, A.; Irran, E.; Qu, Z.-W.; Grimme, S.; Klare, H. F. T.; Oestreich, M. Catalytic Difunctionalization of Unactivated Alkenes with Unreactive Hexamethyldisilane through Regeneration of Silylium Ions. *Angew. Chem., Int. Ed.* **2019**, *58*, 17307–17311. (b) He, T.; Wang, G.; Long, P.-W.; Kemper, S.; Irran, E.; Klare, H. F. T.; Oestreich, M. Intramolecular Friedel–Crafts alkylation with a silylium-ion-activated cyclopropyl group: formation of tricyclic ring systems from benzylsubstituted vinylcyclopropanes and hydrosilanes. *Chem. Sci.* **2021**, *12*, 569–575.

[S4] Wu, Q.; Qu, Z.-W.; Omann, L.; Irran, E.; Klare, H. F. T.; Oestreich, M. Cleavage of Unactivated Si−C(sp^3) Bonds with Reed's Carborane Acids: Formation of Known and Unknown Silylium Ions. *Angew. Chem., Int. Ed.* **2018**, *57*, 9176–9179.

[S5] (a) Danheiser, R. L.; Carini, D. J.; Fink, D. M.; Basak A. Scope and stereochemical course of the (trimethylsilyl)cyclopentene annulation. *Tetrahedron* **1983**, *39*, 935–947. (b) Danheiser, R. L.; Tsai, Y.-M.; Fink, D. M. A General Method for the Synthesis of Allenylsilanes: 1-Methyl-1-(trimethylsilyl)allene. *Org. Synth*. **1988**, *66*, 1.

[S6] Yang, Q.; Draghici, C.; Njardarson, J. T.; Li, F.; Smith, B. R.; Das P. Evolution of an oxidative dearomatization enabled total synthesis of vinigrol. *Org. Biomol. Chem.* **2014**, *12*, 330–344.

[S7] Miura, H.; Doi, M.; Yasui, Y.; Masaki, Y.; Nishio, H.; Shishido, T. Diverse Alkyl–Silyl Cross-Coupling via Homolysis of Unactivated C(sp^3)–O Bonds with the Cooperation of Gold Nanoparticles and Amphoteric Zirconium Oxides. *J. Am. Chem. Soc.* **2023**, *145*, 4613–4625.

[S8] Kjellgren, J.; Sundén, H.; Szabó, K. J. Palladium Pincer Complex Catalyzed Stannyl and Silyl Transfer to Propargylic Substrates: Synthetic Scope and Mechanism. *J. Am. Chem. Soc.* **2005**, *127*, 1787–1796.

[S9] Zuo, H.; Irran, E.; Klare, H. F. T.; Oestreich, M. Electrophilic Activation of S–Si Reagents by Silylium Ions for Their Regio- and Diastereoselective Addition Across C–C Multiple Bonds. *Angew. Chem., Int. Ed.* **2024**, *63*, e202401599.

[S10] Unoh, Y.; Hirano, K.; Miura, M. Metal-Free Electrophilic Phosphination/Cyclization of Alkynes. *J. Am. Chem. Soc.* **2017**, *139*, 6106–6109.

[S11] Liu, Y.; Wang, L.; Deng, L. Selective Double Carbomagnesiation of Internal Alkynes Catalyzed by Iron-*N*-Heterocyclic Carbene Complexes: A Convenient Method to Highly Substituted 1,3-Dienyl Magnesium Reagents. *J. Am. Chem. Soc.* **2016**, *138*, 112–115.

[S12] Sokolov, J.; Lizal, T.; Sindelar, V. Dimeric molecular clips based on glycoluril. *New J. Chem.* **2017**, *41*, 6105–6111.

[S13] Kim, J. H.; Song, T.; Chung, Y. K. Rhodium-Catalyzed Intermolecular

Carbonylative [2+2+1] Cycloaddition of Alkynes Using Alcohol as the Carbon Monoxide Source for the Formation of Cyclopentenones. *Org. Lett.* **2017**, *19*, 1248–1251.

[S14] Kandasamy, M.; Huang, Y.-H.; Ganesan, B.; Senadi, G. C.; Lin, W.-Y. In Situ Generation of Alkynylzinc and Its Subsequent Negishi Reaction in a Flow Reactor. *Eur. J. Org. Chem.* **2019**, 4349–4356.

[S15] Sun, J.-L.; Jiang, H.; Dixneuf, P. H.; Zhang, M. Multicomponent Reductive Coupling for Selective Access to Functional γ-Lactams by a Single-Atom Cobalt Catalyst. *J. Am. Chem. Soc.* **2023**, *14*, 17329–17336.

[S16] Gao, L.; Li, Z. Direct Synthesis of 1-Arylprop-1-ynes with Calcium Carbide as an Acetylene Source. *Synlett* **2019**, *30*, 1580–1584.

[S17] Li, Y.; Liu, D.; Wan, L.; Zhang, J.-Y.; Lu, X.; Fu, Y. Ligand-Controlled Cobalt-Catalyzed Regiodivergent Alkyne Hydroalkylation. *J. Am. Chem. Soc.* **2022**, *144*, 13961–13972.

[S18] Wender, P. A.; Inagaki, F.; Pfaffenbach, M.; Stevens, M. C. Propargyltrimethylsilanes as Allene Equivalents in Transition Metal-Catalyzed [5+2] Cycloadditions. *Org. Lett.* **2014**, *16*, 2923–2925.

[S19] *TURBOMOLE V7.4*, **2019**, a development of University of Karlsruhe and Forschungszentrum Karlsruhe GmbH, 1989–2007, TURBOMOLE GmbH, since 2007; available from http://www.turbomole.com.

[S20] Tao, J.; Perdew, J. P.; Staroverov, V. N.; Scuseria, G. E. Climbing the Density Functional Ladder: Nonempirical Meta-Generalized Gradient Approximation Designed for Molecules and Solids. *Phys. Rev. Lett.* **2003**, *91*, 146401.

[S21] Grimme, S.; Antony, J.; Ehrlich S.; Krieg, H. A consistent and accurate *ab initio* parametrization of density functional dispersion correction (DFT-D) for the 94 elements H-Pu. *J. Chem. Phys.* **2010**, *132*, 154104–154119.

[S22] Grimme, S.; Ehrlich S.; Goerigk L. Effect of the damping function in dispersion corrected density functional theory. *J. Comput. Chem.* **2011**, *32*, 1456–1465.

[S23] Weigend, F.; Häser, M.; Patzelt, H.; Ahlrichs, R. RI-MP2: optimized auxiliary basis sets and demonstration of efficiency. *Chem. Phys. Lett.* **1998**, *294*, 143–152.

[S24] Weigend, F.; Ahlrichs, R. Balanced basis sets of split valence, triple zeta valence and quadruple zeta valence quality for H to Rn: Design and assessment of accuracy. *Phys. Chem. Chem. Phys.* **2005**, *7*, 3297–3305.

[S25] Klamt A.; Schüürmann, G. COSMO: a new approach to dielectric screening in solvents with explicit expressions for the screening energy and its gradient. *J. Chem. Soc, Perkin Trans. 2*, **1993**, 799–805.

[S26] Eichkorn, K.; Weigend, F.; Treutler, O.; Ahlrichs, R. Auxiliary basis sets for main row atoms and transition metals and their use to approximate Coulomb potentials. *Theor. Chem. Acc.* **1997**, *97*, 119–124.

[S27] Weigend, F. Accurate Coulomb-fitting basis sets for H to Rn. *Phys. Chem. Chem. Phys.* **2006**, *8*, 1057–1065.

[S28] Deglmann, P.; May, K.; Furche, F.; Ahlrichs, R. Nuclear second analytical derivative calculations using auxiliary basis set expansions. *Chem. Phys. Lett.* **2004**, *384*, 103–107.

[S29] Grimme, S. Supramolecular Binding Thermodynamics by Dispersion-Corrected Density Functional Theory. *Chem. Eur. J.* **2012**, *18*, 9955–9964.

[S30] Eckert, F.; Klamt, A. Fast solvent screening via quantum chemistry: COSMO-RS approach. *AIChE J.* **2002**, *48*, 369–385.

[S31] Eckert, F.; Klamt, A. COSMOtherm, Version C3.0, Release 16.01; COSMOlogic GmbH & Co. KG, Leverkusen, Germany **2015**.

[S32] Zhao, Y.; Truhlar, D. G. Design of Density Functionals That Are Broadly Accurate for Thermochemistry, Thermochemical Kinetics, and Nonbonded Interactions. *J. Phys. Chem. A* **2005**, *109*, 5656–5667.

[S33] Weigend, F.; Furche, F.; Ahlrichs, R. Gaussian basis sets of quadruple zeta valence quality for atoms H–Kr. *J. Chem. Phys.* **2003**, *119*, 12753–12762.

[S34] Goerigk, L.; Hansen, A.; Bauer, C.; Ehrlich, S.; Najibi, A.; Grimme, S. A look at the density functional theory zoo with the advanced GMTKN55 database for general main group thermochemistry, kinetics and noncovalent interactions. *Phys. Chem. Chem. Phys.* **2017**, *19*, 32184–32215.
